# Discovery of Novel Human Constitutive Androstane Receptor Agonists with the Imidazo[1,2-*a*]pyridine Structure

**DOI:** 10.1021/acs.jmedchem.2c01140

**Published:** 2023-02-09

**Authors:** Ivana Mejdrová, Jan Dušek, Kryštof Škach, Alžbeta Stefela, Josef Skoda, Karel Chalupský, Klára Dohnalová, Ivona Pavkova, Thales Kronenberger, Azam Rashidian, Lucie Smutná, Vojtěch Duchoslav, Tomas Smutny, Petr Pávek, Radim Nencka

**Affiliations:** † Institute of Organic Chemistry and Biochemistry, Czech Academy of Sciences, Flemingovo nám. 2, 166 10 Prague 6, Czech Republic; ‡ Department of Pharmacology and Toxicology, Faculty of Pharmacy in Hradec Kralove, Charles University, Akademika Heyrovskeho 1203, 500 05 Hradec Kralove, Czech Republic; § Czech Centre for Phenogenomics, Institute of Molecular Genetics of the Czech Academy of Sciences, Vídeňská 1083, 142 20 Prague, Czech Republic; ∥ 1st Medical Faculty, Charles University, Katerinska 32, 112 08 Prague, Czech Republic; ⊥ Faculty of Military Health Sciences, University of Defense, Trebeska 1575, 500 01 Hradec Kralove, Czech Republic; # Department of Internal Medicine VIII, University Hospital of Tübingen, 72076 Tübingen, Germany; ¶ School of Pharmacy, Faculty of Health Sciences, University of Eastern Finland, 70211 Kuopio, Finland; ∇ Department of Pharmaceutical and Medicinal Chemistry, Institute of Pharmaceutical Sciences, Eberhard Karls Universität, 72076 Tübingen, Germany

## Abstract

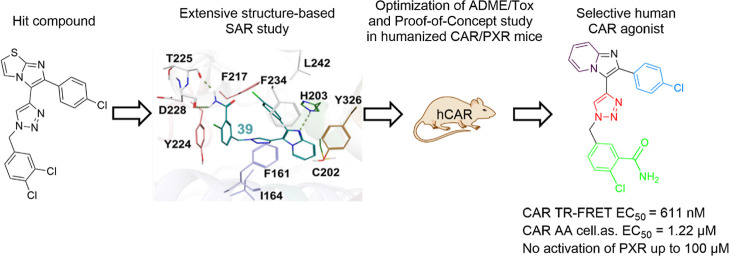

The nuclear constitutive androstane receptor (CAR, NR1I3) plays significant roles in many hepatic functions, such as fatty acid oxidation, biotransformation, liver regeneration, as well as clearance of steroid hormones, cholesterol, and bilirubin. CAR has been proposed as a hypothetical target receptor for metabolic or liver disease therapy. Currently known prototype high-affinity human CAR agonists such as CITCO (6-(4-chlorophenyl)imidazo[2,1-*b*][1,3]thiazole-5-carbaldehyde-*O*-(3,4-dichlorobenzyl)oxime) have limited selectivity, activating the pregnane X receptor (PXR) receptor, a related receptor of the NR1I subfamily. We have discovered several derivatives of 3-(1*H*-1,2,3-triazol-4-yl)imidazo[1,2-*a*]pyridine that directly activate human CAR in nanomolar concentrations. While compound **39** regulates CAR target genes in humanized CAR mice as well as human hepatocytes, it does not activate other nuclear receptors and is nontoxic in cellular and genotoxic assays as well as in rodent toxicity studies. Our findings concerning potent human CAR agonists with in vivo activity reinforce the role of CAR as a possible therapeutic target.

## Introduction

The constitutive androstane receptor (CAR, NR1I3) is a ligand-activated transcription factor belonging to the nuclear receptor subfamily NR1I.

Human CAR is dominantly expressed in hepatocytes. While the endogenous ligands of human CAR are obscure, a number of naturally occurring steroids such as androstanol, androstenol, and 5β-pregnane-3,20-dione have been proposed as endogenous inverse agonists in supraphysiological concentrations.^1,2^ Recent animal studies with a mouse agonist suggest that CAR plays an important role in the metabolism of glucose, lipids, and fatty acids as well as in the endobiotic metabolism of bile acids, cholesterol, bilirubin, and thyroid hormones.^3^ It has been proposed in several independent animal studies that CAR activation may ameliorate glucose homeostasis and insulin sensibility in the treatment of type 2 diabetes.^4,5^ In addition, since CAR activation affects the expression of lipogenic genes in mice, this might also be a promising therapeutic intervention in the treatment of human obesity, steatosis, or hypercholesterolemia,^4,6−9^ although contradictory and species-specific reports also exist.^10−12^ CAR activators have been also proposed as a potential therapy for steatohepatitis or liver regeneration.^13,14^

So far, only two human CAR crystal structures with a human agonist bound have been reported.^15^ The CAR ligand-binding domain (LBD) cavity has a mostly hydrophobic and flexible character with a pocket size of 675 Å.^3,8,16^ The hydrophobic cavity suggests that human CAR ligands are mostly highly lipophilic compounds.

Human CAR displays unique properties in comparison with other nuclear receptors as well as its rodent orthologues. CAR variant 1 (wtCAR, CAR1, and wild-type CAR) exhibits strong constitutive activity that can be further activated by agonists or repressed by inverse agonists. In addition, both direct LBD-dependent and LBD-independent activation are known for CAR.

Human CAR is present in at least three transcript variants (wtCAR, CAR2, and CAR3) in the liver, which differ in their ligand-dependent activation and basal constitutive activities. The wild-type variant CAR (348 AA, NM_005122.4, and transcript variant 3) features high constitutive activity in the regulation of basal expression of target genes and high sensitivity for inverse agonists. This variant represents about 40% of CAR transcripts in the liver parenchyma. The variant CAR3, also called CAR-SV2 (353 AA, XM_005245697.4, transcript variant X4), which has an insertion of the five amino acids APYLT into the LBD, represents 50% of transcripts. CAR3 has low constitutive activity but is highly inducible by ligands and much more active in the upregulation of CAR target genes in the liver. The transcript variant CAR2 (352 AA, NM_001077480.2) is a minor variant with moderate induction activity. The exact physiological functions of the variants are obscure, but several selective activators of individual variants have been described in the literature.^8,17−19^

There are no highly potent, specific, and drug-like (with suitable physicochemical and ADME properties) agonists of the human CAR receptor without off-target effects that can be therapeutically used or can serve as a tool in therapeutic intervention with human CAR ligands. The unique properties of human CAR, mainly its hydrophobic pocket and high constitutive activity, make the discovery of specific ligands difficult.^20^ Therefore, determining suitable drug candidate molecules targeting human CAR and high-affinity endogenous ligands remains problematic.^21^

The only compound known to date is 6-(4-chlorophenyl)imidazo[2,1-*b*]thiazole-5-carbaldehyde *O*-(3,4-dichlorobenzyl)oxime (CITCO, **1**), which is a potent human—but not a mouse—CAR agonist.^22^ However, this highly lipophilic compound also significantly activates the related pregnane X receptor (NR1I2, PXR) of the same subfamily through π–π interactions with the W299 residue.^22−24^ This may exert an unfavorable effect on glycemia and liver steatosis.^25^ On the contrary, the prototype mouse CAR ligand 1,4-bis[(3,5-dichloropyridine-2-yl)oxy]benzene (TCPOBOP) does not activate human CAR.^26^

Different strategies have been used in high-content CAR ligand screenings recently performed, including nuclear translocation assays with an adenoviral-enhanced yellow fluorescent protein-tagged hCAR (Ad/EYFP-hCAR) vector in hepatocytes,^27,28^ mammalian one-hybrid assays using a fusion protein of CAR or its LBD,^21,23,29−31^ and assays employing stable luciferase reporter cell lines expressing wtCAR and treated with an inverse agonist,^32^ as well as with a CAR3-selective screening method combined with other CAR assays.^33^ In addition, studies employing pharmacophore computational modeling and the virtual screening of chemical databases have been performed.^30,34^

**Figure 1:**
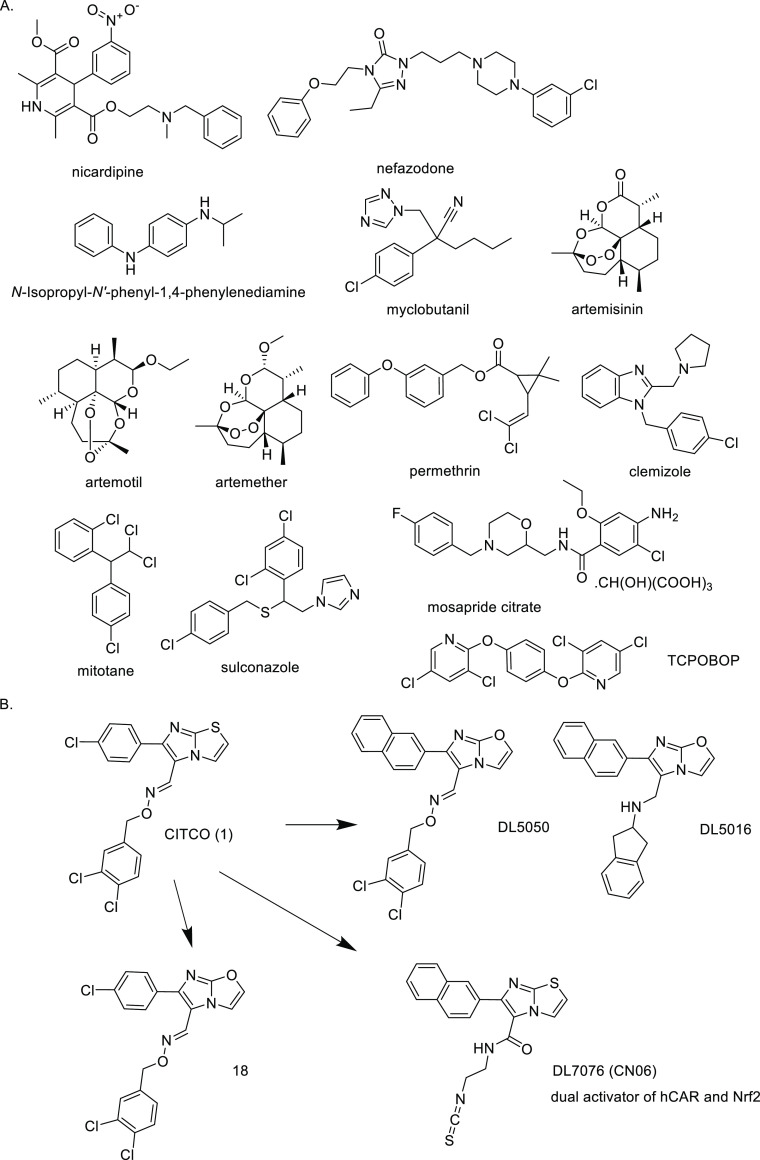
CAR activators discovered by screening chemical libraries (A) or modifications of CITCO as the lead compound (B). TCPOBOP is a mouse CAR ligand.

In the past, several CAR activators with various structural features have been discovered in the screened libraries (Figure 1A) or after modification of the lead compound CITCO (**1**), Figure 1B.^27−29,33−37^

These human CAR ligands, however, still have limited potency to activate human CAR in nanomolar concentrations in comparison with the prototype high-affinity CAR ligand CITCO. Limited studies are currently being undertaken which explore structure–activity relationship variations by systematic synthesis on the human CAR ligand after the initial hit compound discovery or modification of the human CAR agonist CITCO as a template.

Recently, Liang et al. specifically modified the 4-chlorophenyl, imidazothiazole, and 3,4-dichlorphenyl groups of CITCO.^36^ Especially, their discovered compound (*E*)-6-(4-chlorophenyl)imidazo[2,1-*b*]oxazole-5-carbaldehyde *O*-(3,4-dichlorobenzyl)oxime and compound DL5050, ((*E*)-6-(naphthalen-2-yl)imidazo[2,1-*b*]oxazole-5-carbaldehyde *O*-(3,4-dichlorobenzyl)oxime) with the imidazoxazole core exert increased potency and selectivity for human CAR activation over human PXR in a human CAR1-expressing reporter cell line and primary human hepatocytes (PHH).^36^ In an additional study, Liang et al. synthesized a library of CITCO analogues with the 6-(4-chlorophenyl)imidazo[2,1-*b*]oxazole core as well as with modified 4-chlorophenyl or 3,4-dichlorophenyl rings with a variety of substituted arene moieties. In all these novel compounds, the oxime linker of CITCO, which might cause chemical instability, was replaced by groups such as amine, amide, imine, and ether.^37^ In their study, compound DL5016 (*N*-((6-(naphthalen-2-yl)imidazo[2,1-*b*]oxazol-5-yl)methyl)-2,3-dihydro-1*H*-inden-2-amine), which has an EC_50_ value of 0.66 μM in cellular reporter assays, appeared as an efficient and selective human CAR agonist with lower PXR activation than CITCO. In addition, the ligand was shown to induce receptor translocation into the nucleus, to upregulate the expression of the human CAR target gene, and to enhance the efficacy of cyclophosphamide-based cytotoxicity to non-Hodgkin lymphoma cells (Figure 1).^37^ Very recently, DL7076 (CN06) has been discovered as a dual activator of the CAR and nuclear factor erythroid 2-related factor 2 (Nrf2).^38^

Recently, we have described 2-(3-methoxyphenyl)quinazoline derivatives modified at position 4 with 4-methoxy, 4-methylthio, or 4(1*H*)-thione) moieties as potent but nonspecific human CAR ligands also activating PXR and vitamin D receptors.^39^ Similarly, a human CAR agonist FL81 (5-(3,4-dimethoxybenzyl)-3-phenyl-4,5-dihydroisoxazole) discovered by another group also activates the PXR receptor to some extent.^23^

Interestingly, in recent years, several inverse agonists of human CAR have been discovered with IC_50_ in submicromolar/nanomolar concentrations such as PK11195 (1-(2-chlorophenyl)-*N*-methyl-*N*-(1-methylpropyl)-3-isoquinolinecarboxamide),^40^ S07662 (1-[(2-methylbenzofuran-3-yl)methyl]-3-(thiophen-2-ylmethyl) urea),^21^ and CINPA1 ([5-[(diethylamino)acetyl]-10,11-dihydro-5*H*-dibenz[*b*,*f*]azepin-3-yl]carbamic acid ethyl ester).^41^

In the present work, we aimed to discover selective human CAR agonists that do not activate PXR or other nuclear receptors but still possess suitable ADME properties for further experiments in human hepatocyte cellular models or application in humanized CAR mouse models. In a library of kinase inhibitors, we found two lead structures (compounds **2** and **3**) which appeared to be analogues to the known human CAR ligand CITCO.

## Results and Discussion

**Figure 2:**
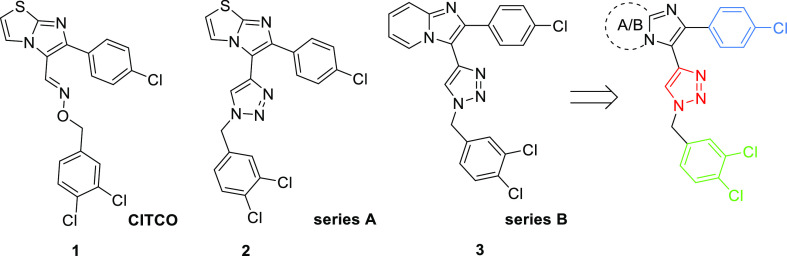
Known human CAR ligand compound **1** (CITCO) and two lead structures (compounds **2** and **3**) with areas of modification in substituted phenyl ring (blue), central heterocyclic linker (red), and substituted benzyl ring (green).

We initially synthesized two analogues **2** and **3** of a known yet unspecific CAR agonist, CITCO, by modifying the middle flexible oxime linker to the triazole ring (Figure 2). The oxime moiety is unstable under acidic conditions, which may complicate its use in vivo. The triazole ring offered stability, less flexibility, and good accessibility via an undemanding click reaction. Because of the synthetic feasibility and possibility to expand the variety of derivatives via a single reaction, the CuAAC reaction has been chosen. Different substitution patterns of blue, green, and red areas allowed us to explore the SAR of the compounds regarding the binding site, the bioavailability of the prepared compounds, and selectivity/specificity toward the key receptors. Our decisions were also based on preliminary docking data (e.g., Figure S-3).

**Figure 3:**
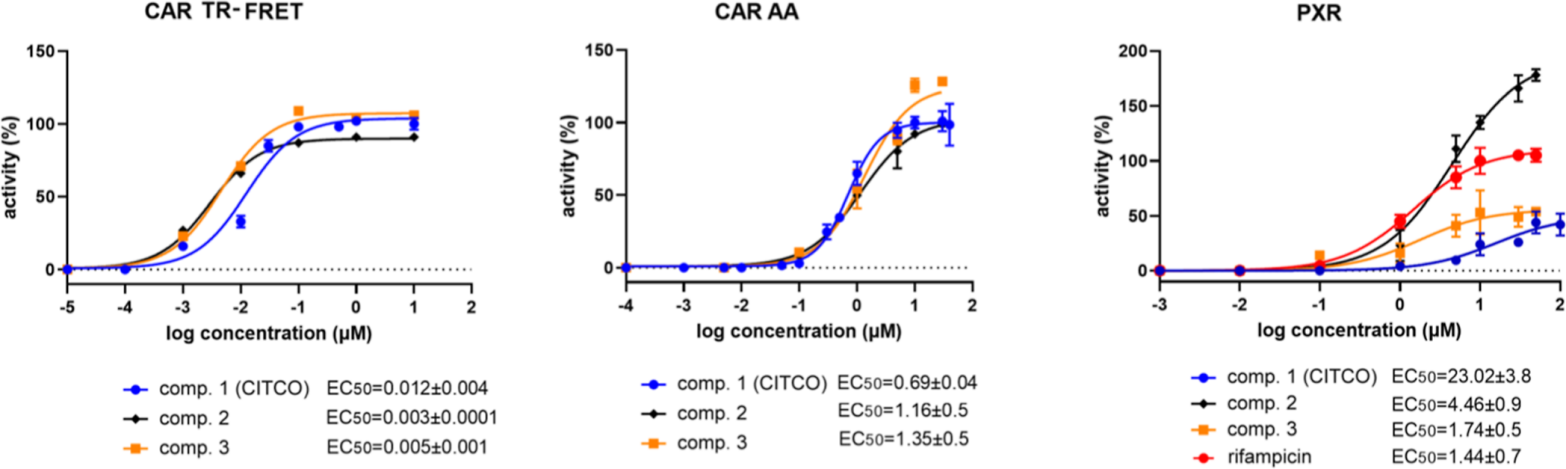
Lead compounds **2** and **3** significantly activate CAR and PXR in the TR-FRET LanthaScreen CAR coactivator assay (CAR TR-FRET), in the CAR LBD assembly assay (CAR AA), or in the PXR-responsive luciferase reporter assay. EC_50_ (in μM) values were obtained based on sigmoidal dose–response fitting. Activities of CITCO and rifampicin, a PXR agonist, at 10 μM are set to be 100%.

We found that analogues **2** and **3** significantly activate both CAR and PXR. Their activities and affinities toward human CAR were similar to those of CITCO in both the recombinant CAR LBD-dependent TR-FRET assay and cellular luciferase reporter assays. Their potency toward PXR was, however, more significant in comparison with the compound CITCO (Figure 3). Compound **2** displayed less cytotoxicity in COS-1 cells than compound **3** (Table S-1).

Compound **2** with the original imidazo[2,1-*b*]thiazole moiety of CITCO and compound **3** with imidazo[1,2-*a*]pyridine moiety were further modified in three key areas: blue (substituted phenyl ring), red (central heterocyclic linker), and green (substituted benzyl ring) (Figure 2).

### Design and Synthesis of the First Generation of Novel CAR Ligands

**Scheme 1:**
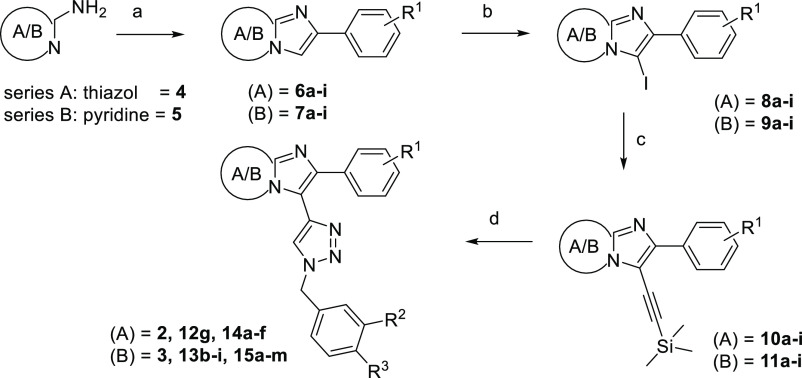
Preparation of the Lead Compounds and Analogues Reagents and conditions: (a) for example, 2-bromo-1-(4-chlorophenyl)ethan-1-one, NaHCO_3_, EtOH, 70 °C, o.n.; (b) NIS, DCM, 25 °C; (c) TMS-acetylene, CuI, TEA, Pd(PPh_3_)_2_Cl_2_, DMF, 0–25 °C; (d) 4-(azidomethyl)-1,2-dichlorobenzene, CuSO_4_·5H_2_O, KF, Na-ascorbate, THF/H_2_O (1:1), 0–25 °C, 1 h.

The first modification of the phenyl and later benzyl ring led to two series A and B based on the compounds **2** and **3**, respectively. The synthesis of the key compounds **2** and **3** as well as their modified analogues with a preserved triazole central heterocyclic linker is illustrated in Scheme 1.

The synthesis of both series started from 2-aminothiazole **4** (for series A) or 2-aminopyridine **5** (for series B). Cyclization with appropriate phenylacetyl chloride with various substitution patterns (Table 1) in EtOH at 70 °C led to 6-substituted imidazo[2,1-*b*]thiazoles **6a–i** or 2-substituted imidazo[1,2-*a*]pyridines **7a–i**. Iodination with NIS in DCM led to the iodinated intermediates **8a–i** and **9a–i**, respectively, in high to quantitative yields. A subsequent Sonogashira reaction with TMS-acetylene under Pd(PPh_3_)_2_Cl_2_ catalysis resulted in compounds **10a–i** and **11a–i**. Lastly, a triazole ring was formed via a CuAAC click reaction, yielding final compounds **2**, **12g**, and **14a–f** and **3**, **13b–i**, and **15a–m** (Table 1).

**Table 1:**
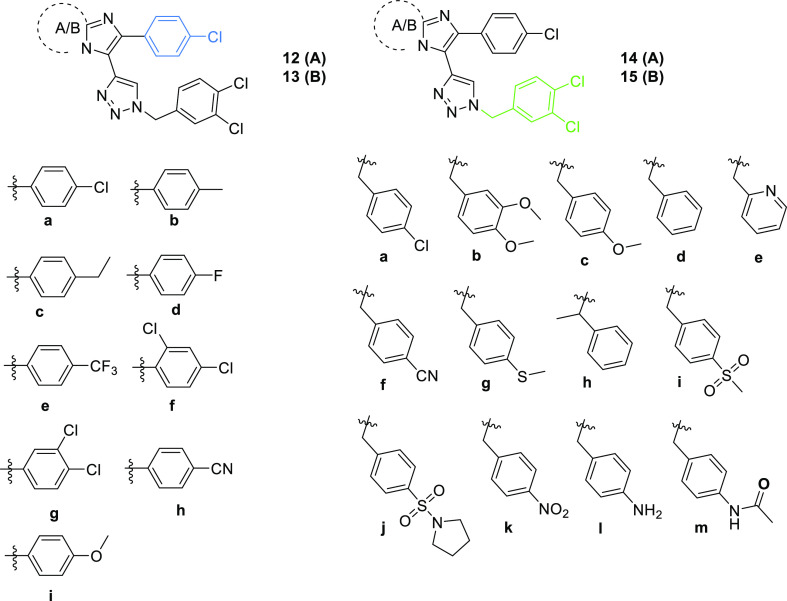
Modification of the Lead Compounds **2** and **3**

comp.	yield^a^ (%)	comp	yield^a^ (%)	comp	yield^a^ (%)	comp	yield^a^ (%)
**2**	90	**13f**	74	**14d**	87	**15f**	82
**12g**	92	**13g**	90	**14e**	80	**15g**	80
**3**	57	**13h**	89	**14f**	87	**15h**	80
**13b**	84	**13i**	92	**15a**	92	**15i**	92
**13c**	70	**14a**	88	**15b**	81	**15j**	93
**13d**	74	**14b**	86	**15c**	75	**15k**	88
**13e**	86	**14c**	86	**15d**	78	**15l**	78
				**15e**	83	**15m**	92

a Yields in % correspond to the last cyclization step after purification.

### Biology

The compounds with a substituted phenyl ring (**12g–13i**) appeared as potent agonists of the CAR with nanomolar EC_50_ in a CAR TR-FRET assay. However, these compounds also significantly activated PXR, and most of them decreased the viability of COS-1 or HepG2 cells (Tables 2 and S-1).

**Table 2:** Effects of Compounds with Phenyl and Benzyl Ring Modification on the Activation of CAR and PXR^a^

Comp.	CAR TR-FRET EC_50_ (μM)	CAR AA EC_50_ (μM)	CAR3 % CITCO activity^b^	PXR % RIF activity^b^
**2**	0.003 ± 0.0001	1.16 ± 0.5	78 ± 4	136 ± 8
**3**	0.005 ± 0.001	1.35 ± 0.5	167 ± 12	53 ± 4
**12g**	0.016	Nd	411 ± 13	57 ± 5
**13b**	0.0003	nd^tox.^	184 ± 21	47 ± 5
**13c**	0.0003	nd^tox.^	nd^tox.^	68 ± 7
**13d**	0.001	nd^tox.^	nd^tox.^	55 ± 4
**13e**	0.001	1.34 ± 0.2	nd^tox.^	42 ± 3
**13f**	0.062	Nd	166 ± 10	128 ± 8
**13g**	0.002	nd^tox.^	323 ± 27	43 ± 4
**13h**	nd^#^	nd^tox.^	nd^tox.^	nd^tox.^
**13i**	<0.001	nd^tox^	nd ^tox.^	89 ± 5
**14a**	0.007	Nd	143	86 ± 10
**14b**	0.432	0.15 ± 0.02	171	111 ± 7
**14c**	0.656	Nd	170	109 ± 10
**14d**	0.007	Nd	91 ± 9	215 ± 21
**14e**	0.01	Nd	109 ± 9	170 ± 13
**14f**	>5	Nd	15 ± 2	87 ± 8
**15a**	0.002	0.05 ± 0.01	78 ± 4	120 ± 10
**15b**	0.019	Nd	123 ± 7	89 ± 9
**15c**	0.040	0.12 ± 0.01	135 ± 11	117 ± 10
**15d**	0.001	0.46 ± 0.02	176 ± 12	195 ± 12
**15e**	1.38	Nd	134 ± 7	212 ± 21
**15f**	0.06	0.12 ± 0.01	33 ± 4	59 ± 8
**15g**	0.011	2.76 ± 0.2	62 ± 5	25 ± 2
**15h**	0.08	0.04 ± 0.007	44 ± 7	68 ± 7
**15i**	0.009	3.05 ± 0.8	18 ± 2	5 ± 0.2
**15j**	no activity	no activity	3 ± 0.3	16 ± 2
**15k**	>5	no activity	33 ± 4	21 ± 3
**15l**	>5	0.50 ± 0.01	73 ± 6	29 ± 4
**15m**	no activity	no activity	12 ± 0.4	20 ± 4
CITCO	0.012 ± 0.004	0.69 ± 0.04		
10 μM			396 ± 25	27 ± 5
1 μM			100%	8 ± 1
Rifampicin 10 μM				100%

a TR-FRET LanthaScreen CAR coactivation assay (CAR TR-FRET), CAR LBD assembly assay (CAR AA), luciferase reporter assay with CAR3 variant, or PXR-responsive luciferase reporter assay were used.

b Compounds were tested at 1 μM (for the CAR3 assay) or at 10 μM for the PXR assay (*n* = 3). nd—not determined due to significant cytotoxicity (nd^tox.^), due to extensive PXR activation or low CAR activation, nd^#^—not determined due to solubility problem and potential precipitation in solution. EC_50_ is the concentration required to achieve half-maximum activation in the TR-FRET Lantha Screen CAR Coactivation assay or the CAR AA assay (in μM).

**Figure 4:**
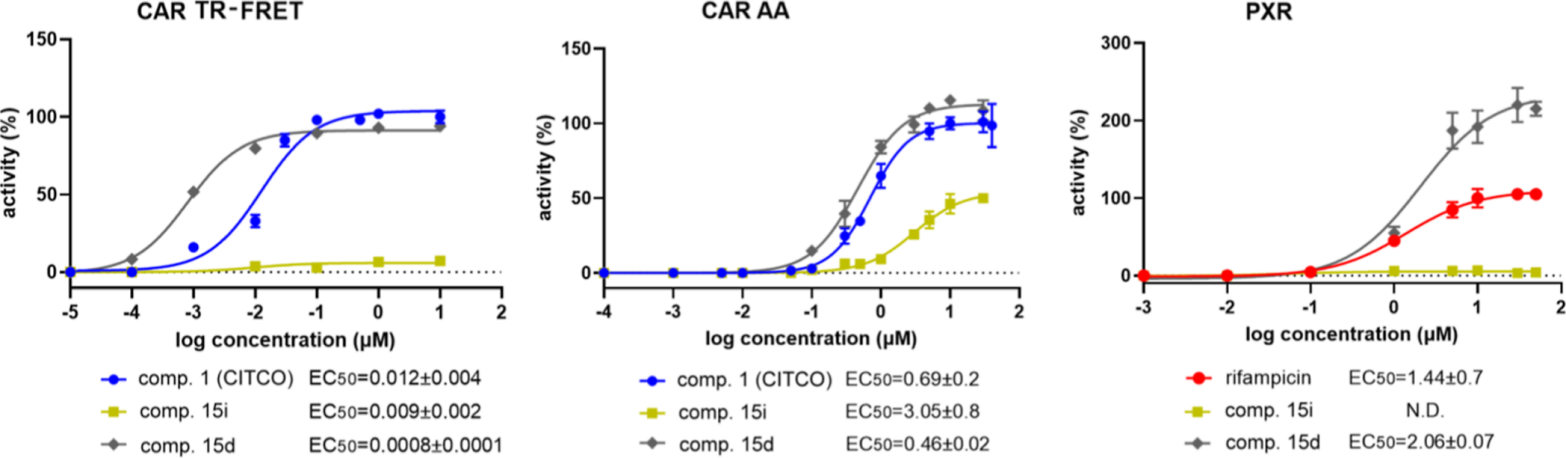
Activities of compounds **15i** and **15d** to stimulate the CAR in the TR-FRET LanthaScreen CAR Coactivator assay (CAR TR-FRET), in the CAR LBD assembly assay (CAR AA), or in the PXR-responsive luciferase reporter assay. EC_50_ values (μM) were obtained based on sigmoidal dose–response curve fitting. Activities of CITCO and rifampicin at 10 μM are set to be 100%.

Compounds **14a–14f** were also found as potent agonists of both CAR and PXR. Similarly, compounds **15a–h** displayed significant activation of CAR and PXR with some moderate effects on cellular viability. Among these compounds, **15d** was found as a highly efficient CAR agonist, while at the same time, it significantly activated PXR with *E*_max_ higher than that of rifampicin (Figure 4 and Table 2). Showing an opposite result, compounds **15i–m** have marginal activities on the CAR and weak activity toward PXR (Table 2).

Compound **15i** appeared to be a selective CAR ligand. However, its activity in the TR-FRET CAR coactivator assay was negligible, and its activity in the CAR LBD assembly assay was weaker compared to CITCO (Figure 4). Thus, compound **15i** demonstrates the phenomenon that some high-potency compounds in the TR-FRET assay with nanomolar EC_50_ but less efficacy in cellular assays are, in fact, partial agonists of the CAR. These compounds do not reach the maximal activity (*E*_max_) of full agonists such as CITCO or compound **15d** (Figure 4). We should also consider the possibility that the tested compounds are likely distributed into cell membranes in cellular assays, which results in lower potency (higher EC_50_ in CAR AA and CAR3 assays) in comparison with the in vitro TR-FRET assay.

In contrast, compound **15j** with a sulfonyl pyrrolidine moiety does not possess any activity to the CAR. We suppose that it is too bulky to fit into the CAR LBD domain (Table 2). We can conclude that the substitution of the phenyl ring with a lipophilic moiety increased activities for both the CAR and PXR. Similarly, lipophilic substitution or no substitution on the benzyl ring increased the nonselective activation of CAR and PXR. Compound **15d** was found as an efficient dual CAR/PXR agonist (Figure 4). Interestingly, compounds **15f** and **15h** displayed high potency for wtCAR in TR-FRET and CAR AA assays (with EC_50_ in the nanomolar range), but they were less potent in the CAR3 assay, suggesting some selectivity for the wtCAR variant over the CAR3 variant (Table 2).

### Design and Synthesis of the Second Generation of Novel CAR Ligands

For the next series of compounds, we focused on the middle heterocyclic linker. The other rings (phenyl and benzyl) maintained the substitution pattern of compounds **2** and **3**. The triazole ring was replaced by several heterocycles with one to three heteroatoms, such as thiadiazol or oxazole. The complete list is shown in Table 3.

**Table 3:**
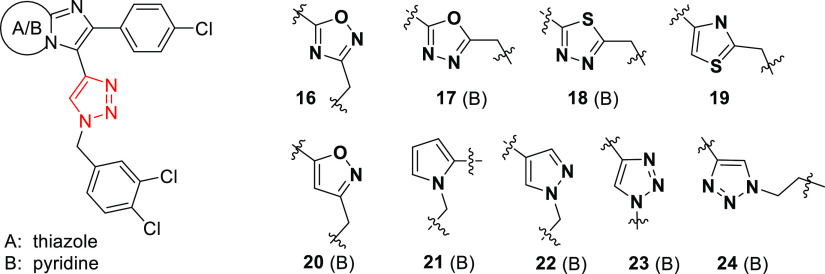
Triazole Ring Modifications

comp.	yield^a^ (%)	comp.	yield^a^ (%)
**16A**	52	**20**	66
**16B**	50	**21**	21
**17**	6	**22**	32
**18**	21	**23**	73
**19A**	61	**24**	95
**19B**	62		

a Yields in % correspond to the last reaction step after purification.

**Scheme 2:**
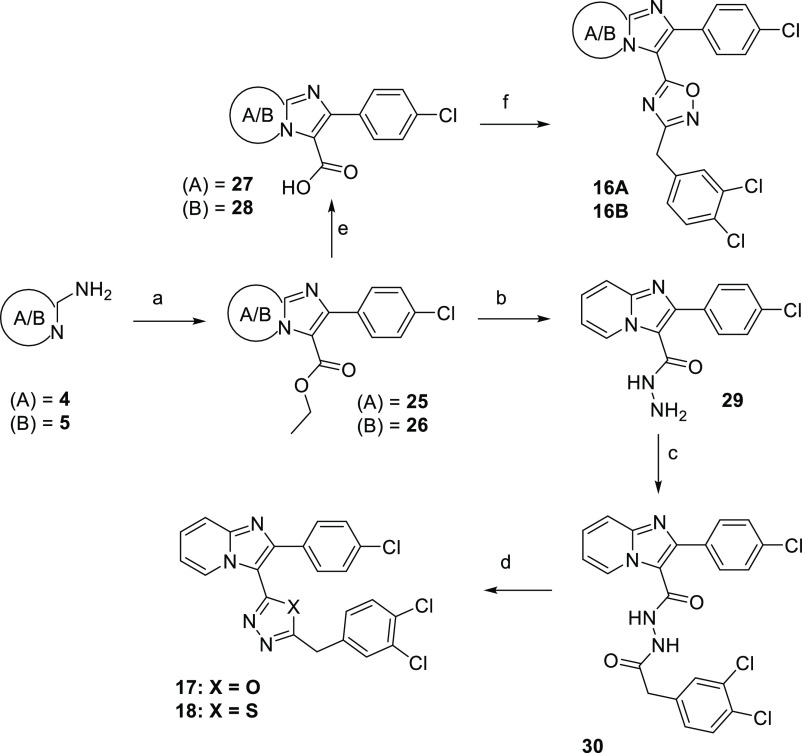
Preparation of Novel Middle-Ring Heterocyclic Analogues Reagents and conditions: (a) ethyl 3-(4-chlorophenyl)-3-oxopropanoate, CBr_4_, CH_3_CN, 80 °C, o.n., 82%; (b) N_2_H_4_·H_2_O (3 equiv), EtOH, reflux, o.n., 87%; (c) ethyl 2-(4-chlorophenyl)imidazo[1,2-*a*]pyridine-3-carboxylate, HATU, DIPEA, DMF, 25 °C, o.n., 90%; (d) tosyl chloride (1.5 equiv), TEA (3 equiv), DCM, 0 °C for **17**, 6%; Lawesson’s reagent (3 equiv), toluene, 100 °C, o.n. for **18**, 21%; (e) LiOH·H_2_O, THF/H_2_O 4:1, 25 °C, 3 h, quant.; (f) (*E*)-2-(3,4-dichlorophenyl)-*N*′-hydroxyacetimidamide, EDC, HOBt, DMF, 25–80 °C, o.n., 52% resp 50%.

Compounds **16A**, **16B**, **17**, and **18** originated from the same precursors **25** or **26**, which were synthesized by a condensation reaction of **4** or **5** with ethyl 3-(4-chlorophenyl)-3-oxopropanoate in the presence of CBr_4_. The ethyl ester moiety of precursors **25** and **26** was hydrolyzed using LiOH·H_2_O, and the obtained acid derivatives **27** and **28** were treated with EDC and HOBt at 25 °C, followed by the addition of substituted *N*′-hydroxyacetimidamide at 80 °C to yield compounds **16A** and **16B** (Scheme 2).

In order to synthesize compounds **17** and **18**, the ester derivative **26** was reacted with an excess of hydrazine hydrate in EtOH, providing compound **29** and further acylated with 2-(3,4-dichlorophenyl)acetic acid by means of the peptide coupling reagent HATU in DMF. Ring-closing reaction of **30** with tosyl chloride at 25 °C or Lawesson’s reagent at 100 °C overnight led to final compounds **17** and **18** respectively, although at very low yields (Scheme 2).

**Scheme 3:**
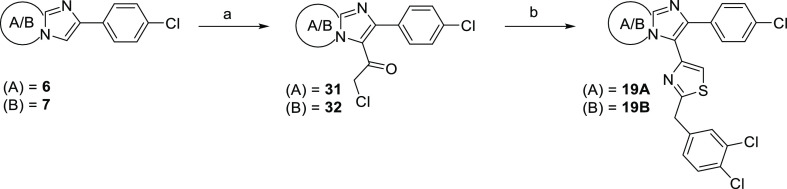
Preparation of Novel Thiazole Analogues of the Lead Compounds Reagents and conditions: (a) chloroacetyl chloride (3 equiv), dioxane, 70 °C, 30 min, 100 °C, o.n., 91% resp 76%; (b) 2-(3,4-dichlorophenyl)ethanethioamide, EtOH, reflux, o.n., 61% resp 62%.

Thiazole analogues **19A** and **19B** were prepared from intermediates **6a** and **7a**, which were reacted with an excess of chloroacetyl chloride in dry dioxane at 70 °C for 30 min and then heated up to 100 °C overnight, followed by cyclization with ethanethioamide (1.5 equiv) in EtOH at reflux (Scheme 3).

**Scheme 4:**
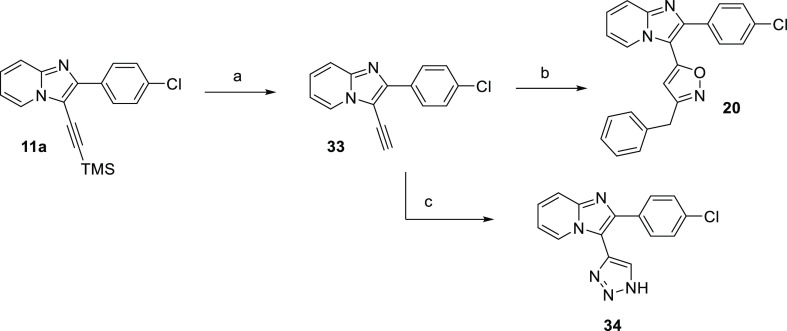
Preparation of Oxazole Derivative **20** and Metabolite M3 (**34**) Reagents and conditions: (a) K_2_CO_3_, MeOH, 25 °C, 2 h, 90%; (b) phenylacetaldehyde, NH_2_OH·HCl, NaOH, H_2_O/*t*-BuOH, CuI, chloramine T, 25 °C, o.n., 66%; (c) TMSN_3_, CuI, DMF/MeOH 10:1, 70 °C, 84%.

Sonogashira reaction of **9a** with TMS acetylene under Pd(PPh_3_)_2_Cl_2_ catalysis provided intermediate **11a**, following deprotection of the TMS group with K_2_CO_3_ in MeOH yielded compound **33**. Pretreatment of phenylacetaldehyde with hydroxylamine hydrochloride and following reaction with intermediate **33** in the presence of chloramine T and CuI at 25 °C provided compound **20**. The click reaction of compound **33** with TMSN_3_ and CuI in a DMF/MeOH mixture under an inert atmosphere led to the unsubstituted triazole derivative **34** (Scheme 4).

**Scheme 5:**
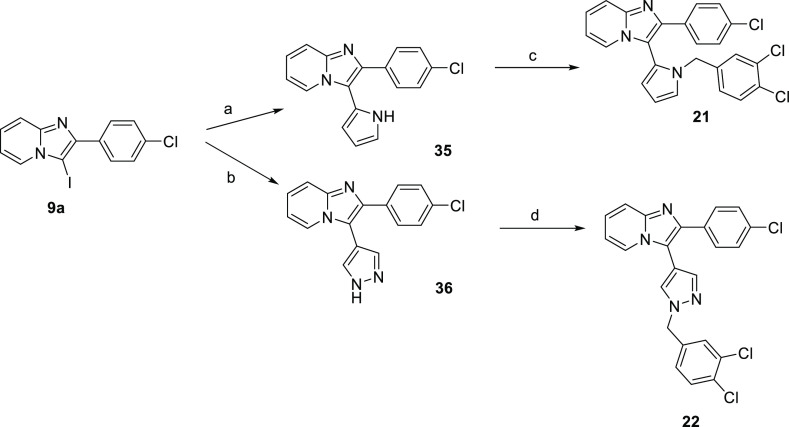
Synthesis of Pyrrole and Pyrazole Derivatives **21** and **22** Reagents and conditions: (a) (1-(*tert*-butoxycarbonyl)-1*H*-pyrrol-3-yl)boronic acid, Pd(PPh_3_)_4_, Na_2_CO_3_, dioxane/H_2_O, 90 °C, o.n., 35%; (b) 4-(4,4,5,5-tetramethyl-1,3,2-dioxaborolan-2-yl)-1*H*-pyrazole, Pd(dppf)Cl_2_, Na_2_CO_3_, dioxane/H_2_O, 90 °C, o.n., 40%; (c) 1,2-dichloro-4-(chloromethyl)benzene, NaH, DMF, 25 °C, o.n., 21%; (d) 1,2-dichloro-4-(chloromethyl)benzene, CH_3_CN, K_2_CO_3_, 25 °C, o.n., 32%.

Pyrrole and pyrazole derivatives (**21** and **22**) were obtained in two-step synthesis starting from iodinated precursor **9a**, which was coupled in a Suzuki reaction under Pd(PPh_3_)_4_ catalysis with (1-(*tert*-butoxycarbonyl)-1*H*-pyrrol-3-yl)boronic acid or 4-(4,4,5,5-tetramethyl-1,3,2-dioxaborolan-2-yl)-1*H*-pyrazole with Pd(dppf)Cl_2_ as a catalyst, respectively, followed by a substitution reaction with benzyl chloride under basic reaction conditions (Scheme 5). Compounds **23** and **24** were synthesized from intermediate **11a** via a click reaction with 4-azido-1,2-dichlorobenzene or 4-(2-azidoethyl)-1,2-dichlorobenzene according to Scheme 1.

### Biology

**Figure 5:**
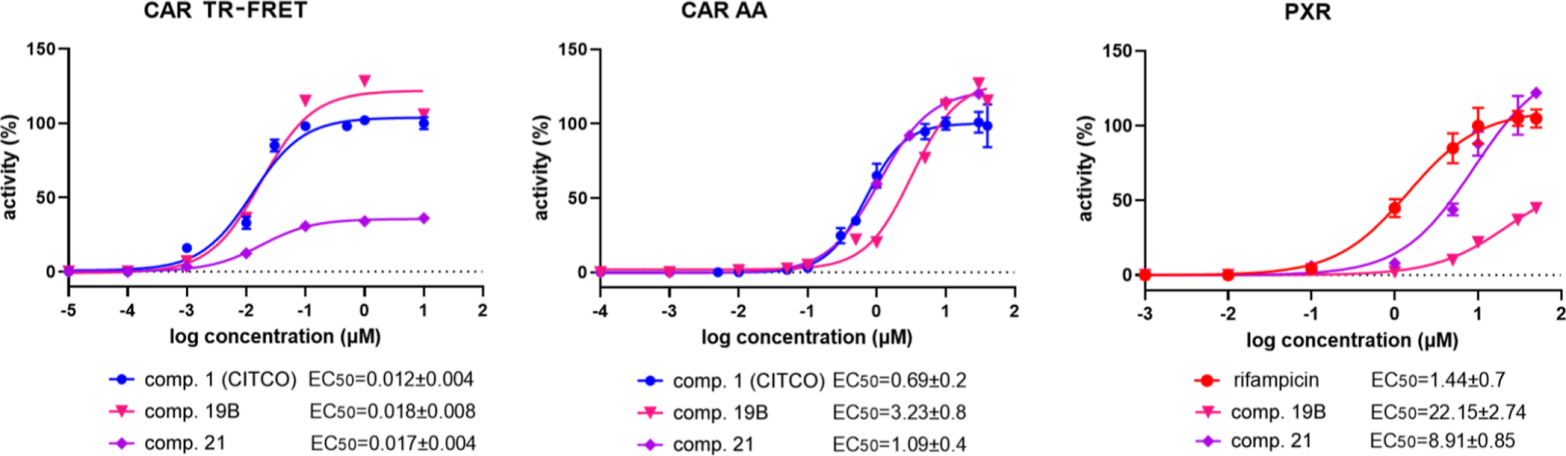
Relative activities of compounds **19B** and **21** to activate CAR in TR-FRET CAR coactivator assays (CAR TR-FRET), in the CAR LBD assembly assay (CAR AA), or the PXR-responsive luciferase reporter assay. EC_50_ values (μM) were obtained based on sigmoidal dose–response curve fitting. Activities of CITCO and rifampicin at 10 μM are set to be 100%.

When we tested the compounds with the modified middle heterocyclic linker, we found that the central moiety also contributes to both CAR and PXR activation, although there were no dramatic variations in the effects of different heterocycles. Out of the series of compounds, compound **19B** showed higher relative selectivity to the CAR as it significantly activates CAR and CAR3, but it tends to activate PXR from 5 μM (Figure 5). Similarly, compound **21** has high activity toward the CAR in the CAR LBD assembly assay, but it has a significant activity to PXR at a 1 μM concentration. Interestingly, in the CAR TR-FRET assay, the compound seems to be a partial agonist with the *E*_max_ lower than that of CITCO or compound **19B** (Table 4, Figure 5).

**Table 4:** Effects of Middle-Ring Heterocyclic Analogues on the Activation of the CAR and PXR^a^

comp.	CAR TR-FRET EC_50_ (μM)	CAR AA EC_50_ (μM)	CAR3% CITCO activity^b^	PXR % RIF activity^b^
**16A**	0.014	>5	22 ± 0.4	6 ± 0.7
**16B**	0.035	2.61 ± 0.4	33 ± 4	7 ± 0.5
**17**	0.023	nd	144 ± 11	56 ± 4
**18**	0.015	8.84 ± 1.15	93 ± 10	20 ± 1
**19A**	0.013	1.16 ± 0.09	231 ± 14	122 ± 10
**19B**	0.018 ± 0.08	3.23 ± 0.8	138 ± 12	22 ± 3
**20**	0.01	0.1 ± 0.04	263 ± 27	130 ± 13
**21**	0.017 ± 0.04	1.09 ± 0.04	29 ± 0.4	88 ± 10
**22**	0.0005	nd^tox.^	nd^tox.^	124 ± 13
**23**	>10	nd	12 ± 2	5 ± 0.4
**24**	0.04	2.8 ± 0.71	82 ± 7	141 ± 10
CITCO	0.012 ± 0.004	0.69 ± 0.2		
10 μM			396 ± 25	27 ± 5
1 μM			100%	8 ± 1
Rifampicin 10 μM				100%

a TR-FRET LanthaScreen CAR Coactivation assay (CAR TR-FRET), CAR LBD assembly assay (CAR AA), luciferase reporter assay with CAR3 variant, or PXR-responsive luciferase gene assay were used.

b Compounds were tested at 1 μM (for CAR3 assay) or at 10 μM for PXR assays (*n* = 3). nd—not determined due to significant cytotoxicity (nd^tox.^), due to extensive PXR activation, or low CAR activation (nd). EC_50_ is the concentration required to achieve half-maximum activation in the TR-FRET LanthaScreen CAR coactivation assay or CAR AA assay (in μM).

No significant cytotoxicity was observed for these compounds in COS-1 and HepG2 cells (Table S-2).

### Design and Synthesis of the Third Generation of Novel CAR Ligands

Since the most promising biological results were found with compound **3** derivatives, in the next step, we addressed the modification of the benzyl ring with the main emphasis on the *meta* position while maintaining the triazole ring. The chlorine atom was replaced with a series of acyl molecules (Table 5). This resulted in improved estimated water solubility and bioavailability of the compounds.

**Table 5:**
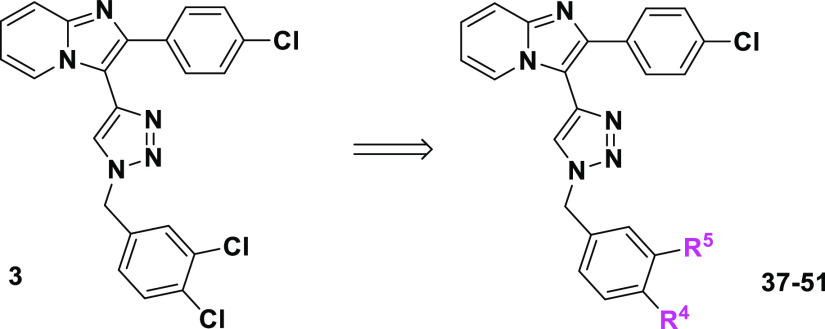

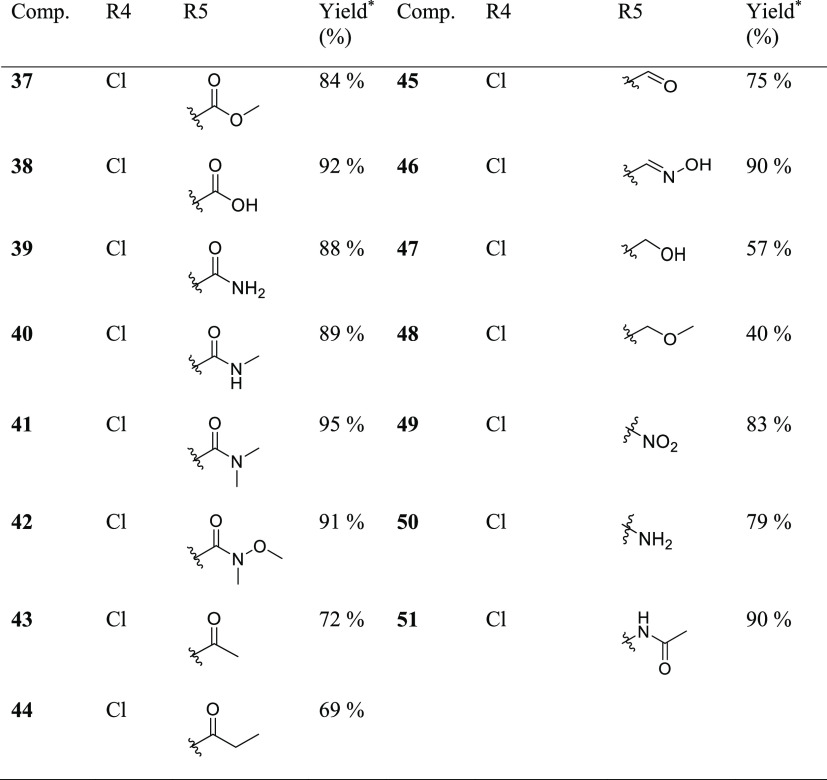
Modification of the Benzyl Ring of the Lead Compound **3**

a Yields in % correspond to the last reaction step after purification.

**Scheme 6:**
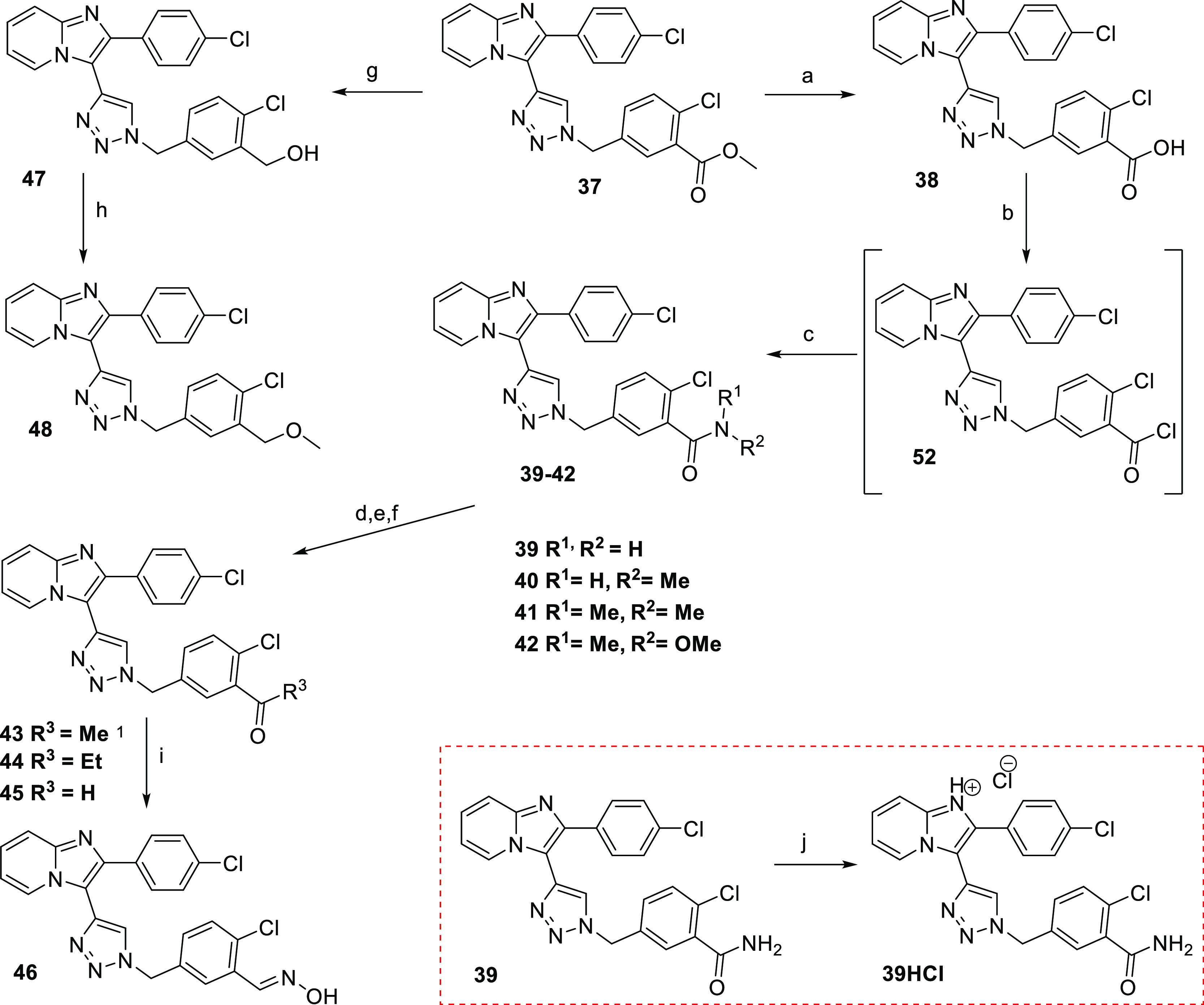
Synthesis of Lead Compound **3** Analogues with Benzyl Ring Modification Reagents and conditions: (a) LiOH·H_2_O, THF/H_2_O, 25 °C, 2 h, 92%; (b) SOCl_2_, toluene; (c) NHR^1^R^2^, DIPEA, 88–95%; (d,e) MeMgBr or EtMgBr, THF, 0–25 °C, 72% resp. 69%; (f) LAH (1 equiv), THF, 5 °C, 2 h, 75%; (g) K_2_CO_3_, MeOH, 25 °C, 2 h; (h) CH_3_I, K_2_CO_3_, DMF, 65%; (i) **45**, NH_2_OH·HCl, DCM, 2 h, 90%; (j) HCl/diethylether, THF, 20 min, 0 °C, quant.

The key compound **37** was prepared by the same means as compound **3** (Scheme 1) with a minor modification in the azide coupling partner. Straightforward hydrolysis led to acid analogue **38**, which was converted to amide analogues **39–42** via acylchloride **52** (Scheme 6).

In order to increase bioavailability, compound **39** was converted to its HCl salt **39** HCl (Scheme 6). The subsequent reaction of *N*-methoxy-*N*-methylbenzamide derivative **42** with a Grignard reagent or LAH at low temperature yielded ketone (**43**,**44**) or aldehyde analogues **45**, which upon the reaction with hydroxylamine provided derivative **46**. Finally, the reduction of the ester derivative with subsequent methylation provided compounds **47** and **48** (Scheme 6).

**Scheme 7:**
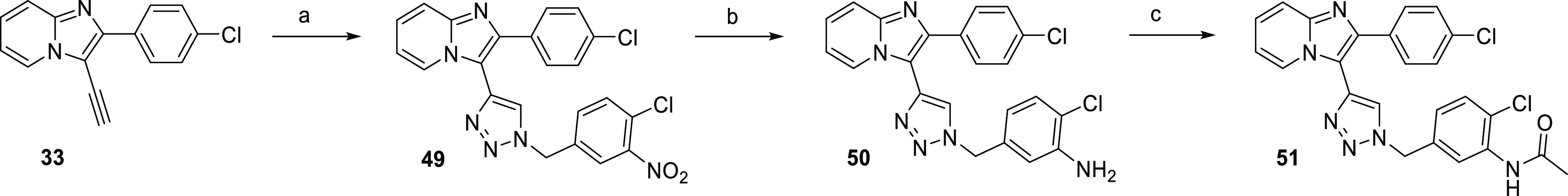
Synthetic Pathway of Nitro, Amino, and Acetylamino Derivates Reagents and conditions: (a) 4-(azidomethyl)-1-chloro-2-nitrobenzene, CuSO_4_·5H_2_O, KF, Na-ascorbate, THF/H_2_O, 25 °C, 1 h, 83% (b) AcOH, Fe, MeOH, reflux, 79%; (c) Ac_2_O, pyridine, dioxane, 25 °C, o.n., 90%.

*N*-acyl derivative **51** was synthesized in two steps from nitro derivative **49** after reduction and the succeeding acylation reaction (Scheme 7).

**Scheme 8:**
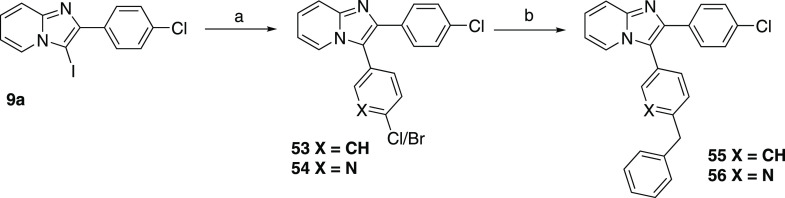
Preparation of Six-Membered Heterocyclic Analogues Reagents and conditions: (a) (4-bromophenyl)boronic acid or (6-chloropyridin-3-yl)boronic acid, dioxane/H_2_O mixture (4:1), Na_2_CO_3_, Pd(dppf)Cl_2_·DCM, 95 °C, o.n., 59% resp 63%; (b) Pd_2_dba_3_, XantPhos, benzylzinc bromide solution 0.5 M in THF, THF, 60 °C, o.n., 72% resp 75%.

Next, we decided to broaden the number of examples of heterocyclic linkers with six-membered heterocycle pyridine and to replace the original triazole with an aryl ring. Syntheses of these compounds started from iodinated precursor **9a**, which was coupled with aryl/pyridyl boronic acid (Scheme 8), followed by the Negishi coupling reaction with benzylzinc bromide catalyzed by Pd, providing compounds **55** and **56**, respectively. Unfortunately, these compounds were barely soluble, so we did not test them.

**Scheme 9:**
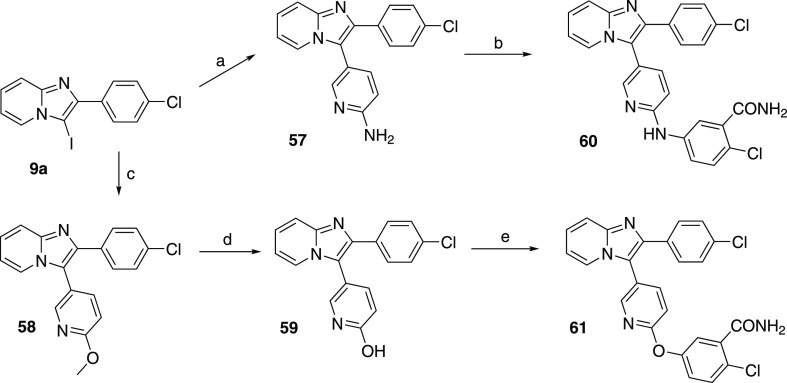
Synthesis of Pyridine Derivatives with a N/O Linker Reagents and conditions: (a) 2-aminopyridine-5-boronic acid pinacol ester, dioxane, Na_2_CO_3_ in 2 mL of H_2_O, Pd(dppf)Cl_2_, 90 °C, o.n., 53%; (b) 5-bromo-2-chlorobenzamide, dioxane, Na*t*BuO, XantPhos, Pd_2_dba_3_, 100 °C, o.n., 51%; (c) (6-methoxypyridin-3-yl)boronic acid, dioxane, Na_2_CO_3_, H_2_O, Pd(dppf)Cl_2_, 90 °C, o.n., 53%; (d) 4 M HCl/dioxane, 95 °C, o.n., 73%; (e) 5-bromo-2-chlorobenzamide, BPPO, CuI, K_3_PO_4_, DMF, 110 °C, o.n., 6%.

Moreover, the methylene part of the linker was exchanged for O and NH. Similarly, to the previously mentioned compounds, intermediate **9a** was coupled with appropriate boronic acid (Scheme 9), providing intermediate compounds **57** and **58** with a free amino group and methoxy group, respectively. Demethylation of compound **58** afforded compound **59** with a free hydroxy group. Both compounds **57** and **59** were coupled with 5-bromo-2-chlorobenzamide by way of Buchwald or Ullmann coupling conditions, yielding compounds **60** and **61** (Scheme 9, Table 6).

**Table 6:** Modification of the Methylene Linker

comp.	R	yield^a^ (%)
**60**	N bridge	51
**61**	O bridge	6

a Yields in % correspond to the last reaction step after purification.

### Biology

**Figure 6:**
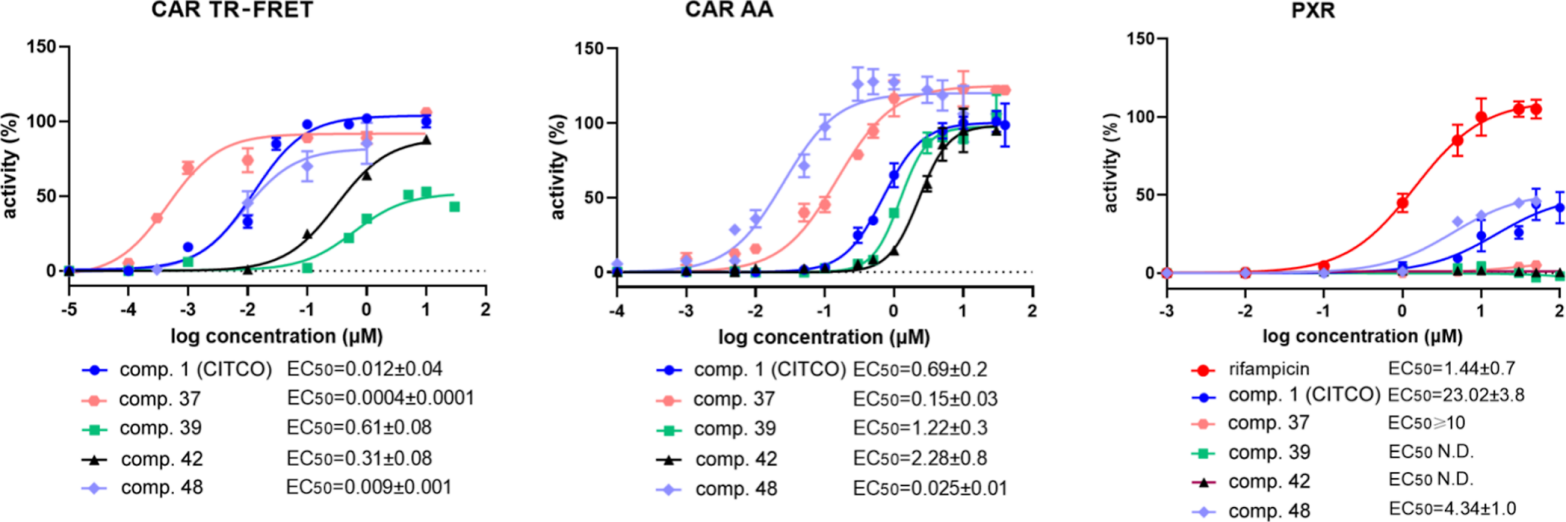
Activation of CAR and PXR in TR-FRET LanthaScreen CAR coactivation assay (CAR TR-FRET), in the CAR LBD assembly assay (CAR AA), or the PXR-responsive luciferase assay. EC_50_ values (μM) were obtained based on sigmoidal dose–response curve fitting. Activities of CITCO and rifampicin at 10 μM are set to be 100%.

When we tested derivatives of compound **3** (Series B) with acyl moieties in the meta position of the benzyl ring with the preserved triazole ring as a linker (**37–44**), we found that the moieties significantly contribute to CAR activation but not to PXR activation. Carboxylic acid itself in the meta position (**38**) resulted in a complete loss of CAR activation. However, amides, as well as esters, significantly activated CAR in all assays. Compound **37** appeared as the most efficient to activate the CAR in the TR-FRET LanthaScreen CAR coactivation assay and highly efficient to activate a CAR LBD assembly assay with an EC_50_ lower than that of CITCO (EC_50_ = 0.4 and 152 nM vs 12 and 690 nM, respectively). Importantly, a methylester (compound **37**), amides (**39**, **40**, and **41**) as well as *N*-methoxy-*N*-methylamide (compound **42**) all have minimal (**37** and **41**) or no activity (**39**, **40**, and **42**) to activate PXR at 10 μM (Figure 6 and Table 7). Other compounds from the set also display low activation of PXR. These compounds were also noncytotoxic in viability assays (Table S-3).

**Table 7:** Activation of CAR and PXR with Benzyl Ring Modification Analogues of Compound **3** in the TR-FRET LanthaScreen CAR Coactivation Assay (CAR TR-FRET), in the Reporter Assay with the CAR3 Variant, in the CAR LBD Assembly Assay (CAR AA), or the PXR-Responsive Luciferase Gene Assay

comp.	CAR TR-FRET EC_50_ (μM)	CAR AA EC_50_ (μM)	CAR3 % CITCO activity^a^	PXR % RIF activity^a^
**37**	0.0004 ± 0.0001	0.148 ± 0.03	137 ± 8	4 ± 0.6
**38**	no activity	nd	14 ± 1	2 ± 3
**39**	0.611 ± 0.08	1.22 ± 0.3	68 ± 4	no activation
**40**	1.686	1.527 ± 0.3	63 ± 4	no activation
**41**	1.066	1.086 ± 0.1	91 ± 8	4 ± 3
**42**	0.313 ± 0.08	2.28 ± 0.8	44 ± 2	no activation
**43**	0.001	nd^tox.^	333 ± 30	30 ± 2
**44**	0.011	0.84 ± 0.04	224 ± 21	26 ± 1
**45**	0.008	<0.1	69 ± 7	43 ± 4
**46**	0.007	0.160 ± 0.01	82 ± 6	29 ± 3
**47**	0.001	nd^tox.^	71 ± 7	77 ± 7
**48**	0.009 ± 0.001	0.025 ± 0.01	345 ± 32	37 ± 4
**49**	0.004	0.060 ± 0.008	59 ± 7	35 ± 4
**50**	0.006	nd^tox.^	70 ± 8	43 ± 4
**51**	0.134	nd^tox.^	97 ± 7	71 ± 6
**60**	no activity	nd^tox.^	8 ± 0.7	23 ± 2
**61**	no activity	no activity	2 ± 0.3	12 ± 1
CITCO	0.012±	0.69 ± 0.2		
10 μM			396 ± 25	27 ± 5
1 μM			100%	8 ± 1
Rifampicin 10 μM				100%

a Compounds were tested at 1 μM (for CAR3 assay) or at 10 μM for PXR assays (*n* = 3). nd—not determined due to significant cytotoxicity (nd^tox.^), due to extensive PXR activation, or low CAR activation (nd). EC_50_ is the concentration required to achieve half-maximum activation in the TR-FRET LanthaScreen CAR coactivation assay or CAR LBD assay (in μM).

When we looked in detail at the CAR agonists **39**, **40**, **41**, and **42** without significant PXR activation, their potencies in the TR-FRET LanthaScreen CAR coactivation assay were by an order of magnitude lower (and EC_50_ higher) than that of CITCO and compound **39** seems to be a partial agonist of the CAR in the assay. In the case of CAR LBD assembly and CAR3 variant assays, these compounds activated the CAR LBD with lower but still comparable affinities in comparison with CITCO. This phenomenon may be explained by the different activation of the CAR LBD by these compounds via another coactivator than with PGC1α, which is involved in the TR-FRET CAR coactivation assay. Indeed, SRC-1 (NCOA1) along with other coactivators are important for the coactivation of CAR^8^ and we can suppose an array of different coactivators in CAR variants activation in cellular assays. We may also suppose the intracellular accumulation of these compounds, for example, via an uptake mechanism, which may increase their potencies in cellular CAR LBD assembly and CAR3 variant assays, but not in the TR-FRET LanthaScreen CAR coactivation assay.

Interestingly, compounds **39**, **40**, and **42** moderately deactivated the PXR-responsive construct in concentrations higher than 10 μM.

Compounds with substitution of the meta position of the benzyl ring with other substituents (**43–51**) and with the preserved triazole ring as a linker retained efficient CAR activation with high potency (EC_50_ below 0.1 μM) (Table 7), although these compounds also activate PXR to some degree. Some of these display substantial effects on cellular viability (Table S-3), which may affect cellular assays. With a methoxyethyl moiety, compound **48** was found to activate the CAR LBD assembly assay with the lowest EC_50_ = 24.9 nM; however, the compound is not selective for the CAR and significantly activates PXR (EC_50_ = 4.34 ± 1 μM). Compound **48** was also highly potent in the activation of the CAR3 variant in the CAR3 variant assay (Table 7). Interestingly, some compounds such as **45** and **47** had high potency for wtCAR in the TR-FRET and CAR AA assays, but they were less potent in the CAR3 assay, suggesting some selectivity for the wtCAR variant.

Compounds **60–61** with a replaced methylene part of the linker with O and NH (Table 6) lost the activity to the CAR and retained a weak activity to PXR.

### Characterization of Induction Properties of Selected Candidates in Human Hepatocyte Models and Their Interactions with Human CAR Variants

**Figure 7:**
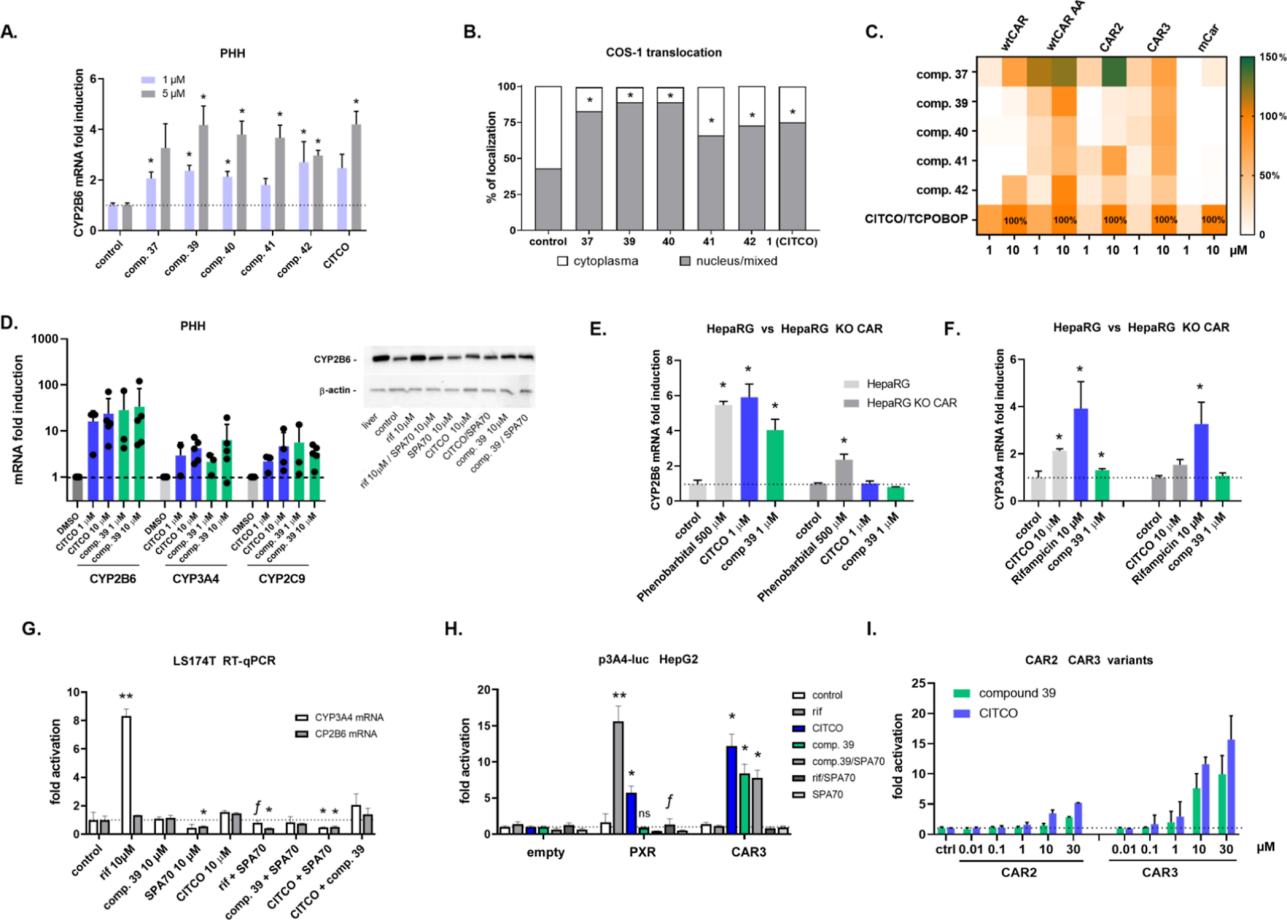
Induction of CAR target genes in primary human hepatocyte models and interactions of selected candidates with CAR transcription variants and mouse CAR. (A) PHHs were treated with compounds **37**, **39**, **40**, **41**, and **42** together with CITCO for 24 h. The expression of *CYP2B6* mRNA, a prototype CAR target gene, was analyzed using RT-qPCR in technical triplicates. Data are presented as mRNA fold induction to control (vehicle-treated) samples. (B) Translocation experiments with EGFP-hCAR + Ala chimera in COS-1 cells treated with tested compounds (10 μM) for 24 h before confocal microscopy. Data are presented as % of cells with specific cytoplasm or mixed/nuclear localization of pEGFP-hCAR + Ala chimeric protein. (C) Interactions of compounds **37**, **39**, **40**, **41**, and **42** with human wtCAR in the CAR LBD assembly assay (wtCAR AA) or with wtCAR inhibited with PK11195 (0.1 μM), with CAR2 or CAR3 variants, or with mouse Car (mCar). (D) PHHs from five donors were treated with compound **39** and CITCO (1 and 10 μM, respectively) for 48 h. CAR target genes *CYP2B6*, *CYP3A4*, and *CYP2C9* mRNA expression have been studied using RT-qPCR. Data are presented as fold induction to control (vehicle-treated) samples. Western blotting experiments with primary human (BioIVT) treated with comp. **39**, rifampicin (rif), CITCO, and PXR antagonist SPA70 (10 μM) for 48 h. Monoclonal anti-CYP2B6 antibody (PA5-35032) was used to detect CYP2B6 protein. (E,F) HepaRG cells and HepaRG KO CAR cells without functional CAR activity were treated with phenobarbital (500 μM), CITCO, rifampicin (10 μM), or compound **39** at a 1 μM concentration for 48 h. *CYP2B6* and CYP3A4 mRNA expression have been analyzed using RT-qPCR. (G) LS174T cells expressing PXR, but without functional CAR, were treated with compound **39**, rifampicin (rif), CITCO, SPA70, or compound **39** at a 10 μM concentration for 48 h. *CYP2B6* and *CYP3A4* mRNA expression has been analyzed using RT-qPCR. (H) Luciferase gene reporter assay with the *CYP3A4* gene promoter construct (p3A4-luc) in HepG2 cells transfected with either PXR or CAR3 expression constructs. Cells were treated for 24 h before analysis. (I) Dose–response activation of CAR2 and CAR3 variants with compound **39** in luciferase reporter gene assays. *p < 0.05 and **p < 0.01-significant *CYP2B6* or *CYP3A4* mRNA upregulation, p3A4-luc activation or EGFP-hCAR + Ala fusion protein nuclear translocation to control samples; *f*-statistically significant effect of SPA70 on rifampicin-mediated *CYP3A4* mRNA expression or activation of the p3A4-luc luciferase construct.

Next, we decided to analyze our novel selective CAR agonists **37**, **39**, **40**, **41**, and **42** to determine whether they could upregulate *CYP2B6* gene mRNA, the typical CAR target gene, in PHH from one donor. We found that all compounds could significantly upregulate *CYP2B6* mRNA. Compounds **39** and **42** tend to be the most potent with a 1 μM concentration in the experiments. These data suggest that the compounds are metabolically stable in metabolically competent hepatocyte cells and that they enter hepatocytes to activate the CAR (Figure 7A).

In translocation experiments with the EGFP-hCAR + Ala chimera, we examined the tested compounds to determine whether they stimulate cytoplasm-to-nuclear translocation of the activated human CAR with extra alanine in the LBD (CAR + A).^42^ We noted that mainly CITCO and compounds **37**, **39**, and **40** significantly decrease the number of cells with specific cytoplasm EGFP-hCAR + Ala localization, and they increase the portion of cells with nuclear localization of the CAR chimera (Figures 7B and S-1). In these experiments, compound **39** appeared as the most promising candidate.

In agreement with cellular assays or induction experiments in PHHs, tested compounds have similar activities in comparison with CITCO (comp. **1**) or compound **37**, which are high-affinity CAR agonists in TR-FRET CAR assays. These data suggest that the cellular environment and signaling have a significant determination on CAR activation.

In the next experiments, we sought to determine whether the discovered selective CAR agonists **37**, **39**, **40**, **41**, and **42** interact with wild-type CAR (wtCAR), human CAR variants 2 (CAR2) and 3 (CAR3), as well as with mouse CAR orthologue in luciferase reporter assays. Efficacy to activate wtCAR was assessed using the CAR LBD assembly assay (CAR AA) or with a wtCAR expression vector that was inhibited with PK111195 (0.1 μM), a known CAR inhibitor. We found that compound **37** is highly efficient in the stimulation of the variant CAR2 and other variants of CAR in comparison with CITCO (100% activity). Compound **39** significantly activated wtCAR in the CAR LBD assembly assay and the CAR3 variant in the gene reporter assay. Its activity in the assay with wtCAR and its inhibitor PK11195, however, was low, suggesting a weak efficacy to compete with the PK111195 inhibitor in the CAR LBD. Other candidate compounds have lower potency in comparison to CITCO (100% activity) in the activation of CAR variants. Compound **42** appeared as a combined agonist of wtCAR and its variants in all assays. Only compound **37** was found to stimulate the mouse CAR when compared to the mouse ligand TCPOBOP (Figure 7C).

In the follow-up studies, we examined the stability of compound **37** in human and mouse microsomes and in plasma. We found that compound **37** is unstable in both mouse and human microsomes with *t*_1/2_ = 4.78 ± 1.31 min and *t*_1/2_ = 6.38 ± 0.67 min, respectively. Importantly, we found that compound **37** is also unstable in mouse plasma as well with *t*_1/2_ = 22.76 ± 0.03 min (Figure S-2).

### Selection of the Candidate for Animal Studies and Detailed Characterization of Compound **39**

In the next experiments, we studied the most efficient compound **39** in five PHHs from five different donors to determine whether it could upregulate *CYP2B6*, *CYP3A4*, and *CYP2C9* mRNA. These genes are significantly, but not exclusively, regulated via the CAR in human hepatocytes. Despite high variability in response in different hepatocyte preparations, we found that compound **39** has similar activity to induce these genes in comparison with CITCO (Figure 7D). Western blotting experiments in PHHs (BioIVT) treated with comp. **39**, rifampicin, CITCO, and PXR antagonist SPA70 (10 μM) for 48 h revealed that compound **39** up-regulates CYP2B6 protein and that the upregulation is not abolished by the PXR antagonist SPA70 (Figure 7D, inserted panel).

To confirm that compound **39** induces *CYP2B6* mRNA via the activated CAR, we performed experiments with HepaRG and its KO CAR counterpart cell line without CAR expression. We observed the upregulation of *CYP2B6* and *CYP3A4* mRNA only in the HepaRG cells but not in the HepaRG KO CAR cells after treatment with both CITCO and compound **39** (Figure 7E,F).

In the next experiments, *CYP2B6* and *CYP3A4* mRNA expression were analyzed in LS174T cells using RT-qPCR. LS174T cells express endogenous PXR but lack functional CAR.^43^ We did not observe any significant induction of these genes by compound **39** in these cells (Figure 7G).

Then, we performed luciferase gene reporter assays with the *CYP3A4* gene promoter construct (p3A4-luc) in HepG2 cells transfected with either PXR or CAR3 expression constructs. Compound **39** activated the luciferase construct only in the presence of CAR3, and the PXR antagonist SPA70 had no significant effect on the activation (Figure 7H).

Finally, we examined the dose–response activation of CAR2 and CAR3 variants with compound **39** (Figure 7I). Unfortunately, the profiles of the dose–response curves did not reach the plateau phase and did not allow us to calculate EC_50_ and *E*_max_ values in the range of concentrations up to 30 μM (Figure 7I).

Based on the data, we can conclude that compound **37** is the most active ligand for all CAR variants. Compound **39** displayed the most significant activity in the induction experiments in PHH and HepaRG cells irrespective of their lower affinities to wtCAR or CAR3 variants in CAR TR-FRET and cellular assays as well as marginal activity toward the CAR2 variant. In addition, we found that compound **39** does not induce *CYP2B6* or *CYP3A4* mRNA via PXR activation.

Next, we considered the physicochemical properties of selected compounds such as molecular weight (*M*_w_), Log *S* (the solubility of a substance, measured in mol/L), and Log *P* (the partition coefficient is a ratio of concentrations of nonionized compound between water and octanol). Compound **39** is the smallest and less lipophilic candidate compound with better-predicted water solubility among the selected candidate compounds (Table 8).

**Table 8:** Physicochemical Properties of Selected Selective CAR Ligands

compound	*M*_w_	log *P*	Log *S*
**37**	478.33	5.76	–7.52
**39**	463.32	4.85	–6.96
**40**	477.35	5.08	–7.22
**41**	491.38	5.32	–7.34
**42**	493.35	5.28	–7.39

Therefore, we decided to use compound **39** in further experiments with other nuclear receptor assays and in humanized CAR mice.

### Novel CAR Ligands Interact with His203 and Occupy a Hydrophobic Pocket in Human wtCAR-LBD

**Figure 8:**
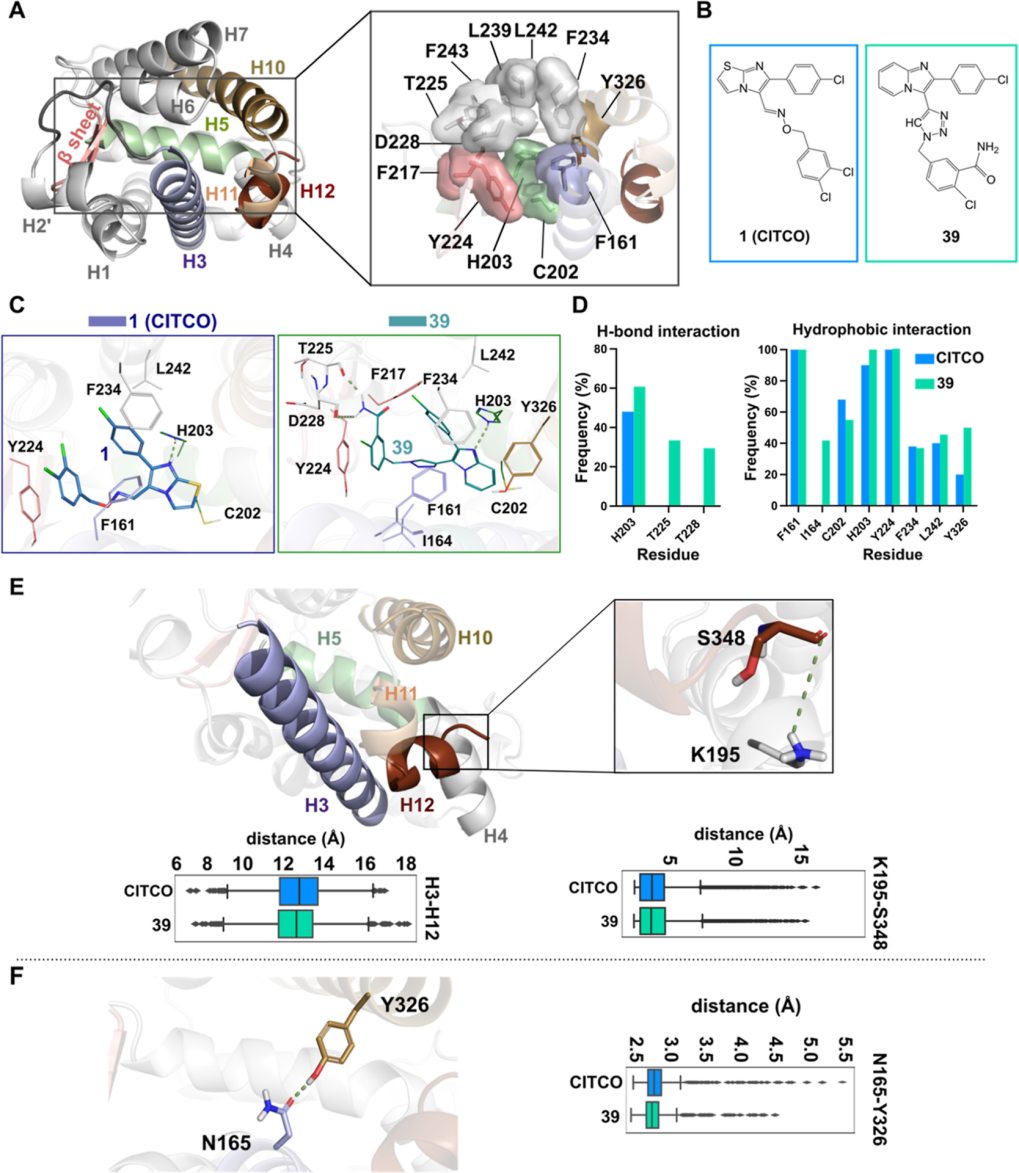
(A) Overview of the CAR LBD structure (wtCAR as the reference structure) and the small-molecule ligand (**39**) used in this study. The regions of interest are highlighted as follows: H2′-H3 loop (residues 140–153), dark gray; H3 loop (residues 157–178), light blue; H5 (residues 196–209), pale green; β sheets (residues 217–223), pink; H10 (residues 308–333), light brown; H11 (residues 336–339), light orange; H12 (residues 341–348), dark brown. The rectangular area denotes the location of the ligand-binding pocket (LBP) and the residues forming the LBP. The main residues participating in ligand binding are depicted in the stick model with a transparent molecular surface. Residues are colored according to their respective regions (see left structure). (B) 2D structure of CITCO and compound **39**. (C) Representative snapshots of LBP with CITCO and compound **39** are shown. The green dashed line represents the hydrogen bond. (D) Frequencies of protein–ligand hydrogen bonds and protein–ligand hydrophobic interactions in percent are shown on the right of the panel. (E) Close view of H3 and H12 zooming in K195 (on H4) and S348 (on H12). The green dashed line represents the hydrogen bond between K195 and S348. Distance between H3 and H12 (center of mass) is represented in the left box plot. Distance between K195 and S348 (oxygen atoms) is represented in the right box plot. The black line in each box represents the median value. (F) Hydrogen bond between the Y326 oxygen atom and N165 polar group is shown as the green dashed line. Color codes are the same as in panel A. Distance between N165 and Y326 (oxygen atoms) is represented in the right box plot.

For the modeling analyses, we docked the compounds **37**, **39**, **40**, and **48** in human wtCAR-LBD using CITCO as a reference (Figure 8A,B). Furthermore, to explore the wtCAR-LBD conformational dynamics and the interactions of these novel compounds, we conducted 25 μs of all-atom MD simulations (5 μs for each system plus CITCO). We studied the differences in the protein–ligand interactions among the systems, in comparison to CITCO. We observed the relevant role of the hydrogen bond interaction of H203 with the phenylimidazole ring in the novel compounds ranging from ∼65 to 90%. This interaction was observed in particular for compounds **39** and **40** for ∼65 and 90%, respectively (Figure 8C,D; Figures S-4–S-7). In addition to H203, T225 and D228 also have a relevant role in compound **39** stabilization. These interactions were formed between the amide of compound **39** and the T225 and D228 backbone oxygen, (located on H6) for ∼35 and 28% of the simulation time, respectively (Figure 8C,D; Figure S-7). It is noteworthy that in the wtCAR/CITCO simulations, these additional polar interactions are not observed.

In addition to the hydrogen bond interactions, all novel compounds show high hydrophobic interaction frequency with F161 (∼100%), the H203 imidazole ring (∼70 to 100%), and Y224 (90–100%, except for the compound **40**, which is around 20%), and lower interaction with C202, F234, Y326, and L242 (no interaction with compound **40**) (Figure 8C,D; Figures S-7 and S-8). These interactions were similarly observed with CITCO. Also, some interactions are compound-specific such as I164 with compound **39**, L206 with compounds **37** and **40**, F217 with compounds **37**, **40**, and **48**, and L239 with compounds **40** and **48**. Overall, we observed that all the novel compounds adopted U-shaped conformations similar to CITCO within the wtCAR-LBD (Figure S-6). This conformation is mainly supported by hydrophobic interactions, with an exclusive interaction for compound **39** with I164, and extra T225 and D228 hydrogen bonds for compound **39**, which stabilizes the compound within human wtCAR-LBD.

### H12 Positioned in Close Vicinity of H3

MDs revealed no direct interaction between CITCO and residues from H12. In this regard, we then proceeded to investigate the changes in geometry and dynamicity of this region relative to the LBD with novel compounds and CITCO. For this purpose, we calculated the distance between H12 and H3 (center of mass of each helix). The result showed that all novel compounds can stabilize the conformation of H12 in the close vicinity of H3 similar to CITCO (Figure 8E, Figure S-9A). This geometry is known to initiate receptor activation.^15^ It has been reported that H12 stays away from the pocket due to the barrier formed by hydrophobic residues in the LBD,^44^ where H11 directs the H12 in this active position.^15^ Previous studies also indicate that the free carboxylate of the H12 C-terminus interacts with the K195 side chain (on H4), leading to further H12 stabilization.^15^ To assess this phenomenon over the simulation time, we next calculated the distance between the carboxylate group of the H12 C-terminus and the polar group of K195 (Figure 8E; Figure S-9B). The median value for this distance in both wtCAR/CITCO and wtCAR/compounds **37**, **39**, and **40** stands around 3.1 Å, with a further distribution with compound **48**. This geometry enables the hydrogen bond formation between the H3 and H12 regions, providing extra stability to the systems. Taken together, this supports our result in terms of the high binding affinity and potency of our novel compounds.

### Further Geometry Stabilization through N165–Y326 Interaction

Along with the closeness of H12 and H3, and the interaction between the H4 and H12 C-terminus, the stabilization of the systems comes through the hydrogen bond interaction between N165 (H3) and Y326 (H10). Both CITCO and **39** show relatively similar rigidity in this region (Figure 8F). The same trends are also observed with other novel compounds (Figure S-8C) with further distribution in the presence of compounds **40** and **48**. Although this interaction has been previously observed in the crystal structure with CITCO,^15^ MD data indicates that it is also relevant for our novel compounds.

Taken together, our docking data followed by microsecond timescale all-atom MD simulations revealed that CITCO and compound **39** interact with wtCAR-LBD mainly by hydrophobic contacts and that stronger polar contacts were formed between compound **39** and wtCAR-LBD compared to CITCO due to hydrogen bond interactions between comp. **39** amide moiety and T225 and D228 backbone oxygen. Interestingly, previous findings report that no specific hydrogen bonds are required for CITCO stability inside the CAR.^18,45^ Analyses of the MD trajectories showed that the interaction between compound **39** and I164 besides the higher interaction frequency with Y326 (hydrophobic interaction) compared to that of the CITCO (Figure 8D) could highlight the critical role of H3 and H10 in protein stabilization. Of note, H10 lies on the heterodimerization interface where RXRα binds to the CAR. Our MD data also revealed that the H12 region is ordered and stable upon ligand binding. This event has been earlier reported as a driving force for CAR constitutive activity^15^ and, therefore, supports the agonistic effect of compound **39**.

### Selectivity of Compound **39** to Other Nuclear Receptors

**Figure 9:**
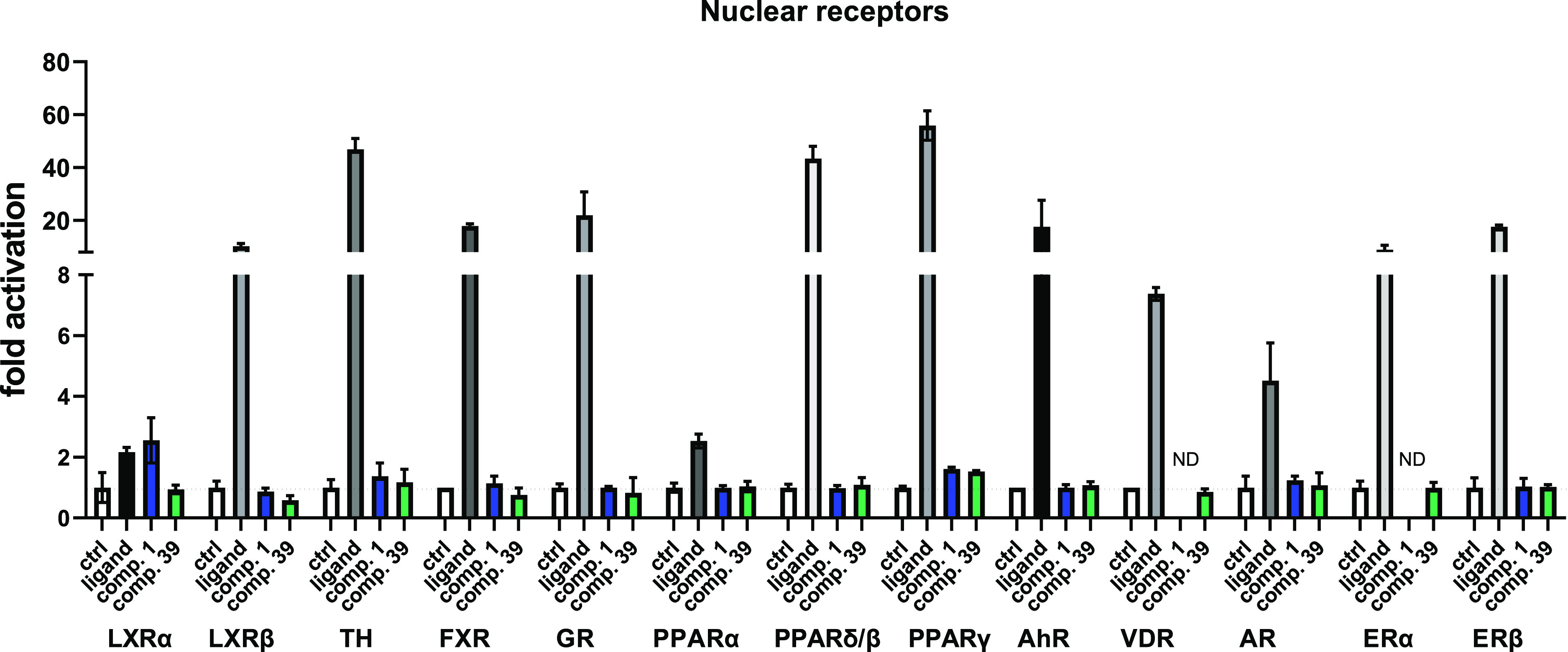
Luciferase reporter assays for human nuclear receptors LXRα, LXRβ, TH, FXR, GR, PPARα, PPARδ/β, PPARγ, VDR, AR, Erα, and ERβ and for the AhR transcription factor were used to confirm the selectivity of compound **39**. Specific ligands (GW3965, thyroxin, obeticholic acid, dexamethasone, fenofibrate, GW501516, rosiglitazone, 3-methylcholantrene, calcitriol, testosterone, and estradiol) have been used in various luciferase reporter assays. Compound **1** (CITCO) and compound **39** have been tested at 10 μM in HepG2 cells treated for 24 h.

Next, we sought to determine whether compound **39** is selective to the human CAR and whether it activates other nuclear receptors, for which a set of luciferase reporter assays was employed. We confirmed the selectivity of compound **39** for CAR as with no other nuclear receptor or the transcription factor aryl hydrocarbon receptor (AhR) was significantly activated by the compound at 10 μM concentration (Figure 9).

### Microsomal Stability Experiments and Pilot Animal Pharmacokinetic Study

**Figure 10:**
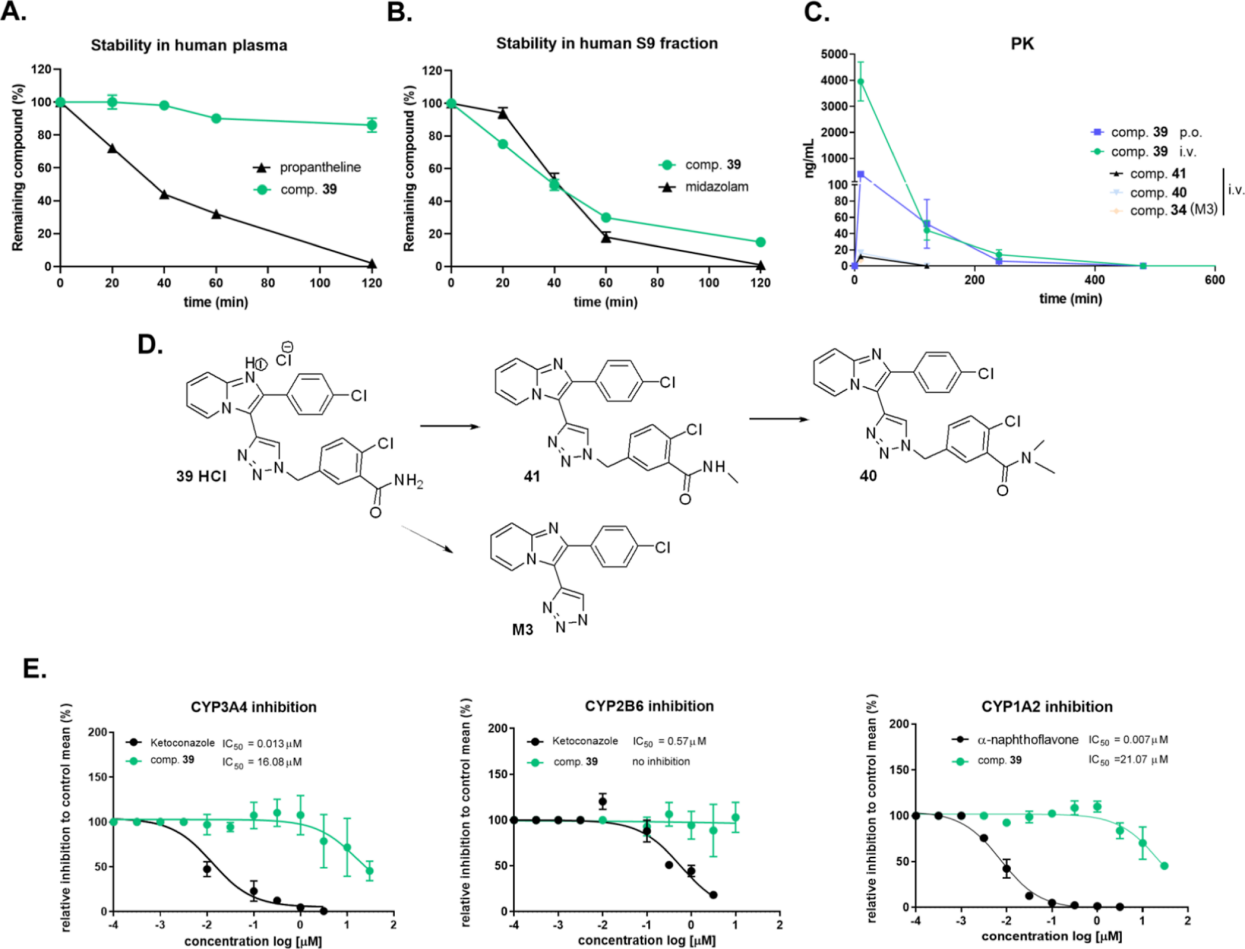
Plasma and microsomal stability experiments and single-dose pharmacokinetics in C57BL/6N mice. The stability of compound **39** in human plasma (A) and human microsomes with S9 fraction (B) were analyzed after 2 h of treatment. (C) Pharmacokinetics (PK) after single-dose application of compound **39** as hydrochloric salt either via i.v. or peroral application (10 mg/kg, *n* = 4) were analyzed in mice over 480 min. (D) Metabolites M1 (comp. **41**), M2 (comp. **40**), and M3 (comp. **34**, 2-(4-chlorophenyl)-3-(1*H*-1,2,3-triazol-4-yl)imidazo[1,2-*a*]pyridine) of compound **39** were observed after i.v. application. Samples have been analyzed using HPLC-MS/MS. (E) Inhibition of CYP3A4, CYP2B6, and CYP1A2 enzymes in microsomes. Compound **39** was tested in the concentration range from 0.1 nM up to 30 μM. Relative activity data were fitted, and dose–response curves were used to obtain IC_50_.

In the following experiments, we evaluated both the plasma and microsomal stability of compound **39** HCl in human plasma, human liver microsomes, as well as liver fraction S9 in time intervals of up to 120 min (Figure 10A,B; Table S-5). We found that compound **39** is highly stable in human plasma (*t*_1/2_ ≥ 240 min). However, we observed a significant decline of compound **39** concentration in human microsomes as well as fraction S9 (*t*_1/2_ = 38.04 min and *t*_1/2_ = 42.4 min, respectively) (Figure 10B; Table S-5).

We also evaluated the plasma protein binding of compound **39** in both human and mouse plasma, determining that 98% of compound **39** is bound to human plasma proteins (Table S-6). We observed very similar properties of compound **39** in mouse plasma and mouse hepatic microsomes (Tables S-5–S-7).

In a pilot single-dose pharmacokinetic study, we found fast absorption of compound **39** HCl hydrochloric salt after p.o. application in gavage, although the compound was rapidly eliminated from the plasma (Figure 10C; Table S-9). Significantly, we detected traces of metabolites for compound **39** after i.v. application in plasma. Metabolites M1 and M2 represent compounds **41** and **40**. Both compounds are N-methylated derivatives of compound **39** with significant CAR activity. Minor metabolite M3 (compound **34**) is 2-(4-chlorophenyl)-3-(1*H*-1,2,3-triazol-4-yl)imidazo[1,2-*a*]pyridine, indicating that hepatic metabolic enzymes may attack the methylene bridge between the heterocycle and phenyl rings (Figure 10D). The metabolite is inactive with respect to the CAR activation, and the metabolite was not observed in human liver microsomes with the S9 fraction (data not shown). These data suggest that compound **39** is the main active compound, and it is likely eliminated intact as the parent compound. Nevertheless, further detailed pharmacokinetic studies should focus on the distribution, biliary elimination, and phase II metabolic clearance of the compound.

In addition, we conducted another examination to determine whether compound **39** inhibits the activities of major human cytochrome P450 enzymes. We found that compound **39** has a minor effect on major cytochrome P450 enzymes. Compound **39** inhibits enzymatic activities of CYP3A4 (with IC_50_ = 16.08 μM) and CYP1A2 (IC_50_ = 21.07 μM) in higher micromolar concentrations, but the compound has no activity on the CYP2B6 enzyme up to 30 μM concentration (Figure 10E).

### Effects of Compound **39** in CAR Humanized Mice

Next, we treated humanized PXR/CAR/CYP3A mice with compound **39** to study the regulation of CAR target genes after a single i.p. application.

**Figure 11:**
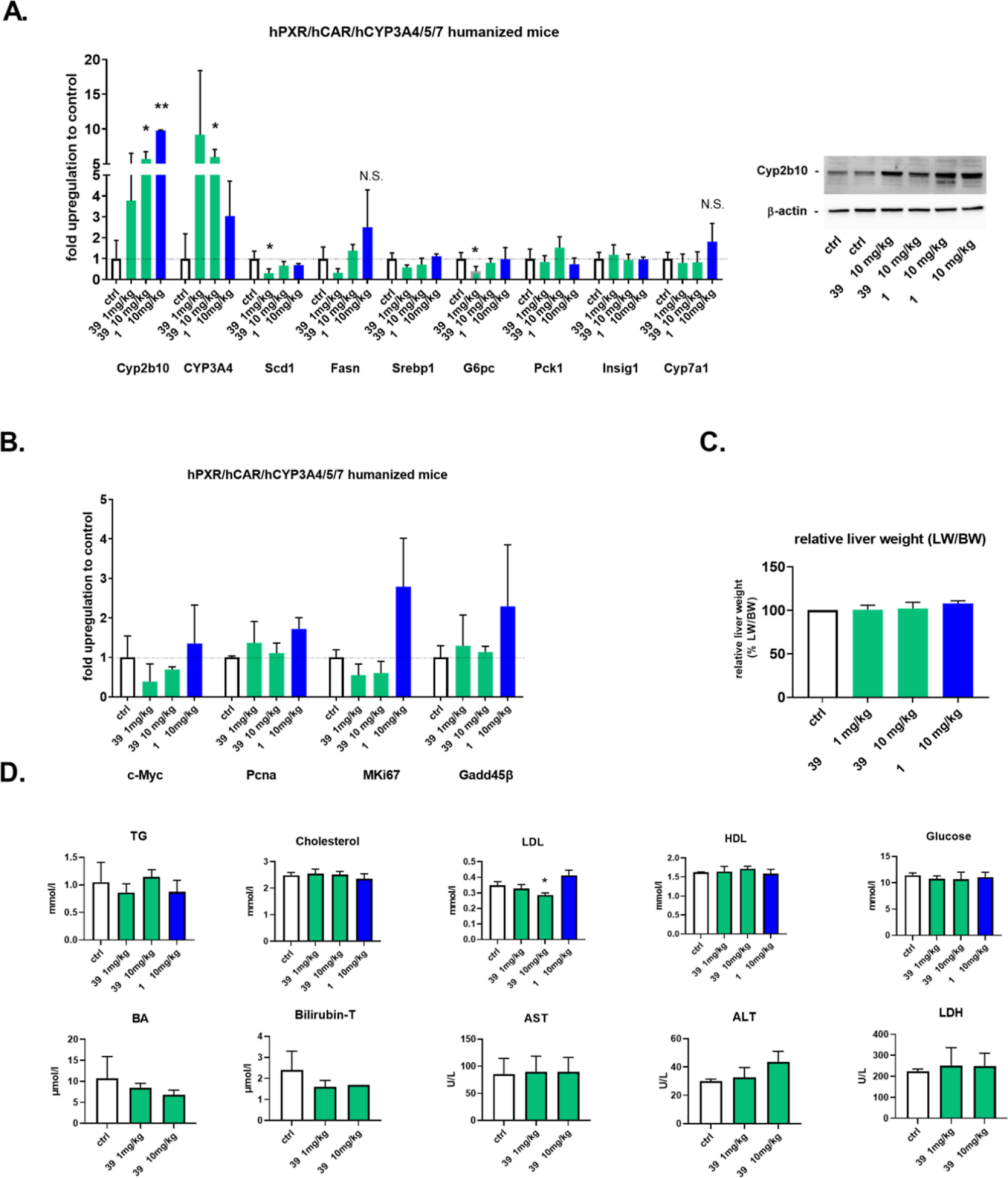
In vivo effects of compound **39** on liver CAR target genes involved in the intermediary metabolism of glucose, lipids and bile acids, hepatocyte proliferation, and apoptosis in humanized PXR/CAR/CYP3A mice (*n* = 4) after single i.p. application of the dose 1 or 10 mg/kg. Mice were sacrificed 36 h after application; livers were subjected to RT-qPCR analysis (A,B), western blotting analysis with anti-Cyp2b10 antibody, or were weighted (C). Blood samples were analyzed for biochemical parameters (D). *p < 0.05-significant effect vs control (vehicle-treated) mice.

We found that compound **39** significantly upregulates *Cyp2b10* mRNA and protein, and human *CYP3A4* mRNA in the humanized model, but significantly decreases the expression of genes *Scd1* and *G6pc* after a single dose of 1 mg/kg. The latter genes are critically involved in triglyceride synthesis and gluconeogenesis in the liver. CITCO appeared more potent to induce *Cyp2b10* mRNA but less potent to upregulate *CYP3A4* mRNA expression, confirming the high efficiency of compound **39** to regulate the key CAR targets genes in murine hepatocytes. We also observed the trend of a decrease of *Srebp1* and *Fasn* mRNA expression after compound **39** application (1 mg/kg) (Figure 11A), which agrees with data observed with the mouse CAR ligand TCPOBOP. This suggests that the human CAR ligand **39** recapitulates the significant effect of the murine ligand TCPOBOP on the regulation of lipid metabolism.^4,5^

We did not observe upregulation of the genes involved in rodent liver proliferation after CAR activation and liver weight gain in the experiments (Figure 11B,C). Nevertheless, long-term studies are needed to examine liver hypertrophy and hyperplasia after repeated treatment with compound **39**.

In analyzing blood biochemistry data after the single-dose application of compound **39** (dose 10 mg/kg), we observed a statistically significant decrease in plasma low-density lipoprotein (LDL) levels. This is consistent with results found with the mouse CAR ligand TCPOBOP in wild-type mice, indicating a positive effect of CAR activation on LDL plasma levels.^9^ We also observed a decrease in bile acid and total bilirubin (bilirubin-T) plasma levels after the application of compound **39**, although these effects were not statistically significant. Neither glucose, plasma triglycerides (TG), HDL lipoproteins, nor liver injury biomarkers (AST, ALT, and LDH) was significantly affected by compound **39** after the single-dose application (Figure 11D).

These results of the pilot single-dose pharmacokinetic study suggest that compound **39** is a novel effective human CAR agonist in animal experiments, a finding which warrants further repeated-dose long-term proof-of-concept studies.

### Toxicity Studies of Compound **39**

We observed no cytotoxicity in HepG2, COS-1 (Table S-3), HepaRG, HepaRG KO CAR, or in the PHHs after 48 h treatment (data not presented). Furthermore, in the Repeated Dose 7 day Oral Toxicity Study in Rodents (EMA/CPMP/ICH/286/1995, 2009 guidelines), no significant signs of toxicity were observed after the 7 days of oral administration of compound **39** HCl into rats. In particular, no significant changes in body weight, changes in behavior, gross pathology, hematology, and biochemistry parameters were observed after the 7 days of oral administration of the compound **39** HCl in all groups (groups with 1, 10, and 30 mg/kg b.w.) when compared to the control group. We also tested the cardiotoxicity of compound **39** in a modified hERG fluorescence polarization assay. We did not observe any binding of compound **39** to hERG up to 20 μM (Supporting Information, Chapter 10).

Finally, we did not observe any frame-shift or base-pair substitution mutagenicity of compound **39** in a modified Ames fluctuation assay performed on *Salmonella typhimuriumTA100* and *TA98* strains at a concentration of 1 and 10 μM (Table S-10).

## Conclusions

Attempts to delineate the therapeutic implications of the CAR in humans have been hindered by the significant overlap in the pharmacology of human CAR and PXR receptors and the lack of a highly selective and potent human CAR agonist with suitable ADME properties.

In this work, we used a rational design of novel selective human CAR agonists. We applied a bioisosteric approach to the central part of the hit molecules **2** and **3** to prepare new ligands for this human nuclear receptor. We were thus able to design a series of novel compounds that differed significantly in both nominal activities as CAR agonists and selectivity toward the PXR receptor as well as enhanced stability in comparison with the model compound CITCO. Based on our results, we performed a careful multiparametric selection of suitable candidates for further pharmacodynamic and pharmacokinetic studies. We found that the imidazo[1,2-*a*]pyridine core with the 1,2,3-triazole linker can be used for the further design of specific human CAR ligands. Replacement of the flexible oxime linker of CITCO with the triazole ring offered stability, less flexibility, and good accessibility via an undemanding click reaction. Modification of the 3,4-dichlorphenyl moiety of the hit compound **3** with amides (analogues **39–42**) resulted in CAR ligands without agonistic activities to PXR (Scheme 6, Table 7, and Figure 6). Although extremely potent CAR agonists emerged in the resulting library of compounds, we also had to consider their metabolic stability and activity toward PXR. As a result, we decided to use compound **39** for further experiments, which, although not among our most potent CAR agonists, exhibited a desirable CAR/PXR profile and reasonable metabolic stability, allowing subsequent in vivo experiments. Using this chemical tool, which we have shown to have no observable toxicity or genotoxic potential, we were able to prove that compound **39** significantly activates the human CAR, both in vitro in human hepatocyte models and CAR humanized mice. Significantly, we noted that compound **39** regulates typical CAR target genes involved in xenobiotic (*Cyp2b10*), lipid (*Scd1*), or glucose (*G6pc*) metabolism, and it decreases plasma LDL lipoproteins even after a single dose in humanized PXR/CAR/CYP3A4/3A7 mice.

In summary, our work identifies a selective CAR receptor agonist for which we have demonstrated both in vitro and in vivo activities in relevant models for the human CAR. Compound **39** thus warrants further preclinical studies in humanized CAR models or human hepatocyte models to better understand the unique function of the human CAR without confounding off-target effects on PXR receptor activation.

## Methods

### Experimental Methods

#### Synthesis of Novel Ligands

General chemical procedures: NMR spectra were measured on a Bruker AVANCE II-600 and/or Bruker AVANCE II-500 instruments (600.1 or 500.0 MHz for ^1^H and 150.9 or 125.7 MHz for ^13^C) in hexadeuterodimethyl sulfoxide and referenced to the solvent signal (δ 2.50 and 39.70, respectively). Mass spectra were measured on a LTQ Orbitrap XL (Thermo Fischer Scientific) using electrospray ionization (ESI) and a GCT Premier (Waters) using EI. The elemental analyses were obtained on a Perkin Elmer CHN Analyzer 2400, Series II Sys (PerkinElmer), and X-ray fluorescence spectrometer SPECTRO iQ II (SPECTRO Analytical Instruments, Germany). Column chromatography and thin-layer chromatography (TLC) were performed using Silica gel 60 (Fluka) and Silufol Silica gel 60 F_254_ foils (Merck), respectively. The purity of newly synthesized compounds was >95%, confirmed by UPLC-MS. Solvents were evaporated at 2 kPa and a bath temperature of 30–60 °C. The compounds were dried at 13 Pa and 50 °C.

#### General Procedure I: Cyclization of Heterocycle

2-Aminothiazole (**3**) or 2-aminopyridine (**4**) was dissolved in EtOH, and substituted or unsubstituted bromoacetophenone derivative (1 equiv) was added, followed by the addition of NaHCO_3_ (1 equiv). The reaction mixture was heated at 70 °C overnight. After the completion of the reaction (monitored by TLC or UPLC), the solvent was evaporated to a minimal volume, and the residue was diluted with EtOAc and washed with water. The water phase was extracted twice more with EtOAc, and the combined organic phases were dried over sodium sulfate and evaporated. The residue was purified by flash column chromatography (eluent petrol ether/EtOAc or EtOAc/MeOH).

#### General Procedure II: Iodination

2-Substituted imidazo[1,2-*a*]pyridines or imidazo[2,1-*b*]thiazoles were dissolved in CH_3_CN (5 mL/mmol) and NIS (1.05 equiv) was added in one portion. The suspension was stirred at 25 °C, and the conversion was monitored by TLC. After the completion of the reaction (1–4 h), the reaction mixture was diluted with EtOAc and washed with a saturated Na_2_S_2_O_3_ solution. The inorganic phase was extracted twice more with EtOAc; combined organic phases were dried over sodium sulfate and evaporated. The residue was purified by flash column chromatography, with the mobile phase petrol ether/EtOAc (10:50%).

#### General Procedure III: Sonogashira Coupling

3-Iodoimidazo[1,2-*a*]pyridines or 5-iodoimidazo[2,1-*b*]thiazoles were placed in a dried round-bottom flask, diluted with dry DMF, degassed at 0 °C, and flushed with argon. CuI (10 mol %) and Pd(PPh_3_)_2_Cl_2_ (5 mol %) were added, the mixture was properly degassed, dry TEA (3 equiv) was added, and the mixture was degassed again. Finally, TMS-acetylene (5 equiv) was added in one portion. The reaction mixture was stirred at 25 °C under an argon atmosphere. After the completion of the reaction (monitored by TLC), the mixture was diluted if necessary with CHCl_3_ and filtered over Celite. The filtrate was washed with water; the water phase was extracted twice more with CHCl_3_; the combined organic phases were dried over sodium sulfate and evaporated. The residue was purified by flash column chromatography (mobile phase petrol ether/EtOAc).

#### General Procedure IV: Click Reaction

Trimethylsilyl(ethynyl)imidazo[2,1-*b*]thiazole or trimethylsilyl(ethynyl)imidazo[1,2-*a*]pyridine derivatives were dissolved in THF/H_2_O mixture (1:1) and an appropriate azido intermediate (1 equiv) was added. The reaction mixture was degassed at 0 °C, refilled with argon, and CuSO_4_·5H_2_O (10 mol %), KF (1 equiv), were Na-ascorbate (1 equiv) were added in one portion. The reaction mixture was stirred at 25 °C and monitored by TLC. After the completion of the reaction, the mixture was diluted with EtOAc and washed with water. The water phase was extracted twice more with EtOAc; combined organic phases were dried over sodium sulfate and evaporated. The residue was purified by flash column chromatography (eluent petrol ether/EtOAc or EtOAc/MeOH).

#### General Procedure V: Ester Hydrolysis

The methyl or ethyl ester derivative was dissolved in THF/H_2_O 2:1, and LiOH·H_2_O (4 equiv) was added in one portion. The reaction mixture was stirred at 25 °C and monitored by TLC. After the completion of the reaction, the mixture was extracted with EtOAc, and the water phase was acidified to pH 2 and extracted again with EtOAc. The organic phase was dried over sodium sulfate and purified by reverse-phase flash column chromatography.

#### General Procedure VI: Amide Preparation

2-Chloro-5-((4-(2-(4-chlorophenyl)imidazo[1,2-*a*]pyridin-3-yl)-1*H*-1,2,3-triazol-1-yl)methyl)-benzoic acid (**38**) was placed in a round-bottom flask, and toluene was added (5 mL) followed by the addition of thionyl chloride (0.5 mL, in excess). The reaction mixture was stirred at 90 °C overnight. The reaction mixture was evaporated to dryness, coevaporated with toluene, and used directly for the next step without any purification. The intermediate 2-chloro-5-((4-(2-(4-chlorophenyl)imidazo[1,2-*a*]pyridin-3-yl)-1*H*-1,2,3-triazol-1-yl)methyl)benzoyl chloride (**53**) was dissolved in dry DCM and cooled in an ice bath. An appropriate amine (1.2 equiv) was added, followed by the addition of DIPEA (1.5 or 2 equiv in case of amine salts). The reaction mixture was stirred at 25 °C and monitored by TLC or LCMS. After completion of the reaction, the mixture was diluted with DCM, washed with water, and purified by reverse-phase flash CC or flash column chromatography.

#### General Procedure VII: Ketone Preparation Using the Grignard Reagent

2-Chloro-5-((4-(2-(4-chlorophenyl)imidazo[1,2-*a*]pyridin-3-yl)-1*H*-1,2,3-triazol-1-yl)methyl)-*N*-methoxy-*N*-methylbenzamide was dissolved in dry THF (6 mL), cooled in an ice bath, degassed, and refilled with argon. The Grignard reagent (3 M in DEE, 2 equiv) was added in one portion, and the mixture was allowed to warm to 25 °C and stirred overnight. For the LAH reduction in case of an aldehyde, the mixture was stirred at 5 °C for 2 h. The mixture was quenched with a saturated NH_4_Cl solution and extracted with EtOAc. Combined organic phases were dried over sodium sulfate, evaporated, and purified if necessary by flash column chromatography (petrol ether/EtOAc 70:100%).

##### 6-(4-Chlorophenyl)-5-(1-(3,4-dichlorobenzyl)-1*H*-1,2,3-triazol-4-yl)imidazo[2,1-*b*]thiazole (**2**)

The title compound was prepared according to General Procedure IV. Mobile phase petrol ether/EtOAc (20:50%). Yield: 268 mg (90%). ^1^H NMR (500 MHz, DMSO-*d*_6_): δ 8.49 (s, 1H), 8.04 (d, *J* = 4.5 Hz, 1H), 7.67–7.70 (m, 4H), 7.42–7.45 (m, 2H), 7.39 (d, *J* = 4.5 Hz, 1H), 7.34 (ddm, *J* = 2.1 Hz, *J* = 8.3 Hz, 1H), 5.69 (br s, 2H). ^13^C NMR (101 MHz, DMSO): δ 149.63, 142.82, 137.13, 136.99, 133.30, 132.39, 131.49, 131.24, 131.19, 130.42, 129.30, 128.73, 128.66, 123.62, 120.02, 114.52, 113.92, 51.85. HRMS: calcd for [M + H], 459.99518; found, 459.99522.

##### 2-(4-Chlorophenyl)-3-(1-(3,4-dichlorobenzyl)-1*H*-1,2,3-triazol-4-yl)imidazo[1,2-*a*]pyridine (**3**)

The title compound was prepared according to General Procedure IV. Mobile phase petrol ether/EtOAc (20:70%). Yield: Yield: 130 mg (57%). ^1^H NMR (500 MHz, DMSO-*d*_6_): δ 8.53 (s, 1H), 8.48 (dt, *J* = 1.1 Hz, *J* = 6.9 Hz, 1H), 7.67–7.71 (m, 5H), 7.41–7.44 (m, 2H), 7.39 (ddd, *J* = 1.2 Hz, *J* = 6.8 Hz, *J* = 9.1 Hz, 1H), 7.35 (dd, *J* = 2.1 Hz, *J* = 8.4 Hz, 1H), 7.00 (td, *J* = 1.2 Hz, *J* = 6.8 Hz, 1H), 5.75 (s, 2H). ^13^C NMR (101 MHz, CDCl_3_): δ 144.95, 142.52, 137.05, 136.40, 133.04, 132.86, 131.53, 131.28, 131.21, 130.34, 129.67, 128.71, 128.59, 126.30, 125.95, 125.45, 117.13, 113.38, 111.57, 51.94. HRMS: calcd for [M + H], 454.03876; found, 454.03886.

##### 6-(4-Chlorophenyl)imidazo[2,1-*b*]thiazole (**6a**)

The title compound was prepared according to General Procedure I. Mobile phase petrol ether/EtOAc (20:60%). Yield: 981 mg (82%). ^1^H NMR (401 MHz, DMSO-*d*_6_): δ 8.26 (s, 1H), 7.94 (d, *J* = 4.4 Hz, 1H), 7.90–7.80 (m, 2H), 7.48–7.39 (m, 2H), 7.28 (d, *J* = 4.4 Hz, 1H). 13C NMR (101 MHz, DMSO): δ 149.88, 145.54, 133.64, 131.78, 129.09, 126.85, 120.52, 113.85, 110.30. EI MS: calcd for [M + H], 234.0018; found, 234.0020.

##### 6-(3,4-Dichlorophenyl)imidazo[2,1-*b*]thiazole (**6g**)

The title compound was prepared according to General Procedure I. Mobile phase petrol ether/EtOAc (20:50%). Yield: 878 mg (65%). ^1^H NMR (401 MHz, DMSO-*d*_6_): δ 8.37 (s, 1H), 8.06 (d, *J* = 2.0 Hz, 1H), 7.97 (d, *J* = 4.5 Hz, 1H), 7.82 (dd, *J* = 8.4, 2.1 Hz, 1H), 7.64 (d, *J* = 8.4 Hz, 1H), 7.30 (d, *J* = 4.5 Hz, 1H). ^13^C NMR (101 MHz, DMSO): δ 149.83, 143.93, 135.18, 131.65, 131.04, 129.21, 126.38, 124.93, 120.26, 113.98, 110.90. HRMS: calcd for [M + Na], 268.97015; found, 268.97029.

##### 2-(4-Chlorophenyl)imidazo[1,2-*a*]pyridine (**7a**)

The title compound was prepared according to General Procedure I. Mobile phase petrol ether/EtOAc (20:60%). Yield: 1.49 g (88%). ^1^H NMR (401 MHz, chloroform-*d*): δ 8.12 (dt, *J* = 1.2 Hz, *J* = 6.8 Hz, 1H), 7.93–7.87 (m, 2H), 7.84 (d, *J* = 0.7 Hz, 1H), 7.64 (dq, *J* = 1.0 Hz, *J* = 9.2 Hz, 1H), 7.44–7.40 (m, 2H), 7.20 (ddd, *J* = 1.3 Hz, *J* = 6.8 Hz, *J* = 9.2 Hz, 1H), 6.80 (td, *J* = 1.2 Hz, *J* = 6.8 Hz, 1H). ^13^C NMR (101 MHz, CDCl_3_): δ 145.86, 144.82, 133.79, 132.45, 129.02, 127.38, 125.73, 125.03, 117.69, 112.72, 108.31. EI MS: calcd for [M + H], 228.0454; found, 228.0456.

##### 2-(*p*-Tolyl)imidazo[1,2-*a*]pyridine (**7b**)

The title compound was prepared according to the described procedure. The identity and purity were confirmed by NMR and HRMS. Mobile phase petrol ether/EtOAc (20:70%). Yield: 853 mg (76%).

^1^H NMR (401 MHz, DMSO-*d*_6_): δ 8.50 (dt, *J* = 6.8, 1.2 Hz, 1H), 8.33 (d, *J* = 0.7 Hz, 1H), 7.88–7.82 (m, 2H), 7.56 (dq, *J* = 9.1, 1.0 Hz, 1H), 7.27–7.19 (m, 3H), 6.87 (td, *J* = 6.7, 1.2 Hz, 1H), 2.33 (s, 3H). ^13^C NMR (101 MHz, DMSO): δ 145.21, 144.96, 137.43, 131.62, 129.75, 127.24, 125.97, 125.23, 116.99, 112.61, 109.10, 21.33. HRMS: calcd for [M + H], 209.10732; found, 209.10745.

##### 2-(4-Ethylphenyl)imidazo[1,2-*a*]pyridine (**7c**)

The title compound was prepared according to General Procedure I. Mobile phase petrol ether/EtOAc (20:70%). Yield: 1.02 g (74%). ^1^H NMR (401 MHz, DMSO-*d*_6_): δ 8.51 (dt, *J* = 6.8, 1.2 Hz, 1H), 8.34 (d, *J* = 0.7 Hz, 1H), 7.90–7.85 (m, 2H), 7.56 (dq, *J* = 9.1, 1.0 Hz, 1H), 7.29–7.26 (m, 2H), 7.23 (ddd, *J* = 9.1, 6.7, 1.3 Hz, 1H), 6.88 (td, *J* = 6.7, 1.2 Hz, 1H), 2.63 (q, *J* = 7.6 Hz, 2H), 1.21 (t, *J* = 7.6 Hz, 3H). ^13^C NMR (101 MHz, DMSO): δ 145.21, 144.97, 143.79, 131.88, 128.56, 127.25, 126.04, 125.24, 117.00, 112.63, 109.14, 28.44, 16.01. HRMS: calcd for [M + H], 223.12298; found, 223.12301.

##### 2-(4-Fluorophenyl)imidazo[1,2-*a*]pyridine (**7d**)

The title compound was prepared according to General Procedure I. Mobile phase petrol ether/EtOAc (20:70%). Yield: 988 mg (88%). ^1^H NMR (401 MHz, DMSO-*d*_6_): δ 8.52 (dt, *J* = 1.2, 7.0 Hz, 1H), 8.38 (s, 1H), 8.04–7.96 (m, 2H), 7.57 (d, *J* = 9.1 Hz, 1H), 7.31–7.21 (m, 3H), 6.89 (td, *J* = 1.3, 6.8 Hz, 1H). ^13^C NMR (101 MHz, DMSO): δ 145.30, 143.91, 130.93, 127.37, 125.51, 117.06, 112.80, 109.41. ^13^C NMR (101 MHz, DMSO-*d*_6_): δ 162.29 (d, *J* = 244.2 Hz), 130.93, 127.97 (d, *J* = 8.2 Hz), 116.05 (d, *J* = 21.5 Hz). HRMS: calcd for [M + H], 213.08225; found, 213.08226.

##### 2-(4-(Trifluoromethyl)phenyl)imidazo[1,2-*a*]pyridine (**7e**)

The title compound was prepared according to General Procedure I. Mobile phase petrol ether/EtOAc (20:70%). Yield: 1.07 g (77%). ^1^H NMR (401 MHz, DMSO-*d*_6_): δ 8.57–8.53 (m, 1H), 8.21–8.15 (m, 1H), 7.82–7.77 (m, 1H), 7.61 (dq, *J* = 9.1, 1.0 Hz, 0H), 7.29 (ddd, *J* = 9.1, 6.7, 1.3 Hz, 1H), 6.93 (td, *J* = 6.8, 1.2 Hz, 1H). ^13^C NMR (101 MHz, DMSO): δ 145.20, 142.84, 138.12, 128.06, 127.75, 127.30, 126.21, 125.89, 125.86, 125.82, 125.78, 125.71, 117.04, 112.84, 110.73. HRMS: calcd for [M + H], 263.07906; found, 263.07907.

##### 2-(2,4-Dichlorophenyl)imidazo[1,2-*a*]pyridine (**7f**)

The title compound was prepared according to General Procedure I. Mobile phase petrol ether/EtOAc (20:70%). Yield: 634 mg (76%). ^1^H NMR (401 MHz, DMSO-*d*_6_): δ 8.64 (d, *J* = 0.7 Hz, 1H), 8.61 (dt, *J* = 6.8, 1.2 Hz, 1H), 8.29 (d, *J* = 8.5 Hz, 1H), 7.71 (d, *J* = 2.2 Hz, 1H), 7.60 (dq, *J* = 9.1, 1.0 Hz, 1H), 7.54 (dd, *J* = 8.6, 2.2 Hz, 1H), 7.30 (ddd, *J* = 9.1, 6.7, 1.3 Hz, 1H), 6.94 (td, *J* = 6.8, 1.2 Hz, 1H). ^13^C NMR (101 MHz, DMSO): δ 144.26, 139.80, 132.92, 132.22, 131.75, 131.67, 130.15, 128.08, 127.69, 126.21, 117.15, 113.42, 113.01. HRMS: calcd for [M + H], 263.01373; found, 263.01390.

##### 2-(3,4-Dichlorophenyl)imidazo[1,2-*a*]pyridine (**7g**)

The title compound was prepared according to General Procedure I. Mobile phase petrol ether/EtOAc (20:50%). Yield: 533 mg (87%). ^1^H NMR (401 MHz, DMSO-*d*_6_): δ 8.54–8.49 (m, 2H), 8.18 (d, *J* = 2.0 Hz, 1H), 7.93 (dd, *J* = 8.4, 2.0 Hz, 1H), 7.68 (d, *J* = 8.4 Hz, 1H), 7.58 (dq, *J* = 9.1, 1.0 Hz, 1H), 7.27 (ddd, *J* = 9.1, 6.7, 1.3 Hz, 1H), 6.91 (td, *J* = 6.8, 1.2 Hz, 1H). ^13^C NMR (101 MHz, DMSO): δ 145.10, 141.99, 134.88, 131.74, 131.13, 130.02, 127.25, 127.23, 125.73, 125.70, 116.94, 112.84, 110.47. HRMS: calcd for [M + H], 263.01373; found, 263.01393.

##### 4-(Imidazo[1,2-*a*]pyridin-2-yl)benzonitrile (**7h**)

The title compound was prepared according to General Procedure I. Mobile phase petrol ether/EtOAc (20:50%). Yield: 952 mg (82%). ^1^H NMR (401 MHz, DMSO-*d*_6_): δ 8.56 (s, 1H), 8.54 (dt, *J* = 6.8, 1.2 Hz, 1H), 8.18–8.12 (m, 2H), 7.91–7.85 (m, 2H), 7.60 (dq, *J* = 9.1, 1.0 Hz, 1H), 7.28 (ddd, *J* = 9.1, 6.7, 1.3 Hz, 1H), 6.92 (td, *J* = 6.7, 1.2 Hz, 1H). ^13^C NMR (101 MHz, DMSO): δ 145.40, 142.63, 138.68, 133.03, 127.44, 126.39, 126.10, 119.33, 117.14, 113.13, 111.41, 110.06. HRMS: calcd for [M + H], 220.08692; found, 220.08686.

##### 2-(4-Methoxyphenyl)imidazo[1,2-*a*]pyridine (**7i**)

The title compound was prepared according to General Procedure I. Mobile phase petrol ether/EtOAc (20:70%). Yield: 200 mg (84%). ^1^H NMR (401 MHz, DMSO-*d*_6_): δ 8.49 (dt, *J* = 6.8, 1.2 Hz, 1H), 7.92–7.87 (m, 2H), 7.54 (dq, *J* = 9.1, 1.0 Hz, 1H), 7.21 (ddd, *J* = 9.1, 6.7, 1.3 Hz, 1H), 7.03–6.98 (m, 2H), 6.86 (td, *J* = 6.7, 1.2 Hz, 1H). ^13^C NMR (101 MHz, DMSO): δ 159.48, 145.19, 144.90, 127.33, 127.16, 127.00, 125.09, 116.86, 114.59, 112.51, 108.48, 55.60. HRMS: calcd for [M + H], 240.07675; found, 240.07674.

##### 6-(4-Chlorophenyl)-5-iodoimidazo[2,1-*b*]thiazole (**8a**)

The title compound was prepared according to General Procedure II. Mobile phase petrol ether/EtOAc (10:50%). Yield: 421 mg (89%). ^1^H NMR (401 MHz, DMSO-*d*_6_): δ 7.98 (m, 2H), 7.87 (d, *J* = 4.5 Hz, 1H), 7.52 (m, 2H), 7.42 (d, *J* = 4.5 Hz, 1H). ^13^C NMR (101 MHz, DMSO): δ 149.88, 145.54, 133.64, 131.78, 129.09, 126.85, 120.52, 113.85, 110.30. HRMS: calcd for [M + H], 360.90577; found, 360.90586.

##### 6-(3,4-Dichlorophenyl)-5-iodoimidazo[2,1-*b*]thiazole (**8g**)

The title compound was prepared according to General Procedure II. Mobile phase petrol ether/EtOAc (10:50%). Yield: 514 mg (96%). ^1^H NMR (401 MHz, DMSO-*d*_6_): δ 8.17 (d, *J* = 2.1 Hz, 1H), 7.99 (dd, *J* = 8.5, 2.1 Hz, 1H), 7.90 (d, *J* = 4.5 Hz, 1H), 7.75 (d, *J* = 8.5 Hz, 1H), 7.46 (d, *J* = 4.5 Hz, 1H). ^13^C NMR (101 MHz, DMSO): δ 150.98, 145.37, 134.98, 131.61, 131.21, 130.34, 128.53, 126.97, 120.67, 114.97, 60.96. HRMS: calcd for [M + H], 394.86679; found, 394.86699.

##### 2-(4-Chlorophenyl)-3-iodoimidazo[1,2-*a*]pyridine (**9a**)

The title compound was prepared according to General Procedure II. Mobile phase petrol ether/EtOAc (10:50%). Yield: 352 mg (98%). ^1^H NMR (401 MHz, DMSO-*d*_6_): δ 8.42 (dt, *J* = 6.9, 1.2 Hz, 1H), 8.11–8.06 (m, 2H), 7.63 (dt, *J* = 9.0, 1.2 Hz, 1H), 7.60–7.55 (m, 2H), 7.38 (ddd, *J* = 9.0, 6.8, 1.3 Hz, 1H), 7.09 (td, *J* = 6.8, 1.2 Hz, 1H). ^13^C NMR (101 MHz, CDCl_3_): δ 147.52, 145.52, 132.99, 132.85, 129.73, 128.67, 127.35, 126.47, 117.16, 113.93, 63.82. HRMS: calcd for [M + H], 354.94935; found, 354.94944.

##### 3-Iodo-2-(*p*-tolyl)imidazo[1,2-*a*]pyridine (**9b**)

The title compound was prepared according to General Procedure II. Mobile phase petrol ether/EtOAc (10:50%). Yield: 501 mg (94%). ^1^H NMR (401 MHz, DMSO-*d*_6_): δ 8.41 (dt, *J* = 6.9, 1.2 Hz, 1H), 7.98–7.92 (m, 2H), 7.61 (dt, *J* = 9.0, 1.1 Hz, 1H), 7.36 (ddd, *J* = 9.0, 6.7, 1.3 Hz, 1H), 7.31 (d, *J* = 8.0 Hz, 2H), 7.06 (td, *J* = 6.8, 1.2 Hz, 1H), 2.36 (s, 3H). ^13^C NMR (101 MHz, DMSO): δ 147.70, 147.12, 137.95, 131.41, 129.41, 128.32, 127.44, 126.35, 117.29, 113.92, 63.23, 21.36. HRMS: calcd for [M + H], 335.00397; found, 335.00397.

##### 2-(4-Ethylphenyl)-3-iodoimidazo[1,2-*a*]pyridine (**9c**)

The title compound was prepared according to General Procedure II. Mobile phase petrol ether/EtOAc (10:50%). Yield: 1.23 g (86%). ^1^H NMR (401 MHz, DMSO-*d*_6_): δ 8.41 (dt, *J* = 6.9, 1.1 Hz, 1H), 8.00–7.95 (m, 2H), 7.62 (dt, *J* = 9.0, 1.1 Hz, 1H), 7.39–7.36 (m, 3H), 7.35–7.31 (m, 2H), 7.07 (td, *J* = 6.8, 1.2 Hz, 1H), 2.66 (q, *J* = 7.6 Hz, 2H), 1.22 (t, *J* = 7.6 Hz, 3H). ^13^C NMR (101 MHz, DMSO): δ 147.41, 146.83, 143.95, 131.35, 128.10, 127.93, 127.16, 126.08, 117.00, 113.65, 62.95, 28.15, 15.67. HRMS: calcd for [M + H], 349.01962; found, 349.01982.

##### 2-(4-Fluorophenyl)-3-iodoimidazo[1,2-*a*]pyridine (**9d**)

The title compound was prepared according to General Procedure II. Mobile phase petrol ether/EtOAc (10:50%). Yield: 1.42 g (97%). ^1^H NMR (401 MHz, DMSO-*d*_6_): δ 8.40 (dt, *J* = 6.9, 1.1 Hz, 1H), 8.12–8.06 (m, 2H), 7.62 (dt, *J* = 9.0, 1.1 Hz, 1H), 7.40–7.30 (m, 3H), 7.07 (td, *J* = 6.8, 1.2 Hz, 1H). ^13^C NMR (101 MHz, DMSO): δ 162.15 (d, *J* = 245.5 Hz), 147.44, 145.88, 130.42 (d, *J* = 3.0 Hz), 130.15 (d, *J* = 8.3 Hz), 127.25, 126.28, 117.05, 115.48 (d, *J* = 21.5 Hz), 113.78, 63.34. HRMS: calcd for [M + H], 338.97890; found, 338.97902.

##### 3-Iodo-2-(4-(trifluoromethyl)phenyl)imidazo[1,2-*a*]pyridine (**9e**)

The title compound was prepared according to General Procedure II. Mobile phase petrol ether/EtOAc (10:50%). Yield: 1.25 g (92%). ^1^H NMR (401 MHz, DMSO-*d*_6_): δ 8.46 (d, *J* = 6.9 Hz, 1H), 8.31 (d, *J* = 8.0 Hz, 2H), 7.88 (d, *J* = 8.1 Hz, 2H), 7.67 (d, *J* = 9.0 Hz, 1H), 7.42 (dd, *J* = 8.9, 6.9 Hz, 1H), 7.12 (t, *J* = 6.8 Hz, 1H). ^13^C NMR (101 MHz, DMSO): δ 147.90, 145.35, 138.31, 128.87, 128.65 127.74, 126.96, 125.81 (*J* = 3.78 Hz), 124.77, 117.61, 114.38, 65.13. HRMS: calcd for [M + H], 388.97570; found, 388.97582.

##### 2-(2,4-Dichlorophenyl)-3-iodoimidazo[1,2-*a*]pyridine (**9f**)

The title compound was prepared according to General Procedure II. Mobile phase petrol ether/EtOAc (10:50%). Yield: 238 mg (89%). ^1^H NMR (401 MHz, DMSO-*d*_6_): δ 8.40 (dt, *J* = 6.9, 1.2 Hz, 1H), 7.79 (d, *J* = 2.0 Hz, 1H), 7.64 (dt, *J* = 9.0, 1.1 Hz, 1H), 7.59–7.51 (m, 2H), 7.40 (ddd, *J* = 9.0, 6.8, 1.3 Hz, 1H), 7.12 (td, *J* = 6.8, 1.2 Hz, 1H). ^13^C NMR (101 MHz, DMSO): δ 147.39, 146.72, 134.57, 134.42, 134.34, 132.87, 129.62, 127.72, 127.46, 126.52, 117.62, 114.32, 67.98. HRMS: calcd for [M + H], 388.91037; found, 388.91071.

##### 2-(3,4-Dichlorophenyl)-3-iodoimidazo[1,2-*a*]pyridine (**9g**)

The title compound was prepared according to General Procedure II. Mobile phase petrol ether/EtOAc (10:50%). Yield: 638 mg (98%). ^1^H NMR (401 MHz, chloroform-*d*): δ 8.24 (dt, *J* = 6.9, 1.1 Hz, 1H), 8.22 (d, *J* = 2.1 Hz, 1H), 7.96 (dd, *J* = 8.4, 2.1 Hz, 1H), 7.66 (dt, *J* = 9.0, 1.2 Hz, 1H), 7.55 (d, *J* = 8.4 Hz, 1H), 7.33 (ddd, *J* = 9.1, 6.8, 1.3 Hz, 1H), 6.99 (td, *J* = 6.9, 1.2 Hz, 1H). ^13^C NMR (101 MHz, DMSO): δ 179.90, 147.82, 144.25, 134.93, 131.66, 131.24, 131.09, 129.70, 128.14, 127.74, 127.08, 117.55, 114.43, 64.93. HRMS: calcd for [M + H], 388.91037; found, 388.91052.

##### 4-(3-Iodoimidazo[1,2-*a*]pyridin-2-yl)benzonitrile (**9h**)

The title compound was prepared according to General Procedure II. Mobile phase petrol ether/EtOAc (10:50%). Yield: 245 mg (90%). ^1^H NMR (401 MHz, DMSO-*d*_6_): δ 8.46 (dt, *J* = 7.0, 1.1 Hz, 1H), 8.31–8.26 (m, 2H), 8.00–7.95 (m, 2H), 7.66 (dt, *J* = 9.1, 1.1 Hz, 1H), 7.41 (ddd, *J* = 9.1, 6.8, 1.2 Hz, 1H), 7.11 (td, *J* = 6.8, 1.2 Hz, 1H). ^13^C NMR (101 MHz, DMSO): δ 147.64, 144.64, 138.54, 132.60, 128.49, 127.49, 126.85, 119.02, 117.37, 114.20, 110.54, 65.39. HRMS: calcd for [M + H], 345.98357; found, 345.98370.

##### 3-Iodo-2-(4-methoxyphenyl)imidazo[1,2-*a*]pyridine (**9i**)

The title compound was prepared according to General Procedure II. Mobile phase petrol ether/EtOAc (10:50%). Yield: 238 mg (89%). ^1^H NMR (401 MHz, DMSO-*d*_6_): δ 8.39 (dt, *J* = 6.9, 1.1 Hz, 1H), 8.05–7.96 (m, 2H), 7.60 (dt, *J* = 9.0, 1.1 Hz, 1H), 7.35 (ddd, *J* = 9.0, 6.7, 1.2 Hz, 1H), 7.09–7.05 (m, 3H), 3.82 (s, 3H). ^13^C NMR (101 MHz, DMSO): δ 159.68, 147.67, 146.99, 129.70, 127.38, 126.59, 126.27, 117.16, 114.27, 113.83, 62.62, 55.66. HRMS: calcd for [M + H], 350.99888; found, 350.99893.

##### 6-(4-Chlorophenyl)-5-((trimethylsilyl)ethynyl)imidazo[2,1-*b*]thiazole (**10a**)

The title compound was prepared according to General Procedure III (Scheme 1). Mobile phase petrol ether/EtOAc (10:50%). Yield: 458 mg (61%). ^1^H NMR (500 MHz, DMSO-*d*_6_): δ 8.10 (m, 2H), 7.92 (d, *J* = 4.4 Hz, 1H), 7.51–7.54 (m, 2H), 7.44 (d, *J* = 4.4 Hz, 1H), 0.31. ^13^C NMR (101 MHz, DMSO): δ 149.86, 145.03, 133.72, 132.31, 128.81, 127.46, 119.32, 115.60, 106.93, 105.19, 93.51, −0.17. EI MS: calcd for [M + H], 330.0414; found, 330.0416.

##### 2-(4-Chlorophenyl)-3-((trimethylsilyl)ethynyl)imidazo[1,2-*a*]pyridine (**11a**)

The title compound was prepared according to General Procedure III. Mobile phase petrol ether/EtOAc (10:50%). Yield: 625 mg (56%). ^1^H NMR (401 MHz, DMSO-*d*_6_): δ 8.41 (dt, *J* = 6.8, 1.2 Hz, 1H), 8.28–8.21 (m, 2H), 7.70 (dt, *J* = 9.0, 1.1 Hz, 1H), 7.59–7.53 (m, 2H), 7.46 (ddd, *J* = 9.0, 6.8, 1.3 Hz, 1H), 7.14 (td, *J* = 6.8, 1.2 Hz, 1H), 0.34 (s, 9H). ^13^C NMR (101 MHz, DMSO): δ 146.17, 144.70, 133.43, 132.04, 128.88, 128.28, 127.82, 125.82, 117.30, 114.28, 108.95, 104.06, 93.27, −0.10. HRMS: calcd for [M + H], 325.09223; found, 325.09232.

##### 2-(*p*-Tolyl)-3-((trimethylsilyl)ethynyl)imidazo[1,2-*a*]pyridine (**11b**)

The title compound was prepared according to General Procedure III. Mobile phase petrol ether/EtOAc (10:50%). Yield: 117 mg (64%). ^1^H NMR (401 MHz, DMSO-*d*_6_): δ 8.39 (d, *J* = 6.8 Hz, 1H), 8.16 (d, *J* = 7.8 Hz, 2H), 7.69 (d, *J* = 9.0 Hz, 1H), 7.43 (dd, *J* = 8.9, 6.9 Hz, 1H), 7.30 (d, *J* = 7.9 Hz, 2H), 7.12 (t, *J* = 6.8 Hz, 1H), 2.36 (s, 3H), 0.34 (s, 9H). ^13^C NMR (101 MHz, DMSO): δ 147.99, 144.94, 138.80, 130.71, 129.64, 127.79, 126.95, 125.98, 117.43, 114.32, 108.68, 103.79, 94.13, 21.38, 0.24. HRMS: calcd for [M + H], 305.14685; found, 305.14690.

##### 2-(4-Ethylphenyl)-3-((trimethylsilyl)ethynyl)imidazo[1,2-*a*]pyridine (**11c**)

The title compound was prepared according to General Procedure III. Mobile phase petrol ether/EtOAc (10:50%). Yield: 126 mg (63%). ^1^H NMR (401 MHz, DMSO-*d*_6_): δ 8.39 (dt, *J* = 6.8, 1.2 Hz, 1H), 8.22–8.16 (m, 2H), 7.69 (dt, *J* = 9.0, 1.1 Hz, 1H), 7.42 (ddd, *J* = 9.0, 6.8, 1.3 Hz, 1H), 7.35–7.29 (m, 2H), 7.11 (td, *J* = 6.8, 1.2 Hz, 1H), 2.65 (q, *J* = 7.6 Hz, 2H), 1.20 (t, *J* = 7.6 Hz, 3H), 0.34 (s, 9H). ^13^C NMR (101 MHz, DMSO): δ 147.66, 144.72, 144.65, 130.69, 128.12, 127.44, 126.73, 125.64, 117.14, 113.98, 108.33, 103.51, 93.84, 28.18, 15.57, −0.06. HRMS: calcd for [M + H], 319.16250; found, 319.16255.

##### 2-(4-Fluorophenyl)-3-((trimethylsilyl)ethynyl)imidazo[1,2-*a*]pyridine (**11d**)

The title compound was prepared according to General Procedure III. Mobile phase petrol ether/EtOAc (10:50%). Yield: 250 mg (69%). ^1^H NMR (401 MHz, DMSO-*d*_6_): δ 8.41 (dd, *J* = 6.9, 1.4 Hz, 1H), 8.32–8.25 (m, 2H), 7.70 (dd, *J* = 9.1, 1.4 Hz, 1H), 7.45 (ddt, *J* = 9.0, 6.8, 1.3 Hz, 1H), 7.38–7.31 (m, 2H), 7.13 (tt, *J* = 6.9, 1.3 Hz, 1H), 0.36–0.31 (m, 9H). ^13^C NMR (101 MHz, DMSO): δ 162.46 (d, *J* = 246.2 Hz), 146.53, 144.70, 129.74 (d, *J* = 3.0 Hz), 128.79 (d, *J* = 8.4 Hz), 127.74, 125.81, 117.24, 117.24, 115.81 (d, *J* = 21.6 Hz), 114.21, 114.21, 108.58, 103.68, 93.46, −0.05. HRMS: calcd for [M + H], 309.12178; found, 309.12195.

##### 2-(4-(Trifluoromethyl)phenyl)-3-((trimethylsilyl)ethynyl)imidazo[1,2-*a*]pyridine (**11e**)

The title compound was prepared according to General Procedure III. Mobile phase petrol ether/EtOAc (10:50%). Yield: 123 mg (54%). ^1^H NMR (401 MHz, DMSO-*d*_6_): δ 8.45 (tt, *J* = 6.8, 1.0 Hz, 3H), 7.91–7.82 (m, 2H), 7.74 (dt, *J* = 9.1, 1.1 Hz, 1H), 7.49 (ddd, *J* = 9.1, 6.8, 1.3 Hz, 1H), 7.17 (td, *J* = 6.8, 1.2 Hz, 1H), 0.37 (s, 9H). ^13^C NMR (101 MHz, DMSO): δ 145.80, 145.12, 137.36, 137.35, 129.25, 128.93, 128.38, 127.42, 126.23, 126.08 (q, *J* = 3.9 Hz), 117.80, 114.82, 109.61, 105.20, 93.24, 0.18. HRMS: calcd for [M + H], 359.11859; found, 359.11862.

##### 2-(2,4-Dichlorophenyl)-3-((trimethylsilyl)ethynyl)imidazo[1,2-*a*]pyridine (**11f**)

The title compound was prepared according to General Procedure III. Mobile phase petrol ether/EtOAc (10:50%). Yield: 98 mg (64%). ^1^H NMR (401 MHz, chloroform-*d*): δ 8.30 (dt, *J* = 6.8, 1.2 Hz, 1H), 7.64 (m, 2H), 7.54 (d, *J* = 2.1 Hz, 1H), 7.38–7.27 (m, 2H), 6.97 (td, *J* = 6.8, 1.2 Hz, 1H), 0.27 (s, 9H). ^13^C NMR (101 MHz, CDCl_3_): δ 146.79, 144.80, 135.05, 134.32, 133.22, 131.39, 130.12, 126.95, 126.52, 125.55, 117.98, 113.42, 108.14, 92.17, 0.04. HRMS: calcd for [M + H], 321.14177; found, 321.14179.

##### 2-(3,4-Dichlorophenyl)-3-((trimethylsilyl)ethynyl)imidazo[1,2-*a*]pyridine (**11g**)

The title compound was prepared according to General Procedure III and used as crude in the next step without purification.

##### 4-(3-((Trimethylsilyl)ethynyl)imidazo[1,2-*a*]pyridin-2-yl)benzonitrile (**11h**)

The title compound was prepared according to General Procedure III. Mobile phase petrol ether/EtOAc (10:50%). Yield: 170 mg (65%). ^1^H NMR (401 MHz, DMSO-*d*_6_): δ 8.44 (dt, *J* = 6.8, 1.2 Hz, 1H), 8.42–8.38 (m, 2H), 7.99–7.94 (m, 2H), 7.74 (dt, *J* = 9.0, 1.1 Hz, 1H), 7.49 (ddd, *J* = 9.1, 6.8, 1.3 Hz, 1H), 7.17 (td, *J* = 6.8, 1.1 Hz, 1H). ^13^C NMR (101 MHz, DMSO): δ 145.16, 144.88, 137.51, 132.85, 128.24, 127.06, 125.97, 118.91, 117.56, 114.62, 110.97, 109.67, 105.24, 92.83, −0.14. HRMS: calcd for [M + H], 316.12645; found, 316.12656.

##### 2-(4-Methoxyphenyl)-3-((trimethylsilyl)ethynyl)imidazo[1,2-*a*]pyridine (**11i**)

The title compound was prepared according to General Procedure III. Mobile phase petrol ether/EtOAc (10:50%). Yield: 98 mg (64%). ^1^H NMR (401 MHz, DMSO-*d*_6_): δ 8.38 (dt, *J* = 6.8, 1.2 Hz, 1H), 8.26–8.18 (m, 2H), 7.67 (dt, *J* = 9.0, 1.1 Hz, 1H), 7.42 (ddd, *J* = 9.0, 6.8, 1.3 Hz, 1H), 7.11 (td, *J* = 6.8, 1.2 Hz, 1H), 7.08–7.03 (m, 2H), 3.82 (s, 3H), 0.34 (s, 9H). ^13^C NMR (101 MHz, DMSO): δ 159.83, 147.63, 144.60, 128.13, 127.19, 125.75, 125.49, 116.93, 114.07, 113.72, 108.09, 102.92, 94.03, 55.32, −0.04, −0.06, −0.08. HRMS: calcd for [M + H], 321.14177; found, 321.14179.

##### 5-(1-(3,4-Dichlorobenzyl)-1*H*-1,2,3-triazol-4-yl)-6-(3,4-dichlorophenyl)imidazo[2,1-*b*]thiazole (**12g**)

The title compound was prepared according to General Procedure IV. Mobile phase petrol ether/EtOAc (30:60%). Yield: 51 mg (92%). ^1^H NMR (401 MHz, DMSO-*d*_6_): δ 8.57 (s, 1H), 8.01 (d, *J* = 4.5 Hz, 1H), 7.87 (d, *J* = 2.0 Hz, 1H), 7.71 (d, *J* = 2.1 Hz, 1H), 7.69–7.64 (m, 2H), 7.61 (d, *J* = 8.4 Hz, 1H), 7.40 (d, *J* = 4.5 Hz, 1H), 7.36 (dd, *J* = 8.3, 2.1 Hz, 1H), 5.70 (s, 2H). ^13^C NMR (101 MHz, DMSO): δ 149.77, 141.34, 136.86, 136.81, 135.00, 131.52, 131.39, 131.24, 131.21, 130.87, 130.44, 130.14, 128.90, 128.65, 127.44, 123.91, 119.85, 114.89, 114.40, 51.92. HRMS: calcd for [M + H], 493.95620; found, 493.95625.

##### 3-(1-(3,4-Dichlorobenzyl)-1*H*-1,2,3-triazol-4-yl)-2-(*p*-tolyl)imidazo[1,2-*a*]pyridine (**13b**)

The title compound was prepared according to General Procedure IV. Mobile phase petrol ether/EtOAc (20:70%). Yield: 140 mg (84%).^1^H NMR (401 MHz, DMSO-*d*_6_): δ 8.47 (dd, *J* = 7.1, 1.5 Hz, 1H), 8.45 (d, *J* = 1.5 Hz, 0H), 7.72–7.65 (m, 1H), 7.56–7.51 (m, 1H), 7.39–7.33 (m, 1H), 7.16 (d, *J* = 7.8 Hz, 1H), 6.97 (td, *J* = 6.9, 1.7 Hz, 0H), 5.74 (s, 1H), 2.32 (s, 2H). ^13^C NMR (101 MHz, DMSO): δ 144.82, 143.87, 137.48, 137.11, 136.79, 131.51, 131.27, 131.23, 131.16, 130.25, 129.17, 128.54, 125.88, 125.74, 125.30, 116.96, 113.06, 110.91, 51.86, 21.01. HRMS: calcd for [M + H], 434.09338; found, 434.09355.

##### 3-(1-(3,4-Dichlorobenzyl)-1*H*-1,2,3-triazol-4-yl)-2-(4-ethylphenyl)imidazo[1,2-*a*]pyridine (**13c**)

The title compound was prepared according to General Procedure IV. Mobile phase petrol ether/EtOAc (20:70%). Yield: 350 mg (70%). ^1^H NMR (401 MHz, DMSO-*d*_6_): δ 8.48–8.43 (m, 2H), 7.72–7.65 (m, 3H), 7.59–7.54 (m, 2H), 7.38–7.33 (m, 2H), 7.20–7.15 (m, 2H), 6.97 (td, *J* = 6.9, 1.2 Hz, 1H), 5.74 (s, 2H), 2.61 (q, *J* = 7.6 Hz, 2H), 1.19 (t, *J* = 7.6 Hz, 3H). ^13^C NMR (101 MHz, DMSO): δ 144.85, 143.88, 143.75, 137.12, 136.79, 131.52, 131.24, 131.19, 130.26, 128.56, 127.95, 127.90, 125.90, 125.86, 125.28, 116.98, 113.09, 110.92, 51.91, 28.10, 15.55. HRMS: calcd for [M + H], 448.10903; found, 448.10914.

##### 3-(1-(3,4-Dichlorobenzyl)-1*H*-1,2,3-triazol-4-yl)-2-(4-fluorophenyl)imidazo[1,2-*a*]pyridine (**13d**)

The title compound was prepared according to General Procedure IV. Mobile phase petrol ether/EtOAc (30:80%). Yield: 211 mg (74%). ^1^H NMR (401 MHz, DMSO-*d*_6_): δ 8.50 (s, 1H), 8.48 (dt, *J* = 7.0, 1.2 Hz, 1H), 7.75–7.65 (m, 6H), 7.41–7.32 (m, 2H), 7.24–7.15 (m, 2H), 6.99 (td, *J* = 6.8, 1.2 Hz, 1H), 5.74 (s, 2H). ^13^C NMR (101 MHz, DMSO): δ 162.40 (d, *J* = 245.1 Hz), 145.14, 143.14, 137.30, 136.80, 131.80, 131.53, 131.47, 130.91 (d, *J* = 3.1 Hz), 130.62, 130.31 (d, *J* = 8.3 Hz), 128.81, 126.41, 126.10, 125.68, 117.33, 115.82 (d, *J* = 21.5 Hz), 113.53, 111.49, 52.21. HRMS: calcd for [M + H], 438.06831; found, 438.06851.

##### 3-(1-(3,4-Dichlorobenzyl)-1*H*-1,2,3-triazol-4-yl)-2-(4-(trifluoromethyl)phenyl)imidazo[1,2-*a*]pyridine (**13e**)

The title compound was prepared according to General Procedure IV. Mobile phase petrol ether/EtOAc (30:80%). Yield: 143 mg (86%). ^1^H NMR (401 MHz, DMSO-*d*_6_): δ 8.52 (dt, *J* = 7.0, 1.2 Hz, 1H), 8.38 (s, 1H), 8.04–7.96 (m, 2H), 7.57 (d, *J* = 9.1 Hz, 1H), 7.31–7.21 (m, 3H), 6.89 (td, *J* = 6.8, 1.3 Hz, 1H). ^13^C NMR (101 MHz, DMSO): δ 162.01 (d, *J* = 244.2 Hz), 145.01, 143.63, 130.65, 127.68 (d, *J* = 8.2 Hz), 127.09, 125.23, 116.78, 115.77 (d, *J* = 21.5 Hz), 112.51, 109.12. HRMS: calcd for [M + H], 488.06511; found, 488.06511.

##### 3-(1-(3,4-Dichlorobenzyl)-1*H*-1,2,3-triazol-4-yl)-2-(2,4-dichlorophenyl)imidazo[1,2-*a*]pyridine (**13f**)

The title compound was prepared according to General Procedure IV. Mobile phase petrol ether/EtOAc (40:80%). Yield: 120 mg (74%). ^1^H NMR (401 MHz, DMSO-*d*_6_): δ 9.11 (dt, *J* = 7.0, 1.1 Hz, 1H), 8.02 (s, 1H), 7.71 (dt, *J* = 9.1, 1.2 Hz, 1H), 7.68 (d, *J* = 2.1 Hz, 1H), 7.64 (d, *J* = 8.3 Hz, 1H), 7.58 (d, *J* = 8.3 Hz, 1H), 7.54 (d, *J* = 2.1 Hz, 1H), 7.52 (dd, *J* = 8.3, 2.1 Hz, 1H), 7.42 (ddd, *J* = 9.1, 6.7, 1.3 Hz, 1H), 7.25 (dd, *J* = 8.3, 2.1 Hz, 1H), 7.11 (td, *J* = 6.8, 1.2 Hz, 1H), 5.67 (s, 2H). ^13^C NMR (101 MHz, DMSO): δ 144.69, 140.47, 137.27, 137.05, 134.19, 133.95, 133.64, 132.58, 131.46, 131.09, 130.00, 129.45, 128.30, 127.75, 126.19, 126.02, 123.26, 117.31, 113.72, 113.61, 51.61. HRMS: calcd for [M + H], 417.06837; found, 417.06839.

##### 3-(1-(3,4-Dichlorobenzyl)-1*H*-1,2,3-triazol-4-yl)-2-(3,4-dichlorophenyl)imidazo[1,2-*a*]pyridine (**13g**)

The title compound was prepared according to General Procedure IV. Mobile phase petrol ether/EtOAc (30:60%). Yield: 56 mg (90%). ^1^H NMR (401 MHz, DMSO-*d*_6_): δ 8.62 (s, 1H), 8.46 (dt, *J* = 7.0, 1.2 Hz, 1H), 7.85 (d, *J* = 1.9 Hz, 1H), 7.74–7.69 (m, 3H), 7.69–7.66 (m, 1H), 7.65 (d, *J* = 2.0 Hz, 1H), 7.62 (d, *J* = 8.4 Hz, 1H), 7.41 (ddd, *J* = 9.1, 6.7, 1.3 Hz, 1H), 7.37 (dd, *J* = 8.3, 2.1 Hz, 1H), 7.02 (td, *J* = 6.8, 1.2 Hz, 1H), 5.76 (s, 2H). ^13^C NMR (101 MHz, DMSO): δ 144.96, 141.00, 136.95, 136.02, 134.78, 131.55, 131.40, 131.26, 130.91, 130.65, 130.39, 129.30, 128.58, 127.85, 126.58, 126.09, 125.46, 117.20, 113.57, 112.06, 51.97. HRMS: calcd for [M + H], 487.99978; found, 487.99980.

##### 4-(3-(1-(3,4-Dichlorobenzyl)-1*H*-1,2,3-triazol-4-yl)imidazo[1,2-*a*]pyridin-2-yl)benzonitrile (**13h**)

The title compound was prepared according to General Procedure IV. Mobile phase petrol ether/EtOAc (30:80%). Yield: 189 mg (89%). ^1^H NMR (401 MHz, DMSO-*d*_6_): δ 8.59 (s, 1H), 8.44 (dt, *J* = 7.0, 1.1 Hz, 1H), 7.89–7.81 (m, 4H), 7.75–7.69 (m, 3H), 7.42 (ddd, *J* = 9.1, 6.7, 1.3 Hz, 1H), 7.36 (dd, *J* = 8.3, 2.1 Hz, 1H), 7.03 (td, *J* = 6.8, 1.2 Hz, 1H), 5.75 (s, 2H). ^13^C NMR (101 MHz, DMSO): δ 178.80, 132.59, 131.51, 131.28, 131.21, 130.39, 128.58, 128.51, 126.70, 126.27, 125.49, 117.31, 113.66, 52.00. HRMS: calcd for [M + H], 445.07298; found, 445.07299.

##### 3-(1-(3,4-Dichlorobenzyl)-1*H*-1,2,3-triazol-4-yl)-2-(4-methoxyphenyl)imidazo[1,2-*a*]pyridine (**13i**)

The title compound was prepared according to General Procedure IV. Mobile phase petrol ether/EtOAc (30:80%). Yield: 385 mg (92%). ^1^H NMR (401 MHz, DMSO-*d*_6_): δ 8.46 (d, *J* = 6.5 Hz, 1H), 7.72–7.64 (m, 1H), 7.63–7.57 (m, 1H), 7.38–7.32 (m, 1H), 6.96 (td, *J* = 6.8, 1.2 Hz, 0H), 6.95–6.90 (m, 1H), 5.74 (s, 1H), 3.78 (s, 2H). ^13^C NMR (101 MHz, DMSO): δ 159.28, 144.78, 143.76, 137.11, 136.88, 131.49, 131.23, 131.14, 130.23, 129.21, 128.52, 126.50, 125.77, 125.70, 125.23, 116.84, 114.00, 112.96, 110.44, 55.28, 51.88. HRMS: calcd for [M + H], 450.08829; found, 450.08826.

##### 5-(1-(4-Chlorobenzyl)-1*H*-1,2,3-triazol-4-yl)-6-(4-chlorophenyl)imidazo[2,1-*b*]thiazole (**14a**)

The title compound was prepared according to General Procedure IV. Mobile phase petrol ether/EtOAc (20:60%). Yield: 136 mg (88%). ^1^H NMR (401 MHz, DMSO-*d*_6_): δ 8.45 (s, 1H), 8.03 (d, *J* = 4.5 Hz, 1H), 7.75–7.64 (m, 2H), 7.49–7.41 (m, 4H), 7.40–7.36 (m, 3H), 5.68 (s, 2H). ^13^C NMR (101 MHz, DMSO): δ 149.57, 142.75, 137.05, 135.01, 133.28, 133.07, 132.35, 130.04, 129.25, 128.95, 128.69, 123.45, 119.96, 114.46, 113.94, 52.40. HRMS: calcd for [M + H], 426.03415; found, 426.03423.

##### 6-(4-Chlorophenyl)-5-(1-(3,4-dimethoxybenzyl)-1*H*-1,2,3-triazol-4-yl)imidazo[2,1-*b*]thiazole (**14b**)

The title compound was prepared according to General Procedure IV. Mobile phase petrol ether/EtOAc (20:50%). Yield: 120 mg (86%). ^1^H NMR (401 MHz, DMSO-*d*_6_): δ 8.40 (s, 1H), 8.01 (d, *J* = 4.5 Hz, 1H), 7.71–7.64 (m, 2H), 7.45–7.40 (m, 2H), 7.38 (d, *J* = 4.5 Hz, 1H), 7.05 (d, *J* = 2.0 Hz, 1H), 6.95 (d, *J* = 8.2 Hz, 1H), 6.90 (dd, *J* = 2.0, 8.2 Hz, 1H), 5.56 (s, 2H), 3.74 (s, 6H). ^13^C NMR (101 MHz, DMSO): δ 149.80, 149.24, 142.95, 137.18, 133.60, 132.60, 129.53, 128.95, 128.45, 123.44, 121.13, 120.20, 114.75, 114.35, 112.48, 112.30, 56.00, 55.97, 53.45. HRMS: calcd for [M + H], 452.09425; found, 452.09438.

##### 6-(4-Chlorophenyl)-5-(1-(4-methoxybenzyl)-1*H*-1,2,3-triazol-4-yl)imidazo[2,1-*b*]thiazole (**14c**)

The title compound was prepared according to General Procedure IV. Mobile phase petrol ether/EtOAc (20:50%). Yield: 121 mg (86%). ^1^H NMR (401 MHz, DMSO-*d*_6_): δ 8.40 (s, 1H), 8.02 (d, *J* = 4.5 Hz, 1H), 7.76–7.61 (m, 3H), 7.45–7.41 (m, 3H), 7.38 (d, *J* = 4.5 Hz, 1H), 7.33 (d, *J* = 8.7 Hz, 2H), 7.01–6.88 (m, 3H), 5.59 (s, 2H), 3.75 (s, 3H). ^13^C NMR (101 MHz, DMSO): δ 159.63, 149.81, 142.96, 137.20, 133.60, 132.60, 130.06, 129.52, 128.97, 128.22, 123.42, 120.23, 114.60, 114.34, 55.62, 53.06. HRMS: calcd for [M + H], 422.08369; found, 422.08372.

##### 5-(1-Benzyl-1*H*-1,2,3-triazol-4-yl)-6-(4-chlorophenyl)imidazo[2,1-*b*]thiazole (**14d**)

The title compound was prepared according to General Procedure IV. Mobile phase petrol ether/EtOAc (20:50%). Yield: 154 mg (87%). ^1^H NMR (401 MHz, DMSO-*d*_6_): δ 8.45 (s, 1H), 8.03 (d, *J* = 4.5 Hz, 1H), 7.75–7.65 (m, 2H), 7.45–7.41 (m, 1H), 7.40–7.32 (m, 8H), 5.68 (s, 2H). ^13^C NMR (101 MHz, DMSO): δ 149.84, 143.02, 137.27, 136.33, 133.59, 132.62, 129.54, 129.25, 128.97, 128.64, 128.34, 123.75, 120.24, 114.75, 114.29, 53.49. HRMS: calcd for [M + Na], 392.07312; found, 392.07318.

##### 6-(4-Chlorophenyl)-5-(1-(pyridin-2-ylmethyl)-1*H*-1,2,3-triazol-4-yl)imidazo[2,1-*b*]thiazole (**14e**)

The title compound was prepared according to General Procedure IV. Mobile phase petrol ether/EtOAc (20:50%). Yield: 143 mg (80%). ^1^H NMR (401 MHz, DMSO-*d*_6_): δ 8.57 (ddd, *J* = 4.8, 1.9, 1.0 Hz, 1H), 8.46 (s, 1H), 8.05 (d, *J* = 4.5 Hz, 1H), 7.85 (td, *J* = 7.7, 1.8 Hz, 1H), 7.77–7.69 (m, 2H), 7.47–7.42 (m, 2H), 7.41–7.35 (m, 2H), 7.33 (dd, *J* = 7.8, 1.1 Hz, 1H), 5.81 (s, 2H). ^13^C NMR (101 MHz, DMSO): δ 154.95, 149.61, 149.53, 142.69, 137.55, 136.85, 133.31, 132.31, 129.26, 128.67, 124.16, 123.44, 122.24, 119.94, 114.47, 114.02, 54.64. HRMS: calcd for [M + H], 393.06837; found, 393.06845.

##### 4-((4-(6-(4-Chlorophenyl)imidazo[2,1-*b*]thiazol-5-yl)-1*H*-1,2,3-triazol-1-yl)methyl)-benzonitrile (**14f**)

The title compound was prepared according to General Procedure IV. Mobile phase petrol ether/EtOAc (20:70%). Yield: 87 mg (87%). ^1^H NMR (401 MHz, DMSO-*d*_6_): δ 8.49 (s, 1H), 8.05 (d, *J* = 4.5 Hz, 1H), 7.92–7.79 (m, 2H), 7.73–7.66 (m, 2H), 7.52–7.47 (m, 2H), 7.47–7.42 (m, 2H), 7.39 (d, *J* = 4.5 Hz, 1H), 5.80 (s, 2H). ^13^C NMR (101 MHz, DMSO): δ 149.61, 142.82, 141.48, 137.13, 133.27, 132.93, 132.37, 129.28, 128.83, 128.72, 123.76, 120.00, 118.72, 114.49, 113.87, 111.11, 52.56. HRMS: calcd for [M + H], 487.99978; found, 487.99993.

##### 3-(1-(4-Chlorobenzyl)-1*H*-1,2,3-triazol-4-yl)-2-(4-chlorophenyl)imidazo[1,2-*a*]pyridine (**15a**)

The title compound was prepared according to General Procedure IV. Mobile phase petrol ether/EtOAc (20:50%). Yield: 145 mg (92%). ^1^H NMR (401 MHz, DMSO-*d*_6_): δ 8.49 (s, 1H), 8.45 (dt, *J* = 7.0, 1.2 Hz, 1H), 7.71–7.66 (m, 3H), 7.51–7.47 (m, 2H), 7.45–7.36 (m, 5H), 7.00 (td, *J* = 6.8, 1.2 Hz, 1H), 5.73 (s, 2H). ^13^C NMR (101 MHz, DMSO): δ 149.57, 142.75, 137.05, 135.01, 133.28, 133.07, 132.35, 130.04, 129.25, 128.95, 128.69, 123.45, 119.96, 114.46, 113.94, 52.40. HRMS: calcd for [M + H], 420.07773; found, 420.07765.

##### 2-(4-Chlorophenyl)-3-(1-(3,4-dimethoxybenzyl)-1*H*-1,2,3-triazol-4-yl)imidazo[1,2-*a*]pyridine (**15b**)

The title compound was prepared according to General Procedure IV. Mobile phase petrol ether/EtOAc (30:60%). Yield: 111 mg (81%). ^1^H NMR (401 MHz, DMSO-*d*_6_): δ 8.47–8.42 (m, 2H), 7.71–7.66 (m, 3H), 7.43–7.36 (m, 3H), 7.05 (d, *J* = 1.9 Hz, 1H), 7.03–6.95 (m, 2H), 6.91 (dd, *J* = 2.0, 8.2 Hz, 1H), 5.62 (s, 2H), 3.75 (d, *J* = 3.8 Hz, 6H). ^13^C NMR (101 MHz, DMSO): δ 149.27, 149.24, 145.12, 142.55, 136.41, 133.26, 133.11, 129.92, 128.94, 128.51, 126.60, 125.78, 125.63, 121.03, 117.34, 113.67, 112.40, 112.33, 112.05, 56.00, 55.97, 53.53. HRMS: calcd for [M + Na], 446.13783; found, 446.13786.

##### 2-(4-Chlorophenyl)-3-(1-(4-methoxybenzyl)-1*H*-1,2,3-triazol-4-yl)imidazo[1,2-*a*]pyridine (**15c**)

The title compound was prepared according to General Procedure IV. Mobile phase petrol ether/EtOAc (20:50%). Yield: 96 mg (75%). ^1^H NMR (401 MHz, DMSO-*d*_6_): δ 8.50–8.35 (m, 2H), 7.71–7.66 (m, 3H), 7.44–7.31 (m, 5H), 7.02–6.93 (m, 1H), 5.63 (s, 2H), 3.76 (s, 3H). ^13^C NMR (101 MHz, DMSO): δ 159.64, 145.18, 142.68, 136.46, 133.33, 133.07, 129.96, 129.90, 128.95, 128.27, 126.51, 125.76, 125.62, 117.40, 114.64, 113.63, 112.01, 55.63, 53.16. HRMS: calcd for [M + H], 416.12726; found, 416.12730.

##### 3-(1-Benzyl-1*H*-1,2,3-triazol-4-yl)-2-(4-chlorophenyl)imidazo[1,2-*a*]pyridine (**15d**)

The title compound was prepared according to General Procedure IV. Mobile phase petrol ether/EtOAc (20:50%). Yield: 139 mg (78%). ^1^H NMR (401 MHz, DMSO-*d*_6_): δ 8.50 (s, 1H), 8.45 (dt, *J* = 1.1, 6.9 Hz, 1H), 7.69 (m, 3H), 7.44–7.37 (m, 8H), 7.00 (td, *J* = 1.2, 6.8 Hz, 2H), 5.73 (s, 2H). ^13^C NMR (101 MHz, DMSO): δ 145.20, 142.73, 136.53, 136.40, 133.32, 133.09, 129.92, 129.29, 128.95, 128.65, 128.24, 126.54, 126.12, 125.63, 117.40, 113.65, 111.95, 53.57. HRMS: calcd for [M + H], 386.11670; found, 386.11681.

##### 2-(4-Chlorophenyl)-3-(1-(pyridin-2-ylmethyl)-1*H*-1,2,3-triazol-4-yl)imidazo[1,2-*a*]pyridine (**15e**)

The title compound was prepared according to General Procedure IV. Mobile phase petrol ether/EtOAc (20:50%). Yield: 147 mg (83%). ^1^H NMR (401 MHz, DMSO-*d*_6_): δ 8.58 (dt, *J* = 4.7, 1.4 Hz, 1H), 8.52 (s, 1H), 8.47 (dt, *J* = 6.9, 1.2 Hz, 1H), 7.85 (td, *J* = 7.7, 1.8 Hz, 1H), 7.77–7.71 (m, 2H), 7.68 (dt, *J* = 9.1, 1.2 Hz, 1H), 7.44–7.39 (m, 2H), 7.39–7.33 (m, 3H), 6.99 (td, *J* = 6.8, 1.2 Hz, 1H), 5.87 (s, 2H). ^13^C NMR (101 MHz, DMSO): δ 155.00, 149.65, 144.91, 142.41, 137.55, 136.14, 133.05, 132.80, 129.64, 128.63, 126.55, 126.16, 125.31, 123.45, 122.21, 117.10, 113.29, 111.70, 54.75. HRMS: calcd for [M + H], 387.11195; found, 387.11198.

##### 4-((4-(2-(4-Chlorophenyl)imidazo[1,2-*a*]pyridin-3-yl)-1*H*-1,2,3-triazol-1-yl)methyl)benzonitrile (**15f**)

The title compound was prepared according to General Procedure IV. Mobile phase petrol ether/EtOAc (40:80%). Yield: 148 mg (82%). ^1^H NMR (401 MHz, DMSO-*d*_6_): δ 8.53 (s, 1H), 8.48 (dt, *J* = 6.9, 1.2 Hz, 1H), 7.94–7.88 (m, 2H), 7.72–7.67 (m, 3H), 7.53–7.48 (m, 2H), 7.47–7.42 (m, 2H), 7.39 (ddd, *J* = 9.1, 6.7, 1.3 Hz, 1H), 7.01 (td, *J* = 6.8, 1.2 Hz, 1H), 5.85 (s, 2H). ^13^C NMR (101 MHz, DMSO): δ 145.23, 142.82, 141.84, 136.69, 133.31, 133.27, 133.14, 129.94, 129.06, 129.00, 126.57, 126.42, 125.70, 119.02, 117.41, 113.65, 111.82, 111.44, 52.96. HRMS: calcd for [M + H], 411.11195; found, 411.11215.

##### 2-(4-Chlorophenyl)-3-(1-(4-(methylthio)benzyl)-1*H*-1,2,3-triazol-4-yl)imidazo[1,2-*a*]pyridine (**15g**)

The title compound was prepared according to General Procedure IV. Mobile phase petrol ether/EtOAc (40:100%). Yield: 72 mg (80%). ^1^H NMR (401 MHz, DMSO-*d*_6_): δ 8.46 (s, 1H), 8.44 (dt, *J* = 6.9, 1.2 Hz, 1H), 7.72–7.65 (m, 3H), 7.44–7.35 (m, 3H), 7.33–7.27 (m, 3H), 6.99 (td, *J* = 6.8, 1.2 Hz, 1H), 5.67 (s, 2H), 2.47 (s, 3H). ^13^C NMR (101 MHz, DMSO): δ 144.91, 142.43, 138.60, 136.24, 133.02, 132.81, 132.52, 129.62, 128.76, 128.67, 126.28, 126.24, 125.67, 125.34, 117.10, 113.35, 111.67, 52.88, 14.76. HRMS: calcd for [M + H], 431.09715; found, 431.09723.

##### 2-(4-Chlorophenyl)-3-(1-(1-phenylethyl)-1*H*-1,2,3-triazol-4-yl)imidazo[1,2-*a*]pyridine (**15h**)

The title compound was prepared according to General Procedure IV. Mobile phase petrol ether/EtOAc (40:100%). Yield: 72 mg (80%). ^1^H NMR (401 MHz, DMSO-*d*_6_): δ 8.58 (s, 1H), 8.41 (dt, *J* = 6.9, 1.2 Hz, 1H), 7.69 (dd, *J* = 8.4, 1.7 Hz, 3H), 7.44–7.32 (m, 9H), 6.99 (td, *J* = 6.8, 1.2 Hz, 1H), 6.08 (q, *J* = 7.1 Hz, 1H), 1.96 (d, *J* = 7.1 Hz, 3H). ^13^C NMR (101 MHz, DMSO): δ 144.88, 142.41, 141.14, 136.02, 133.00, 132.75, 129.52, 128.95, 128.58, 128.24, 126.43, 126.20, 125.28, 124.60, 117.08, 113.30, 111.70, 59.89, 21.25. HRMS: calcd for [M + H], 400.13235; found, 400.13214.

##### 2-(4-Chlorophenyl)-3-(1-(4-(methylsulfonyl)benzyl)-1*H*-1,2,3-triazol-4-yl)imidazo[1,2-*a*]pyridine (**15i**)

The title compound was prepared according to General Procedure IV. Mobile phase petrol ether/EtOAc (40:100%). Yield: 613 mg (92%). ^1^H NMR (401 MHz, DMSO-*d*_6_): δ 8.52 (dt, *J* = 6.8, 1.1 Hz, 1H), 8.07 (s, 1H), 7.98 (m, 2H), 7.72 (dt, *J* = 9.1, 1.1 Hz, 1H), 7.69 (m, 2H), 7.60 (m, 2H), 7.45 (m, 2H), 7.42 (ddd, *J* = 9.1, 6.8, 1.1 Hz, 1H), 7.04 (td, *J* = 6.8, 1.1 Hz, 1H), 5.97 (s, 2H), 3.23 (s, 3H). ^13^C NMR (101 MHz, DMSO-*d*_6_): δ 145.13, 143.06, 141.54, 140.72, 137.61, 135.29, 133.03, 132.90, 129.83, 128.91, 128.72, 127.68, 126.50, 125.39, 117.20, 113.61, 111.13, 57.56, 43.65. HRMS: calcd for [M - H], 462.07970; found, 462.07935.

##### 2-(4-Chlorophenyl)-3-(1-(4-(pyrrolidin-1-ylsulfonyl)benzyl)-1*H*-1,2,3-triazol-4-yl)imidazo[1,2-*a*]pyridine (**15j**)

The title compound was prepared according to General Procedure IV. Mobile phase petrol ether/EtOAc (40:100%). Yield: 144 mg (93%). ^1^H NMR (401 MHz, DMSO-*d*_6_): δ 8.49 (d, *J* = 6.6 Hz, 1H), 8.03 (s, 1H), 7.85 (m, 2H), 7.67–7.73 (m, 3H), 7.57 (m, 2H), 7.43 (m, 2H), 7.40 (m, 1H), 7.02 (t, *J* = 6.6 Hz, 1H), 5.94 (s, 2H), 3.17 (m, 4H), 1.66 (m, 4H). ^13^C NMR (101 MHz, DMSO-*d*_6_): δ 144.97, 142.99, 140.4, 137.50, 136.50, 134.97, 132–132.89 (m), 129.62, 128.55, 127.63, 126.13, 125.07, 116.98, 113.26, 110.97, 57.43, 47.71, 24.65. HRMS: calcd for [M – H], 517.12190; found, 517.12134.

##### 2-(4-Chlorophenyl)-3-(1-(4-nitrobenzyl)-1*H*-1,2,3-triazol-4-yl)imidazo[1,2-*a*]pyridine (**15k**)

The title compound was prepared according to General Procedure IV. Mobile phase petrol ether/EtOAc (40:100%). Yield: 253 mg (88%). ^1^H NMR (401 MHz, DMSO-*d*_6_): δ 8.56 (s, 1H), 8.49 (dt, *J* = 6.9, 1.2 Hz, 1H), 8.31–8.21 (m, 2H), 7.73–7.67 (m, 3H), 7.62–7.54 (m, 2H), 7.47–7.41 (m, 2H), 7.39 (ddd, *J* = 9.0, 6.7, 1.3 Hz, 1H), 7.00 (td, *J* = 6.8, 1.3 Hz, 1H), 5.92 (s, 2H). ^13^C NMR (101 MHz, DMSO): δ 147.73, 145.24, 143.78, 142.85, 136.75, 133.30, 133.14, 129.95, 129.39, 129.00, 126.55, 126.44, 125.70, 124.43, 117.40, 113.64, 111.80, 52.70. HRMS: calcd for [M - H], 430.09; found, 429.555.

##### 4-((4-(2-(4-Chlorophenyl)imidazo[1,2-*a*]pyridin-3-yl)-1*H*-1,2,3-triazol-1-yl)methyl)aniline (**15l**)

2-(4-Chlorophenyl)-3-(1-(4-nitrobenzyl)-1*H*-1,2,3-triazol-4-yl)imidazo[1,2-*a*]pyridine (**15k**) was dissolved in MeOH and AcOH (7 equiv), and Fe (3.5 equiv) was added. The reaction mixture was stirred at reflux until the completion of the reaction. After cooling to 25 °C, the mixture was extracted with EtOAc, washed with NaHCO_3_ solution, and the organic phase was dried over sodium sulfate and evaporated. The residue was purified by flash column chromatography, mobile phase cyclohexane/EtOAc (40–100%). Yield: 123 mg (78%). ^1^H NMR (401 MHz, DMSO-*d*_6_): δ 8.41 (dt, *J* = 7.0, 1.3 Hz, 1H), 8.39 (s, 1H), 7.72–7.65 (m, 3H), 7.43–7.32 (m, 3H), 7.10–7.04 (m, 2H), 6.98 (td, *J* = 6.8, 1.3 Hz, 1H), 6.59–6.53 (m, 2H), 5.48 (s, 2H), 5.20 (s, 2H). ^13^C NMR (101 MHz, DMSO): δ 149.31, 145.14, 142.57, 136.35, 133.35, 133.06, 129.86, 129.63, 128.90, 126.44, 125.58, 125.47, 122.83, 117.37, 114.23, 113.57, 112.10, 53.72. HRMS: calcd for [M + H], 401.12760; found, 401.12735.

##### *N*-(4-((4-(2-(4-Chlorophenyl)imidazo[1,2-*a*]pyridin-3-yl)-1*H*-1,2,3-triazol-1-yl)methyl)phenyl)-acetamide (**15m**)

4-((4-(2-(4-Chlorophenyl)imidazo[1,2-*a*]pyridin-3-yl)-1*H*-1,2,3-triazol-1-yl)methyl)aniline was dissolved in dioxane, and Ac_2_O (1.5 equiv) was added followed by pyridine (1.5 equiv). The reaction mixture was stirred at 25 °C overnight. After the completion of the reaction, the mixture was evaporated, diluted with EtOAc, and washed with water. The organic phase was dried over sodium sulfate and evaporated. The residue was purified by flash column chromatography. Mobile phase cyclohexane/EtOAc (40–100%). Yield: 63 mg (92%). ^1^H NMR (401 MHz, DMSO-*d*_6_): δ 10.02 (s, 1H), 8.45 (s, 1H), 8.45–8.42 (m, 1H), 7.72–7.66 (m, 3H), 7.63–7.58 (m, 2H), 7.39 (s, 2H), 7.39–7.34 (m, 1H), 7.33–7.28 (m, 2H), 6.99 (td, *J* = 6.8, 1.2 Hz, 1H), 5.65 (s, 2H), 2.04 (s, 3H). ^13^C NMR (101 MHz, DMSO): δ 168.54, 144.88, 142.37, 139.48, 136.20, 133.04, 132.78, 130.37, 129.59, 128.65, 128.62, 126.18, 125.58, 125.34, 119.31, 117.08, 113.30, 111.69, 66.52, 53.00, 24.17. HRMS: calcd for [M + H], 443.13816; found, 443.13773.

##### 5-(6-(4-Chlorophenyl)imidazo[2,1-*b*]thiazol-5-yl)-3-(3,4-dichlorobenzyl)-1,2,4-oxadiazole (**16A**)

6-(4-Chlorophenyl)imidazo[2,1-*b*]thiazole-5-carboxylic acid was dissolved in dry DMF, degassed, and refilled with argon. EDC (1 equiv) and HOBt (1 equiv) were added in one portion, and the mixture was stirred at 25 °C for 30 min. Then, a solution of (*E*)-2-(3,4-dichlorophenyl)-*N*′-hydroxyacetimidamide in dry DMF was added, and the reaction mixture was stirred at 80 °C overnight. After cooling to 25 °C, the mixture was diluted with EtOAc, washed with NaHCO_3_ solution, and water, and the organic phase was dried over sodium sulfate. The product was isolated by flash column chromatography, mobile phase petrol ether/EtOAc (50:70%). Yield: 250 mg (52%). ^1^H NMR (500 MHz, DMSO-*d*_6_): δ 8.33 (d, *J* = 4.4 Hz, 1H), 7.94–7.91 (m, 2H), 7.71 (d, *J* = 2.1 Hz, 1H), 7.63 (d, *J* = 4.0 Hz, 1H), 7.62 (s, 1H), 7.57–7.53 (m, 2H), 7.42–7.39 (m, 1H), 4.23 (s, 2H). ^13^C NMR (126 MHz, DMSO): δ 168.78, 167.94, 153.92, 150.51, 137.29, 134.31, 132.08, 131.65, 131.45, 131.25, 131.09, 130.18, 130.10, 128.70, 121.58, 117.20, 109.75, 30.64. HRMS: calcd for [M + H], 460.97919; found, 460.97921.

##### 5-(2-(4-Chlorophenyl)imidazo[1,2-*a*]pyridin-3-yl)-3-(3,4-dichlorobenzyl)-1,2,4-oxadiazole (**16B**)

2-(4-Chlorophenyl)imidazo[1,2-*a*]pyridine-3-carboxylic acid was dissolved in dry DMF, degassed, and refilled with argon. EDC (1 equiv) and HOBt (1 equiv) were added in one portion, and the mixture was stirred at 25 °C for 30 min. Then, a solution of (*E*)-2-(3,4-dichlorophenyl)-*N*′-hydroxyacetimidamide in dry DMF was added, and the reaction mixture was stirred at 80 °C overnight. After cooling to 25 °C, the mixture was diluted with EtOAc and washed with NaHCO_3_ solution and water, and the organic phase was dried over sodium sulfate. The product was isolated by flash column chromatography, mobile phase petrol ether/EtOAc (50:70%). Yield: 252 mg (50%). ^1^H NMR (401 MHz, DMSO-*d*_6_): δ 9.41 (dt, *J* = 6.9, 1.2 Hz, 1H), 7.90 (dt, *J* = 9.0, 1.2 Hz, 1H), 7.88–7.84 (m, 2H), 7.71 (d, *J* = 2.0 Hz, 1H), 7.67 (ddd, *J* = 9.0, 6.9, 1.3 Hz, 1H), 7.63 (d, *J* = 8.3 Hz, 1H), 7.60–7.54 (m, 2H), 7.42 (dd, *J* = 8.3, 2.1 Hz, 1H), 7.36 (td, *J* = 7.0, 1.3 Hz, 1H), 4.27 (s, 2H). ^13^C NMR (101 MHz, DMSO): δ 168.27, 167.90, 150.18, 147.19, 137.02, 134.29, 132.16, 131.54, 131.33, 131.19, 130.82, 129.90, 129.77, 129.38, 128.39, 128.01, 117.58, 115.47, 30.46. HRMS: calcd for [M + H], 455.02277; found, 455.02288.

##### 2-(2-(4-Chlorophenyl)imidazo[1,2-*a*]pyridin-3-yl)-5-(3,4-dichlorobenzyl)-1,3,4-oxadiazole (**17**)

2-(4-Chlorophenyl)-*N*′-(2-(3,4-dichloro-phenyl)acetyl)imidazo[1,2-*a*]pyridine-3-carbohydrazide was dissolved in dry DCM, and tosyl chloride (1.5 equiv) was added, followed by TEA (3 equiv) at 0 °C. The reaction mixture was stirred at 25 °C overnight. The mixture was diluted with water and extracted with EtOAc, and the organic phase was washed with a saturated NaHCO_3_ solution. The organic phase was dried over sodium sulfate. The product was isolated by flash column and RP-flash column chromatography; mobile phase hexane/EtOAc (30:60%), and (H_2_O/CH_3_CN 10:80%). Yield: 16 mg (6%). ^1^H NMR (500 MHz, DMSO-*d*_6_): δ 9.30 (dt, *J* = 7.0, 1.2 Hz, 1H), 7.82 (dt, *J* = 9.0, 1.2 Hz, 1H), 7.79–7.75 (m, 2H), 7.61–7.57 (m, 3H), 7.43–7.39 (m, 2H), 7.31–7.25 (m, 2H), 4.35 (s, 2H). ^13^C NMR (126 MHz, DMSO): δ 163.81, 158.08, 147.83, 146.98, 135.75, 134.31, 132.41, 131.77, 131.62, 131.32, 131.12, 130.70, 129.92, 128.65, 128.52, 127.96, 117.70, 115.12, 106.70, 30.17. HRMS: calcd for [M + H], 455.02277; found, 455.02282.

##### 2-(2-(4-Chlorophenyl)imidazo[1,2-*a*]pyridin-3-yl)-5-(3,4-dichlorobenzyl)-1,3,4-thiadiazole (**18**)

A round-bottom flask was charged with 2-(4-chlorophenyl)-*N*′-(2-(3,4-dichlorophenyl)acetyl)imidazo[1,2-*a*]pyridine-3-carbohydrazide, degassed, and refilled with argon. Dry toluene was added, and the mixture was degassed once more and refilled with argon. Lawesson’s reagent (3 equiv) was added, and the mixture was stirred at 100 °C overnight. After cooling to 25 °C, the mixture was diluted with water and extracted with EtOAc. An organic phase was dried over sodium sulfate and evaporated. A residue was purified by flash column chromatography. Mobile phase H_2_O/MeOH (30:100%). Yield: 44 mg (21%). ^1^H NMR (500 MHz, DMSO-*d*_6_): δ 9.35 (dt, *J* = 7.0, 1.2 Hz, 1H), 7.85 (dt, *J* = 9.0, 1.2 Hz, 1H), 7.78–7.73 (m, 2H), 7.65–7.57 (m, 3H), 7.42–7.38 (m, 2H), 7.31 (ddd, *J* = 8.2, 4.5, 1.7 Hz, 2H), 4.35 (s, 2H). ^13^C NMR (126 MHz, DMSO): δ 163.75, 158.07, 147.63, 146.86, 135.84, 134.23, 132.26, 131.72, 131.62, 131.43, 131.16, 130.56, 130.10, 128.84, 128.55, 128.08, 117.70, 115.31, 106.65, 29.99. HRMS: calcd for [M + Na], 477.00472; found, 477.00467.

##### 6-(4-Chlorophenyl)-5-(2-(3,4-dichlorobenzyl)thiazol-4-yl)imidazo[2,1-*b*]thiazole (**19A**)

2-Chloro-1-(6-(4-chlorophenyl)imidazo[2,1-*b*]thiazol-5-yl)ethan-1-one (34) was dissolved in EtOH and 2-(3,4-dichlorophenyl)ethanethioamide (1.5 equiv) was added. A reaction mixture was stirred at reflux overnight. After cooling to 25 °C, the mixture was purified by RP-flash column chromatography. Mobile phase H_2_O/CH_3_CN (20:80%). Yield: 213 mg (61%). ^1^H NMR (401 MHz, DMSO-*d*_6_): δ 8.01 (d, *J* = 4.5 Hz, 1H), 7.71 (d, *J* = 2.0 Hz, 1H), 7.68–7.61 (m, 4H), 7.42–7.36 (m, 4H), 4.46 (s, 2H). ^13^C NMR (101 MHz, DMSO): δ 169.35, 149.37, 143.99, 143.09, 139.54, 133.79, 132.56, 131.60, 131.53, 131.24, 130.15, 130.04, 129.84, 128.83, 120.43, 118.35, 117.72, 114.49, 37.55. HRMS: calcd for [M + H], 475.96110; found, 475.96130.

##### 4-(2-(4-Chlorophenyl)imidazo[1,2-*a*]pyridin-3-yl)-2-(3,4-dichlorobenzyl)thiazole (**19B**)

2-Chloro-1-(2-(4-chlorophenyl)imidazo[1,2-*a*]pyridin-3-yl)ethan-1-one (35) was dissolved in EtOH, and 2-(3,4-dichlorophenyl)ethanethioamide (1.5 equiv) was added. A reaction mixture was stirred at reflux overnight. After cooling to 25 °C, the mixture was purified by RP-flash column chromatography. Mobile phase: H_2_O/CH_3_CN (20:80%). Yield: 112 mg (62%). ^1^H NMR (401 MHz, DMSO-*d*_6_): δ 8.42 (d, *J* = 6.9 Hz, 1H), 7.83 (s, 1H), 7.74 (d, *J* = 2.1 Hz, 1H), 7.67 (q, *J* = 8.1 Hz, 4H), 7.45–7.35 (m, 4H), 6.99 (t, *J* = 6.9 Hz, 1H), 4.51 (s, 2H). ^13^C NMR (101 MHz, DMSO): δ 169.57, 144.48, 143.11, 142.15, 139.27, 133.16, 132.66, 131.29, 131.25, 130.96, 129.86, 129.73, 129.68, 128.56, 126.10, 125.41, 121.39, 117.09, 115.60, 113.16, 37.34. HRMS: calcd for [M + H], 470.00468; found, 470.00488.

##### 3-Benzyl-5-(2-(4-chlorophenyl)imidazo[1,2-*a*]pyridin-3-yl)isoxazole (**20**)

Phenylacetaldehyde (0.045 mL, 1 equiv) was dissolved in an H_2_O/*t*-BuOH mixture (4 mL) and NH_2_OH·HCl (30 mg, 1 equiv), followed by NaOH (20 mg, 1 equiv) addition. The reaction mixture was stirred at rt for 3 h; then, 2-(4-chlorophenyl)-3-ethynylimidazo[1,2-*a*]pyridine **33** (100 mg, 0.39 mmol), chloramine T (120 mg, 1 equiv), and CuI (8 mg, 10 mol %) were added; and the reaction mixture was stirred overnight. The reaction mixture was diluted with water and extracted with EtOAc. Combined organic phases were dried over sodium sulfate and evaporated. The residue was purified by RP-flash column chromatography. Mobile phase H_2_O/CH_3_CN (10:100%). Yield: 99 mg (66%). ^1^H NMR (401 MHz, DMSO-*d*_6_): δ 8.48 (dt, *J* = 6.9, 1.1 Hz, 1H), 7.76 (dt, *J* = 9.1, 1.2 Hz, 1H), 7.68–7.63 (m, 2H), 7.51–7.46 (m, 3H), 7.35 (m, 4H), 7.26 (ddt, *J* = 6.9, 4.6, 3.1 Hz, 1H), 7.12 (td, *J* = 6.9, 1.2 Hz, 1H), 6.82 (s, 1H), 4.11 (s, 2H). ^13^C NMR (101 MHz, DMSO): δ 163.90, 160.02, 146.13, 145.18, 137.73, 133.78, 132.59, 130.33, 129.23, 129.09, 129.04, 127.82, 127.16, 126.15, 117.66, 114.67, 105.32, 31.94. Anal. (C_23_H_16_ClN_3_O·0.75H_2_O): C, H, N. HRMS: calcd for [M + H], 386.10547; found, 386.10564. EA: C, 69.17; H, 4.42; N, 10.52. Found: C, 68.94; H, 4.11; N, 10.41.

##### 2-(4-Chlorophenyl)-3-(1-(3,4-dichlorobenzyl)-1*H*-pyrrol-2-yl)imidazo[1,2-*a*]pyridine (**21**)

2-(4-Chlorophenyl)-3-(1*H*-pyrrol-2-yl)imidazo[1,2-*a*]pyridine **35** (76 mg, 0.26 mmol) was dissolved in dry DMF (2 mL), degassed, and refilled with argon. NaH (10 mg, 1.3 equiv, 60% in mineral oil) was added, and the mixture was stirred at 25 °C for 30 min. 1,2-Dichloro-4-(chloromethyl)benzene (0.05 mL, 1.3 equiv) was added, and the reaction mixture was stirred at 25 °C overnight. Then, the mixture was diluted with water and extracted with EtOAc. Combined organic phases were dried over sodium sulfate and evaporated. The residue was purified by flash column chromatography, mobile phase petrol ether/EtOAc (15:50%). Yield: 25 mg (21%). ^1^H NMR (401 MHz, DMSO-*d*_6_): δ 7.57 (m, 3H), 7.47 (d, *J* = 6.8 Hz, 1H), 7.39 (dd, *J* = 2.7, 1.7 Hz, 1H), 7.38–7.33 (m, 2H), 7.27 (ddd, *J* = 9.0, 6.7, 1.2 Hz, 1H), 7.17 (d, *J* = 8.2 Hz, 1H), 6.74 (td, *J* = 6.8, 1.1 Hz, 1H), 6.64 (d, *J* = 2.0 Hz, 1H), 6.58 (dd, *J* = 8.3, 2.0 Hz, 1H), 6.51 (dd, *J* = 3.6, 1.7 Hz, 1H), 6.43–6.38 (m, 1H), 4.80 (d, *J* = 15.4 Hz, H), 4.53 (d, *J* = 15.4 Hz, 1H). ^13^C NMR (101 MHz, DMSO): δ 144.60, 142.39, 138.69, 132.84, 132.59, 130.86, 130.34, 129.80, 128.70, 128.62, 127.99, 127.04, 126.00, 125.48, 123.94, 118.69, 116.79, 113.98, 112.70, 112.19, 109.16, 49.78. HRMS: calcd for [M + H], 452.04826; found, 452.04842.

##### 2-(4-Chlorophenyl)-3-(1-(3,4-dichlorobenzyl)-1*H*-pyrazol-4-yl)imidazo[1,2-*a*]pyridine (**22**)

2-(4-Chlorophenyl)-3-(1*H*-pyrazol-4-yl)imidazo[1,2-*a*]pyridine **36** (30 mg, 0.1 mmol) was dissolved in CH_3_CN (2 mL), and the solution was degassed and refilled with argon. K_2_CO_3_ (15 mg, 1.1 equiv) was added, followed by the addition of 1,2-dichloro-4-(chloromethyl)benzene (0.02 mL, 1 equiv). The reaction mixture was refluxed overnight under an argon atmosphere. The product was isolated from preparative TLC; mobile phase: petrol ether/EtOAc 3:2. Yield: 15 mg (32%). ^1^H NMR (401 MHz, DMSO-*d*_6_): δ 8.26 (s, 0H), 8.09 (dd, *J* = 6.9, 1.5 Hz, 0H), 7.80 (s, 0H), 7.73–7.68 (m, 1H), 7.68–7.60 (m, 1H), 7.58 (d, *J* = 2.1 Hz, 1H), 7.41 (dd, *J* = 8.5, 1.7 Hz, 1H), 7.36–7.24 (m, 1H), 6.97–6.90 (m, 1H), 5.49 (s, 1H). ^13^C NMR (101 MHz, DMSO): δ 144.74, 141.23, 140.90, 139.00, 133.78, 132.57, 132.37, 131.36, 130.87, 130.02, 129.79, 129.36, 128.82, 128.38, 125.81, 124.78, 117.28, 113.42, 113.20, 109.08, 54.16. HRMS: calcd for [M + H], 453.04351; found, 453.04356.

##### 2-(4-Chlorophenyl)-3-(1-(3,4-dichlorophenyl)-1*H*-1,2,3-triazol-4-yl)imidazo[1,2-*a*]pyridine (**23**)

The title compound was prepared according to General Procedure IV. Mobile phase petrol ether/EtOAc (30:100%). Yield: 225 mg (73%). ^1^H NMR (401 MHz, DMSO-*d*_6_): δ 9.31 (s, 1H), 8.49 (dt, *J* = 6.9, 1.1 Hz, 2H), 8.37 (d, *J* = 2.5 Hz, 1H), 8.07 (dd, *J* = 8.8, 2.5 Hz, 1H), 7.95 (d, *J* = 8.8 Hz, 1H), 7.85–7.78 (m, 3H), 7.73 (dt, *J* = 9.1, 1.0 Hz, 2H), 7.48–7.44 (m, 3H), 7.44–7.39 (m, 1H), 7.02 (td, *J* = 6.8, 1.2 Hz, 1H). ^13^C NMR (101 MHz, DMSO): δ 145.10, 142.84, 136.98, 136.19, 132.88, 132.84, 132.55, 132.06, 131.49, 129.65, 128.73, 126.53, 125.45, 124.34, 122.30, 120.57, 117.14, 113.39, 110.89. HRMS: calcd for [M + H], 440.02311; found, 440.02321.

##### 2-(4-Chlorophenyl)-3-(1-(3,4-dichlorophenethyl)-1*H*-1,2,3-triazol-4-yl)imidazo[1,2-*a*]pyridine (**24**)

The title compound was prepared according to General Procedure IV. Mobile phase petrol ether/EtOAc (30:100%). Yield: 138 mg (95%). ^1^H NMR (401 MHz, DMSO-*d*_6_): δ 8.33 (s, 0H), 8.25 (d, *J* = 6.9 Hz, 1H), 7.69 (d, *J* = 9.0 Hz, 1H), 7.64 (d, *J* = 8.5 Hz, 1H), 7.58–7.49 (m, 1H), 7.39 (d, *J* = 8.5 Hz, 2H), 7.21–7.13 (m, 1H), 6.99 (t, *J* = 6.6 Hz, 1H), 4.78 (t, *J* = 6.7 Hz, 1H), 3.26 (t, *J* = 6.7 Hz, 1H). ^13^C NMR (101 MHz, DMSO): δ 144.84, 142.21, 139.06, 135.60, 132.98, 132.77, 131.08, 131.05, 130.64, 129.49, 129.46, 129.40, 128.61, 126.20, 125.77, 125.00, 117.13, 113.27, 111.64, 50.51, 34.70. HRMS: calcd for [M + H], 468.05441; found, 468.05450.

##### Ethyl 6-(4-Chlorophenyl)imidazo[2,1-*b*]thiazole-5-carboxylate (**25**)

2-Aminothiazole (3 equiv) was dissolved in CH_3_CN, and ethyl 3-(4-chlorophenyl)-3-oxopropanoate (1 equiv) was added as a solution in CH_3_CN, followed by the addition of CBr_4_ (2 equiv). The reaction mixture was stirred at 80 °C overnight. After the completion of the reaction, the mixture was evaporated to a minimal volume, diluted with water, and extracted with EtOAc. Combined organic phases were dried over sodium sulfate and purified by flash column chromatography. Mobile phase petrol ether/EtOAc (25:50%). Yield: 365 mg (82%). ^1^H NMR (401 MHz, DMSO-*d*_6_): δ 8.22 (d, *J* = 4.5 Hz, 1H), 7.89–7.85 (m, 2H), 7.54 (d, *J* = 4.5 Hz, 2H), 7.52–7.48 (m, 2H), 4.30 (q, *J* = 7.1 Hz, 2H), 1.27 (t, *J* = 7.1 Hz, 3H). ^13^C NMR (101 MHz, DMSO): δ 159.18, 152.65, 151.43, 133.50, 132.49, 131.49, 127.90, 121.94, 116.12, 114.53, 60.83, 14.12. HRMS: calcd for [M + H], 307.03025; found, 307.03034.

##### Ethyl 2-(4-Chlorophenyl)imidazo[1,2-*a*]pyridine-3-carboxylate (**26**)

2-Aminopyridine (3 equiv) was dissolved in CH_3_CN, and ethyl 3-(4-chlorophenyl)-3-oxopropanoate (1 equiv) was added as a solution in CH_3_CN followed by the addition of CBr_4_ (2 equiv). A reaction mixture was stirred at 80 °C overnight. After the completion of the reaction, the mixture was evaporated to a minimal volume, diluted with water, and extracted with EtOAc. Combined organic phases were dried over sodium sulfate and purified by flash column chromatography. Spectral characteristics match those described in the literature.^46^

##### 6-(4-Chlorophenyl)imidazo[2,1-*b*]thiazole-5-carboxylic Acid (**27**)

The title compound was prepared according to General Procedure V with a minor modification. The product was filtered as a precipitate after acidification. Yield: 286 mg (90%). Spectral characteristics matched those described in the literature.^47^

##### 2-(4-Chlorophenyl)imidazo[1,2-*a*]pyridine-3-carboxylic Acid (**28**)

The title compound was prepared according to General Procedure V with a minor modification. The product was filtered as a precipitate after acidification. Yield: 548 mg (quant.). ^1^H NMR (401 MHz, DMSO-*d*_6_): δ 9.40–9.32 (m, 1H), 7.83–7.75 (m, 3H), 7.59–7.52 (m, 1H), 7.50 (dd, *J* = 9.0, 2.5 Hz, 2H), 7.21 (td, *J* = 6.9, 1.3 Hz, 1H). ^13^C NMR (101 MHz, DMSO): δ 162.11, 151.23, 146.75, 133.77, 133.70, 132.35, 129.28, 128.92, 128.80, 128.04, 127.77, 117.53, 115.02, 112.51. HRMS: calcd for [M + H], 273.04253; found, 273.04262.

##### 2-(4-Chlorophenyl)imidazo[1,2-*a*]pyridine-3-carbohydrazide (**29**)

Ester derivative was dissolved in absolute EtOH and N_2_H_4_·H_2_O (10 equiv) was added. The reaction mixture was refluxed overnight. The reaction mixture was cooled to 25 °C, and the formed precipitate was filtered, washed with EtOH, and dried. Yield: 412 mg (87%). ^1^H NMR (401 MHz, DMSO-*d*_6_): δ 9.70 (s, 1H), 8.57 (d, *J* = 7.0 Hz, 1H), 7.92–7.77 (m, 2H), 7.66 (d, *J* = 9.1 Hz, 1H), 7.56–7.43 (m, 2H), 7.40 (t, *J* = 8.0 Hz, 1H), 7.04 (t, *J* = 6.9 Hz, 1H), 4.67 (s, 2H). ^13^C NMR (101 MHz, DMSO): δ 160.38, 144.68, 143.00, 132.96, 132.64, 129.97, 128.57, 126.85, 126.37, 117.04, 115.62, 113.53. HRMS: calcd for [M + H], 287.06942; found, 287.06950.

##### 2-(4-Chlorophenyl)-*N*′-(2-(3,4-dichlorophenyl)acetyl)imidazo[1,2-*a*]pyridine-3-carbohydrazide (**30**)

2-(4-Chlorophenyl)imidazo[1,2-*a*]pyridine-3-carbohydrazide was dissolved with dry DMF and ethyl 2-(4-chlorophenyl)imidazo[1,2-*a*]pyridine-3-carboxylate (1 equiv) was added. The reaction mixture was degassed and refilled with argon. HATU (1.2 equiv) was added, followed by the addition of DIPEA (1.2 equiv). The reaction mixture was stirred at 25 °C and monitored by UPLC. After the completion of the reaction, the solution was evaporated to a minimal volume and purified by reverse-phase flash column chromatography. Mobile phase petrol ether/EtOAc (40:80%). Yield: 298 mg (90%). ^1^H NMR (401 MHz, DMSO-*d*_6_): δ 10.49 (s, 1H), 10.43 (s, 1H), 8.80 (dt, *J* = 7.0, 1.2 Hz, 1H), 8.06–7.98 (m, 2H), 7.95 (s, 1H), 7.71 (dt, *J* = 9.0, 1.2 Hz, 1H), 7.65–7.60 (m, 2H), 7.49 (dd, *J* = 9.0, 2.3 Hz, 2H), 7.44 (ddd, *J* = 9.0, 6.8, 1.3 Hz, 1H), 7.34 (dd, *J* = 8.3, 2.1 Hz, 1H), 7.10 (td, *J* = 6.9, 1.2 Hz, 1H), 3.63 (s, 2H). ^13^C NMR (101 MHz, DMSO): δ 169.21, 160.31, 145.02, 144.01, 136.78, 133.29, 132.28, 131.35, 130.97, 130.62, 130.18, 129.86, 129.58, 128.59, 127.33, 126.68, 117.16, 114.63, 113.89, 35.98. HRMS: calcd for [M + H], 473.03334; found, 473.03327.

##### 2-Chloro-1-(6-(4-chlorophenyl)imidazo[2,1-*b*]thiazol-5-yl)ethan-1-one (**31**)

6-(4-Chlorophenyl)imidazo[2,1-*b*]thiazole (1 mmol) was dissolved in dry dioxane (4 mL), and chloroacetyl chloride (3 equiv) was added in one portion. The reaction mixture was stirred at 70 °C under an argon atmosphere for 30 min and then at 100 °C overnight. After cooling to 25 °C, a precipitate was formed. The suspension was diluted with a saturated NaHCO_3_ solution and extracted with EtOAc. The organic phase was dried over sodium sulfate and purified by flash column chromatography, mobile phase petrol ether/EtOAc (30:60%). Yield: 273 mg (91%). ^1^H NMR (401 MHz, DMSO-*d*_6_): δ 8.46 (d, *J* = 4.4 Hz, 1H), 7.73–7.68 (m, 2H), 7.64 (d, *J* = 4.4 Hz, 1H), 7.62–7.57 (m, 2H), 4.41 (s, 2H). ^13^C NMR (101 MHz, DMSO): δ 180.76, 154.65, 153.23, 134.82, 133.38, 132.00, 129.02, 122.62, 122.19, 117.57, 47.18. HRMS: calcd for [M + H], 310.98072; found, 310.98089.

##### 2-Chloro-1-(2-(4-chlorophenyl)imidazo[1,2-*a*]pyridin-3-yl)ethan-1-one (**32**)

2-(4-Chlorophenyl)imidazo[1,2-*a*]pyridine (1 mmol) was dissolved in dry dioxane (4 mL) and chloroacetyl chloride (3 equiv) was added in one portion. The reaction mixture was stirred at 70 °C under an argon atmosphere for 30 min and then at 100 °C overnight. After cooling to 25 °C, a precipitate was formed. The suspension was diluted with a saturated NaHCO_3_ solution and extracted with EtOAc. The organic phase was dried over sodium sulfate and purified by flash column chromatography; mobile phase petrol ether/EtOAc (30:60%). Yield: 492 mg (76%). ^1^H NMR (401 MHz, DMSO-*d*_6_): δ 9.63 (d, *J* = 6.9 Hz, 1H), 7.94 (d, *J* = 8.9 Hz, 1H), 7.85 (t, *J* = 7.9 Hz, 1H), 7.73 (d, *J* = 8.2 Hz, 2H), 7.65 (d, *J* = 8.2 Hz, 2H), 7.45 (t, *J* = 6.9 Hz, 1H), 4.36 (s, 2H). ^13^C NMR (101 MHz, DMSO): δ 182.37, 151.37, 145.94, 135.44, 132.49, 132.19, 132.15, 129.23, 129.22, 119.42, 117.34, 116.76, 47.93. HRMS: calcd for [M + H], 305.02429; found, 305.02434.

##### 2-(4-Chlorophenyl)-3-ethynylimidazo[1,2-*a*]pyridine (**33**)

2-(4-Chlorophenyl)-3-((trimethylsilyl)ethynyl)imidazo[1,2-*a*]pyridine (**11**, 513 mg, 1.58 mmol) was dissolved in MeOH and K_2_CO_3_ (435 mg, 2 equiv) was added in one portion. The reaction was stirred at 25 °C and monitored by TLC. After the completion of the reaction, the mixture was diluted with DCM and washed with water. Combined organic phases were dried over sodium sulfate and evaporated, and the residue was purified by flash column chromatography. Mobile phase petrol ether/EtOAc (30:50%). Yield: 362 mg (90%). ^1^H NMR (401 MHz, DMSO-*d*_6_): δ 8.46 (dt, *J* = 6.8, 1.2 Hz, 1H), 8.28–8.20 (m, 2H), 7.71 (dt, *J* = 9.0, 1.1 Hz, 1H), 7.60–7.52 (m, 2H), 7.45 (ddd, *J* = 9.0, 6.8, 1.3 Hz, 1H), 7.12 (td, *J* = 6.8, 1.2 Hz, 1H), 5.44 (s, 1H). ^13^C NMR (101 MHz, DMSO): δ 145.95, 144.71, 133.40, 131.99, 128.97, 128.32, 128.28, 127.70, 125.77, 117.27, 114.16, 103.47, 94.39, 72.47. HRMS: calcd for [M + Na], 253.05270; found, 253.05273.

##### 2-(4-Chlorophenyl)-3-(1*H*-1,2,3-triazol-4-yl)imidazo[1,2-*a*]pyridine (**34**)

2-(4-Chlorophenyl)-3-ethynylimidazo[1,2-*a*]pyridine **33** (100 mg, 0.396 mmol) was dissolved in DMF/MeOH (10:1) mixture, degassed, and purged with argon. To this solution, CuI (10 mol %) and TMSN_3_ (1 equiv) were added and the mixture was stirred at 70 °C overnight. After the completion of the reaction, the mixture was diluted with EtOAc and washed with water, and the combined organic phases were dried over sodium sulfate and evaporated. The residue was purified by flash column chromatography, mobile phase cyclohexane/EtOAc (10:70%). Yield: 98 mg (84%). ^1^H NMR (401 MHz, DMSO-*d*_6_): δ 8.42 (dt, *J* = 7.0, 1.2 Hz, 1H), 8.13 (s, 1H), 7.76–7.66 (m, 3H), 7.47–7.41 (m, 2H), 7.37 (ddd, *J* = 9.0, 6.7, 1.2 Hz, 1H), 6.99 (td, *J* = 6.8, 1.2 Hz, 1H). ^13^C NMR (101 MHz, DMSO): δ 145.21, 142.71, 135.51, 133.42, 133.08, 130.22, 129.90, 129.90, 128.95, 126.44, 125.60, 117.35, 113.58, 112.24. HRMS: calcd for [M + H], 296.06975; found, 296.06964.

##### 2-(4-Chlorophenyl)-3-(1*H*-pyrrol-2-yl)imidazo[1,2-*a*]pyridine (**35**)

2-(4-Chlorophenyl)-3-iodoimidazo[1,2-*a*]pyridine (430 mg, 1.21 mmol) was dissolved in dioxane (12 mL) and (1-(*tert*-butoxycarbonyl)-1*H*-pyrrol-3-yl)boronic acid (260 mg, 1 equiv) was added, followed by a solution of Na_2_CO_3_ (392 mg, 3 equiv) in 3 mL of H_2_O. A reaction mixture was degassed and refilled with argon. Pd(PPh_3_)_4_ (72 mg, 5% mol) was added, and the mixture was degassed again and refilled with argon. The reaction mixture was stirred at 90 °C overnight. After cooling to 25 °C, the mixture was diluted with water and extracted with EtOAc. Combined organic phases were dried over sodium sulfate and evaporated. The residue was purified by flash column chromatography; mobile phase petrol ether/EtOAc (20:70%). Yield: 103 mg (35%). ^1^H NMR (401 MHz, DMSO-*d*_6_): δ 11.28 (s, 1H), 7.99 (dt, *J* = 6.9, 1.2 Hz, 1H), 7.69–7.62 (m, 3H), 7.42–7.37 (m, 2H), 7.32 (ddd, *J* = 9.1, 6.7, 1.3 Hz, 1H), 7.07 (td, *J* = 2.7, 1.5 Hz, 1H), 6.93 (td, *J* = 6.8, 1.2 Hz, 1H), 6.40–6.37 (m, 1H), 6.34 (dt, *J* = 3.3, 2.5 Hz, 1H). ^13^C NMR (101 MHz, DMSO): δ 144.27, 141.30, 133.34, 132.24, 128.58, 128.51, 125.77, 124.66, 120.79, 117.38, 116.91, 114.57, 112.91, 111.12, 109.48. HRMS: calcd for [M + H], 294.07925; found, 294.07926.

##### 2-(4-Chlorophenyl)-3-(1*H*-pyrazol-4-yl)imidazo[1,2-*a*]pyridine (**36**)

2-(4-Chlorophenyl)-3-iodoimidazo[1,2-*a*]pyridine (334 mg, 0.94 mmol) was dissolved in dioxane (8 mL) and 4-(4,4,5,5-tetramethyl-1,3,2-dioxaborolan-2-yl)-1*H*-pyrazole (183 mg, 1 equiv) was added, followed by a solution of Na_2_CO_3_ (3 equiv) in 2 mL of H_2_O. A reaction mixture was degassed and refilled with argon. Pd(dppf)Cl_2_ (40 mg, 5% mol) was added, and the mixture was degassed again and refilled with argon. The reaction mixture was stirred at 90 °C overnight. After cooling to 25 °C, the mixture was diluted with water and extracted with EtOAc. Combined organic phases were dried over sodium sulfate and evaporated. The residue was purified by flash column chromatography; mobile phase petrol ether/EtOAc (20:70%). Yield: 112 mg (40%). ^1^H NMR (401 MHz, DMSO-*d*_6_): δ 8.54 (d, *J* = 6.7 Hz, 1H), 8.45 (s, 1H), 8.00 (d, *J* = 8.3 Hz, 2H), 7.59 (d, *J* = 9.1 Hz, 1H), 7.51 (d, *J* = 8.3 Hz, 2H), 7.29–7.22 (m, 1H), 6.92 (t, *J* = 6.7 Hz, 1H). HRMS: calcd for [M + H], 295.07450; found, 295.07467.

##### Methyl 2-Chloro-5-((4-(2-(4-chlorophenyl)imidazo[1,2-*a*]pyridin-3-yl)-1*H*-1,2,3-triazol-1-yl)-methyl)benzoate (**37**)

The title compound was prepared according to General Procedure IV. Mobile phase petrol ether/EtOAc (60:90%). Yield: 456 mg (84%). ^1^H NMR (401 MHz, DMSO-*d*_6_): δ 8.54 (s, 1H), 8.46 (dt, *J* = 7.0, 1.1 Hz, 1H), 7.84–7.81 (m, 1H), 7.72–7.67 (m, 3H), 7.65 (d, *J* = 8.3 Hz, 1H), 7.56 (dd, *J* = 8.3, 2.3 Hz, 1H), 7.44–7.40 (m, 2H), 7.40–7.36 (m, 1H), 7.00 (td, *J* = 6.8, 1.2 Hz, 1H), 5.79 (s, 2H), 3.87 (s, 3H). ^13^C NMR (101 MHz, DMSO): δ 165.39, 144.95, 142.46, 136.39, 135.56, 133.01, 132.84, 132.82, 131.82, 131.52, 130.65, 130.46, 129.62, 128.68, 126.29, 125.94, 125.39, 117.12, 113.37, 111.55, 52.89, 52.07. HRMS: calcd for [M + H], 478.08328; found, 478.08321.

##### 2-Chloro-5-((4-(2-(4-chlorophenyl)imidazo[1,2-*a*]pyridin-3-yl)-1*H*-1,2,3-triazol-1-yl)methyl)-benzoic Acid (**38**)

The title compound was prepared according to General Procedure V. Mobile phase: H_2_O/MeOH (10:80%). Yield: 528 mg (92%). ^1^H NMR (401 MHz, DMSO-*d*_6_): δ 8.56 (s, 1H), 8.46 (d, *J* = 6.9 Hz, 1H), 7.78 (d, *J* = 2.3 Hz, 1H), 7.73–7.67 (m, 3H), 7.62 (d, *J* = 8.3 Hz, 1H), 7.52 (dd, *J* = 8.3, 2.3 Hz, 1H), 7.41 (dd, *J* = 18.9, 8.6 Hz, 3H), 7.01 (t, *J* = 6.8 Hz, 1H), 5.79 (s, 2H). ^13^C NMR (101 MHz, DMSO): δ 166.58, 144.94, 142.42, 136.36, 135.43, 133.00, 132.85, 132.14, 131.83, 131.66, 131.37, 130.37, 129.57, 128.70, 126.28, 125.98, 125.36, 117.11, 113.35, 111.54, 52.11. HRMS: calcd for [M + H], 430.10653; found, 430.10615.

##### 2-Chloro-5-((4-(2-(4-chlorophenyl)imidazo[1,2-*a*]pyridin-3-yl)-1*H*-1,2,3-triazol-1-yl)methyl)benzamide (**39**)

The title compound was prepared according to General Procedure VI. Mobile phase: H_2_O/MeOH (30:100%). Yield: 251 mg (88%). ^1^H NMR (401 MHz, DMSO-*d*_6_): δ 8.56 (s, 1H), 8.51–8.43 (m, 1H), 8.00–7.84 (m, 1H), 7.70 (td, *J* = 6.8, 2.1 Hz, 4H), 7.53 (t, *J* = 7.3 Hz, 1H), 7.49–7.34 (m, 4H), 7.00 (qd, *J* = 6.8, 6.1, 1.1 Hz, 1H), 5.76 (s, 2H). ^13^C NMR (101 MHz, DMSO): δ 167.85, 140.71, 137.56, 135.11, 134.72, 134.37, 133.41, 133.00, 130.31, 130.17, 129.63, 129.36, 128.39, 127.21, 127.06, 126.55, 117.19, 113.25, 113.19, 52.31. HRMS: calcd for [M + H], 463.08354; found, 463.08359.

##### 3-(1-(3-Carbamoyl-4-chlorobenzyl)-1*H*-1,2,3-triazol-4-yl)-2-(4-chlorophenyl)imidazo[1,2-*a*]pyridin-1-ium Chloride (**39HCl**)

2-Chloro-5-((4-(2-(4-chlorophenyl)imidazo[1,2-*a*]pyridin-3-yl)-1*H*-1,2,3-triazol-1-yl)methyl)benzamide was dissolved in THF and cooled in an ice bath, and HCl 1 M in ether was added while stirring vigorously. The formed salt was stirred at 25 °C for 20 min, diluted with dry diethylether, filtered, and washed with more diethylether. EA: C, 54.96%; H, 3.51%; N, 16.54%; Cl, 21.48%.

##### 2-Chloro-5-((4-(2-(4-chlorophenyl)imidazo[1,2-*a*]pyridin-3-yl)-1*H*-1,2,3-triazol-1-yl)methyl)-*N*-methylbenzamide (**40**)

The title compound was prepared according to General Procedure VI. Mobile phase: H_2_O/MeOH (30:100%). Yield: 87 mg (89%). ^1^H NMR (401 MHz, DMSO-*d*_6_): δ 8.57 (s, 1H), 8.47 (dt, *J* = 7.0, 1.1 Hz, 1H), 8.41 (q, *J* = 4.4 Hz, 1H), 7.72–7.67 (m, 3H), 7.55 (d, *J* = 8.2 Hz, 1H), 7.47–7.41 (m, 4H), 7.39 (ddd, *J* = 9.1, 6.7, 1.2 Hz, 1H), 7.00 (td, *J* = 6.8, 1.2 Hz, 1H), 5.75 (s, 2H), 2.77 (d, *J* = 4.6 Hz, 3H). ^13^C NMR (101 MHz, DMSO): δ 166.58, 144.96, 142.43, 137.62, 136.35, 135.20, 133.01, 132.89, 130.40, 130.24, 129.86, 129.60, 128.75, 128.44, 126.31, 125.94, 125.40, 117.13, 113.38, 111.57, 52.22, 26.17. HRMS: calcd for [M + H], 477.09919; found, 477.09927.

##### 2-Chloro-5-((4-(2-(4-chlorophenyl)imidazo[1,2-*a*]pyridin-3-yl)-1*H*-1,2,3-triazol-1-yl)methyl)-*N*,*N*-dimethylbenzamide (**41**)

The title compound was prepared according to General Procedure VI. Mobile phase: H_2_O/MeOH (30:100%). Yield: 228 mg (95%). ^1^H NMR (401 MHz, DMSO-*d*_6_): δ 8.54 (s, 1H), 8.46 (dt, *J* = 6.9, 1.2 Hz, 1H), 8.31 (s, 1H), 7.71–7.66 (m, 3H), 7.58 (d, *J* = 8.3 Hz, 1H), 7.43 (dd, *J* = 8.6, 2.2 Hz, 3H), 7.38 (ddd, *J* = 9.1, 6.7, 1.3 Hz, 1H), 7.35 (d, *J* = 2.2 Hz, 1H), 6.99 (td, *J* = 6.8, 1.2 Hz, 1H), 5.76 (s, 2H), 3.02 (s, 3H), 2.75 (s, 3H). ^13^C NMR (101 MHz, DMSO): δ 166.79, 144.94, 142.44, 136.80, 136.38, 135.78, 133.01, 132.85, 130.03, 130.02, 129.57, 129.06, 128.70, 127.44, 126.26, 125.92, 125.37, 117.11, 113.34, 111.55, 52.23, 37.65, 34.22. HRMS: calcd for [M + H], 491.11484; found, 491.11493.

##### 2-Chloro-5-((4-(2-(4-chlorophenyl)imidazo[1,2-*a*]pyridin-3-yl)-1*H*-1,2,3-triazol-1-yl)methyl)-*N*-methoxy-*N*-methylbenzamide (**42**)

The title compound was prepared according to General Procedure VI with a minor modification. 2-Chloro-5-((4-(2-(4-chlorophenyl)imidazo[1,2-*a*]pyridin-3-yl)-1*H*-1,2,3-triazol-1-yl)methyl)-benzoic acid (528 mg, 1.13 mmol) was dissolved in dry DCM (8 mL) and cooled in an ice bath. Oxalyl chloride (0.2 mL, 2 equiv) was added followed by a catalytic amount of DMF. The reaction mixture was stirred at 25 °C overnight. The solvent was evaporated, and crude acyl chloride was used in the next step without further purification. Crude acyl chloride was dissolved in dry DCM (10 mL), and *N*,*O*-dimethylhydroxylamine hydrochloride (112 mg, 1 equiv) was added followed by TEA (0.32 mL, 2 equiv). The reaction mixture was stirred at 25 °C for 3 h. Then the mixture was diluted with DCM, washed with water, and purified by flash column chromatography, mobile phase petrol ether/EtOAc (60:100%). Yield: 521 (91%). ^1^H NMR (401 MHz, DMSO-*d*_6_): δ 8.55 (s, 1H), 8.50–8.39 (m, 1H), 7.69 (dd, *J* = 8.7, 2.0 Hz, 3H), 7.58 (d, *J* = 8.1 Hz, 2H), 7.48–7.40 (m, 4H), 7.38 (ddd, *J* = 9.0, 6.7, 1.3 Hz, 1H), 6.99 (td, *J* = 6.8, 1.2 Hz, 1H), 5.78 (s, 2H), 3.39 (s, 3H), 3.29 (s, 3H). ^13^C NMR (101 MHz, DMSO): δ 166.91, 144.93, 142.44, 136.35, 135.93, 135.22, 133.01, 132.85, 130.22, 129.83, 129.57, 129.40, 128.69, 127.15, 126.24, 125.90, 125.34, 117.11, 113.32, 111.55, 61.20, 52.22, 31.99. HRMS: calcd for [M + H], 507.10976; found, 507.10983.

##### 1-(2-Chloro-5-((4-(2-(4-chlorophenyl)imidazo[1,2-*a*]pyridin-3-yl)-1*H*-1,2,3-triazol-1-yl)methyl)-phenyl)ethan-1-one (**43**)

The title compound was prepared according to General Procedure VII. Mobile phase: H_2_O/MeOH (30:100%). Yield: 68 mg (72%). ^1^H NMR (401 MHz, DMSO-*d*_6_): δ 8.54 (s, 1H), 8.47 (dt, *J* = 6.9, 1.1 Hz, 1H), 7.73–7.67 (m, 4H), 7.61 (d, *J* = 8.3 Hz, 1H), 7.50 (dd, *J* = 8.3, 2.2 Hz, 1H), 7.45–7.41 (m, 2H), 7.38 (ddd, *J* = 9.0, 6.8, 1.3 Hz, 1H), 7.00 (td, *J* = 6.8, 1.1 Hz, 1H), 5.78 (s, 2H), 2.59 (s, 3H). ^13^C NMR (101 MHz, DMSO): δ 199.81, 144.92, 142.46, 139.01, 136.36, 135.53, 133.01, 132.82, 131.95, 131.18, 129.76, 129.62, 129.14, 128.68, 126.25, 125.89, 125.38, 117.11, 113.32, 111.56, 52.20, 30.64. HRMS: calcd for [M + H], 462.08829; found, 462.08840.

##### 1-(2-Chloro-5-((4-(2-(4-chlorophenyl)imidazo[1,2-*a*]pyridin-3-yl)-1*H*-1,2,3-triazol-1-yl)methyl)phenyl)propan-1-one (**44**)

The title compound was prepared according to General Procedure VII. Mobile phase: H_2_O/MeOH (30:100%). Yield: 51 mg (69%). ^1^H NMR (401 MHz, DMSO-*d*_6_): δ 8.54 (s, 1H), 8.49–8.42 (m, 1H), 7.72–7.67 (m, 3H), 7.65 (d, *J* = 2.1 Hz, 1H), 7.60 (d, *J* = 8.3 Hz, 1H), 7.50–7.46 (m, 1H), 7.44–7.36 (m, 3H), 7.00 (td, *J* = 6.8, 1.1 Hz, 1H), 5.77 (s, 2H), 2.93 (q, *J* = 7.2 Hz, 2H), 1.12–1.02 (m, 3H). ^13^C NMR (101 MHz, DMSO): δ 203.21, 144.92, 142.44, 139.56, 136.35, 135.51, 133.01, 132.82, 131.52, 130.94, 129.60, 129.30, 128.67, 128.46, 126.26, 125.91, 125.38, 117.11, 113.32, 111.56, 52.23, 35.75, 8.03. HRMS: calcd for [M + H], 476.10394; found, 476.10407.

##### 2-Chloro-5-((4-(2-(4-chlorophenyl)imidazo[1,2-*a*]pyridin-3-yl)-1*H*-1,2,3-triazol-1-yl)methyl)benzaldehyde (**45**)

The title compound was prepared according to General Procedure VII with LAH. Crystalized from ACN. Yield: 158 mg (75%). ^1^H NMR (401 MHz, DMSO-*d*_6_): δ 10.34 (s, 1H), 8.56 (s, 1H), 8.47 (dt, *J* = 6.9, 1.2 Hz, 1H), 7.83 (t, *J* = 1.4 Hz, 1H), 7.72–7.67 (m, 5H), 7.45–7.41 (m, 2H), 7.38 (ddd, *J* = 9.1, 6.7, 1.3 Hz, 1H), 6.99 (td, *J* = 6.8, 1.3 Hz, 1H), 5.83 (s, 2H). ^13^C NMR (101 MHz, DMSO): δ 189.72, 144.93, 142.46, 136.39, 136.33, 136.13, 135.17, 133.00, 132.83, 132.36, 131.43, 129.59, 128.79, 128.70, 126.24, 125.95, 125.35, 117.11, 113.33, 111.52, 52.06. HRMS: calcd for [M + H], 448.07264; found, 448.07224.

##### (*E*)-2-Chloro-5-((4-(2-(4-chlorophenyl)imidazo[1,2-*a*]pyridin-3-yl)-1*H*-1,2,3-triazol-1-yl)methyl)benzaldehyde Oxime (**46**)

2-Chloro-5-((4-(2-(4-chlorophenyl)imidazo[1,2-*a*]pyridin-3-yl)-1*H*-1,2,3-triazol-1-yl)methyl)benzaldehyde (**45**) was dissolved in DCM, and NH_2_OH·HCl (1.2 equiv) was added, followed by the addition of TEA (1.2 equiv). The reaction mixture was stirred at 25 °C and washed with water, and the organic phase was dried over sodium sulfate and evaporated. The residue was purified by flash column chromatography. Mobile phase petrol ether/EtOAc (50:100%). Yield: 75 mg (90%). ^1^H NMR (401 MHz, DMSO-*d*_6_): δ 11.75 (s, 1H), 8.51 (s, 1H), 8.46 (dt, *J* = 7.0, 1.2 Hz, 1H), 8.36 (s, 1H), 7.78 (d, *J* = 2.2 Hz, 1H), 7.71–7.66 (m, 4H), 7.56 (d, *J* = 8.3 Hz, 1H), 7.43–7.35 (m, 4H), 7.00 (td, *J* = 6.8, 1.3 Hz, 1H), 5.77 (s, 2H). ^13^C NMR (101 MHz, DMSO): δ 145.22, 144.72, 142.75, 136.69, 136.00, 133.29, 133.13, 132.37, 131.02, 130.78, 130.63, 129.89, 128.97, 126.54, 126.35, 126.16, 125.63, 117.41, 113.67, 111.85, 52.64. HRMS: calcd for [M + H], 463.08354; found, 463.08301.

##### (2-Chloro-5-((4-(2-(4-chlorophenyl)imidazo[1,2-*a*]pyridin-3-yl)-1*H*-1,2,3-triazol-1-yl)methyl)-phenyl)methanol (**47**)

Methyl 2-chloro-5-((4-(2-(4-chlorophenyl)imidazo[1,2-*a*]pyridin-3-yl)-1*H*-1,2,3-triazol-1-yl)-methyl)benzoate (160 mg, 0.334 mmol) was dissolved in dry THF (6 mL), cooled in an ice bath, and degassed. A flask was refilled with argon, and LAH (0.35 mL, 1 equiv) was added. A reaction mixture was allowed to warm to 25 °C and stirred overnight. After the completion of the reaction, the mixture was carefully quenched with ice and extracted with EtOAc. Combined organic phases were dried over sodium sulfate and evaporated. The residue was purified by flash column chromatography (cyclohexane/EtOAc 40:70%). Yield: 85 mg (57%). ^1^H NMR (401 MHz, DMSO-*d*_6_): δ 8.52 (s, 1H), 8.46 (dt, *J* = 6.9, 1.2 Hz, 1H), 7.73–7.67 (m, 4H), 7.51 (d, *J* = 2.3 Hz, 1H), 7.47–7.41 (m, 3H), 7.38 (ddd, *J* = 9.1, 6.7, 1.3 Hz, 1H), 7.30–7.26 (m, 1H), 7.00 (td, *J* = 6.8, 1.2 Hz, 1H), 5.76 (s, 2H), 5.51 (t, *J* = 5.4 Hz, 1H), 4.57 (dt, *J* = 5.4, 0.9 Hz, 2H). ^13^C NMR (101 MHz, DMSO): δ 144.93, 142.41, 140.22, 136.29, 135.16, 133.00, 132.82, 130.72, 129.59, 129.31, 128.71, 127.79, 127.22, 126.24, 125.87, 125.33, 117.11, 113.34, 111.60, 60.22, 52.73. HRMS: calcd for [M + H], 450.08829; found, 450.08835.

##### 3-(1-(4-Chloro-3-(methoxymethyl)benzyl)-1*H*-1,2,3-triazol-4-yl)-2-(4-chlorophenyl)imidazo[1,2-*a*]pyridine (**48**)

(2-Chloro-5-((4-(2-(4-chlorophenyl)imidazo[1,2-*a*]pyridin-3-yl)-1*H*-1,2,3-triazol-1-yl)methyl)phenyl)methanol (85 mg, 0.188 mL) was dissolved in dry THF (4 mL) and NaH (8 mg, 1.1 equiv) was added. After 10 min, CH_3_I (0.013 mL, 1 equiv) was added and the mixture was stirred at 25 °C overnight. The mixture was purified by flash column chromatography (cyclohexane/EtOAc 30:80%). Yield: 35 mg (40%). ^1^H NMR (401 MHz, DMSO-*d*_6_): δ 8.51 (s, 1H), 8.46 (dt, *J* = 7.0, 1.3 Hz, 1H), 7.71–7.65 (m, 3H), 7.51–7.45 (m, 2H), 7.40 (dd, *J* = 8.6, 2.0 Hz, 2H), 7.35 (d, *J* = 1.3 Hz, 0H), 7.32 (dd, *J* = 8.2, 2.3 Hz, 1H), 6.99 (td, *J* = 6.8, 1.3 Hz, 1H), 5.75 (s, 2H), 4.48 (s, 2H), 3.36 (d, *J* = 1.8 Hz, 3H, water overlapping). ^13^C NMR (101 MHz, DMSO): δ 144.92, 142.42, 136.41, 136.33, 135.26, 133.01, 132.81, 131.72, 129.69, 129.59, 128.71, 128.64, 128.46, 126.20, 125.79, 125.30, 117.10, 113.31, 111.60, 70.77, 58.30, 52.58. HRMS: calcd for [M + H], 464.10394; found, 464.10396.

##### 3-(1-(4-Chloro-3-nitrobenzyl)-1*H*-1,2,3-triazol-4-yl)-2-(4-chlorophenyl)imidazo[1,2-*a*]pyridine (**49**)

The title compound was prepared according to General Procedure IV (Scheme 1). Mobile phase petrol ether/EtOAc (40:100%). Yield: 835 mg (83%). ^1^H NMR (401 MHz, DMSO-*d*_6_): δ 8.58 (s, 1H), 8.50 (dt, *J* = 6.9, 1.2 Hz, 1H), 8.15 (d, *J* = 2.1 Hz, 1H), 7.82 (d, *J* = 8.4 Hz, 1H), 7.73–7.65 (m, 4H), 7.43–7.38 (m, 2H), 7.36 (ddd, *J* = 9.1, 6.7, 1.3 Hz, 1H), 6.98 (td, *J* = 6.8, 1.2 Hz, 1H). ^13^C NMR (101 MHz, DMSO): δ 147.65, 144.93, 142.50, 136.94, 136.53, 133.62, 132.99, 132.85, 132.31, 129.63, 128.62, 126.15, 125.94, 125.41, 125.39, 125.10, 117.06, 113.23, 111.50, 51.70. HRMS: calcd for [M – H], 465.06281; found, 465.06250.

##### 2-Chloro-5-((4-(2-(4-chlorophenyl)imidazo[1,2-*a*]pyridin-3-yl)-1*H*-1,2,3-triazol-1-yl)methyl)-aniline (**50**)

3-(1-(4-Chloro-3-nitrobenzyl)-1*H*-1,2,3-triazol-4-yl)-2-(4-chlorophenyl)imidazo[1,2-*a*]pyridine (**49**) was dissolved in MeOH and AcOH (7 equiv), and Fe (3.5 equiv) was added. The reaction mixture was stirred at reflux until the completion of the reaction. After cooling to 25 °C, the mixture was extracted with EtOAc, washed with a NaHCO_3_ solution, and the organic phase was dried over sodium sulfate and evaporated. The residue was purified by flash column chromatography. Mobile phase cyclohexane/EtOAc (40:100%). Yield: 490 mg (79%). ^1^H NMR (401 MHz, DMSO-*d*_6_): δ 8.46 (dt, *J* = 7.0, 1.2 Hz, 1H), 8.44 (s, 1H), 7.73–7.66 (m, 3H), 7.44–7.39 (m, 2H), 7.37 (ddd, *J* = 9.1, 6.7, 1.2 Hz, 1H), 7.21 (d, *J* = 8.2 Hz, 1H), 6.99 (td, *J* = 6.8, 1.2 Hz, 1H), 6.71 (d, *J* = 2.1 Hz, 1H), 6.52–6.47 (m, 1H), 5.59 (s, 2H), 5.50 (s, 2H). ^13^C NMR (101 MHz, DMSO): δ 145.11, 144.88, 142.37, 136.29, 135.75, 132.96, 132.81, 132.80, 129.65, 129.60, 129.49, 128.65, 128.61, 126.19, 126.14, 125.74, 125.33, 117.07, 116.94, 116.02, 114.31, 113.30, 111.65, 52.95. HRMS: calcd for [M + H], 435.08863; found, 435.08815.

##### *N*-(2-Chloro-5-((4-(2-(4-chlorophenyl)imidazo[1,2-*a*]pyridin-3-yl)-1*H*-1,2,3-triazol-1-yl)methyl)-phenyl)acetamide (**51**)

2-Chloro-5-((4-(2-(4-chlorophenyl)imidazo[1,2-*a*]pyridin-3-yl)-1*H*-1,2,3-triazol-1-yl)methyl)-aniline (**50**) was dissolved in dioxane and Ac_2_O (1.5 equiv) was added followed by pyridine (1.5 equiv). The reaction mixture was stirred at 25 °C overnight. After the completion of the reaction, the mixture was evaporated, diluted with EtOAc, and washed with water. The organic phase was dried over sodium sulfate and evaporated. The residue was purified by flash column chromatography. Mobile phase cyclohexane/EtOAc (30:100%). Yield: 75 mg (90%). ^1^H NMR (401 MHz, DMSO-*d*_6_): δ 9.58 (s, 1H), 8.54 (s, 1H), 8.46 (dt, *J* = 6.9, 1.2 Hz, 1H), 7.73–7.67 (m, 4H), 7.54 (d, *J* = 8.3 Hz, 1H), 7.46–7.41 (m, 2H), 7.38 (ddd, *J* = 9.0, 6.7, 1.3 Hz, 1H), 7.17 (dd, *J* = 8.3, 2.2 Hz, 1H), 6.99 (td, *J* = 6.8, 1.2 Hz, 1H), 5.74 (s, 2H), 2.11 (s, 3H). ^13^C NMR (101 MHz, DMSO): δ 169.27, 145.22, 142.68, 136.54, 135.83, 135.80, 133.25, 133.11, 130.25, 129.85, 129.00, 127.90, 126.58, 126.28, 125.78, 125.66, 125.41, 117.35, 114.34, 113.64, 111.84, 52.79, 23.86. HRMS: calcd for [M + H], 477.09919; found, 477.09866.

##### 2-Chloro-5-((4-(2-(4-chlorophenyl)imidazo[1,2-*a*]pyridin-3-yl)-1*H*-1,2,3-triazol-1-yl)methyl)benzoyl Chloride (**52**)

The title compound was prepared according to General Procedure V and used in the next step without further purification.

##### 3-(4-Bromophenyl)-2-(4-chlorophenyl)imidazo[1,2-*a*]pyridine **(53)**

2-(4-Chlorophenyl)-3-iodoimidazo[1,2-*a*]pyridine (300 mg, 0.846 mmol) was combined with (4-bromophenyl)boronic acid (1 equiv) and diluted with dioxane/H_2_O mixture (4:1; 8 mL). Na_2_CO_3_ (3 equiv) was added, and the mixture was degassed and refilled with argon. Pd(dppf)Cl_2_·DCM (5 mol %) was added, and the mixture was degassed again and refilled with argon. The mixture was stirred at 95 °C overnight (Scheme 8). After the completion of the reaction, the mixture was diluted with water and extracted with EtOAc. The organic phases were dried over sodium sulfate and evaporated. The residue was purified by flash column chromatography. Mobile phase: cyclohexane/EtOAc (20:60%). Yield: 193 mg (59%). ^13^C NMR (101 MHz, DMSO): δ 144.73, 140.86, 133.34, 133.20, 133.17, 132.72, 129.61, 129.22, 128.92, 128.75, 126.10, 124.32, 123.04, 117.42, 113.42.

##### 2-(4-Chlorophenyl)-3-(6-chloropyridin-3-yl)imidazo[1,2-*a*]pyridine (**54**)

2-(4-Chlorophenyl)-3-iodoimidazo[1,2-*a*]pyridine (300 mg, 0.846 mmol) was combined with (6-chloropyridin-3-yl)boronic acid (1.3 equiv) and diluted with a dioxane/H_2_O mixture (4:1; 8 mL). Na_2_CO_3_ (3 equiv) was added, and the mixture was degassed and refilled with argon. Pd(dppf)Cl_2_·DCM (5 mol %) was added and the mixture was degassed again and refilled with argon. The mixture was stirred at 95 °C overnight (Scheme 8). After the completion of the reaction, the mixture was diluted with water and extracted with EtOAc. The organic phases were dried over sodium sulfate and evaporated. The residue was purified by flash column chromatography. Mobile phase: cyclohexane/EtOAc (15:60%). Yield: 183 mg (63%). ^1^H NMR (401 MHz, DMSO-*d*_6_): δ 8.53 (dd, *J* = 2.5, 0.8 Hz, 1H), 8.17 (dt, *J* = 6.9, 1.2 Hz, 1H), 8.06 (dd, *J* = 8.2, 2.5 Hz, 1H), 7.74 (dd, *J* = 8.2, 0.8 Hz, 1H), 7.68 (dt, *J* = 9.1, 1.2 Hz, 1H), 7.58–7.52 (m, 2H), 7.42–7.33 (m, 3H), 6.93 (td, *J* = 6.8, 1.2 Hz, 1H). ^13^C NMR (101 MHz, DMSO): δ 151.85, 151.07, 145.19, 142.40, 142.01, 133.04, 132.95, 129.75, 129.07, 126.53, 125.76, 125.37, 124.70, 117.40, 116.95, 113.59.

##### 3-(4-Benzylphenyl)-2-(4-chlorophenyl)imidazo[1,2-*a*]pyridine (**55**)

3-(4-Bromophenyl)-2-(4-chlorophenyl)imidazo[1,2-*a*]pyridine (**53**) was dissolved in anhydrous THF (6 mL) under an argon atmosphere. Pd_2_dba_3_ (5 mol %) was added followed by the addition of XantPhos (15 mol %). To the mixture, a benzylzinc bromide solution 0.5 M in THF (2 equiv) was added and the mixture was stirred at 60 °C overnight (Scheme 8). The mixture was evaporated to a minimal volume, adsorbed onto silica, and purified by flash column chromatography followed by reverse-phase flash column chromatography. Mobile phase: cyclohexane/EtOAc (30:100%) and H_2_O/ACN (30:100%). Yield: 143 mg (72%). ^1^H NMR (401 MHz, DMSO-*d*_6_): δ 7.95 (d, *J* = 6.8 Hz, 1H), 7.63 (d, *J* = 9.0 Hz, 1H), 7.61–7.55 (m, 2H), 7.42 (d, *J* = 8.0 Hz, 2H), 7.37 (d, *J* = 8.2 Hz, 2H), 7.35–7.25 (m, 6H), 7.24–7.17 (m, 1H), 6.83 (td, *J* = 6.8, 1.2 Hz, 1H), 4.04 (s, 2H). ^13^C NMR (101 MHz, DMSO): δ 144.17, 142.47, 140.85, 140.15, 133.33, 132.22, 130.77, 130.13, 129.17, 129.03, 128.71, 128.50, 126.78, 126.30, 125.51, 123.96, 120.97, 117.06, 112.93, 66.52.

##### 3-(6-Benzylpyridin-3-yl)-2-(4-chlorophenyl)imidazo[1,2-*a*]pyridine (**56**)

2-(4-Chlorophenyl)-3-(6-chloropyridin-3-yl)imidazo[1,2-*a*]pyridine (**54**) was dissolved in anhydrous THF (6 mL) under an argon atmosphere. Pd_2_dba_3_ (5 mol %) was added, followed by the addition of XantPhos (15 mol %). To the mixture, a benzylzinc bromide solution 0.5 M in THF (2 equiv) was added and the mixture was stirred at 60 °C overnight (Scheme 8). The mixture was evaporated to a minimal volume, adsorbed onto silica, and purified by flash column chromatography followed by reverse-phase flash column chromatography. Mobile phase: cyclohexane/EtOAc (30:100%) and H_2_O/ACN (30:100%). Yield: 158 mg (75%). ^1^H NMR (401 MHz, DMSO-*d*_6_): δ 8.57 (dd, *J* = 2.3, 0.9 Hz, 1H), 8.06 (dt, *J* = 6.9, 1.2 Hz, 1H), 7.89 (dd, *J* = 8.0, 2.3 Hz, 1H), 7.67 (dt, *J* = 9.1, 1.1 Hz, 1H), 7.57–7.52 (m, 2H), 7.48 (dd, *J* = 8.1, 0.9 Hz, 1H), 7.39–7.30 (m, 7H), 7.23 (ddt, *J* = 8.6, 6.2, 1.8 Hz, 1H), 6.88 (td, *J* = 6.8, 1.2 Hz, 1H), 4.21 (s, 2H). ^13^C NMR (101 MHz, DMSO): δ 161.60, 151.00, 144.97, 141.61, 139.81, 139.36, 133.35, 132.78, 129.65, 129.58, 128.97, 126.78, 126.27, 124.52, 124.11, 123.37, 118.26, 117.41, 113.49, 66.82.

##### 5-(2-(4-Chlorophenyl)imidazo[1,2-*a*]pyridin-3-yl)pyridin-2-amine (**57**)

2-(4-Chlorophenyl)-3-iodoimidazo[1,2-*a*]pyridine (0.2 g, 0.56 mmol) was combined with 2-aminopyridine-5-boronic acid pinacol ester (1.1 equiv) and dissolved in dioxane (8 mL) followed by a solution of Na_2_CO_3_ in 2 mL of H_2_O. A reaction mixture was degassed and refilled with argon. Pd(dppf)Cl_2_ (5 mol %) was added, and the mixture was degassed again and refilled with argon. The reaction mixture was stirred at 90 °C overnight (Scheme 9). After cooling to 25 °C, the mixture was diluted with water and extracted with EtOAc. Combined organic phases were dried over sodium sulfate and evaporated. The residue was purified by flash column chromatography; mobile phase petrol ether/EtOAc (20:70%). Yield: 100 mg (53%). ^1^H NMR (401 MHz, DMSO-*d*_6_): δ 8.00 (dt, *J* = 6.9, 1.2 Hz, 1H), 7.96 (d, *J* = 2.3 Hz, 1H), 7.71–7.65 (m, 2H), 7.62 (dt, *J* = 9.1, 1.2 Hz, 1H), 7.47 (dd, *J* = 8.5, 2.4 Hz, 1H), 7.44–7.37 (m, 2H), 7.30 (ddd, *J* = 9.0, 6.7, 1.3 Hz, 1H), 6.88 (td, *J* = 6.8, 1.2 Hz, 1H), 6.64 (d, *J* = 8.5 Hz, 1H), 6.38 (s, 2H). ^13^C NMR (101 MHz, DMSO): δ 160.23, 149.97, 144.19, 140.26, 139.39, 133.53, 132.09, 128.97, 128.58, 125.43, 124.19, 119.37, 117.01, 112.82, 112.32, 108.79. HRMS: calcd for [M + H], 321.0907; found, 321.0909.

##### 2-(4-Chlorophenyl)-3-(6-methoxypyridin-3-yl)imidazo[1,2-*a*]pyridine (**58**)

2-(4-Chlorophenyl)-3-iodoimidazo[1,2-*a*]pyridine (0.2 g, 0.56 mmol) was combined with (6-methoxypyridin-3-yl)boronic acid (1.1 equiv) and dissolved in dioxane (8 mL), followed by a solution of Na_2_CO_3_ in 2 mL of H_2_O. The reaction mixture was degassed and refilled with argon. Pd(dppf)Cl_2_ (5 mol %) was added, and the mixture was degassed again and refilled with argon. The reaction mixture was stirred at 90 °C overnight. After cooling to 25 °C, the mixture was diluted with water and extracted with EtOAc. Combined organic phases were dried over sodium sulfate and evaporated. The residue was purified by flash column chromatography; mobile phase petrol ether/EtOAc (20:70%). Yield: 100 mg (53%). ^1^H NMR (401 MHz, DMSO-*d*_6_): δ 8.29 (dd, *J* = 2.5, 0.8 Hz, 1H), 8.03 (dt, *J* = 6.9, 1.2 Hz, 1H), 7.85 (dd, *J* = 8.5, 2.4 Hz, 1H), 7.66 (dt, *J* = 9.0, 1.2 Hz, 1H), 7.63–7.57 (m, 2H), 7.42–7.36 (m, 2H), 7.33 (ddd, *J* = 9.0, 6.7, 1.3 Hz, 1H), 7.05 (dd, *J* = 8.6, 0.8 Hz, 1H), 6.90 (td, *J* = 6.8, 1.3 Hz, 1H), 3.95 (s, 3H), ^13^C NMR (101 MHz, DMSO): δ 164.28, 149.39, 144.77, 142.09, 141.25, 133.43, 132.65, 129.46, 128.97, 126.10, 124.57, 118.88, 118.25, 117.33, 113.36, 112.12, 53.94. HRMS: calcd for [M + H], 336.0904; found, 336.0902.

##### 5-(2-(4-Chlorophenyl)imidazo[1,2-*a*]pyridin-3-yl)pyridin-2-ol (**59**)

2-(4-Chlorophenyl)-3-(6-methoxypyridin-3-yl)imidazo[1,2-*a*]pyridine **58** was treated with 4M HCl/dioxane at 95 °C overnight. After completion of the reaction, the solvent was evaporated and the residue neutralized with a saturated NaHCO_3_ solution and extracted with EtOAc. The organic phase was dried over sodium sulfate, evaporated, and purified by RP-flash column chromatography. Mobile phase H_2_O/MeOH 15:90%. Yield: 72 mg (73%). ^1^H NMR (401 MHz, DMSO-*d*_6_): δ 12.04 (s, 1H), 8.12 (dt, *J* = 6.9, 1.2 Hz, 1H), 7.74–7.69 (m, 2H), 7.67–7.59 (m, 2H), 7.48–7.41 (m, 3H), 7.32 (ddd, *J* = 9.0, 6.7, 1.2 Hz, 1H), 6.92 (td, *J* = 6.8, 1.2 Hz, 1H), 6.52 (dd, *J* = 9.4, 0.7 Hz, 1H). ^13^C NMR (101 MHz, DMSO): δ 162.36, 144.57, 143.64, 140.96, 138.82, 133.48, 132.60, 129.35, 129.01, 126.01, 124.89, 121.73, 117.63, 117.22, 113.19, 106.40. HRMS: calcd for [M + H], 322.07417; found, 322.07397.

##### 2-Chloro-5-((5-(2-(4-chlorophenyl)imidazo[1,2-*a*]pyridin-3-yl)pyridin-2-yl)amino)benzamide (**60**)

5-(2-(4-Chlorophenyl)imidazo[1,2-*a*]pyridin-3-yl)pyridin-2-amine was combined with 5-bromo-2-chlorobenzamide (1 equiv) and diluted with dioxane, and the mixture was degassed and refilled with argon. To the solution, Na*t*BuO (1.5 equiv), XantPhos (10 mol %), and Pd_2_dba_3_ (5 mol %) were added and the mixture was stirred at 100 °C overnight. The mixture was filtered through Celite, and the solvent was evaporated to a minimal volume and purified by flash chromatography followed by RP-flash column chromatography. Mobile phase EtOAc, then H_2_O/MeOH (20:100%). Yield: 45 mg (51%). ^1^H NMR (401 MHz, DMSO-*d*_6_): δ 9.67 (s, 1H), 8.30 (dd, *J* = 2.3, 0.8 Hz, 1H), 8.10 (dt, *J* = 6.9, 1.2 Hz, 1H), 7.90–7.82 (m, 3H), 7.71 (dd, *J* = 8.6, 2.4 Hz, 1H), 7.68–7.63 (m, 3H), 7.58–7.55 (m, 1H), 7.43–7.39 (m, 2H), 7.38 (d, *J* = 8.8 Hz, 1H), 7.33 (ddd, *J* = 9.1, 6.7, 1.3 Hz, 1H), 7.04 (dd, *J* = 8.7, 0.8 Hz, 1H), 6.91 (td, *J* = 6.8, 1.2 Hz, 1H). ^13^C NMR (101 MHz, DMSO): δ 168.53, 155.68, 149.29, 144.40, 140.74, 140.26, 139.88, 137.53, 133.32, 132.27, 129.89, 129.16, 128.68, 125.71, 124.33, 120.63, 119.95, 118.64, 117.93, 117.06, 115.77, 113.00, 112.08. HRMS: calcd for [M + H], 474.08829; found, 474.08786.

##### 2-Chloro-5-((5-(2-(4-chlorophenyl)imidazo[1,2-*a*]pyridin-3-yl)pyridin-2-yl)oxy)benzamide (**61**)

5-(2-(4-Chlorophenyl)imidazo[1,2-*a*]pyridin-3-yl)pyridin-2-ol (1.5 equiv) was combined with 5-bromo-2-chlorobenzamide (50 mg, 1 equiv) followed by BPPO (2 mol %), CuI (2 mol %), and K_3_PO_4_ (2 equiv). The mixture was degassed and refilled with argon, and dry DMF (1 mL) was added. The mixture was degassed and refilled with argon and heated up to 110 °C overnight. The mixture was filtrated over Celite, dissolved in MeOH, and purified by RP-flash column chromatography. Mobile phase: H_2_O/CH_3_CN (20:100%). The product was crystallized from THF. Yield: 4 mg (6%). ^1^H NMR (500 MHz, DMSO-*d*_6_): δ 8.36 (d, *J* = 6.9 Hz, 1H), 8.05 (d, *J* = 2.5 Hz, 1H), 7.96 (s, 1H), 7.85–7.80 (m, 2H), 7.71 (s, 1H), 7.67–7.62 (m, 4H), 7.50 (dd, *J* = 9.4, 2.6 Hz, 1H), 7.48–7.43 (m, 2H), 7.33 (ddd, *J* = 8.9, 6.8, 1.2 Hz, 1H), 6.94 (td, *J* = 6.8, 1.2 Hz, 1H), 6.69 (d, *J* = 9.4 Hz, 1H). ^13^C NMR (126 MHz, DMSO): δ 167.32, 160.73, 144.41, 143.38, 141.21, 140.86, 139.01, 137.62, 133.12, 132.42, 130.28, 129.69, 129.48, 129.23, 128.81, 127.32, 125.87, 125.10, 121.99, 116.95, 116.82, 112.87, 107.21. HRMS: calcd for [M + H], 474.06503; found, 474.06512.

### Chemicals for Biological Experiments

Compound **1** (CITCO, (6-(4-chlorophenyl)imidazo [2,1-*b*][1,3]thiazole-5-carbaldehyde-*O*-(3,4-dichloro-benzyl)oxime)), rifampicin (rif), TCPOBOP, and PK11195 were obtained from Sigma-Aldrich (St. Louis, Missouri, United States, now Merck), which is now known as Merck (Darmstadt, Germany). Phenobarbital (Luminal 200 mg/mL injection) was manufactured by Desitin Pharma spol. s.r.o. (Prague, Czech Republic). Ligands for nuclear receptors (GW3965, thyroxin, obeticholic acid, dexamethasone, fenofibrate, GW501516, rosiglitazone, 3-methylcholanthrene, calcitriol, testosterone, and estradiol) were purchased from Sigma-Aldrich (now Merck). The prototype ligands were used at 100 nM (dexamethasone, calcitriol), 1 μM (thyroxin), or 10 μM concentrations.

The compounds were dissolved in DMSO, and the final concentration of DMSO in the entire reaction mixture or cultivation media was 0.1%.

### Cell Culture

Human hepatocellular carcinoma HepG2 and monkey fibroblast-like COS-1 cell lines were cultured as we have described before.^48^ All experiments were performed between passages 5–13 after thawing. CAR Knockout HepaRG and parent HepaRG cells were obtained from Sigma-Aldrich, now Merck (Darmstadt, Germany). The cell lines were cultivated and differentiated in the same manner on the 12-well plates. For each experiment, the HepaRG cells were seeded at a density of 26,600 cells/cm^2^ and kept in William’s medium supplemented with 5 μg/mL insulin, 50 μM hydrocortisone, 10% HyClone fetal serum (GE Healthcare Life Sciences, Pittsburgh, USA). 14 days after seeding, the HepaRG cells were differentiated into hepatocyte-like cells using 1.5% DMSO in culture media for another 14 days.^49^

LS174T, an epithelial Caucasian colon adenocarcinoma cell line, was obtained from (Merck Life Science spol. s r.o., Prague, 87060401-1VL). The line has functional PXR but very low CAR nuclear receptor expression.^43^ The cell line has been cultivated for induction experiments as we described before.^50^

### Primary Human Hepatocytes

The PHHs (human hepatocytes in monolayer-long-term cultures) were obtained from Biopredic (Rennes, France) (batch HEP220965, 45-year-old female, Caucasian; HEP220966, 53-year-old female, Caucasian; HEP220969, 78-year-old female, Caucasian; HEP220971, 46-year-old male, Caucasian, HEP220976, 73-year-old male, African, HEP220980, 84-year-old male, Caucasian). The cells were cultivated according to the manufacturer’s protocol. The PHHs were treated with selected test compounds for 24 or 48 h in the use medium at the concentrations of 1, 5, or 10 μM. Cryopreserved human hepatocytes (HJK) were purchased from BioIVT (Westbury, New York, USA). PHHs were cultured in William’s E medium (ThermoFisher Scientific, Waltham, Massachusetts, USA) supplemented with insulin (10 μg/mL)–transferrin (5.5 μg/mL)–sodium selenite (6.7 ng/mL) (Thermo Fisher Scientific), l-glutamine (2 mM)–penicillin (100 U/mL)–streptomycin (100 μg/mL) (Sigma-Aldrich), 100 nM dexamethasone, and 10% fetal bovine serum (HyClone).

Western blotting experiments have been performed with total cellular or tissue lysates (20 μg) with polyclonal anti-CYP2B6 (PA5-35032, dilution 1:1500), antibeta actin recombinant rabbit monoclonal antibody (MA5-32540, clone JF53-10), and CYP3A4 polyclonal antibody (PA1-343, 1:2000) (all from Thermo Fisher). For protein analysis, PHHs have been treated for 48 h.

### RT-qPCR

RT-qPCR was used to examine CAR target gene expression in PHH, HepaRG cells, or in mouse liver samples. Total RNA, reverse transcription, and qPCR were performed, and mRNA expression data was analyzed as we have described before.^51^

All RT-qPCR experiments were performed in triplicate samples, and data are presented as fold induction to vehicle-treated cells with the same reagents we have described before.^48,52^ PCR TaqMan probes for murine genes have been listed in our previous reports.^52^ TaqMan probes for *CYP3A4*, *CYP2C9*, and *CYP2B6* human genes as well as for reference genes *B2M* and *GADPH* genes were obtained from Thermo Fisher: *CYP3A4* (Hs00604506_m1), *CYP2C9* (Hs02383631_s1), *CYP2B6* (Hs04183483_g1), *GAPDH* (Hs02758991_g1), and *B2M* (Hs00984230_m1).

### Luciferase Assays

A human CAR LBD assembly assay (CAR AA) was performed according to protocols published by Carazo and Pavek with two hybrid expression constructs encoding helices 3–12 (pCAR-C/VP16) and helix 1 (pCAR-N/GAL4) parts of human CAR LBD.^53^ Cells were treated for 24 h with tested compounds (range of concentration from 1 nM up to 30 μM). Data are presented as relative activity (%) to compound **1** (CITCO) at 10 μM. The half-maximal effective concentration (EC_50_) to activate CAR in the assay was calculated from at least six points of dose–response curves using the GraphPad Prism software.

Luciferase gene reporter assays to determine interactions with CAR and its variants or with PXR were performed as we have described before in HepG2 or COS-1 cells.^39,48^ Using these assays, the relative activation of the CAR3 variant in comparison with the activity of compound **1** (CITCO) at 1 μM concentration or the relative value of PXR activation (% of rifampicin-mediated PXR activation at 10 μM concentration) were determined (Tables 2, 4, and 7).

The CYP2B6-luc reporter plasmid (originally entitled B-1.6k/PB/XREM) was kindly donated by Dr. Hongbing Wang (University of Maryland School of Pharmacy, Baltimore, MD, USA) and was used in assays with all human CAR variants as well as with the mouse Car expression vector. Expression vectors (based on pcDNA3.1+/C-(K)-DYK vector) for CAR variant 3 (CAR3, 353 AA, CloneID OHu34914, XM_005245697.4, transcript variant X4, mRNA), CAR variant 2 (CAR2, 352 AA, Clone ID OHu10438, NM_001077480.2), and CAR wild type (wtCAR, 348 AA, Clone ID OHu09315, NM_005122.4, transcript variant 3) were purchased from Genscript (Piscataway, NJ, USA). The mouse Car expression vector pCMV6-mCar (NM_009803) was obtained from OriGene Technologies, Rockville, MD, USA). Empty expression vectors were used in control experiments.

In addition, other expression constructs for a ligand-activated CAR transcription variant 3 (CAR3, pTracer-CMV2-CAR3) was a kind gift from Dr. C. J. Omiecinski (Pennsylvania State University, State College, PA, USA), which was used to validate our results with the commercial construct.

The CYP3A4 promoter luciferase reporter construct (p3A4-luc) and PXR expression vector for transient transfection luciferase assays have been described before.^50^ The p3A4-luc plasmid bears a distal XREM (−7836/-7208) and a basal promoter sequence (prPXRE, −362/+53) from the *CYP3A4* gene promoter region.

Transient transfection experiments with various nuclear receptor-responsive luciferase assays have been performed as we described before with the same protocol and plasmids.^54,55^

### CYP Enzymatic Activity Assays

Human recombinant CYP3A4, CYP2B6, and CYP1A2 enzymes expressed from cDNA using baculovirus-infected insect cells with human CYP450 reductase and cytochrome *b*5 in a microsomal fraction (CYP450-Glo CYP3A4 Assay, CYP450-Glo CYP2B6 Assay, and CYP450-Glo CYP1A2 Assay, Promega, Hercules, CA) were used to evaluate the interaction of compound **39** with these enzymes in vitro according to protocols we published before.^56^

### TR-FRET CAR Coactivator Binding Assay

The LanthaScreen TR-FRET CAR Coactivator Binding Assay Kit, goat (Thermo Fisher Scientific, Catr. No PV4836) with GST-tagged human CAR LBD and a fluorescein-labeled PGC1α coactivator peptide was used with slight modifications of the manufacturer’s protocol as we have reported before.^53^ The half-maximal effective concentration (EC_50_) to activate CAR LBD in the assay was calculated from at least six points (range of 10 pM to 10 μM) from at least two experiments (*n* = 2–3) using the GraphPad Prism software.

### Translocation Assay

Nuclear translocation of pEGFP-hCAR + Ala chimera in COS-1 SV40-transformed African green monkey kidney cells was performed as we have described before with the construct generated in the same report.^48^ The method is a modification of the method originally described by Chen et al.^42^

### Animal Experiments

Humanized PXR-CAR-CYP3A4/3A7 mice (model 11585) were obtained from Taconic (Rensselaer, NY) and kept in a temperature-controlled and light-controlled facility with a 12 h light–dark cycling. All animals had free access to a commercially available laboratory chow diet (Velaz, Prague, Czech Republic). Male 9–14 week-old animals (*n* = 4 per group) were randomized into four groups (control; compound **39** 1 mg/kg; compound **39** 10 mg/kg; compound **1** 10 mg/kg), and these compounds were administered as a single application intraperitoneally in a 5% glycerol formulation in saline. Animals were sacrificed 36 h after the administration, and livers were removed, weighted, and snap-frozen in liquid nitrogen for further total RNA isolation. All animal studies were performed in accordance with the European Directive 86/609/EEC, and they were approved by the Czech Central Commission for Animal Welfare.

### Human and Mouse Plasma Protein Binding, Metabolic Stability in Human or Mouse Liver Microsomes, and Human Liver S9 Fraction

Protocols for the plasma protein binding assay and metabolic stability testing in human and mouse liver microsomes or the S9 fraction are described in the Supporting Information, Chapters 6 and 7.

### Pharmacokinetic Study after Single-Dose Application

PK studies were performed in C57BL/6N male mice after 10 mg/kg application of compound **39** via either i.v. or gavage application (*n* = 4 per group) using HPLC-MS/MS analysis. Blood samples were taken in the following intervals: 10, 120, 240, 480, 720, and 1440 min. Detailed protocols and PK parameters calculation are described in the Supporting Information, Chapter 8.

### Genotoxicity Testing and 7 Day Oral Toxicity Study in Rats

The protocols for the assays are described in the Supporting Information, Chapters 9 and 11.

### hERG Fluorescence Polarization Assay

The hERG fluorescence polarization assay was performed as described in the Supporting Information, Chapter 10.

### In Silico Molecular Dynamics Analysis

#### Molecular Modeling

Receptor and Ligand Preparation: The crystal structure of the hCAR model was retrieved from the RCSB Protein Data Bank (www.rcsb.org) (PDB code: 1XVP).^15^

All ligands for docking were drawn using Maestro (2020.2) and prepared using LigPrep to generate the three-dimensional conformation, adjust the protonation state to physiological pH (7.4), and calculate the partial atomic charges, with the force-field OPLS3e. We employed a standard docking to accommodate the compounds **37**, **39**, **40**, and **48** within the CAR’s LBD (PDB ID: 1XVP; resolution: 2.0 Å, cocrystallized with compound **1**,^15^ amino acid numbering follows the crystal structure), using Glide.^57^ Ligands were docked within a grid around 12 Å from the centroid of the cocrystallized ligand generating 10 poses per ligand. To validate the docking obtained for test ligands, and also to evaluate the capability of the docking algorithm to locate the ligands within the LBD, we redocked the cocrystal ligand (compound **1,** a full agonist) inside the CAR LBD. Next, the seven systems (four test compounds plus compound **1**) were prepared and minimized by adding hydrogens, adjusting the protonation states of amino acids, and fixing missing side-chain atoms and protein loops using Maestro PrepWizard 2020.2. The molecular dynamics simulation protocol and respective analyses can be found in the Supporting Information, Chapter 2. For each ligand, simulations of five 1 μs independent replicas were carried out, resulting in 25 μs worth of simulations for all five systems.

#### Statistical Analysis

Data are presented as the means and SD from at least three independent experiments (*n* = 3). A one-way analysis of variance (ANOVA) with Dunnett’s *post hoc* test was applied. GraphPad Prism ver. 9.3.1. Software (GraphPad Software, Inc., San Diego, CA, United States) was used to perform statistical analysis.

EC_50_ indicates the xenobiotic concentration required to achieve half-maximum activation, and relative *E*_max_ represents the overall maximal calculated activation produced by the tested compound (i.e., maximal efficacy). The activities of compound **1** and rifampicin at 10 μM were set to be 100% in the dose–response calculations. IC_50_ represents the half-maximal inhibitory concentration in the viability MTT assay or in cytochrome P450 inhibition assays. A *p*-value of <0.05 was considered to be statistically significant.

## Supporting Information Available

Cytotoxicity analysis of target compounds; in vitro and in vivo pharmacokinetic details of selected compounds; docking and molecular dynamics supporting data and procedures; additional toxicity studies of compound **39**; supplementary experimental procedures; elemental analyses; and NMR and HPLC data (PDF)
Molecular formula strings (CSV)

## Notes

Results of simulations, in the form of trajectory and interaction data, are available on the Zenodo repository (codes: 10.5281/zenodo.5772317).

## Abbreviations

ACN: acetonitrile
ADME: absorption, distribution, metabolism, excretion
CAR AA: CAR ligand-binding domain assembly assay
CYP2B6: cytochrome P450 family two subfamily B member six
CYP3A4: cytochrome P450 family three subfamily A member four
DCM: dichloromethane
DMF: *N*,*N*-dimethylformamide
EC_50_: concentration required to achieve half-maximum activation
EGFP: enhanced green fluorescent protein
*E*_max_: maximal calculated activation
hERG: human Ether-à-go-go-Related Gene
LBD: ligand-binding domain
LBP: ligand-binding pocket
NIS: *N*-iodosuccinimide
ON: overnight
PHH: primary human hepatocyte
PXR: pregnane X receptor
SRC-1: steroid receptor coactivator one
TEA: triethylamine
THF: tetrahydrofuran
TLC: thin-layer chromatography
TMS: trimethylsilyl
TR-FRET: time-resolved fluorescence energy transfer
