# Supplementary material for: Discovery of Novel Human Constitutive Androstane Receptor Agonists with the Imidazo[1,2-a]pyridine Structure
Source: J Med Chem. 2023 Feb 9;66(4):2422–56. doi: 10.1021/acs.jmedchem.2c01140 (PMC10017030; doi:10.1021/acs.jmedchem.2c01140)
Supplement: Supplementary file 1 — jm2c01140_si_001.pdf [file jm2c01140_si_001.pdf]

Supporting Information

Discovery of novel human constitutive androstane receptor agonists with the imidazo[1,2-a]pyridine structure

Ivana Mejdrová[a,‡], Jan Dušek[b,‡], Kryštof Škach[a], Alžbeta Stefela[b], Josef Skoda[b], Karel Chalupský[ac], Klara Dohnalová[cd], Ivona Pavkova[e], Thales Kronenberger[f,g], Azam Rashidian[f], Lucie Smutná[b], Vojtěch Duchoslav[a], Tomas Smutny[b], Petr Pávek[b], Radim Nencka[a*]*

[a]Institute of Organic Chemistry and Biochemistry, Czech Academy of Sciences, Flemingovo nám. 2, 166 10 Prague 6, Czech Republic; [b]Department of Pharmacology and Toxicology, Faculty of Pharmacy in Hradec Kralove, Charles University, Akademika Heyrovskeho 1203, 500 05, Hradec Kralove, Czech Republic; [c]Czech Centre for Phenogenomics, Institute of Molecular Genetics of the Czech Academy of Sciences, Vídeňská 1083, 142 20, Prague; Czech Republic; [d]1st Medical Faculty, Charles University, Katerinska 32, 112 08, Prague, Czech Republic; [e]Faculty of Military Health Sciences, University of Defense, Trebeska 1575, Hradec Kralove, 500 01, Czech Republic; [f] Department of Internal Medicine VIII, University Hospital of Tübingen, 72076, Tübingen, Germany; [g]School of Pharmacy, Faculty of Health Sciences, University of Eastern Finland, 70211, Kuopio, Finland, and Department of Pharmaceutical and Medicinal Chemistry, Institute of Pharmaceutical Sciences, Eberhard Karls Universität, 72076, Tübingen, Germany.

‡- contributed equally; *-corresponding authors

Content

1. Cell viability/cytotoxicity analysis S3
2. Cytosolic to nuclear translocation of pEGFP-hCAR+Ala chimeric protein S5
3. Stability of compound **37** S6
4. Preliminary docking studies S7
5. Molecular dynamics simulations and trajectory analyses S8
6. Metabolite stability and profiling studies for compound **39** S15
7. Human and mouse plasma protein binding of compounds **39** S20
8. Pharmacokinetic study of compound **39** in C57BL/6N mice S23
9. Repeated Dose 7-day Oral Toxicity Study in Rats S28
10. In Vitro Predictor™ hERG Fluorescence Polarization Assay S29
11. Genotoxicity - Ames test S30
12. Supplementary experimental procedures S32
13. Elemental analyses S36
14. SI NMR spectra S38
15. SI HPLC S98
16. References S103

1. Cell viability/cytotoxicity analysis.

Cell viability/cytotoxicity was determined using a proliferation/viability cell assay (MTS assay, CellTiter 96®AQueousOne Solution Cell Proliferation Assay kit from Promega No. G3582) after treatment with all compounds for 48 h in 96-well cultivation plates (30 x 10^3 cells per well) in human hepatocellular carcinoma HepG2 and African green monkey kidney COS-1 cells.[1] The COS-1 cell line is a highly sensitive cell line to cytotoxic insults. IC_{50} is the concentration required to achieve a 50% decrease in viability in tested cell lines (in μM). IC_{50} has been calculated from at least 5 points using GraphPad software fitting. In addition, relative viability to control samples (100%) has been calculated after treatment with 30 μM for 48 h for all compounds.

Table S-1. Effects of compounds with the phenyl and benzyl rings modification on cell viability in HepG2 and COS-1 cell lines

Comp.	COS-1		HepG2	
	IC_{50} (μM)	% viability at 30 µM	IC_{50} (μM)	% viability at 30 μM
**2**	>30	116±3	>30	96±9
**3**	1.51	12±1	>30	110±10
**12g**	>30	68±4	>30	100±2
**13b**	2.75	45±2	>30	92±19
**13c**	0.57	31±6	>30	73±10
**13d**	2.12	43±14	>30	65±7
**13e**	>30	51±13	14.2	38±5
**13f**	>30	110±3	>30	102±7
**13g**	1.63	13±2	>30	100±5
**13h**[#]	<10	N.D.	N.D.	N.D.
**13i**	>30	67±17	17.5	47±5
**14a**	>30	92±3	>30	99±17
**14b**	>30	91±15	>30	113±8
**14c**	>30	108±9	>30	116±14
**14d**	>30	93±11	>30	106±9
**14e**	>30	60±6	>30	92±6
**14f**	>30	110±21	>30	104±8
**15a**	1.25	36±5	>30	92±4
**15b**	>30	90±4	>30	98±4
**15c**	>30	65±2	>30	79±3
**15d**	>30	71±5	>30	108±10
**15e**	>30	88±16	>30	111±7
**15f**	>30	106±14	>30	111±9
**15g**	>30	102	>30	71
**15h**	25	49±6	>30	150±16
**15i**	>30	87±10	>30	120±24
**15j**	>30	61± 10	>30	126±13
**15k**	>30	104±11	>30	79±33
**15l**	>30	75±9	>30	115±12
**15m**	>30	102±9	>30	81±8

IC_{50} is the concentration required to achieve a 50% decrease in viability in tested cell lines (in μM).

Table S-2. Effects of middle-ring heterocyclic analogs on cell viability in HepG2 and COS-1 cell lines

	COS-1		HepG2	
Comp.	IC_{50} (μM)	% viability at 30 μM	IC_{50} (μM)	% viability at 30 μM
**16A**	>30	97±1	>30	126±21
**16B**	>30	104±11	>30	75±16
**17**	>30	94±2	>30	113±25
**18**	>30	124±9	>30	98±4
**19A**	>30	128±19	>30	80±6
**19B**	>30	137±16	>30	107±5
**20**	>30	87±10	>30	88±10
**21**	>30	111±7	>30	116±30
**22**	>30	52±7	10.1	20±6
**23**	>30	162±21	>30	80±13
**24**	>30	55±12	>30	103±19

IC_{50} is the concentration required to achieve a 50% decrease in viability in tested cell lines (in μM).

Table S-3. Effects of benzyl ring modification analogs of compound 3 on cell viability in HepG2 and COS-1 cell lines

	COS-1		HepG2	
Comp.	IC_{50} (μM)	% viability at 30 μM	IC_{50} (μM)	% viability at 30 μM
**37**	>30	88±19	>30	78±5
**38**	>30	90±3	>30	74±22
**39**	>30	86±7	>30	103±11
**40**	>30	101±5	>30	95±20
**41**	10.6	30±27	>30	81±7
**42**	>30	93±13	>30	91±14
**43**	*15.3*	36±4	*23.4*	42±13
**44**	*8.5*	41±3	>30	64±8
**45**	<1	37±5	>30	73±9
**46**	>30	101±9	>30	76±12
**47**	>30	74±18	*9.4*	30±10
**48**	>30	87±5	>30	119±10
**49**	5	26±8	>30	56±9
**50**	8	27±7	29	48±8
**51**	12	0	15	26±6
**60**	12	20±9	8	35±7
**61**	>30	81±10	>30	130±9

IC_{50} is the concentration required to achieve a 50% decrease in viability in tested cell lines (in μM).

2. Cytosolic to nuclear translocation of pEGFP-hCAR+Ala chimeric protein

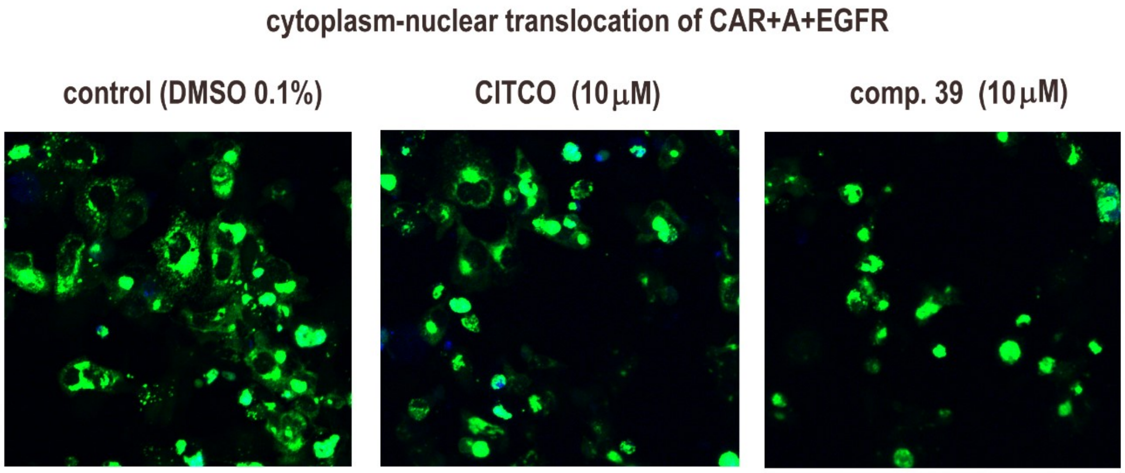

Figure S-1. Translocation experiments with pEGFP-hCAR+Ala chimera in COS-1 cells treated with tested compounds for 24 h before confocal microscopy. Data are presented as % of cells with specific cytoplasm or mixed/nuclear localization of CAR+A+EGFR.

3. **Stability of compound 37**

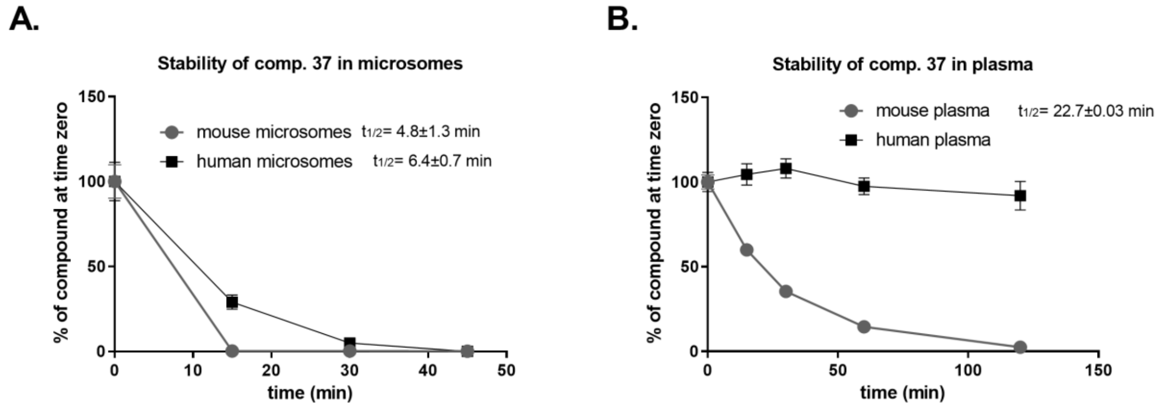

Figure S-2. Stability of compound 37 in mouse and human plasma and pooled microsomes. A. Metabolic stability of compound 37 was assessed at a single concentration (5 µM) at $t = 0$, 10, 30, and at $t = 45$ min. Stability of the compound was tested in human pooled microsomes HMMCPL (ThermoFisher Scientific) and mouse microsomes MSMCPL (ThermoFisher Scientific). Compounds were tested in triplicate with or without NADPH wells as a negative control for P450 metabolism. B. In addition to hepatic metabolism, compounds were also subjected to degradation/modification by enzymes in human (Cat. NO. S4180, VWR International) and mouse (Cat. No. GTX73251, Biomedica, CZ) plasma for $t = 0$, 30, 60, and at $t = 120$ min. Each assay included a substrate with known activity as a positive control. Time points were analyzed by LC/MS/MS. The peak area for the parent compound is compared to the time zero sample in order to assess the amount of remaining compound (displayed as %of initial concentration).

4. Preliminary docking studies

The docking experiments were performed in a similar manner to our previous publications.[2] We used the CAR/RXR heterodimer structure published by Xu and coworkers (PBB ID: 1xvp)[3] as the starting structure for docking. We used Autodock Vina 1.1 docking software[4] with a flexible Met168 residue in the binding site of the protein in the part not occupied by the CITCO ligand (chain B). The center of the grid box was defined as 31.2, 59.6 and 78.1 Å and the grid box size was 24×24×24 Å.

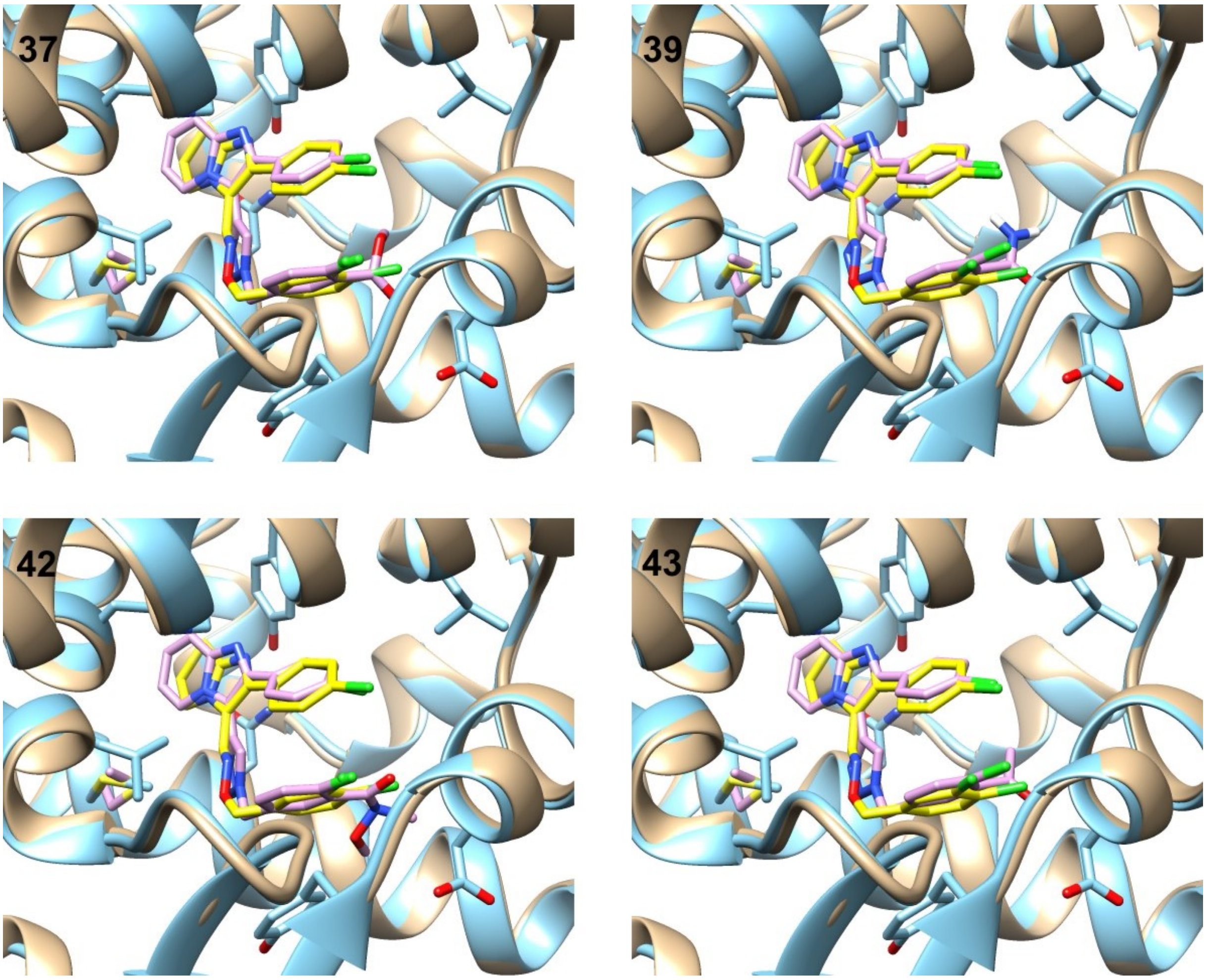

Figure S-3. Examples of preliminary docking experiments we performed for selected compounds. In this case, we selected several compounds from the optimization of the benzyl side chain. The figure shows the overlay of CITCO (yellow) and compounds **37**, **39**, **42** and **43** (light purple), respectively. CITCO is positioned in its original position (PDB ID: 1xvp, chain D, light blue), while the compounds have been docked into the apo structure (PDB ID: 1xvp, chain B, beige). UCSF Chimera was used to prepare this figure.[5]

5. Molecular dynamics simulations and trajectory analyses.

The minimized structures were submitted to molecular dynamics (MD) simulation for further refinement. Selected docking poses were further validated by molecular dynamics simulation, where ligand stability within the proposed pocket and its interactions were evaluated. Molecular Dynamics (MD) simulations were carried out using the Desmond engine [6] with the OPLS3e force-field [7] according to a previously described protocol.[8] The simulated system encompassed the protein-ligand complex, a predefined water model (TIP3P [9]) as a solvent and counterions (Na^+ or Cl^- adjusted to neutralize the overall system charge). The systems were treated in a cubic box with periodic boundary conditions specifying the shape and the size of the box as 13 Å distance from the box edges to any atom of the protein. Short-range coulombic interactions were performed using a time step of 1 fs and a cut-off value of 9.0 Å, whereas long-range coulombic interactions were handled using the Smooth Particle Mesh Ewald (PME) method.[10] Results of simulations, in the form of trajectory and interaction data, are available on the Zenodo repository (codes: 10.5281/zenodo.5772317). MD trajectories were visualized, and figures were produced using PyMOL v.2.4 (Schrödinger LCC, New York, NY, USA) For each ligand, simulations of five 1 μs independent replicas were carried out, resulting in 25 μs worth of simulations for all the five systems.

The RMSD values along the simulations suggest that both CAR/compound **1** (CITCO) and CAR/compound **39** systems are stable (Figure S-4). Although, a minor transition state in CAR/comp. **39** complex during simulation could indicate that this system undergoes conformational changes upon compound **39** binding, this system converges after 5 µs of the simulation. Overall, the simulations converge below 3 Å. Therefore, the systems have been equilibrated, and the simulations are long enough for analysis. In addition, root-mean-square fluctuation (RMSF) analysis revealed that the highest fluctuations are related to the H2'-H3 loop (residues 140-153; Figure S-5; Table S-4;) and this phenomenon is further considerable with compound **39**. These amino acids are mainly contributing to the LBD motions.

Protein-ligand interactions were determined using the Simulation Event Analysis pipeline implemented in Maestro (Maestro v2020.2). The current geometric criteria for protein-ligand hydrogen bond is a distance of 2.5 Å between the donor and acceptor atoms (D — H···A); a donor angle of ≥120° between the donor-hydrogen-acceptor atoms (D — H···A); and an acceptor angle of ≥90° between the hydrogen-acceptor-bonded atom atoms (H···A — X).

Similarly, the protein-water or water-ligand hydrogen bond had a distance of 2.8 Å between the donor and acceptor atoms (D—H···A); a donor angle of ≥110° between the donor-hydrogen-acceptor atoms (D—H···A); and an acceptor angle of ≥90° between the hydrogen-acceptor-bonded atom atoms (H···A—X). Non-specific hydrophobic interactions are defined by hydrophobic sidechain within 3.6 Å of a ligand's aromatic or aliphatic carbons and π-π interactions required two aromatic groups stacked face-to-face or face-to-edge, within 4.5 Å of distance.

Distance calculations were performed using Maestro event analysis tool (Schrödinger, LLC, New York, NY). Distances between specific secondary structure elements were calculated using their centers of mass, using the script *trj_asl_distance.py* having as an argument the atom numbers of the residues involved in the interaction.

Structure and data visualization. Structure visualization was conducted with PyMOL v.2.5 (Schrödinger LLC, New York, NY, USA). Data visualization was completed by Python 3.7, seaborn [10], matplotlib [11] and GraphPad prism (v. 9.3 for Windows, GraphPad Software, San Diego, CA, USA).

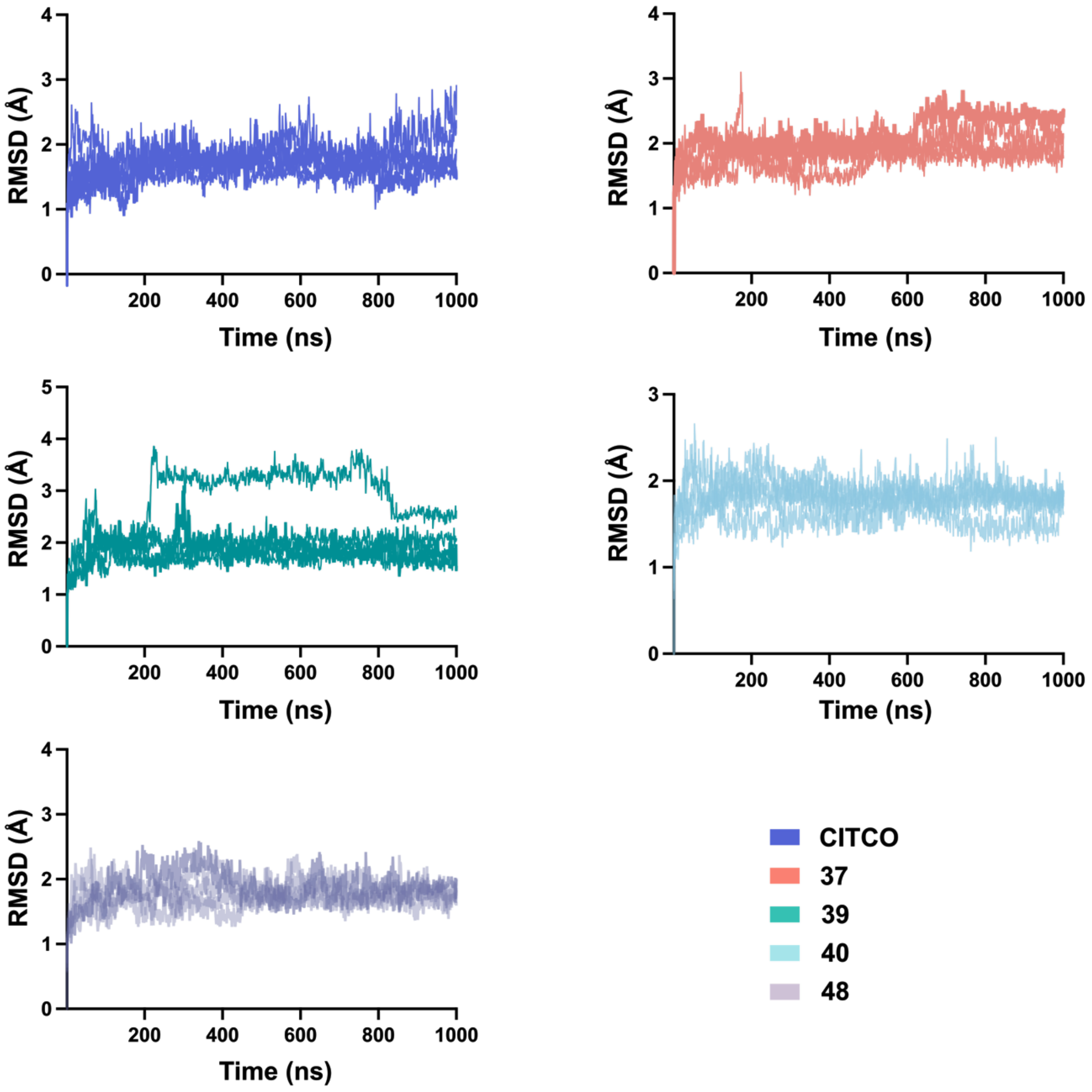

Figure S-4. Root-mean-square Deviation (RMSD) of protein backbone along the simulation time (in nanosecond).

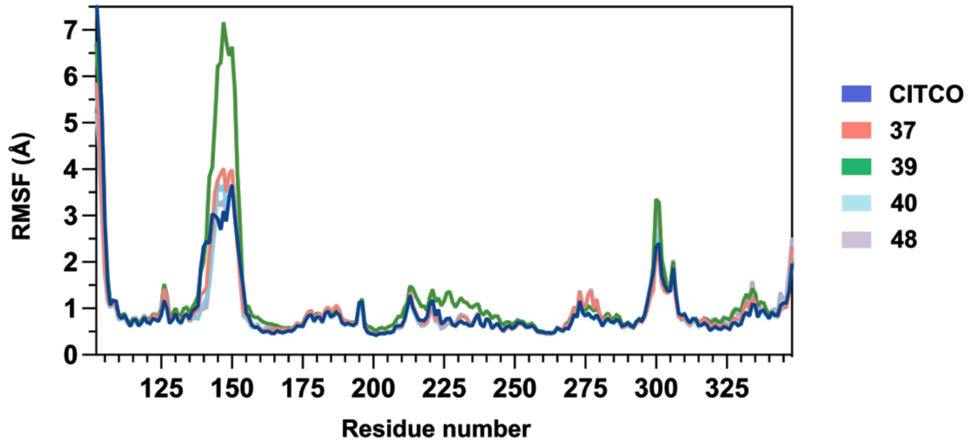

Figure S-5. Root-mean-square fluctuation (RMSF) of the protein backbone.

Table S-4. Root-mean-square fluctuations (RMSFs) of individual residues (backbone atoms) of H2'-H3 loop. Colours are according to the respective ligand

H2'–H3 loop					
Residue Number	**CITCO (1)**	**37**	**39**	**40**	**48**
140	2.32	1.34	2.00	1.10	0.98
141	2.45	1.45	2.55	1.24	1.00
142	2.41	2.20	3.82	1.50	1.55
143	3.04	2.68	4.04	2.20	2.30
144	3.01	3.49	5.00	2.72	3.14
145	2.93	3.75	6.21	3.71	3.31
146	2.70	3.89	6.29	3.50	3.20
147	3.10	4.01	7.16	3.66	3.34
148	2.88	3.50	6.76	3.16	3.02
149	3.39	3.88	6.44	3.48	3.52
150	3.67	3.99	6.63	3.66	3.63
151	2.98	3.15	5.68	3.39	3.35
152	2.46	2.34	3.87	2.45	2.51
153	1.91	1.60	2.65	1.63	1.55

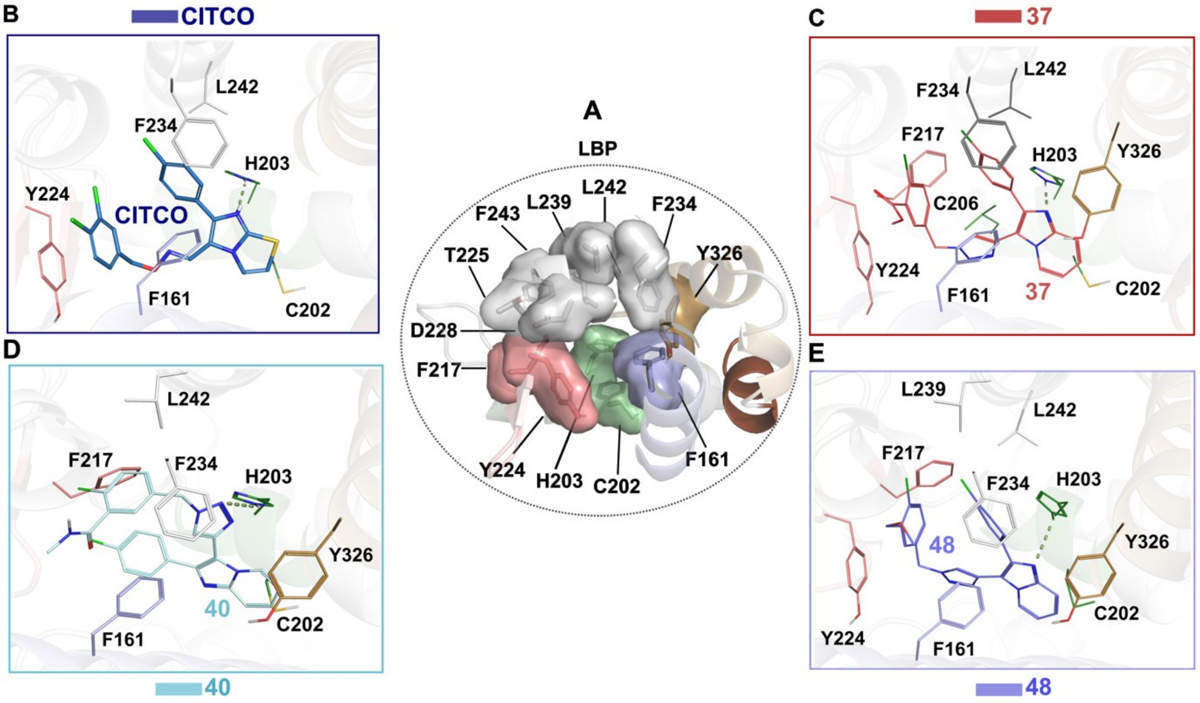

Figure S-6. The representative snapshots of the CAR1's LBP with compound **1** (CITCO) (PDB ID: 1XVP) and the simulated novel compounds. (A) wtCAR-LBP, (B) wtCAR's LBP with compound **1** (CITCO), (C) wtCAR-LBP with compound **37**, (D) wtCAR's LBP with compound **40** and (E) wtCAR's LBP with compound **48.**

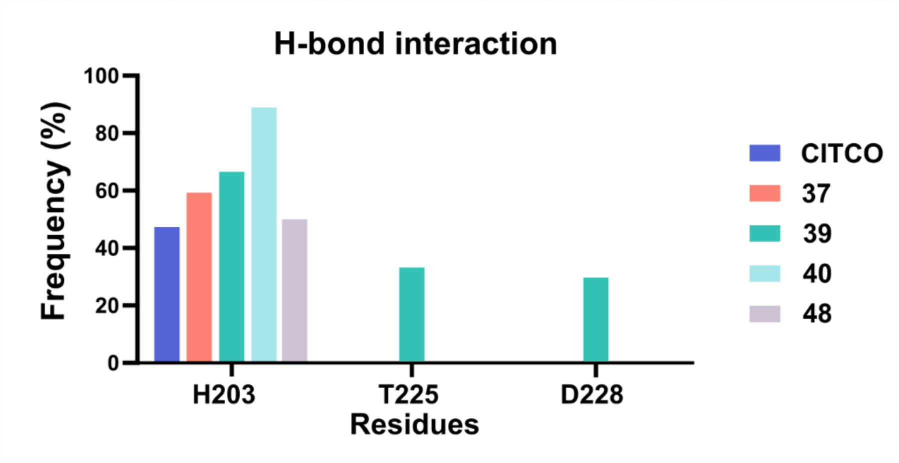

Figure S-7. Protein-Ligand hydrogen bond interaction between wtCAR and the simulated compounds

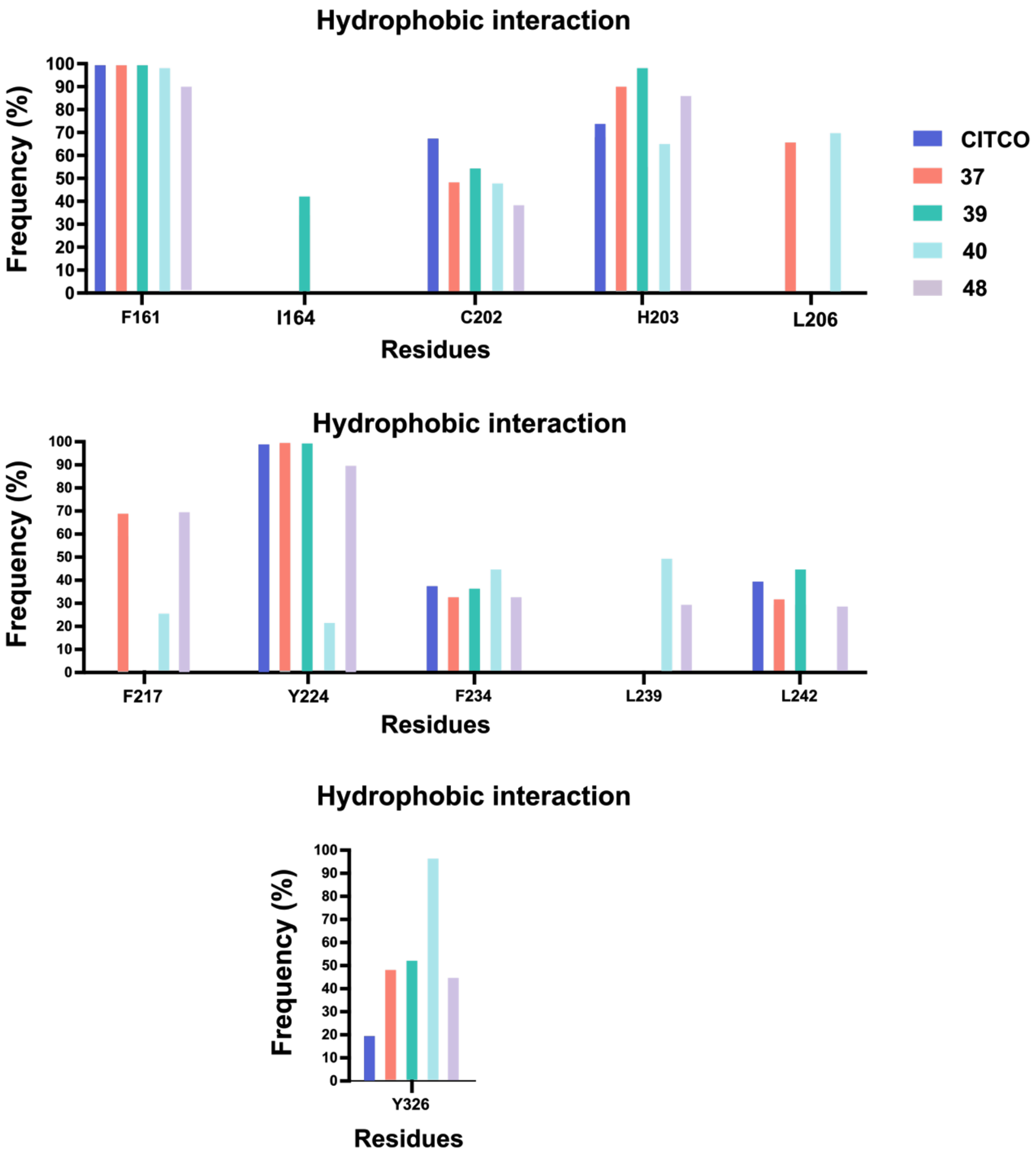

Figure S-8. Protein-Ligand hydrophobic interaction between wtCAR and the simulated ligands.

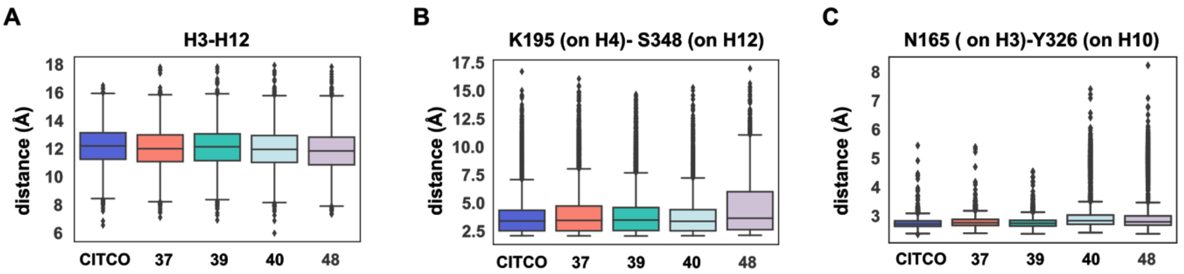

Figure S-9. Compounds **37**, **39**, **40** and **48** can stabilize the conformation of the H12 in the close vicinity of H3. The graph (A) illustrates the distance between H3-H12. Graph shows the distance between K195 (on H4) and S348 (on H12). Graph (C) displays the distance between N165 (on H3) and Y326 (on H10).

6. Metabolite stability and profiling studies for compound 39 in human and mouse hepatic microsomes or S9 fraction.

6.1 Reagents and consumables.

DMSO Chromasolv Plus, HPLC grade, ≥99.7% (Sigma-Aldrich, USA; Lot #34869)
Acetonitrile Chromasolv, gradient grade, for HPLC, ≥99.9% (Sigma-Aldrich, USA; Lot #34851)
Formic acid for mass-spectrometry, ~98% (Fluka, USA; Lot #94318)
DMSO stock solution of the tested compound at 10 mM
Potassium phosphate monobasic (Helicon, Am-O781-0.5)
Potassium phosphate dibasic (Helicon, Am-O705-0.5)
Magnesium chloride hexahydrate (Helicon, Am-O288-0.1)
Glucose-6-phosphate dehydrogenase from baker's yeast, type XV (Sigma-Aldrich, G6378)
Glucose-6-phosphate sodium salt (Sigma-Aldrich, G7879)
β-Nicotinamide adeninedinucleotide-2'-phosphate reduced, tetrasodium salt (Santa Cruz Biotechnology, Inc., sc-202725A)
Uridine 5′-diphosphoglucuronic acid trisodium salt (UDPGA) (Sigma-Aldrich, U6751)
Adenosine 3′-phosphate 5′-phosphosulfate lithium salt hydrate (PAPS) (Sigma-Aldrich, A1651)
Glutathione reduced, GSH (Bio Basic Canada INC., Cat # GB0229)
7-Hydroxycoumarin (Enamine stock, Cat # EN300-18075)
Midazolam (Shire, Belgium)(a prototype drug with fast clearance)
Caffeine hydrochloride (Sigma-Aldrich, Cat. P0884)
Imipramine hydrochloride (Sigma-Aldrich I7379) (a prototype drug with fast clearance)
Propranolol hydrochloride (Sigma-Aldrich, P0884)

6.2. Equipment and Analytical System.

Gradient HPLC system VP (Shimadzu, Japan)
MS/MS detector API 3000 PE with Turbo Ion Spray Electrospray module (AB Sciex, Canada)
VWR Membrane Nitrogen Generators N2-04-L1466, nitrogen purity 99%+ (VWR, USA)
Innova 4080 Incubator Shaker (New Brunswick Scientific, USA)
Centrifuge 4-15C Qiagen (Sigma, Germany) with Sigma 4-15 Qiagen Rotor Nr.09100
Water purification system NANO pure Diamond D11911 (Thermo Scientific Barnstead, USA)
Solid phase extraction 96-well plate (VARIAN SPEC C18 AR, 15 mg of sorbent)

Shimadzu HPLC system comprised of 2 isocratic pumps LC-10ADvp, an autosampler SIL-HTc, a sub-controller FCV-14AH and a degasser DGU-14A. Mass spectrometric analysis was performed using an API 3000 (triple-quadrupole) instrument from AB Sciex (Canada) with an electro-spray (ESI) interface. The data acquisition and system control were performed using Analyst 1.5.2 software from AB Sciex.

6.3. Stability and Metabolite profiling in S9 human hepatocyte fraction.

Metabolite profiling of compound **39** was performed in human liver S9 fraction (XenoTech, H0620.S9/Lot1210091, Sekisui XenoTech, Kansas City, KS, USA). The reference compound midazolam, which is metabolized both by phase I and phase II enzymes, and 7-hydroxycoumarin, which is predominantly metabolised by glucuronidation and sulphation (and not by phase I enzymes), were used as the S9 fraction reference control. The incubations were carried out in 1.1 mL microtubes (in 96-well format plate) in aliquots of 40 μL each (2 for each time point, 0, 5, 11, 30, and 60 min). The S9 incubation medium contained phosphate buffer (100 mM, pH 7.4), $MgCl_2$ (3.3 mM), NADPH (3 mM), glucose-6-phosphate (5.3 mM), glucose-6-phosphate dehydrogenase (0.67 units/ml), UDPGA (2.5 mM), PAPS (0.3 mM), reduced glutathione (GSH, 2mM) with 2.5 mg of S9 protein per 1 mL. Control incubations were performed replacing the cofactors system with phosphate buffer. Additionally, control incubations containing S9 and cofactors but without test compound were performed in order to exclude false positives from endogenous matrix. Test compound **39** (1 μM, final solvent concentration of 1.6%) was incubated with S9 at 37°C, shaking at 100 rpm. Incubations were performed in duplicates. Five time points over 60 minutes (0, 5, 11, 30, and 60 min) had been analyzed. The reactions were stopped by adding 4 volumes of 90% acetonitrile-water to incubation aliquots, followed by protein sedimentation by centrifuging at 5500 rpm for 3 minutes.

The concentrated sample was obtained by solid-phase extraction of test compound and metabolites on SPEC C18 AR sorbent. The incubation with S9 was performed at larger scale (600 μL) and allowed to pass through SPEC C18 AR sorbent. The compounds absorbed on sorbent were washed with 500 µl of water and eluted with 200 µl of acetonitrile (2 x 100 μL) to give the concentrated sample. Both the supernatants (protein sedimentation with acetonitrile) and concentrated samples (three time points, 0, 30 and 60 min) were analyzed using the HPLC system coupled with tandem mass spectrometer.

Metabolic stability of compound **39** in human liver S9 fraction was defined as the percentage of parent compound lost over time in the presence of a metabolically active test system. Along with the parent compound, proposed metabolites - compound **40**, compound **41** and metabolite M3 (2-(4-chlorophenyl)-3-(1H-1,2,3-triazol-4-yl)imidazo[1,2-a]pyridine) were monitored using sensitive and specific MRM method.

6.4. HPLC-MS/MS Conditions.

Chromatographic Conditions:
Column: Symmetry C18 (100 x 2.1 mm, 3.5μm)
Mobile phase A: Acetonitrile: Water: Formic acid = 50: 950: 1
Mobile phase B: Acetonitrile: Formic acid = 100: 0.1
Linear gradient A: 1.2 min 100% B, 1.21 min 0% B, 2.4 min stop.
Linear gradient B: 2 min 0% B, 11.5 min 30% B, 12.5 min 100%, 14 min 100% B; 14.01 min 0% B, 17min stop. A divert valve directed the flow to the detector from 1 to 15 min.
Elution rate: 400 μL/min.
Column temperature: 30°C.
MS/MS Detection:
Scan types: Positive and Negative Q1, 250-520 Da, 500-900 Da (MS), product ion (MS2), MRM.
Ion source: Turbo spray, Ionization mode: ESI
Nebulizer gas: 15 L/min, Turbo Ion Spray Gas: 8 L/min, Curtain gas: 8 L/min,
Collision gas: 4 L/min
Temperature: 400 °

6.5. Metabolite stability and profiling studies for compound 39 in human or mouse hepatic microsomes.

Microsomal incubations were carried out in 96-well plates in 5 aliquots of 40 μL each (one for each time point). Liver microsomal incubation medium contained PBS (100 mM, pH 7.4), $MgCl_2$ (3.3 mM), NADPH (3 mM), glucose-6-phosphate (5.3 mM), glucose-6-phosphate dehydrogenase (0.67 units/mL) with 0.42 mg of liver microsomal protein per 1 mL. Liver microsomes pooled from human adult males and females have been obtained from XenoTech (H0630/lotN1210097, Sekisui XenoTech, Kansas City, KS, USA). Mouse hepatic microsomes were isolated from pooled (50), perfused livers of BALB/c male mice according to the standard protocol. [12] Control incubations were performed replacing the NADPH-cofactor system with

PBS. Test compound (2 μM, final solvent concentration 1.6 %) was incubated with microsomes at 37 °C with shaking at 100 rpm. Incubations were performed in duplicates. Five time points over 40 minutes had been analyzed. The reactions were stopped by adding 12 volumes of 90% acetonitrile-water to incubation aliquots, followed by protein sedimentation by centrifuging at 5500 rpm for 3 minutes. Supernatants were analyzed using the HPLC system coupled with tandem mass spectrometer.

The elimination constant (k_{el}), half-life ($t_{1/2}$) and intrinsic clearance (Cl_{int}) were determined from ln(concentration versus time) plot using linear regression analysis and the following formulas: $t_{1/2}$=0.693/k_{el}, Cl_{int}= 0.693/$t_{1/2}$ ×μL (incubation)/mg (microsomes)

6.6. Results of stability and metabolite profiling.

Compound **39** exhibited moderate stability in human liver microsomes and S9 fraction. Elimination in mouse microsomes is faster than in human liver preparations (Table S-5).

For the search of compound **39** metabolites, mass spectrometer was operated both in positive and negative ion Q1 scan modes (positive and negative parent ions were scanned in the ranges of 250-520, 500-900 Da). A control (quenched at time 0 min) and the incubated samples (30 and 60 min) were analyzed and compared. Additionally, control incubations containing S9 and cofactors but without test compound (0, 30, and 60 min) were analyzed and compared with the test compound incubation samples in order to exclude false positive peaks coming from endogenous matrix. The algorithm of analysis included both stepwise examination of Q1 scans data (20 Da step) and directed search of predicted metabolites. The qualitative (Q1 scans) and quantitative (MRM scans) analysis revealed no formation of suggested metabolites compounds **40**, **41** and M3. One major metabolite M6 with m/z 497.2 (M+1) was observed after incubation with human S9 liver fraction. The mass difference of 34 Da of metabolite M6 from parent compound relates to metabolic biotransformations that include epoxidation of piridin ring followed by hydration. This hypothesis should be confirmed by detailed analysis of MS/MS spectra and high resolution mass spectrometry.

We also analyzed stability of compound **41** in human and mouse microsomes. The compound appeared as less stable in comparion with compound **39** (Table S-5).

Table S-5. Metabolite stability and profiling studies for compound 39 in human and mouse hepatic microsomes or human S9 fraction

Compound	Human microsomes		Human S9 fraction		Mouse microsomes	
	$t_{1/2}$ (min)	Cl_{int} (μL/min/mg)	$t_{1/2}$ (min)	Cl_{int} (μL/min/mg)	$t_{1/2}$ (min)	Cl_{int} (μL/min/mg)
Caffeine	697.45	2.39	-	-	-	-
Propranolol			-	-	41.81	39.95
Imipramine	0.010	69.96	-	-	8.98	185.94
Midazolam	-	-	6.0	58.1	-	-
Compound **39**	38.04	43.90	42.4	8.2	22.01	75.89
Compound **41**	14.22	117.50	-	-	6.81	245.30

7. Human and mouse plasma protein binding of compounds 39.

Human plasma protein binding for compound **39** has been analyzed using micro-equilibrium dialysis and HPLC-MS/MS. The human plasma has been spiked with the test compounds at concentration of 1 μM and dialyzed against buffer until equilibrium is achieved. Concentrations of the compound in both plasma (human plasma pooled with trisodium citrate as anticoagulant) and buffer were determined to calculate the percentage of plasma protein bound compound. 96-well dialysis membrane HTD96b dialyzer complete unit (HTDialysis LLC, Gales Ferry, CT USA; Cat. # 1006) was used. Each individual well unit consisted of 2 chambers separated by a vertically aligned dialysis membrane of predetermined pore size (MWCO 12-14 kDa)(Cat. # 1101). 120 μL of non-diluted plasma spiked with the tested compound (1 μM, final DMSO concentration 1%) was added to one chamber and the same volume of PBS buffer pH 7.4 (pH 7.4, Sigma-Aldrich, now Merck; Cat #P5368) to the other chamber. HTD96b dialyzer was covered with adhesive sealing film (HTDialysis LLC, USA; Cat #1102) and incubated at 37 °C with shaking at 100 rpm for 5 hours. For samples preparation, an aliquot of the content of each chamber had been mixed with the same volume of the blank opposite matrix. In order to define non-specific loss of tested compounds, standard solution was created by mixing an aliquot of spiked plasma with blank buffer without dialysis. Samples were diluted 10-fold with 100% acetonitrile with subsequent plasma proteins sedimentation by centrifuging at 6,000 rpm for 5 minutes. Human plasma was pooled with Na-EDTA as anticoagulant. Supernatants were analyzed using HPLC system coupled with tandem mass spectrometer (see chapter 5). The percentage of plasma protein bound compound and recovery were calculated using following equations:

$$protein\_binding = \left(1 - \frac{peak\_area\_in\_buffer}{peak\_area\_in\_plasma}\right).100\%$$

$$Recovery = \left(\frac{peak\_area\_in\_buffer \ + pear\_area\_in\_plasma}{peak\_area\_in\_standard\_solution}\right).100\%$$

For mouse plasma protein binding analyses, mouse plasma Na-EDTA (Lampire Bioloical Labs, USA, Cat. 7304309) was used. Data are presented as means from two independent experiments.

We found that compound **39** is highly bound both to human and mouse plasma protein (Table S-6).

Table S-6. Human and mouse plasma protein binding

Compound	Human		Mouse	
	% of bound compound, mean	Recovery, %	% of bound compound, mean	Recovery, %
Verapamil	89.4	94±11	89.4	94±11
Compound **39**	98.0	100±0.8	99.3	99.4±2

7.1. Stability of compounds 39 in human or mouse plasma.

Incubation of compound **39** with the Polled Human serum (from PAA Laboratoria GmbH, Cölbe, Germany, Lot #C02010-0443) or with Sterile filtered mouse serum (sm-0500, Equitech-Bio, Inc. Kerrville, TX, USA) was carried out in 5 aliquots of 70 μL each (one for each time point), in duplicates. Test compounds (1 μM, final DMSO concentration 1%) were incubated at 37 °C with shaking at 100 rpm. Five time points over 120 minutes have been analyzed. The reactions were stopped by adding 420 μL of acetonitrile-water mixture (90:10) with subsequent plasma proteins sedimentation by centrifuging at 5,500 rpm for 5 minutes. Supernatants were analyzed by the HPLC system coupled with tandem mass spectrometer. The percentage of the test compounds remaining after incubation in plasma and their half-lives ($t_{1/2}$) were calculated. For the HPLC-MS/MS analysis, Shimadzu VP HPLC (Shimadzu, Japan) equipped with the tandem MS/MS detector API 3000 PE with TurboIonSpray Electrospray module (PE Sciex, USA) were used (see above). Propantheline bromide ≥97% (TLC)(a prototype unstable compound), powder (Sigma-Aldrich, USA; P8891) and Verapamil hydrochloride (Sigma Aldrich, USA; Cat #V4629, a prototype stable compound) were used as control reference compounds.

Stability study in mouse plasma with compound **39** has been performed with sterile filtered mouse serum (sm-0500, Equitech-Bio. Inc.) according to the same protocol as for stability study in human plasma.

Compound **39** showed high stability both in human and mouse plasma (Table S-7).

Table S-7. Stability of compound 39 in human and mouse plasma

Compound	Human plasma ($t_{1/2}$, min)	Mouse plasma ($t_{1/2}$, min)
Propantheline	16.2	13.8
Verapamil	>240	>240
Compound **39**	>240 (494.4)	>240

8. Pharmacokinetic study of compound 39 in C57BL/6N mice following intravenous and peroral administration.

The purpose of this pharmacokinetic study was to determine the pharmacokinetic characteristics of compound **39** HCl salt in C57BL/6N mice following peroral (*p.o.*gavage) and intravenous (*i.v.*) administrations. Levels of the parent compound **39** and potential metabolites (compounds **40, 41** and M3) were determined by LC-MS/MS in the blood plasma over time after a single dose application.

Study design, animal selection, handling and treatment were all in accordance with the Enamine PK study protocols and Institutional Animal Care and Use Guidelines. Animal treatment and plasma samples preparation were conducted by the Animal Laboratory personnel at Enamine/Bienta (Kiev). Male C57BL/6N mice (7-8 weeks old, body weight 18.9 to 23.5 g and average body weight across all groups 20.8 g, SD = 1.1 g) were used in this study. The animals were randomly assigned to the treatment groups before the pharmacokinetic study and all animals were fasted for 4 h before dosing.

The compound **39** HCl salt was used in 5% glycerol suspension in saline and dosed 10 mg/kg b.w. after gavage or *i.v.* application in application volume of 0.1 mL/100 g b.w.

Six time points (10, 120, 240, 480, 720, and 1440 min) were set for this pharmacokinetic study. Each of the time point treatment group included 4 animals. There was also a control group of 2 animals. Mice were injected *i.v.* with 2,2,2-tribromoethanol at the dose of 150 mg/kg prior to drawing the blood. For gavage application, the stainless-steel animal feeding tubes, 20ga x 38mm (Instech, USA, Cat# FTSS-20S-38) was used. Blood collection was performed from the orbital sinus in microtainers containing K_2EDTA (BD Microtainer® Blood Collection Tubes). Animals were sacrificed by cervical dislocation after the blood samples collection. All samples were immediately processed, flash-frozen and stored at -70°C until subsequent analysis.

In parallel to the pharmacokinetic study performed at Bienta/ENAMINE (Kiev), pharmacokinetics of compound **39** have been performed by out team with the same results at the Institute of Organic Chemistry and Biochemisty, Academy of Sciences, (*data not presented*).

8.1. Samples analysis.

The concentration of compound **39** and its supposed metabolites in plasma was determined using high performance liquid chromatography/tandem mass spectrometry (HPLC-MS/MS). Shimadzu HPLC system comprised 2 isocratic pumps LC-10ADvp, an autosampler SIL-20AC, a sub-controller FCV-14AH and a degasser DGU-14A. Mass spectrometric analysis was

performed using an API 3000 (triple-quadrupole) instrument from AB Sciex (Canada) with an electro-spray (ESI) interface. The data acquisition and system control was performed using Analyst 1.5.2 software from AB Sciex (see *Supplementary Information, Chapter 4*).

8.2. Samples processing.

Plasma samples (50 μL) were mixed with 200 µl of IS solution. After mixing by pipetting and centrifuging for 4 min at 6,000 rpm, 2 μL of each supernatant was injected into LC-MS/MS system. Solution of compound IS-19851 (200 ng/ml in acetonitrile-methanol mixture, 1:1, v/v) was used as internal standard (IS) for quantification of compound **39** in plasma samples.

8.3. HPLC-MS/MS Conditions.

Chromatographic Conditions:
Column: Discovery C18 (50 x 2.1 mm, 5µm)
Mobile phase A: Acetonitrile: Water : Formic acid = 50 : 950 : 1
Mobile phase B: Acetonitrile: Formic acid = 100 : 0.1
Linear gradient: 0 min 12% B, 1.0 min 100% B, 1.01 min 12% B, 2.2 min stop
Elution rate: 400 µL/min. A divert valve directed the flow to the detector from 1.4 to 1.7 min
Column temperature: 30°C

8.4. MS/MS Detection.

Scan type: Positive MRM, Ion source: Turbo spray, Ionization mode: ESI
Nebulize gas: 15 L/min, Curtain gas: 8 L/min, Collision gas: 4 L/min
Ionspray voltage: 5000 V, Temperature: 400°C

Table S-8. MS parameters for compound 39 and its putative metabolites

Compound	Parent, m/z	Daughter, m/z	Time, ms	DP, V	FP, V	EP, V	CE, V	CXP, V
Compound **39**	463.01	267.2	40	56	250	11	35	18
Compound **41**	491.052	267.2	15	61	300	11	39	18
Compound **40**	477.022	267.2	15	56	270	11	37	18
Metabolite M3	296.115	268.0	15	46	220	11	25	18
IS-19851	390.063	354.1	40	71	370	11	51	18

8.5. Preparation of calibration standards.

The compound **39** (HCl salt) was dissolved in DMSO at concentration 5 mg/mL and was used to prepare the calibration standards (stock solution). Stock solutions of compounds **41**, **40** and metabolite M3 were prepared by dissolving the compounds in DMSO to yield the final concentration of 10 mM. Aliquots of stock solutions of compounds **39**, **40**, **41** and metabolite M3 were mixed to yield the working mix solution with final concentration of 1 mg/mL. A series of calibration standards was prepared by sequential dilution of the working mix solution with blank mouse plasma to a final concentration of 40000, 20000, 10000, 5000, 2500, 1000, 500, 250, 100, 50, 20 10 and 5 ng/mL. Standard plasma samples (50 µl) were mixed with 200 μL of internal standard compound (IS, compound IS19851). After mixing by pipetting and centrifugation for 4 min at 6,000 rpm, 2 μL of each supernatant was injected into LC-MS/MS system. Validation results are available from authors after request. R were higher than 0.9996 for all compounds.

8.6. Pharmacokinetic Method Analysis.

The concentrations of the parent compound and potential metabolites below the lower limits of quantitation (LLOQ = 5 ng/mL) were designated as zero. The pharmacokinetic data analysis and pharmacokinetic parameters were obtained using noncompartmental, bolus injection or extravascular input analysis models in WinNonlin 5.2 (PharSight Co., Mouitin View, CA, USA) or S.A.A.M II (Epsilon, Charlottesville, VA, USA). The bioavailability for the peroral route was calculated as:

$$F\,(\%) = Dose_{IV} \times AUC_{(0-\infty)PO}\; Dose_{PO} \times AUC_{(0-\infty)IV} \times 100\%$$

8.7. Pharmacokinetic results.

Pharmacokinetics parameters for compound **39** were determined using pharmacokinetic analysis and are summarized in Table S-9.

Proposed metabolites **40**, **41** or M3 were found only after *i.v.* application with concentration below 20 ng/mL (Figure 10C).

These data suggest fat absorption of compound **39** and fast disappearance from the central compartment. This is likely due to extensive distribution into the whole body as suggests the large Vd parameter exceeding murine blood volume (Table S-9). Further studies should reveal whether compound **39** is eliminated into urine or bile or distributed into fat or muscle tissues.

Table S-9. Pharmacokinetic parameters of compound 39 as hydrochloric salt after *p.o.* or *i.v.* application of 10 mg/kg b.w to mice

Administration	Dose (mg/kg)	Tmax (min)	Cmax (ng/mL)	$t_{1/2}$ (min)	$AUC_{0\to\infty}$ (ng.min/mL)	Vd (mL/kg)	F bioavailability (%)
i.v.	10	10	3,950	28.7	148,000	2,790	16
p.o.	10	10	391	38.5	23,400	ND	

9. Repeated Dose 7-day Oral Toxicity Study in Rats.

All animal studies were approved by the Institutional Animal Care and Use Committees (IACUC) at the Institute of Physiology, Czech Academy of Sciences, and the Committee for Animal Protection of the Ministry of Health of the Czech Republic.

Objective of the study was to characterize the toxicity profile of compound **39** after 7-day repeated per oral (*p.o.*) administration (1 administration per day for 7 days consecutively) in rats. The study was carried out according to the ICH Guidelines M3 (R2) on non-clinical safety studies for the conduct of human clinical trials and marketing authorization for pharmaceuticals, EMA/CPMP/ICH/286/1995, 2009 and relevant Test Facility SOPs. The Wistar rats (obtained from AnLab, Prague, Czech Republic) were housed individually in breeding box with individual identification. The room temperature was 20-24 °C, and the relative humidity of air was 30-70 %. During the acclimatization and study periods the animals were fed a standard pellet diet Altromin (Lage, Germany) and water *ad libitum*. The lighting regime was 12 hours light and 12 hours dark.

Compound **39** as hydrochloric acid salt was used in three doses: 1, 10, and 30 mg/kg b.w. as a homogenous suspension in 5% glycerol). Animals were randomized into 4 groups with at least 4 animals: dose group D1 – 1 mg/kg b.w., dose group D2 – 10 mg/kg b.w., dose group D3 – 30 mg/kg b.w. and control group C1 – vehicle only (5% glycerol in saline). The groups were orally administered by vehicle or the formulation in application volume of 0.2-6 mL/ 100 g b.w. All rats were observed for signs of toxicity, morbidity or mortality once a day during acclimatization, frequently during the first 30 minutes after each dosing and then 1, 2, 4, 6, 8 and 24 hours after each dosing and daily in the morning thereafter, for a total of 8 days (including the day of necropsy). Observations included any signs of toxicity, changes in the skin and fur, eyes and mucous membranes, respiratory, circulatory, autonomic and central nervous system, somatomotor activity and behavior pattern. Attention was directed to observations of tremors, convulsion, salivation, diarrhea, lethargy, sleep, coma and changes in gait, posture and response to handling, the presence of clonic or tonic movements and stereotypes. All rats were individually weighed at delivery, before each administration and before the necropsy (D8).

No significant signs of in vivo toxicity were observed after the 7 days oral administration of compound **39** as hydrochloric salt. The body weight of all animals in all groups (D1, D2, D3, C1) corresponded to the standard growth curve. No significant changes in behavior, hematology and biochemistry parameters were observed after the 7 days oral administration of the

compound **39** hydrochloric salt and no significant changes in gross pathology were observed after the 7 days oral administration in all doses groups and the control group. Histopathology examination was performed only for D1 group (30 mg/kg). There were observed the following symptoms: Peyer's plaque hypertrophy in the small intestine (duodenum and jejunum respectively jejunum only) in two animals. In one animal venous hyperaemia of the liver was found.

The biochemical, morphological or histological data from the study are availabel on request.

10. In Vitro Predictor™ hERG Fluorescence Polarization Assay for prediction cardiotoxicity.

The assay was performed to identify potential human Ether-a-go-go-Related Gene (hERG) channel blocking by compound **39**. The PredictorTM hERG Fluorescence Polarization Assay kit (Invitrogen; Cat#PV5365) with 384-well assay plates (U-bottom, black polystyrene, Corning, USA; Cat.# 3677) has been performed according to manufacturer's instructions using a TECAN ULTRA Multifunctional Plate Reader (Tecan, Austria). IC_{50} value for E-4031, a reference compound provided by manufacturer, was found to be approximately 70 nM in accordance with the published data.

The tested compound 39 were assessed at the range of concentrations from 1 μM to 20 μM. All test points were performed in quadruplicates. Both positive and negative controls were used.

Compounds **39** showed no inhibition of the tracer binding even at 20 μM concentration. Any binding detected in this assay should be confirmed by a full electrophysiological patch-clamp analysis, though the assay demonstrates a high correlation with those results obtained from patch clamp techniques.

11. Genotoxicity - Ames test.

The mutagenic activity of the compound **39** was detected using the commercially available Muta-ChromoPlate bacterial strain kit (ebpi, Mississauga, Ontario, Canada), which is a 96-well microplate version of the reverse-mutation *Salmonella typhimurium* Ames test. The test employs two strains of *Salmonella typhimurium*, carrying mutation(s) in the operon coding for histidine biosynthesis. When these bacteria are exposed to mutagenic agents, under certain conditions reverse mutation from amino acid (histidine) auxotrophy to prototrophy may occurs. An alternate assay performed entirely in liquid culture is referred as to the 'Fluctuation test' or Ames MPF test. The test was performed and evaluated according to the manufacturer's instructions with S9 rat-liver extract. The final concentration of the tested compound **39** was 10 μM. *Salmonella typhimurium* tester strains TA 98 (detection of frame shift mutagens) and TA 100 (detection of base-exchange mutations) were used. The compounds were dissolved in DMSO, and the final concentration of DMSO in the entire reaction mixture was 0.1%. The following standard direct-acting mutagens were used as positive controls: sodium azide for use with strain TA100 (final amount of 0.5 μg) and 2-nitrofluorene for use with strain TA98 (final amount of 30 μg). A blank plate was used as a sterility control, and plates of each strain without any test compounds were used as spontaneous mutation controls (background controls). Data have been statistically analyzed according to the manufacture´s protocol.

Our results suggest that compound **39** does cause neither statistically significant frame-shift mutation (in strain ST TA98) nor base-pair substitutions (in strain ST TA100) mutagenicity (Table S-10). Compound **39** thus display no significant genotoxicity in the assay.

Table S-10. Evaluation of mutagenicity in Ames fluctuation assay performed with compound 39 on *Salmonella typhimurium* TA100 and TA98 strains at a concentration of 1 and 10 μM

Treatment	Concentration	Bacteria	Number of positive wells on day 5
Blank -S9	-	-	0
Blank +S9	-	+	0
TA100 -S9	-	+	1
TA100 +S9	-	+	18
TA98 -S9	-	+	1
TA98 +S9	-	+	11

NaN_3 TA100 -S9	-	+	51
2AA TA100 +S9	-	+	50
2NF TA98 -S9	-	+	95
2AA TA98 +S9	-	+	73
Compound **39** TA100 -S9	1 μM	+	1
Compound **39** TA100 +S9	1 μM	+	10
Compound **39** TA98 -S9	1 μM	+	4
Compound **39** TA98 +S9	1 μM	+	6
Compound **39** TA100 -S9	10 μM	+	0
Compound **39** TA100 +S9	10 μM	+	13
Compound **39** TA98 -S9	10 μM	+	3
Compound **39** TA98 +S9	10 μM	+	9

NaN_3- sodium azide (0.5 μg/100 μL), a direct-acting mutagen (i.e., does not require S-9 activation), 2NF -2-nitrofluorene (30μg/100μL), a direct-acting mutagen, 2AA -2-aminoanthracene, a S9 activated mutagen.

12. Supplementary experimental procedures

Scheme S-1

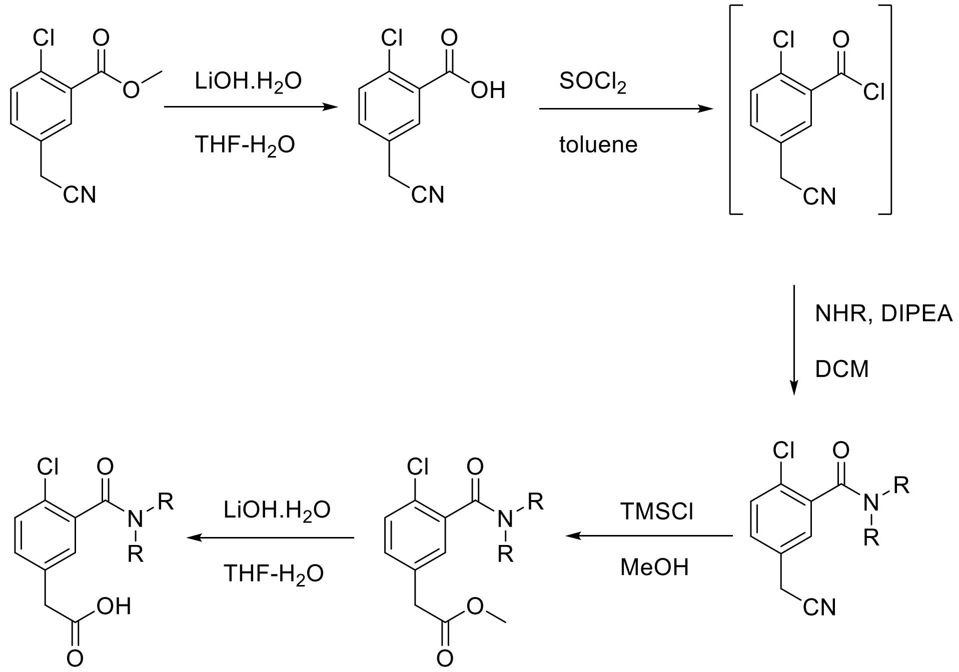

Methyl 2-chloro-5-(cyanomethyl)benzoate (intermediate compound)

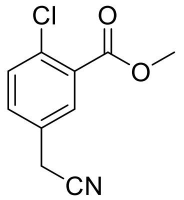

Methyl 5-(bromomethyl)-2-chlorobenzoate (1 g, 3.54 mmol) was dissolved in dry CH_3CN and the mixture was degassed and refilled with argon. K_2CO_3 (540 mg, 1.1 eq) was added in one portion followed by TMSCN (0.66 mL, 1.5 eq). The reaction mixture was stirred at 60 °C for 7 hours (Scheme 1). After cooling to 25°C the mixture was quenched with 1 M NaOH, extracted with EtOAc and dried over sodium sulfate. Residue was purified by flash column chromatography, mobile phase petrolether/EtOAc (15-60 %). Yield 415 mg (56 %) as oil. ^1H NMR (401 MHz, DMSO-d_6) δ 7.83 – 7.81 (m, 1H), 7.63 (d, J = 8.4 Hz, 1H), 7.57 (ddt, J = 8.3, 2.3, 0.7 Hz, 1H), 4.14 (t, J = 0.7 Hz, 2H), 3.88 (s, 3H). ^{13}C NMR (101 MHz, DMSO) δ 165.50, 133.30, 131.85, 131.61, 131.45, 131.08, 130.72, 119.14, 53.16, 22.12. HRMS: calcd for [M + H], 210.03163; found, 210.03151.

2-Chloro-5-(cyanomethyl)benzoic acid (intermediate compound)

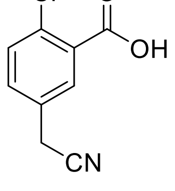

Title compound was prepared according to the General procedure V (SI Scheme 1). Mobile phase H_2O/CH_3CN 10-70 %. ^1H NMR (401 MHz, DMSO-d_6) δ 13.16 (s, 3H), 7.78 (d, J = 2.3 Hz, 1H), 7.58 (d, J = 8.2 Hz, 1H), 7.51 (dd, J = 8.3, 2.3 Hz, 1H), 4.11 (s, 2H). HRMS: calcd for [M - H], 194.00143; found, 194.00129.

2-Chloro-5-(cyanomethyl)-N,N-dimethylbenzamide (intermediate compound)

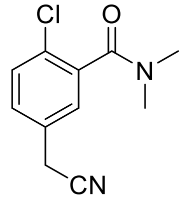

Title compound was prepared according to the General Procedure VI (SI Scheme 1). Mobile phase H_2O/CH_3CN (10-70 %). Yield: 125 mg (86 %). ^1H NMR (401 MHz, DMSO-d_6) δ 7.56 (d, J = 8.3 Hz, 1H), 7.48 – 7.39 (m, 1H), 7.33 (d, J = 2.2 Hz, 1H), 4.08 (s, 2H), 3.01 (s, 3H), 2.77 (s, 3H). ^{13}C NMR (101 MHz, DMSO) δ 166.66, 136.91, 131.34, 130.23, 130.10, 128.50, 127.71, 118.97, 37.67, 34.21, 21.93. HRMS: calcd for [M + H], 223.06327; found, 223.06327.

2-Chloro-5-(cyanomethyl)-N-methylbenzamide (intermediate compound)

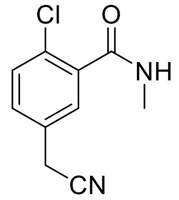

Title compound was prepared according to the General Procedure VI (SI Scheme 1). Mobile phase H_2O/CH_3CN (10-70 %). Yield: 235 mg (91 %). ^1H NMR (401 MHz, DMSO-d_6) δ 8.41 (q, J = 4.7 Hz, 1H), 7.53 (dd, J = 8.0, 0.7 Hz, 1H), 7.47 – 7.33 (m, 2H), 4.07 (t, J = 0.7 Hz, 2H), 2.75 (d, J = 4.6 Hz, 3H). ^{13}C NMR (101 MHz, DMSO) δ 166.70, 137.91, 130.98, 130.81, 130.62, 129.59, 129.01, 119.26, 26.85, 22.11. HRMS: calcd for [M + H], 209.04762; found, 209.04754.

2-Chloro-5-(cyanomethyl)benzamide (intermediate compound)

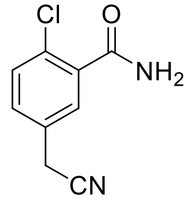

Title compound was prepared according to the General Procedure VI (SI Scheme 1). Mobile phase H_2O/CH_3CN (10-70 %). Yield: 235 mg (91 %). ^1H NMR (401 MHz, DMSO-d_6) δ 7.93 (s, 1H), 7.66 (s, 1H), 7.52 (d, J = 8.1 Hz, 1H), 7.47 – 7.36 (m, 2H), 4.08 (s, 2H). ^{13}C NMR (101 MHz, DMSO) δ 167.87, 137.76, 130.62, 130.39, 130.34, 129.07, 128.51, 119.00, 21.84. HRMS: calcd for [M + Na], 217.01391; found, 217.01405.

SI General procedure I. - Esterification of cyano group

Cyanomethyl benzamide derivative was dissolved in small amount of dry MeOH and TMSCl (2eq) was added. A reaction mixture was stirred at 50 °C for 4 h and then at 25°C overnight. The reaction mixture was diluted with water and extracted with EtOAc. Combined organic phases were dried over sodium sulfate and purified by reverse-phase column chromatography (Scheme 1).

Methyl 2-(4-chloro-3-(dimethylcarbamoyl)phenyl)acetate (intermediate compound)

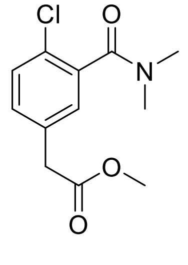

Title compound was prepared according to the SI General Procedure I (SI Scheme 1). Mobile phase H_2O/CH_3CN (10-70 %). Yield: 110 mg (97 %). ^1H NMR (401 MHz, DMSO-d_6) δ 7.47 (d, J = 8.3 Hz, 1H), 7.33 (dd, J = 8.3, 2.2 Hz, 1H), 7.25 (d, J = 2.1 Hz, 1H), 3.74 (s, 2H), 3.62 (s, 2H), 3.00 (s, 2H), 2.76 (s, 2H). ^{13}C NMR (101 MHz, DMSO) δ 171.65, 167.31, 136.55, 134.58, 131.89,

129.66, 129.31, 127.98, 52.30, 39.39, 37.96, 34.45. HRMS: calcd for [M + H], 256.07350; found, 256.07370.

Methyl 2-(4-chloro-3-(methylcarbamoyl)phenyl)acetate (intermediate compound)

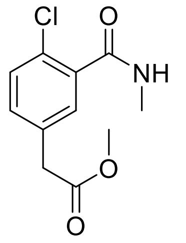

Title compound was prepared according to the SI General Procedure I (SI Scheme 1). Mobile phase H_2O/CH_3CN (10-70 %). Yield: 118 mg (61 %). ^1H NMR (401 MHz, DMSO-d_6) δ 8.35 (d, J = 5.0 Hz, 1H), 7.46 – 7.43 (m, 1H), 7.35 – 7.31 (m, 2H), 3.74 (s, 2H), 3.63 (s, 3H), 2.75 (d, J = 4.6 Hz, 3H).^{13}C NMR (101 MHz, DMSO) δ 171.64, 167.02, 137.40, 134.00, 132.17, 130.25, 129.91, 128.77, 52.29, 26.42. HRMS: calcd for [M + H], 242.05785; found, 242.05789.

Methyl 2-(3-carbamoyl-4-chlorophenyl)acetate (intermediate compound)

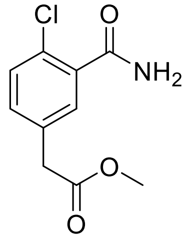

Title compound was prepared according to the SI General Procedure I (SI Scheme 1). Mobile phase H_2O/CH_3CN (10-70 %). Yield: 51 mg (53 %). ^1H NMR (401 MHz, DMSO-d_6) δ 7.88 (d, J = 6.2 Hz, 2H), 7.44 (d, J = 8.1 Hz, 1H), 7.36 (d, J = 2.1 Hz, 1H), 7.31 (d, J = 2.0 Hz, 1H), 3.74 (s, 2H), 3.63 (s, 3H), .^{13}C NMR (101 MHz, DMSO) δ 171.66, 168.61, 137.48, 135.75, 132.06, 130.04, 129.79, 128.56, 52.29, 39.46. HRMS: calcd for [M + H], 228.04220; found, 228.04221.

2-(4-Chloro-3-(dimethylcarbamoyl)phenyl)acetic acid (intermediate compound)

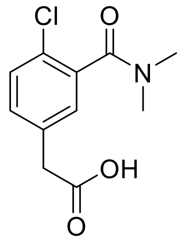

Title compound was prepared according to the SI General Procedure I (SI Scheme 1). Mobile phase H_2O/CH_3CN (10-70 %). Yield: 228 mg (97 %). ^1H NMR (401 MHz, DMSO-d_6) δ 12.47 (s, 1H), 7.45 (d, J = 8.3 Hz, 1H), 7.32 (dd, J = 8.3, 2.2 Hz, 1H), 7.23 (d, J = 2.2 Hz, 1H), 3.62 (s, 2H), 3.00 (s, 3H), 2.76 (s, 3H). ^{13}C NMR (101 MHz, DMSO) δ 172.36, 167.09, 136.17, 134.98, 131.63, 129.24, 129.01, 127.44, 37.67, 34.16. dmso overlap HRMS: calcd for [M + H], 242.05785; found, 242.05764.

2-(4-Chloro-3-(methylcarbamoyl)phenyl)acetic acid (intermediate compound)

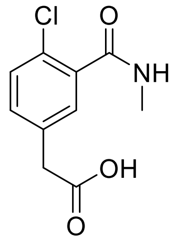

Title compound was prepared according to the SI General Procedure I (SI Scheme 1). Mobile phase H_2O/CH_3CN (10-70 %). Yield: 180 mg (97 %). ^1H NMR (401 MHz, DMSO-d_6) δ 12.45 (s, 1H), 8.34 (q, J = 4.6 Hz, 1H), 7.45 – 7.40 (m, 1H), 7.35 – 7.30 (m, 2H), 3.62 (s, 2H), 2.75 (d, J = 4.6 Hz, 3H). ^{13}C NMR (101 MHz, DMSO) δ 172.67, 167.13, 137.28, 134.66, 132.19, 130.24, 129.79, 128.54, 39.99, 26.42. HRMS: calcd for [M + Na], 250.02414; found, 250.02402.

2-(3-Carbamoyl-4-chlorophenyl)acetic acid (intermediate compound)

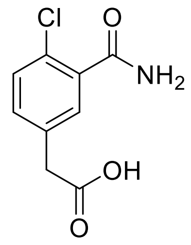

Title compound was prepared according to the SI General Procedure I (SI Scheme 1). Mobile phase H_2O/CH_3CN (10-70 %). Yield: 34 mg (90 %). ^1H NMR (401 MHz, DMSO-d_6) δ 12.51 (bs, 1H), 7.87 (s, 1H), 7.58 (s, 1H), 7.41 (d, J = 8.1 Hz, 1H), 7.32 (dt, J = 8.1, 4.0 Hz, 2H), 3.61 (s, 2H). ^{13}C NMR (101 MHz, DMSO) δ 172.45, 168.32, 137.11, 134.39, 131.82, 129.76, 129.53, 128.01, 39.81. HRMS: calcd for [M + Na], 236.00849; found, 236.00852

13. Elemental analyses

Table S-11.

Comp.	Formula	Calculated (%)			Found (%)		
		**C**	**H**	**N**	**C**	**H**	**N**
**2**	$C_{20}H_{12}Cl_3N_5S$	¸52.14	2.63	15.20	52.51	2.95	14.97
**3**	$C_{22}H_{14}Cl_3N_5$	58.11	3.10	15.40	57.89	3.15	15.06
**12g**	$C_{20}H_{11}Cl_4N_5S.0.5H_2O$	47.64	2.40	13.89	47.41	2.22	13.68
**13b**	$C_{23}H_{17}Cl_2N_5$	63.61	3.95	16.13	63.22	4.23	15.87
**13c**	$C_{24}H_{19}Cl_2N_5.0.17H_2O$	63.87	4.32	15.52	63.77	4.24	15.33
**13d**	$C_{22}H_{14}Cl_2FN_5$	60.29	3.22	15.98	60.18	3.22	15.73
**13e**	$C_{23}H_{14}Cl_2F_3N_5.0.33MeOH$	56.17	3.10	14.04	56.06	3.26	13.72
**13f**	$C_{22}H_{13}Cl_4N_5$	54.02	2.68	14.32	53.98	3.00	13.93
**13g**	$C_{22}H_{13}Cl_4N_5.0.33EtOH$	53.96	3.00	13.88	54.18	3.02	13.81
**13h**	$C_{23}H_{14}Cl_2N_6.0.33MeOH$	61.46	3.39	18.43	61.35	3.18	18.25
**13i**	$C_{23}H_{17}Cl_2N_5O.0.5H_2O$	60.14	3.95	15.25	60.26	3.74	15.08
**14a**	$C_{20}H_{13}Cl_2N_5S$	56.35	3.07	16.43	56.68	3.26	16.09
**14b**	$C_{22}H_{19}N_5O_2S$	58.47	4.01	15.50	58.07	4.01	15.13
**14c**	$C_{21}H_{16}ClN_5OS$	59.78	3.82	16.60	59.41	3.80	16.26
**14d**	$C_{20}H_{14}ClN_5S.0.33H_2O$	60.37	3.72	17.60	60.52	3.60	17.24
**14e**	$C_{19}H_{13}ClN_6S$	58.09	3.34	21.39	58.16	3.45	20.92
**14f**	$C_{21}H_{13}ClN_6S$	60.50	3.14	20.16	60.61	3.15	19.78
**15a**	$C_{22}H_{15}Cl_2N_5$	62.87	3.60	16.66	62.90	3.73	16.35
**15b**	$C_{24}H_{20}ClN_5O_2.0.66H_2O$	62.95	4.70	15.29	62.87	4.45	14.95
**15c**	$C_{23}H_{18}ClN_5O$	66.43	4.36	16.84	66.20	4.44	16.68
**15d**	$C_{22}H_{16}ClN_5.0.25EtOH$	68.01	4.44	17.62	68.16	4.41	17.40
**15e**	$C_{21}H_{15}ClN_6.H_2O$	62.30	4.23	20.76	62.54	4.02	20.28
**15f**	$C_{23}H_{15}ClN_6$	67.24	3.68	20.54	67.14	3.62	20.19
**15g**	$C_{23}H_{18}ClN_5S$	63.96	4.20	16.21	63.64	3.98	15.98
**15h**	$C_{23}H_{18}ClN_5$	69.08	4.54	17.51	68.77	4.33	17.14
**15i**	$C_{23}H_{18}ClN_5O_2S$	59.54	3.91	15.10	59.30	3.75	14.78
**15j**	$C_{26}H_{23}ClN_6O_2S$	60.17	4.47	16.19	59.87	4.22	15.88
**15k**	$C_{22}H_{15}ClN_6O_2$	61.33	3.51	19.51	60.98	3.22	19.21
**15l**	$C_{22}H_{17}ClN_6$	65.92	4.27	20.96	65.74	3.99	20.58
**15m**	$C_{24}H_{19}ClN_6O$	65.08	4.32	18.98	64.88	4.12	18.79
**16A**	$C_{20}H_{11}Cl_3N_4OS$	52.02	2.40	12.13	51.68	2.45	11.92
**16B**	$C_{22}H_{13}Cl_3N_4O$	57.98	2.88	12.29	57.63	2.96	12.04
**17**	$C_{22}H_{13}Cl_3N_4O$	57.98	2.88	12.29	57.59	3.02	11.90
**18**	$C_{22}H_{13}Cl_3N_4S$	56.01	2.78	11.88	55.87	3.03	11.64
**19A**	$C_{21}H_{12}Cl_3N_3S_2$	52.90	2.54	8.81	53.10	2.60	8.60
**19B**	$C_{23}H_{14}Cl_3N_3S$	58.68	3..00	8.93	58.34	2.97	8.72
**20**	$C_{23}H_{16}ClN_3O$	71.60	4.18	10.89	71.24	4.45	10.61
**21**	$C_{24}H_{16}Cl_3N_3$	63.67	3.56	9.28	63.35	3.78	8.99
**22**	$C_{23}H_{15}Cl_3N_4$	60.88	3.33	12.35	60.56	3.45	12.08
**23**	$C_{21}H_{12}Cl_3N_5$	57.23	2.74	15.89	57.02	2.85	15.79
**24**	$C_{23}H_{16}Cl_3N_5$	58.93	3.44	14.94	58.87	3.48	14.89

**34**	C15H10ClN5	60.92	3.41	23.68	60.88	3.50	23.45
**37**	C24H17Cl2N5O2	60.26	3.58	14.64	60.14	3.57	14.38
**38**	C23H15Cl2N5O2	59.50	3.26	15.08	59.24	3.33	14.98
**39**	C23H18Cl2N6	61.48	4.04	18.70	61.22	4.14	18.56
**39HCl**	C23H18Cl2N6.HCl	55.27	3.43	16.82	54.89	3.80	16.50
**40**	C24H18Cl2N6O	60.39	3.80	17.61	60.11	3.91	17.45
**41**	C25H20Cl2N6O	61.11	4.10	17.10	61.02	4.00	16.89
**42**	C25H20Cl2N6O2	59.18	3.97	16.56	58.87	4.01	16.43
**43**	C24H17Cl2N5O	62.35	3.71	15.15	62.13	3.84	14.93
**44**	C25H19Cl2N5O	63.04	4.02	14.70	62.79	4.22	14.45
**45**	C23H15Cl2N5O	61.62	3.37	15.62	61.44	3.40	15.23
**46**	C23H16Cl2N6O	59.62	3.48	18.14	59.28	3.51	17.99
**47**	C23H17Cl2N5O	61.35	3.81	15.55	61.02	3.95	15.20
**48**	C24H19Cl2N5O	62.08	4.12	15.08	61.77	4.26	14.90
**49**	C22H14Cl2N6O2	56.79	3.03	18.06	56.56	3.15	18.24
**50**	C22H16Cl2N6	60.70	3.70	19.31	60.55	3.81	19.09
**51**	C24H18Cl2N6O	60.39	3.80	17.61	60.56	3.98	17.28
**55**	C26H19ClN2	79.08	4.85	7.09	79.26	4.99	6.87
**56**	C25H18ClN3	75.85	4.58	10.61	75.94	4.66	10.35
**60**	C25H17Cl2N5O	63.30	3.61	14.76	63.42	3.78	14.56
**61**	C25H16Cl2N4O2	63.17	3.39	11.79	63.24	3.50	11.63

14. SI NMR spectra

2

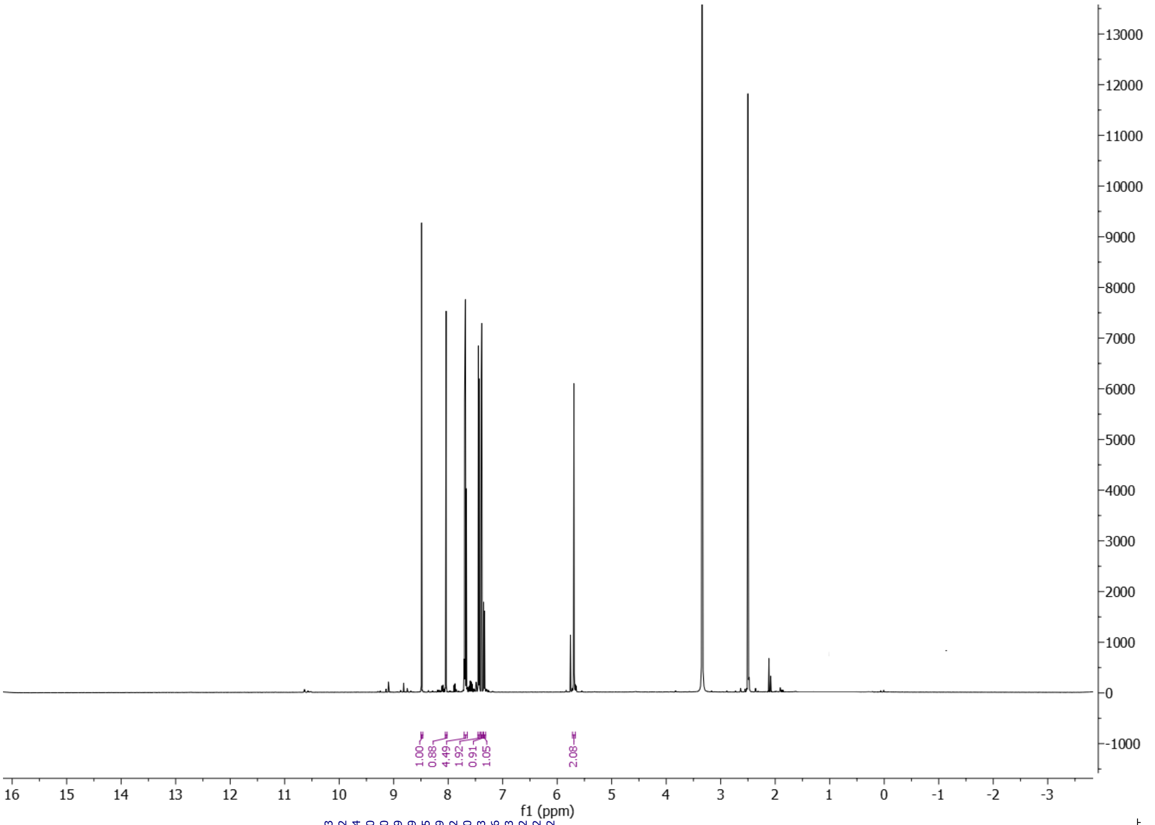

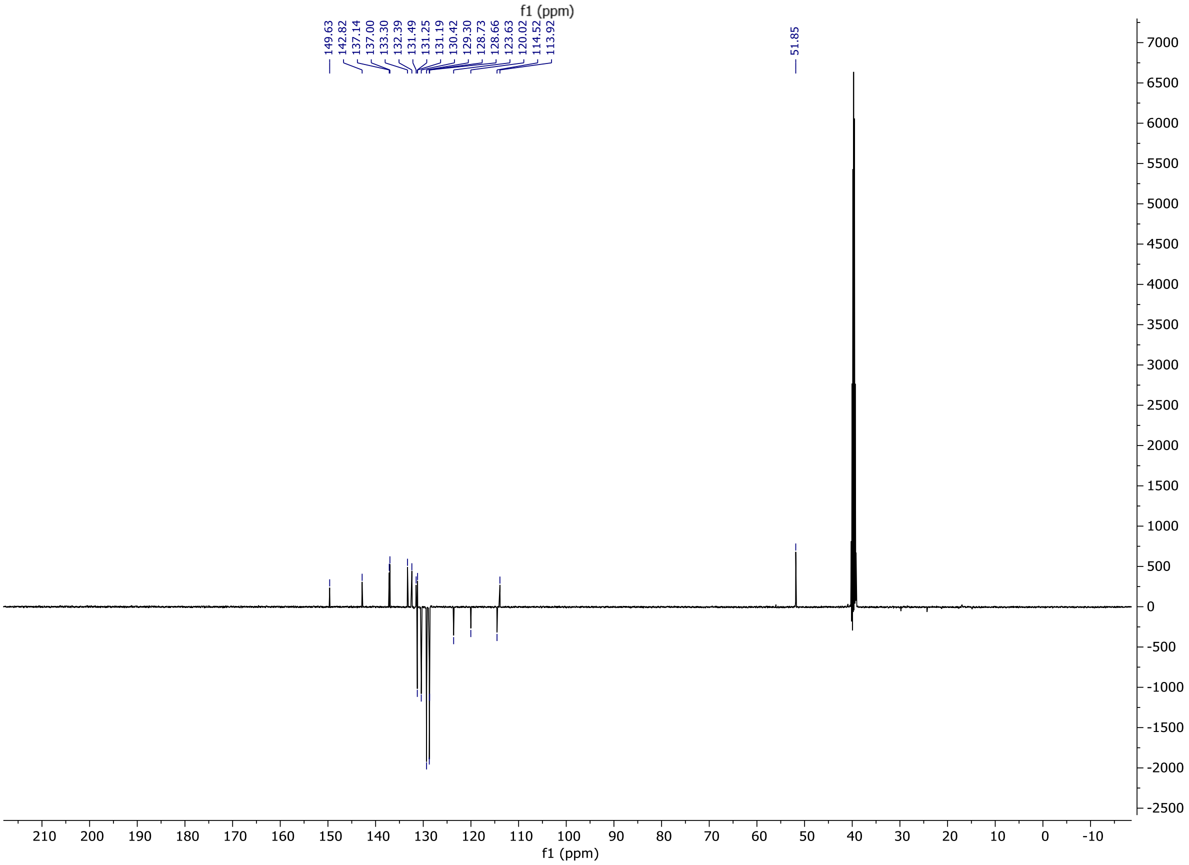

3

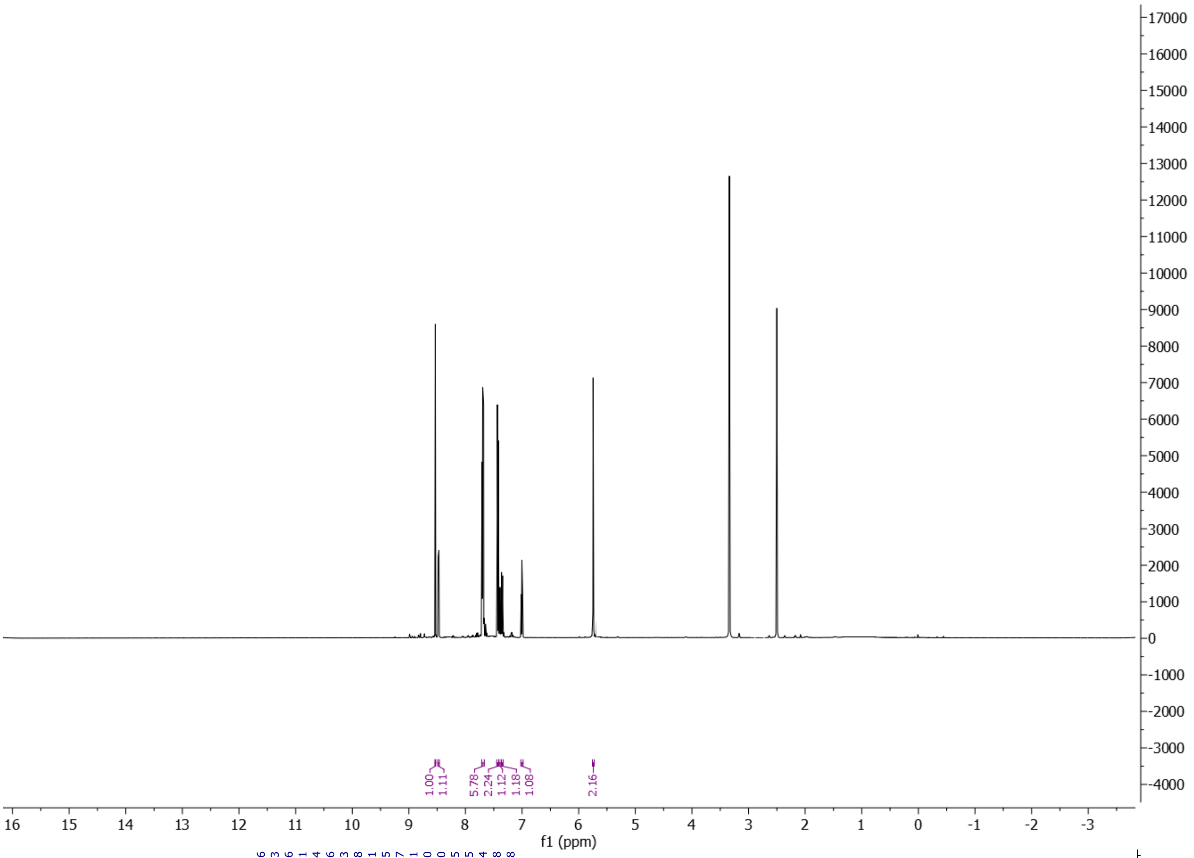
1.00
1.11
5.78
2.24
1.12
1.18
1.08
2.16
f1 (ppm)

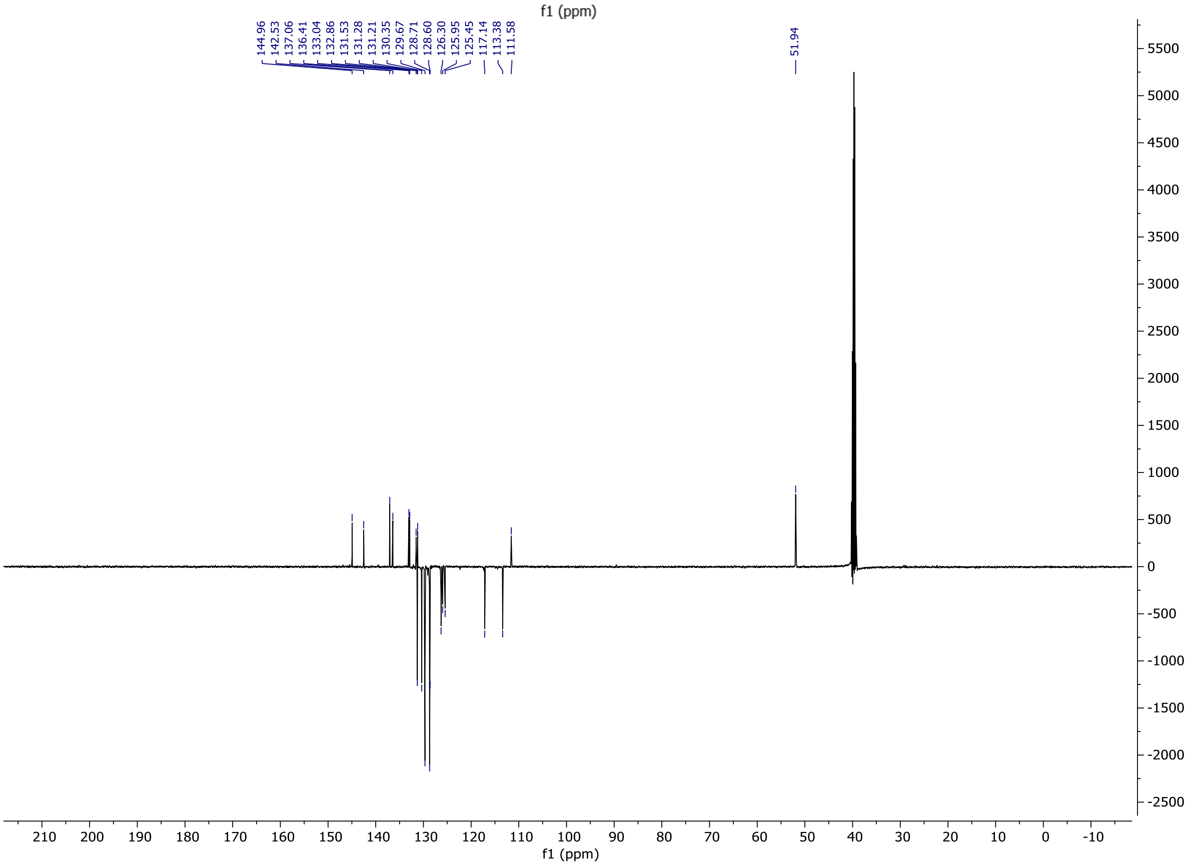
144.96
142.53
137.06
136.41
133.04
132.86
131.53
131.28
131.21
130.35
129.67
128.71
128.60
126.30
125.95
125.45
117.14
113.38
111.58
51.94
f1 (ppm)

12g

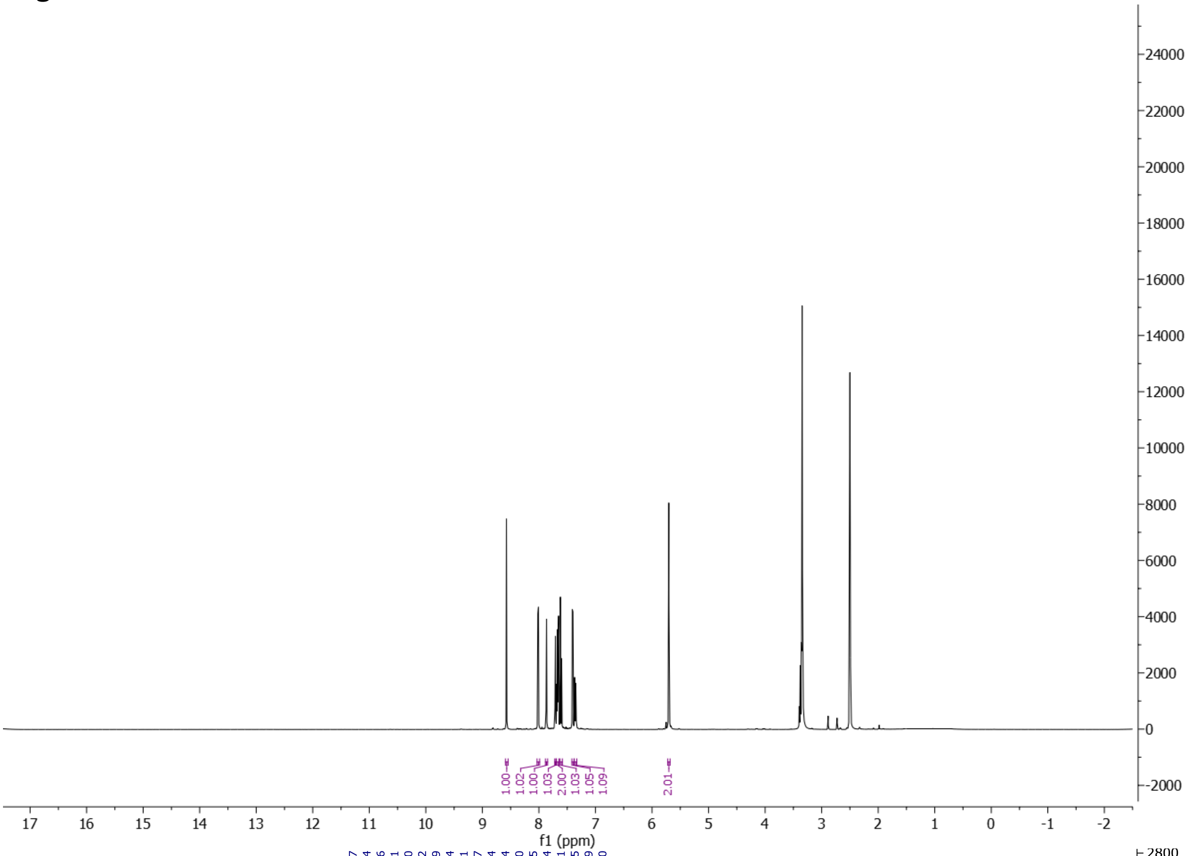
1.00
1.02
1.00
1.03
2.00
1.03
1.05
1.09
2.01
f1 (ppm)

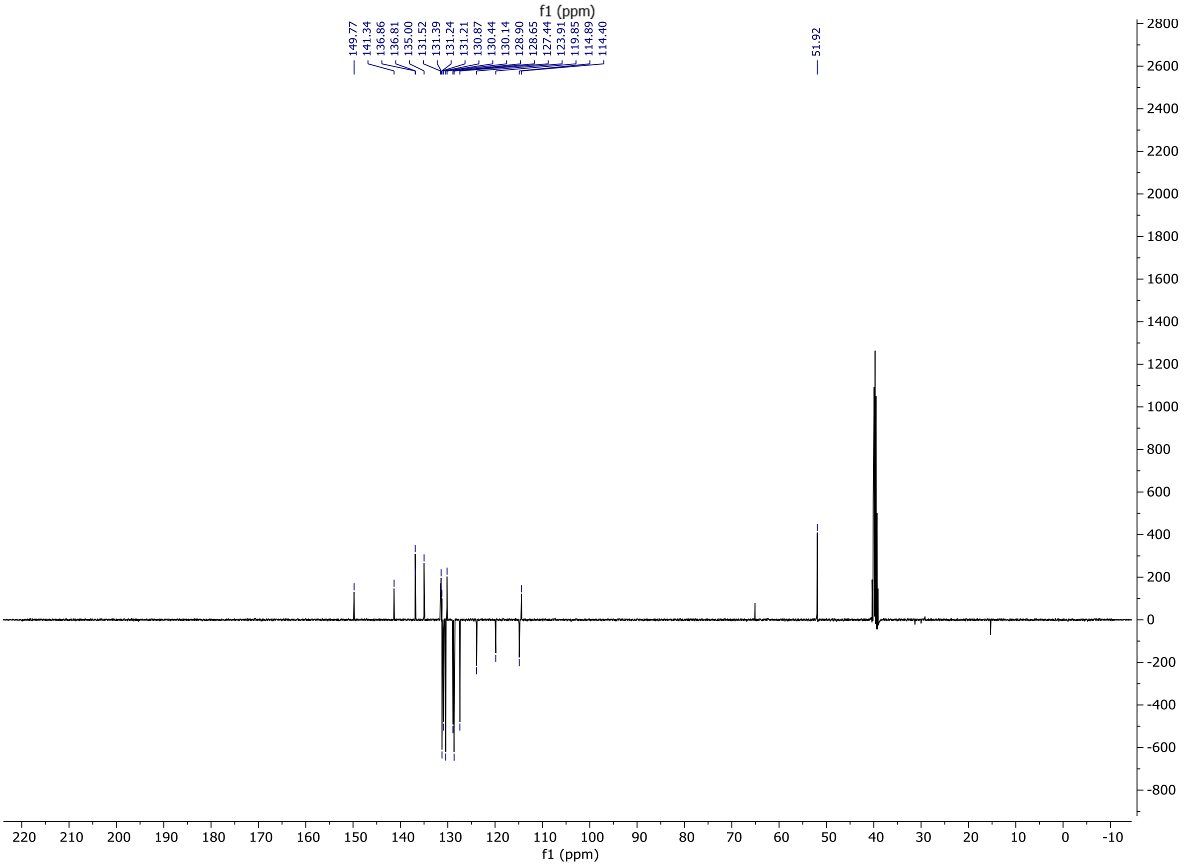
149.77
141.34
136.86
136.81
135.00
131.52
131.39
131.24
131.21
130.87
130.44
130.14
128.90
128.65
127.44
123.91
119.85
114.89
114.40
51.92
f1 (ppm)

13b

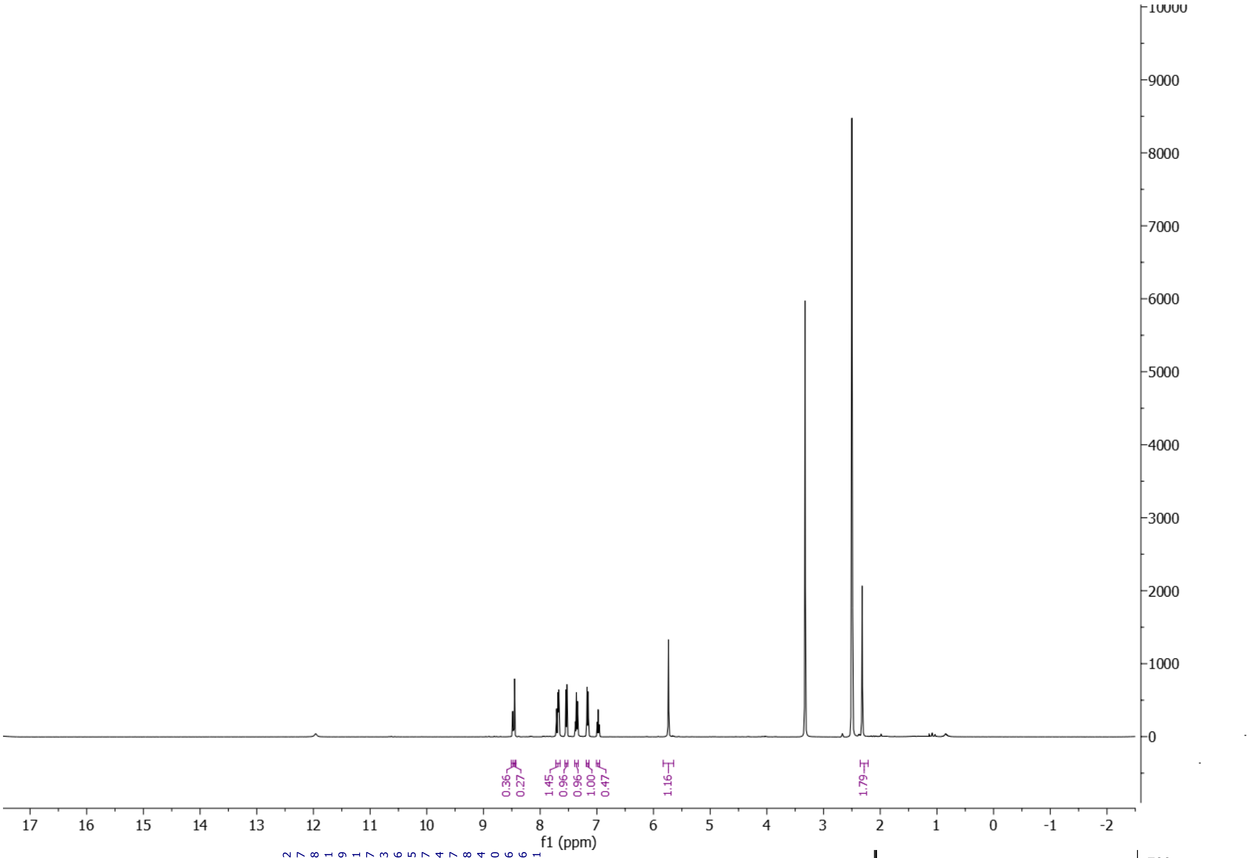

0.36
0.27
1.45
0.96
0.96
1.00
0.47
1.16
1.79
f1 (ppm)

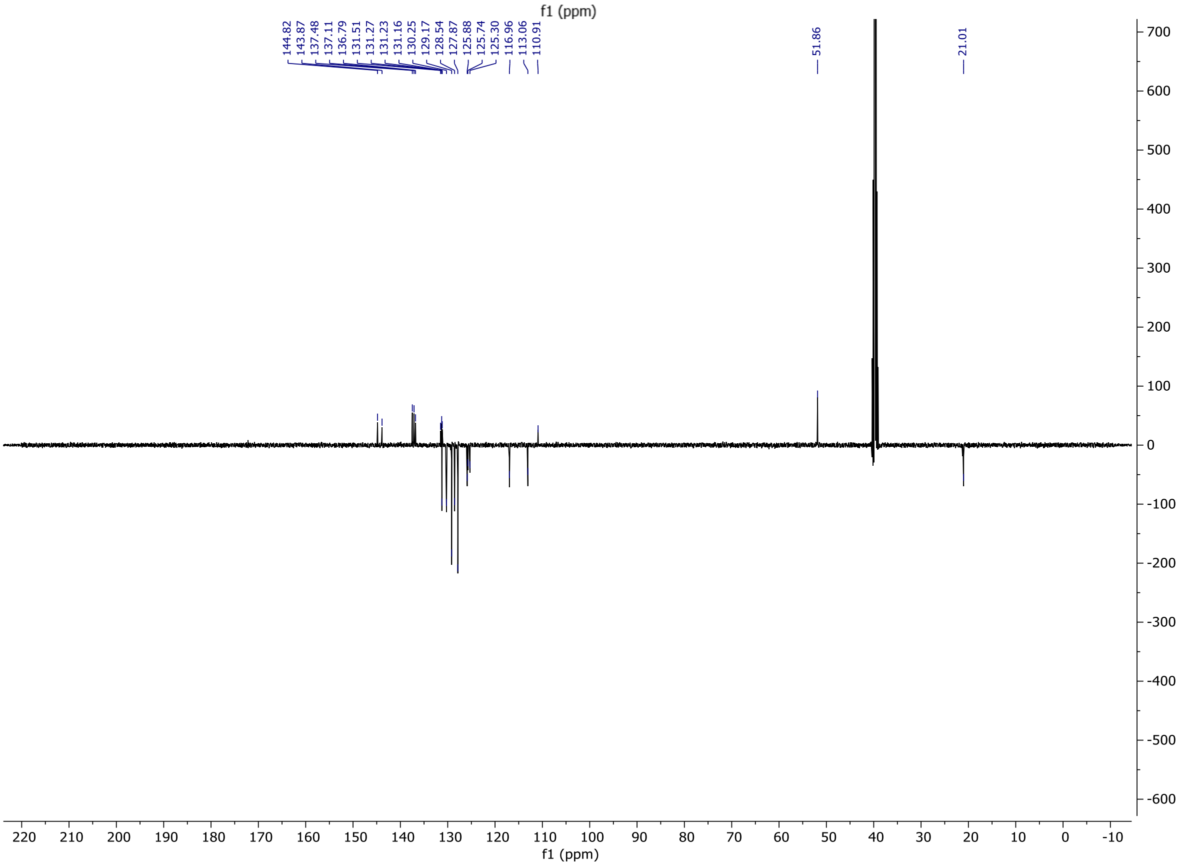

144.82
143.87
137.48
137.11
136.79
131.51
131.27
131.23
131.16
130.25
129.17
128.54
127.87
125.88
125.74
125.30
116.96
113.06
110.91
51.86
21.01
f1 (ppm)

13c

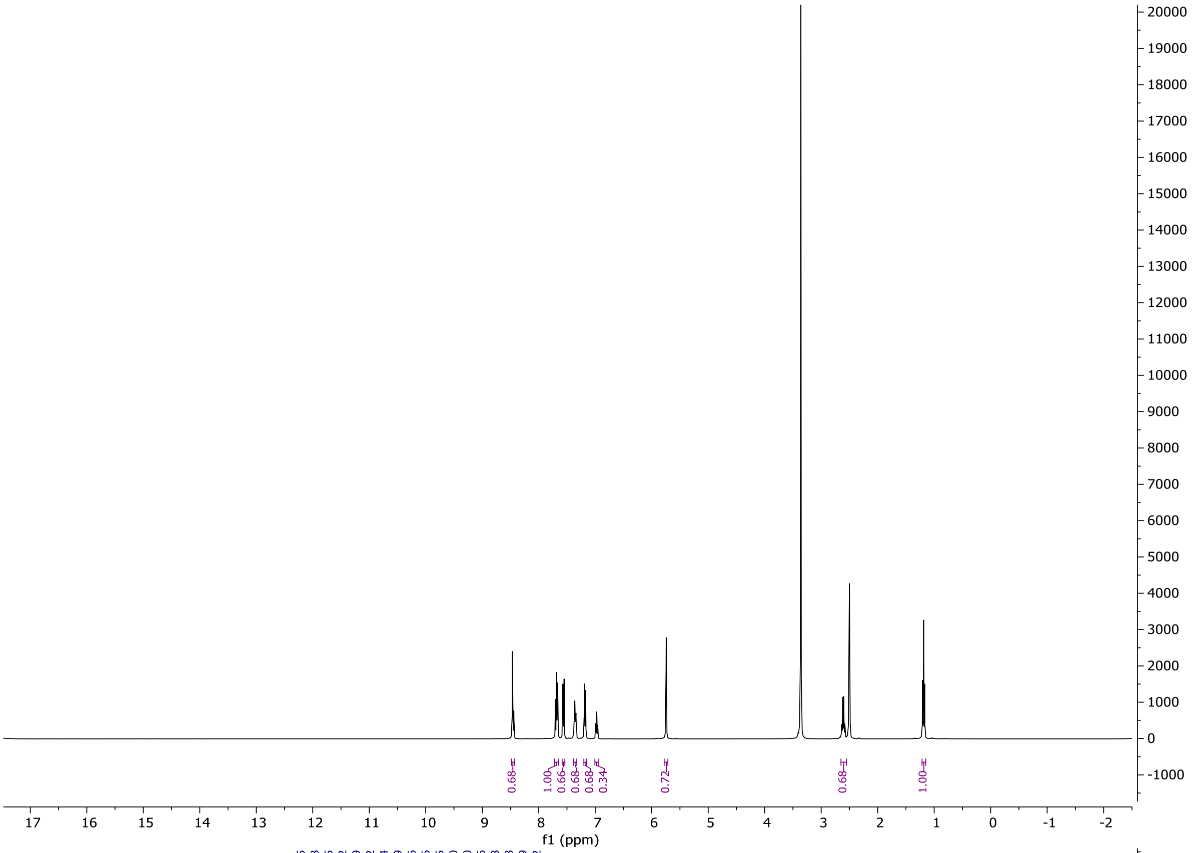

0.68
1.00
0.66
0.68
0.68
0.34
0.72
0.68
1.00
f1 (ppm)

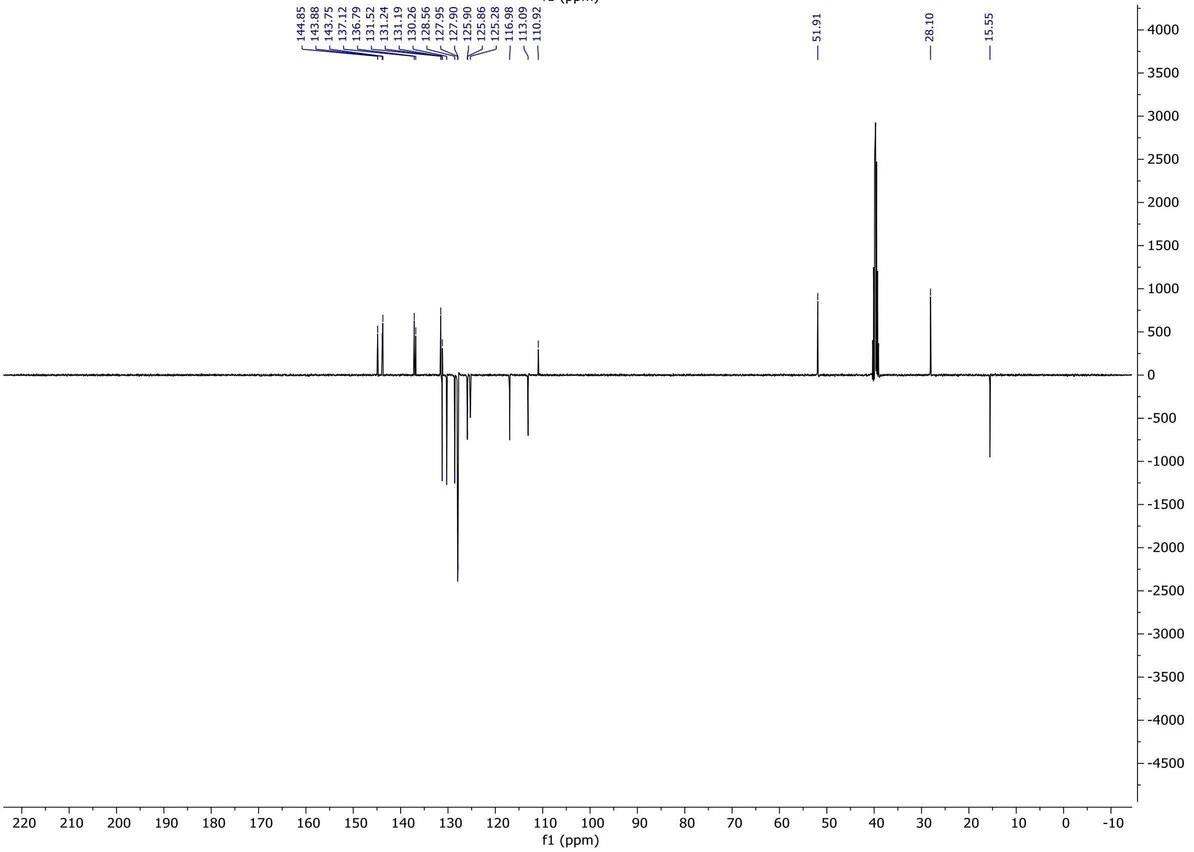

144.85
143.88
143.75
137.12
136.79
131.52
131.24
131.19
130.26
128.56
127.95
127.90
125.90
125.86
125.28
116.98
113.09
110.92
51.91
28.10
15.55
f1 (ppm)

13d

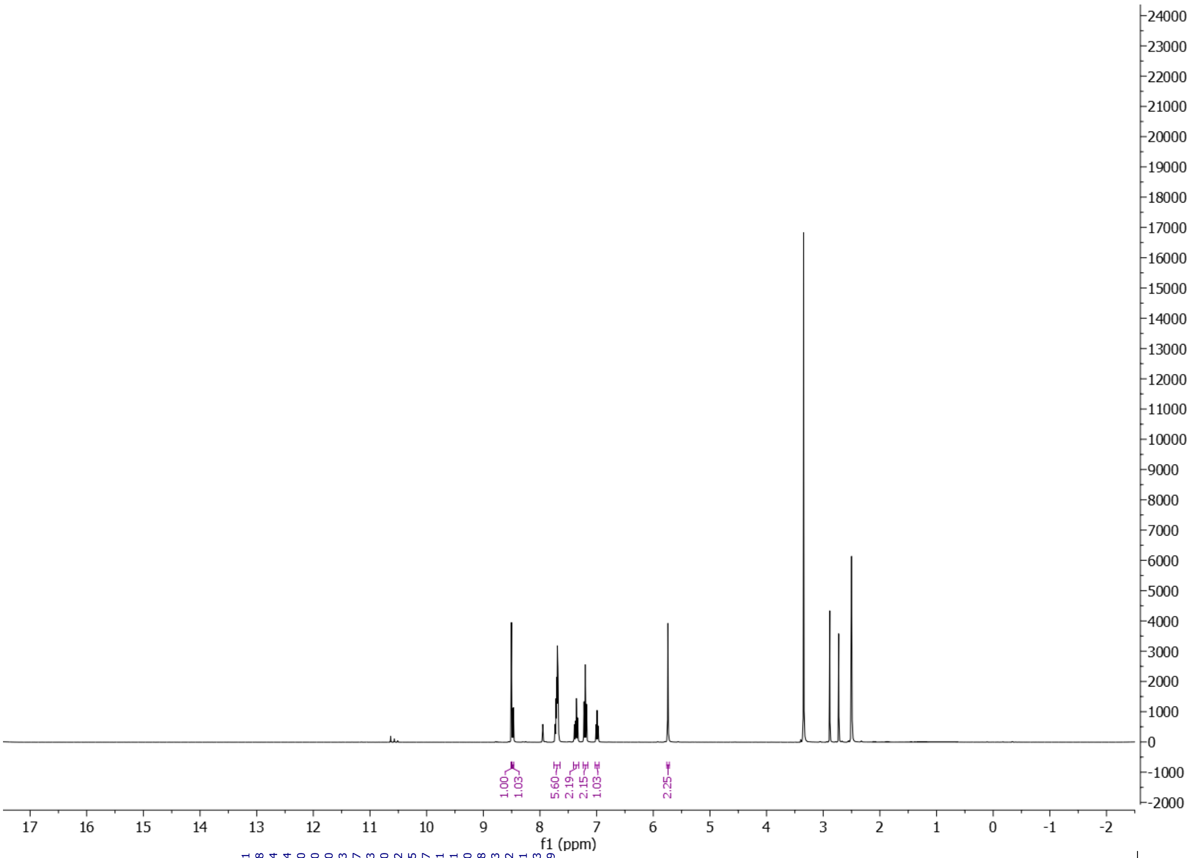
1.00
1.03
5.60
2.19
2.15
1.03
2.25
f1 (ppm)

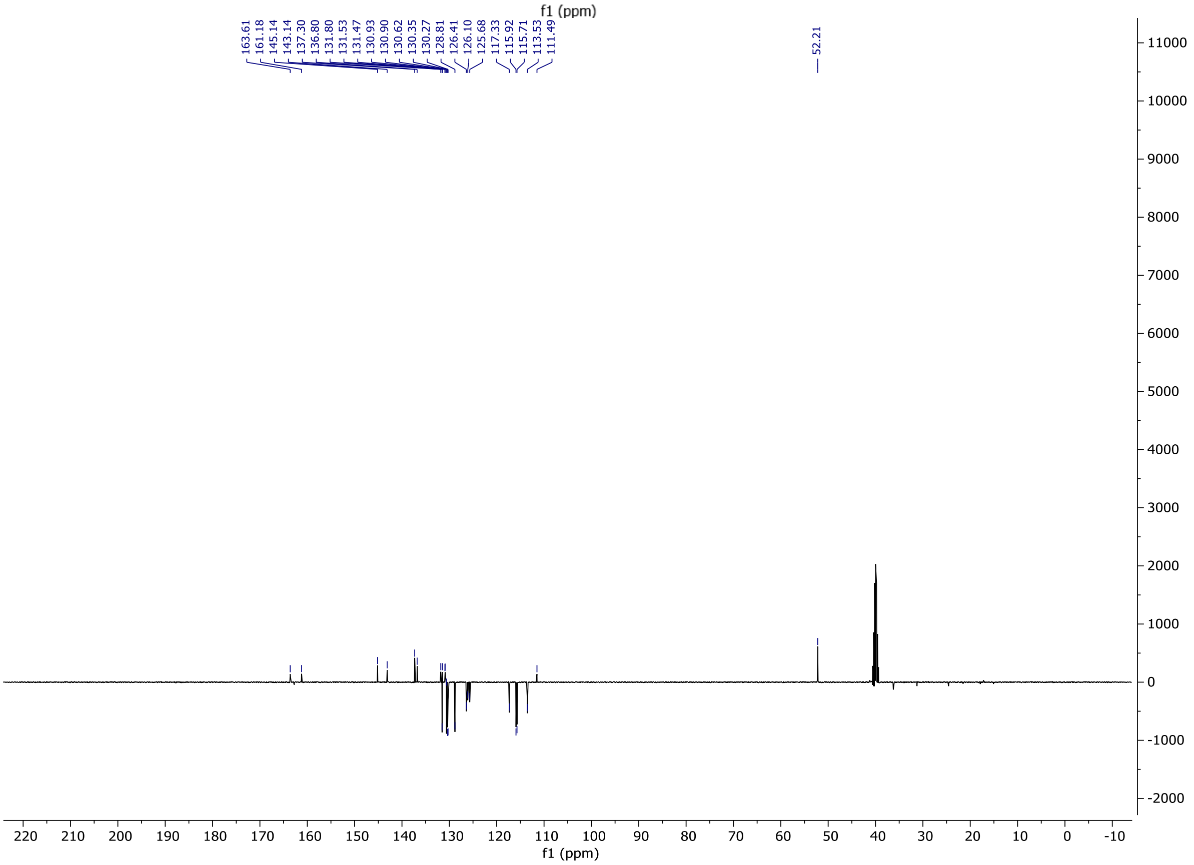
163.61
161.18
145.14
143.14
137.30
136.80
131.80
131.53
131.47
130.93
130.90
130.62
130.35
130.27
128.81
126.41
126.10
125.68
117.33
115.92
115.71
113.53
111.49
52.21
f1 (ppm)

13e

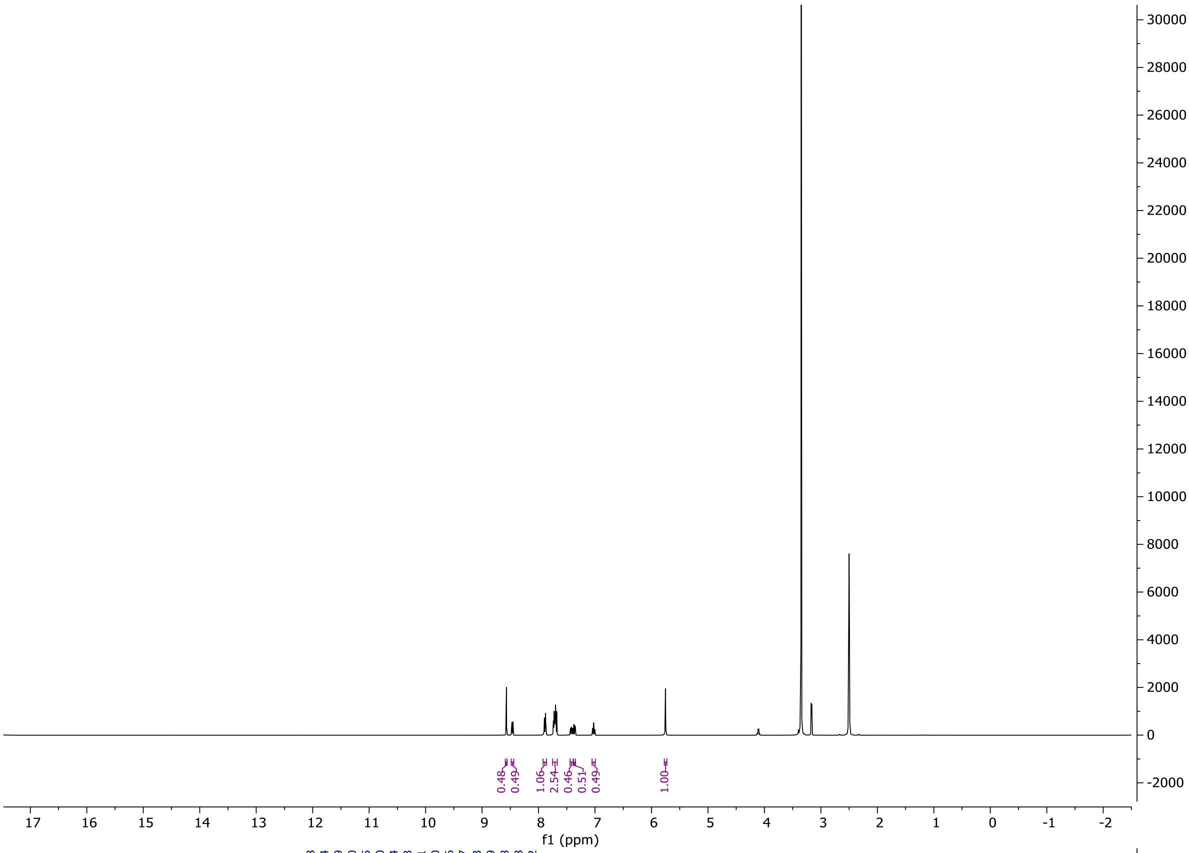
0.48
0.49
1.06
2.54
0.46
0.51
0.49
1.00
f1 (ppm)

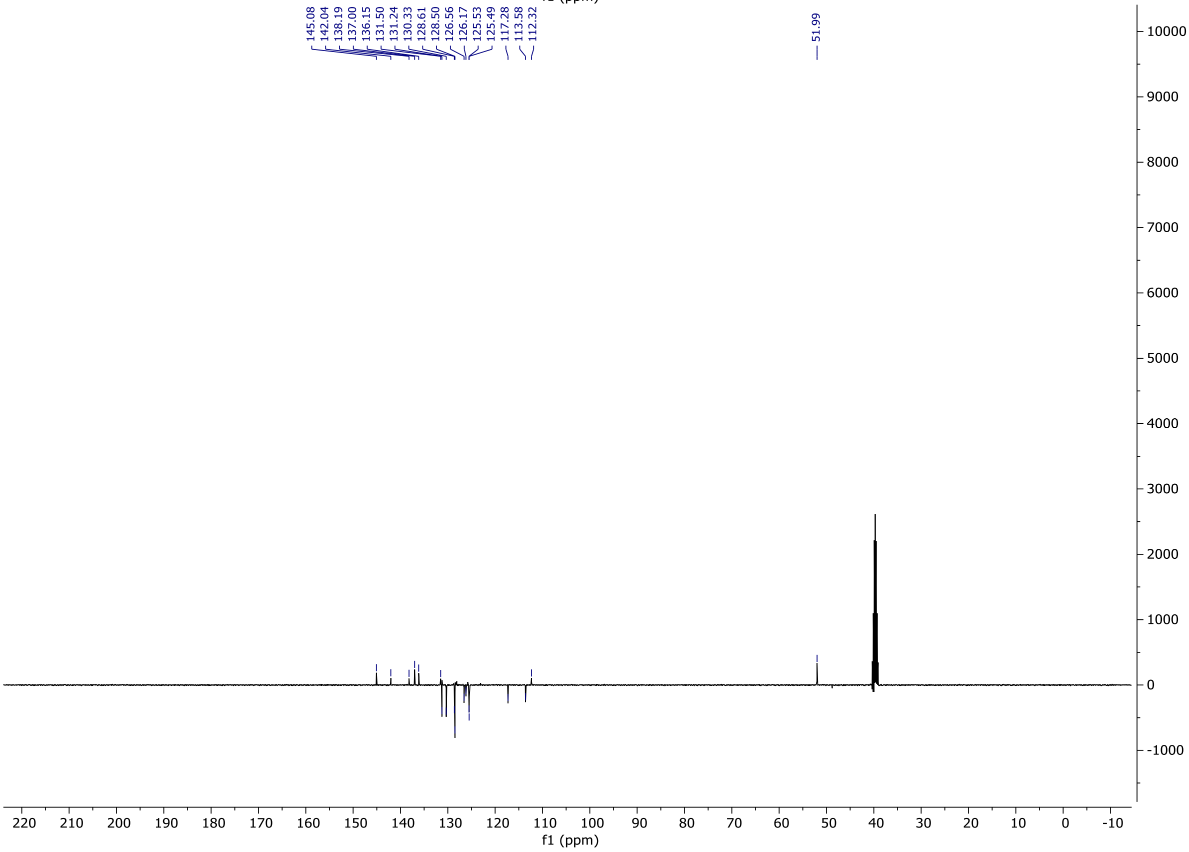
145.08
142.04
138.19
137.00
136.15
131.50
131.24
130.33
128.61
128.50
126.56
126.17
125.53
125.49
117.28
113.58
112.32
51.99
f1 (ppm)

13f

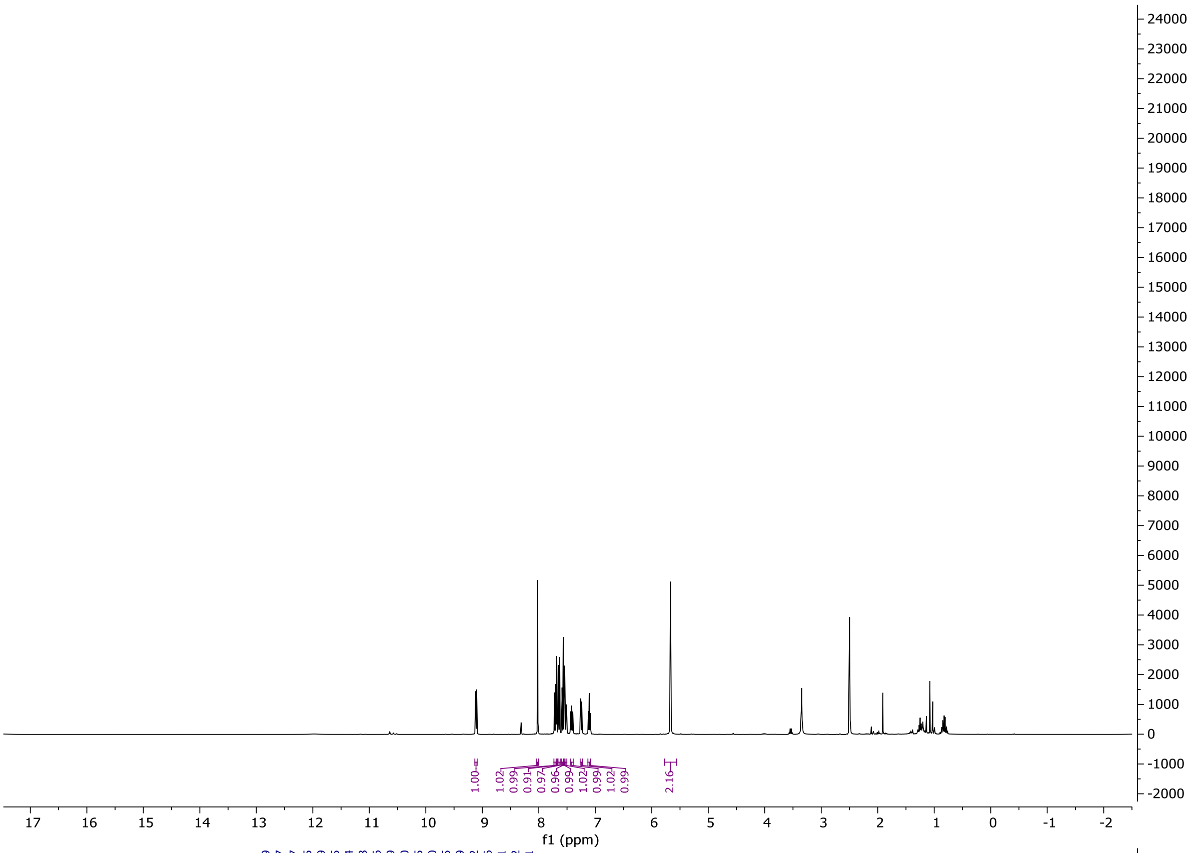

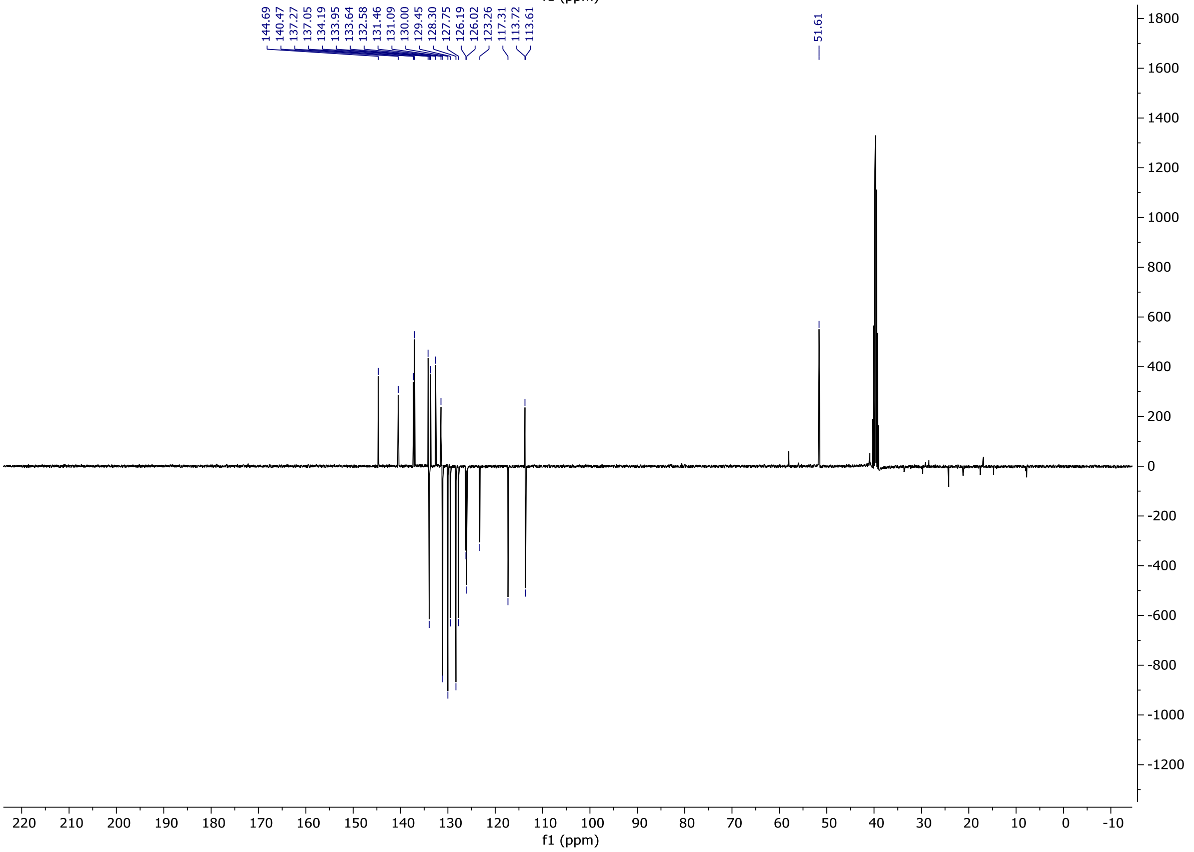

13g

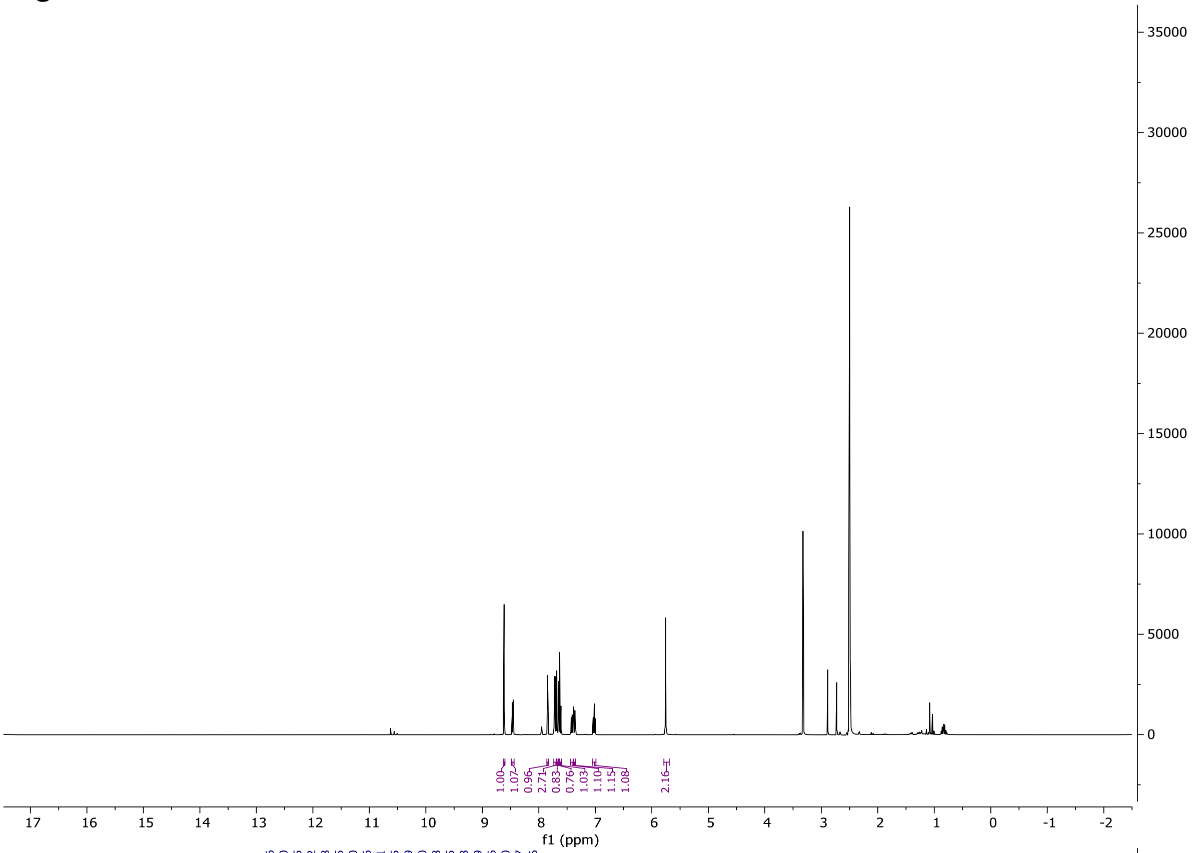

1.00
1.07
0.96
2.71
0.83
0.76
1.03
1.10
1.15
1.08
2.16
f1 (ppm)

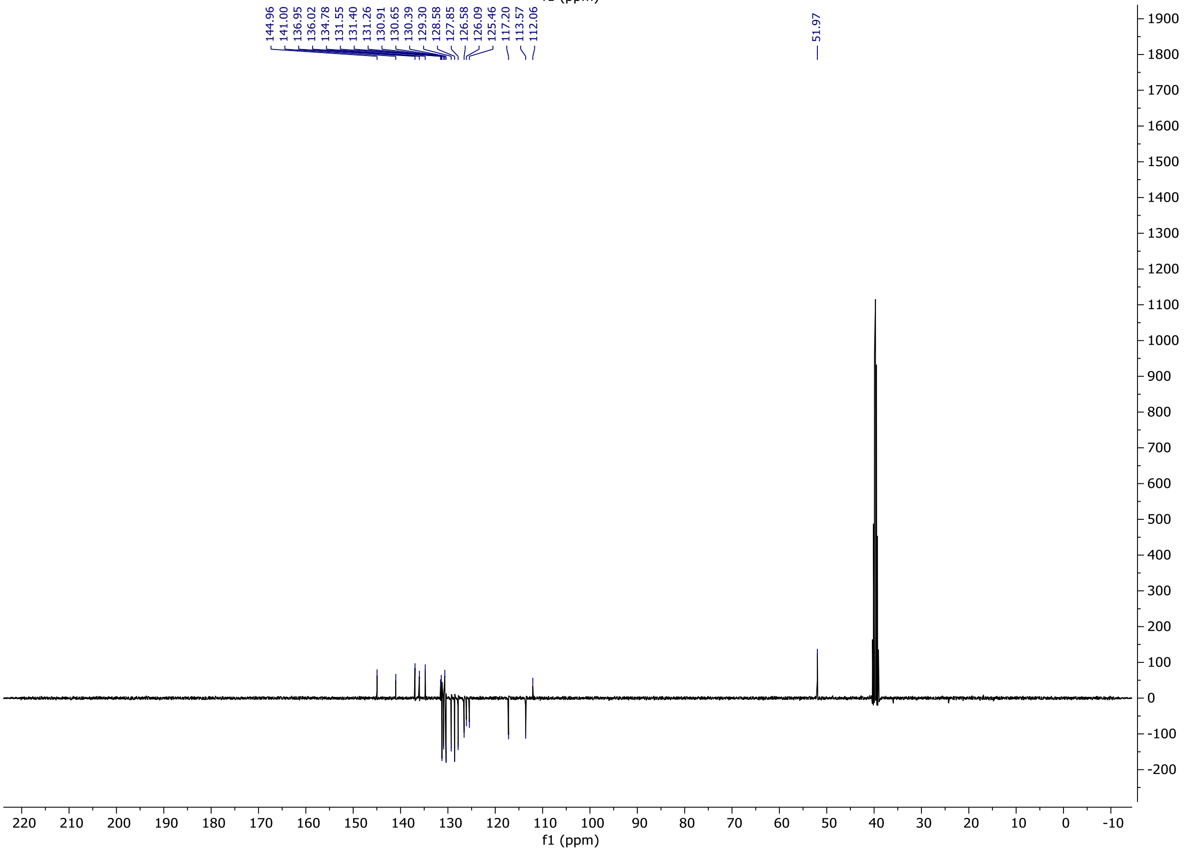

144.96
141.00
136.95
136.02
134.78
131.55
131.40
131.26
130.91
130.65
130.39
129.30
128.58
127.85
126.58
126.09
125.46
117.20
113.57
112.06
51.97
f1 (ppm)

13h

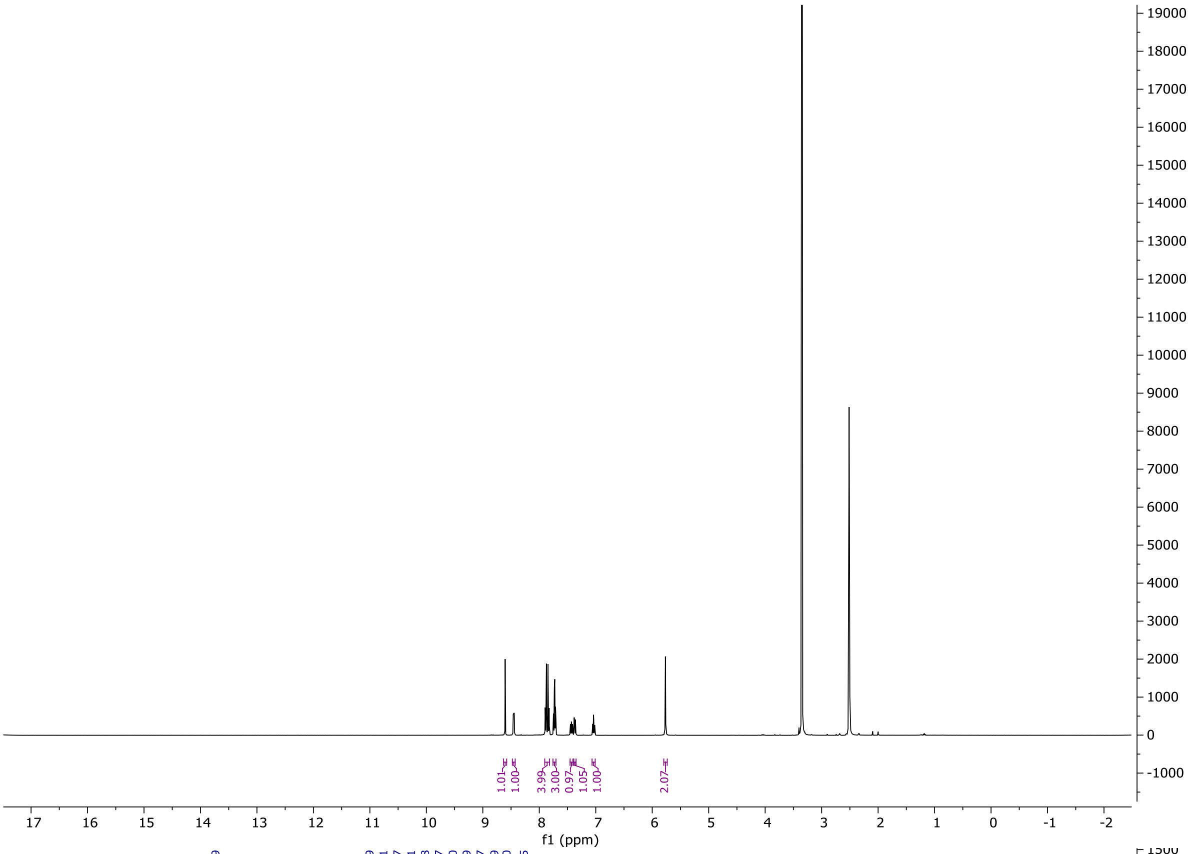

1.01
1.00
3.99
3.00
0.97
1.05
1.00
2.07
f1 (ppm)

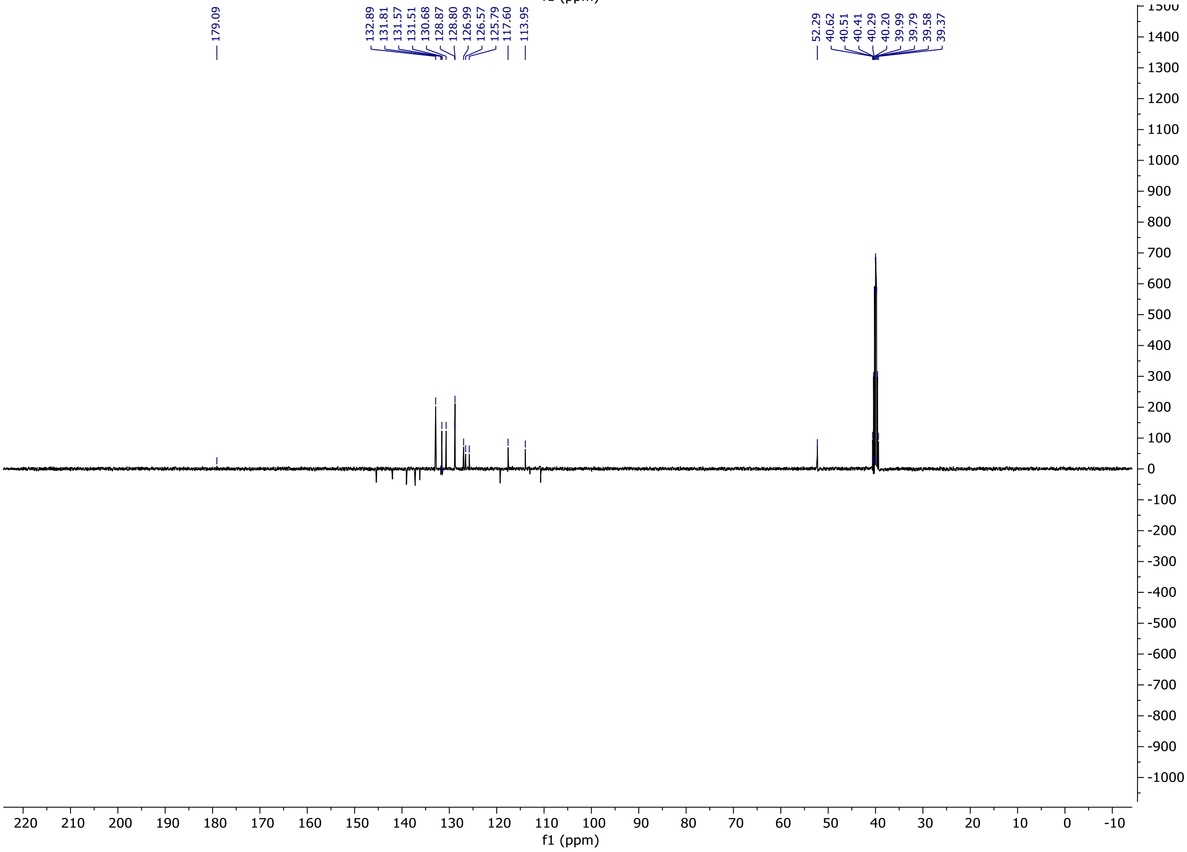

179.09
132.89
131.81
131.57
131.51
130.68
128.87
128.80
126.99
126.57
125.79
117.60
113.95
52.29
40.62
40.51
40.41
40.29
40.20
39.99
39.79
39.58
39.37
f1 (ppm)

13i

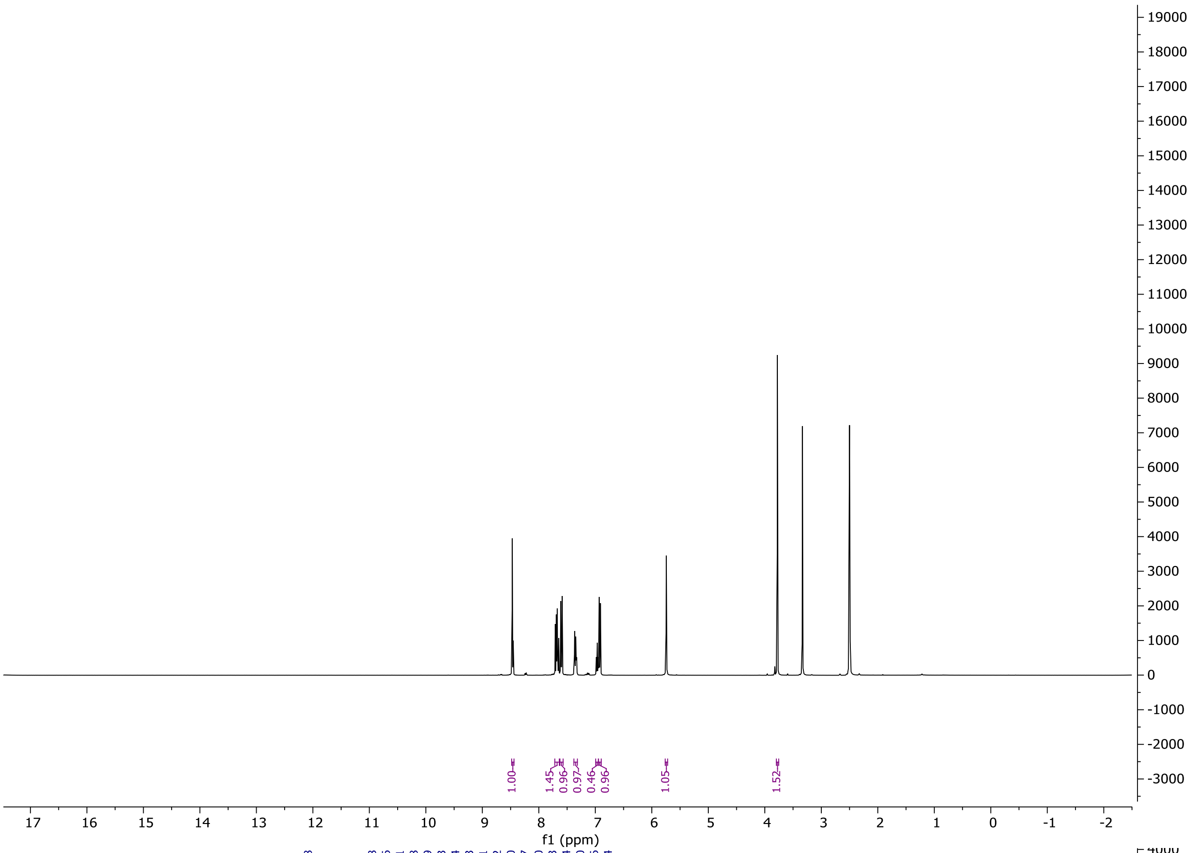

1.00
1.45
0.96
0.97
0.46
0.96
1.05
1.52
f1 (ppm)

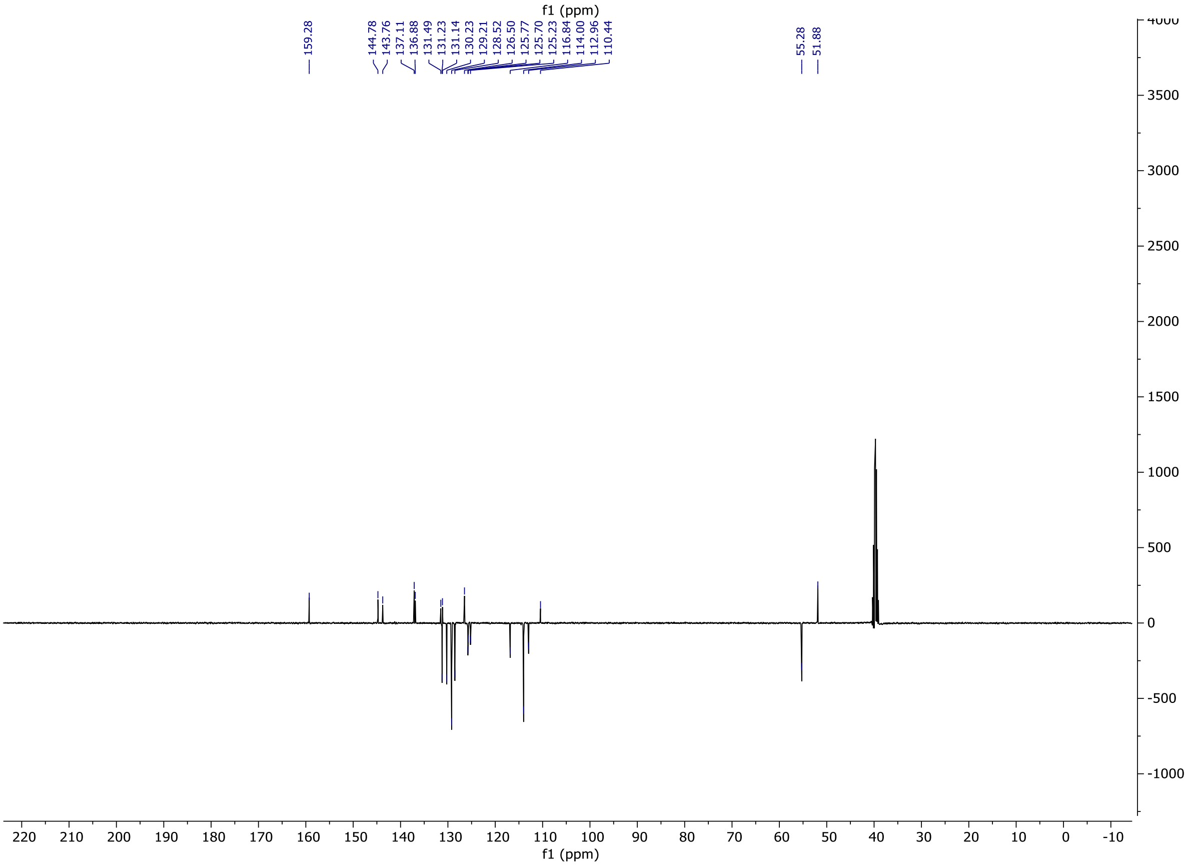

159.28
144.78
143.76
137.11
136.88
131.49
131.23
131.14
130.23
129.21
128.52
126.50
125.77
125.70
125.23
116.84
114.00
112.96
110.44
55.28
51.88
f1 (ppm)

14a

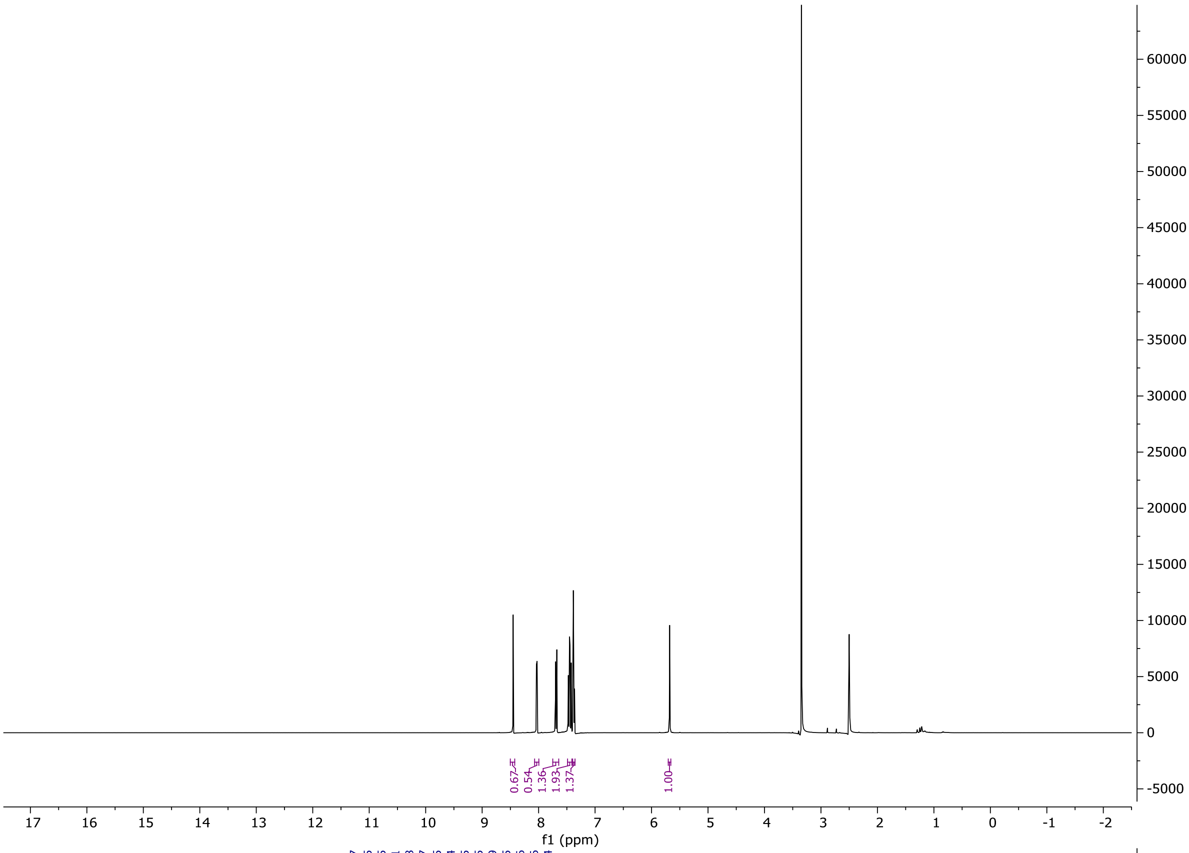
0.67
0.54
1.36
1.93
1.37
1.00
f1 (ppm)

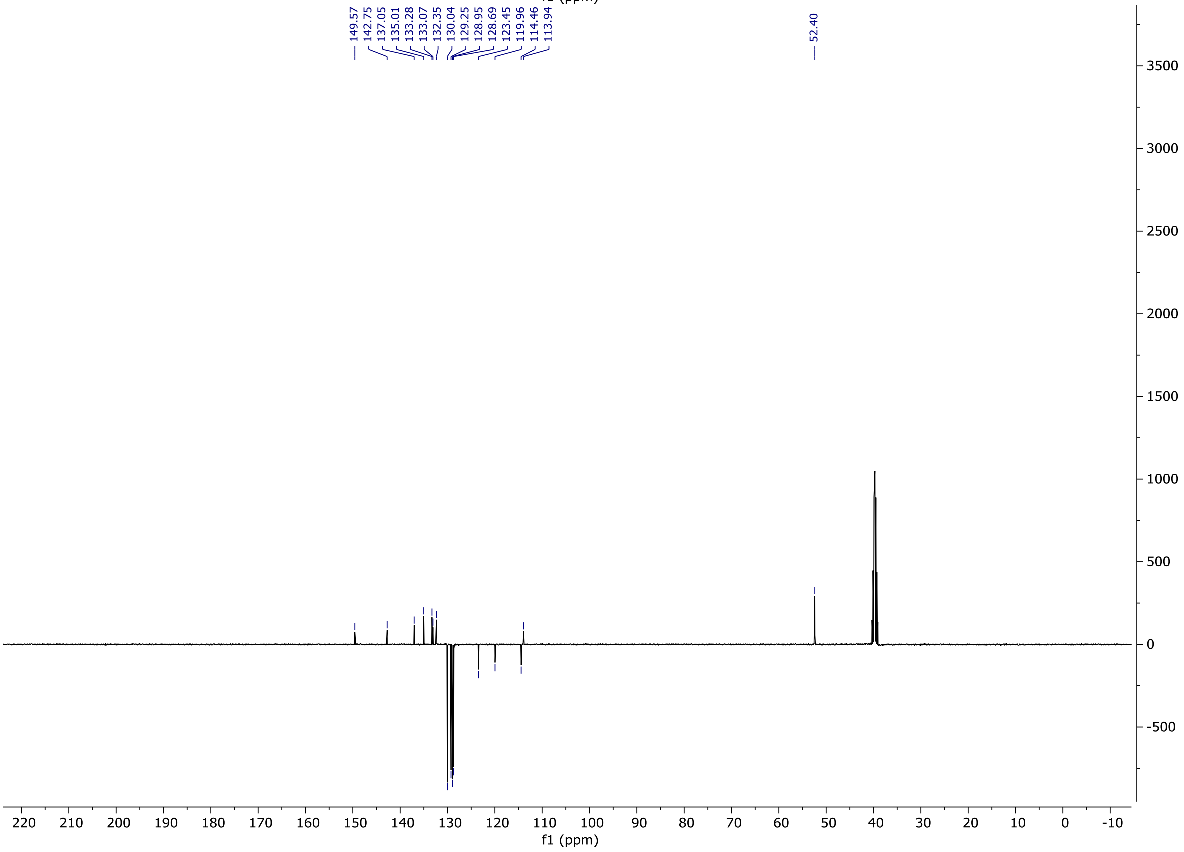
149.57
142.75
137.05
135.01
133.28
133.07
132.35
130.04
129.25
128.95
128.69
123.45
119.96
114.46
113.94
52.40
f1 (ppm)

14b

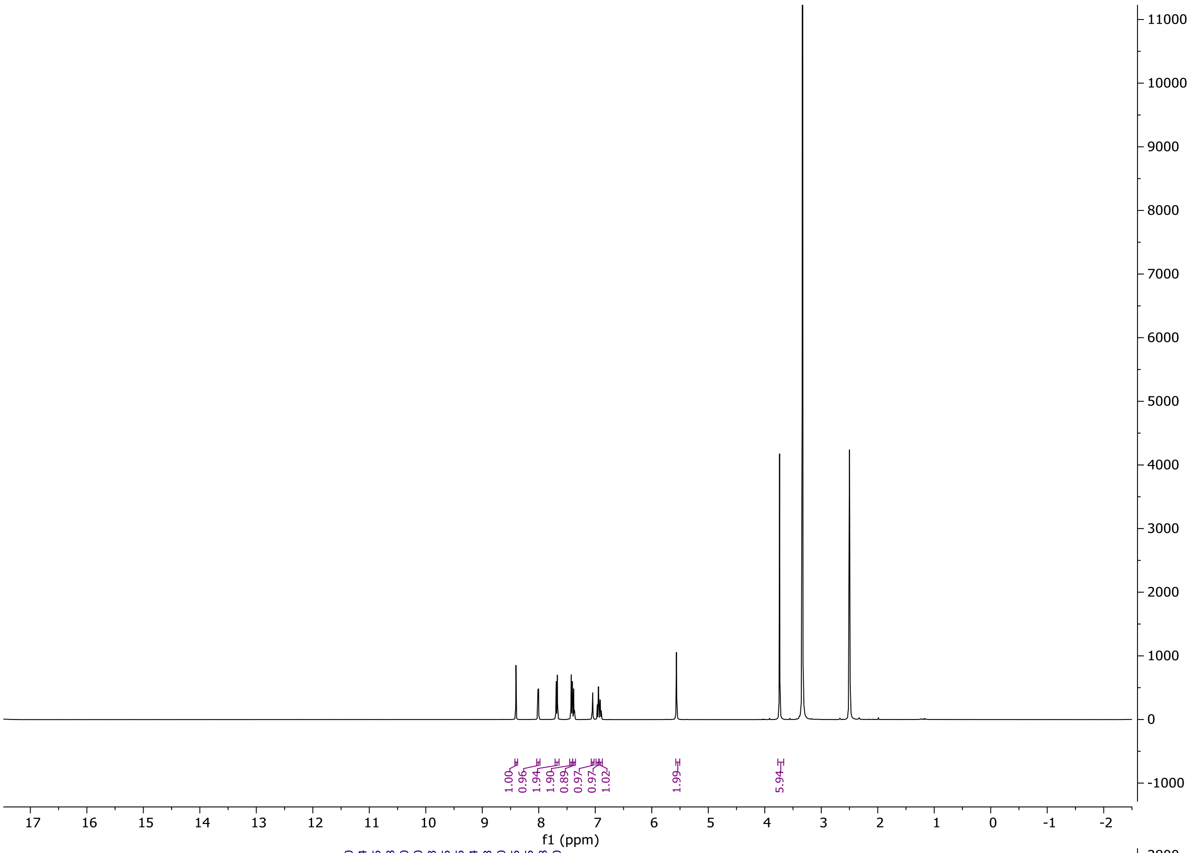
1.00
0.96
1.94
1.90
0.89
0.97
0.97
1.02
1.99
5.94
f1 (ppm)

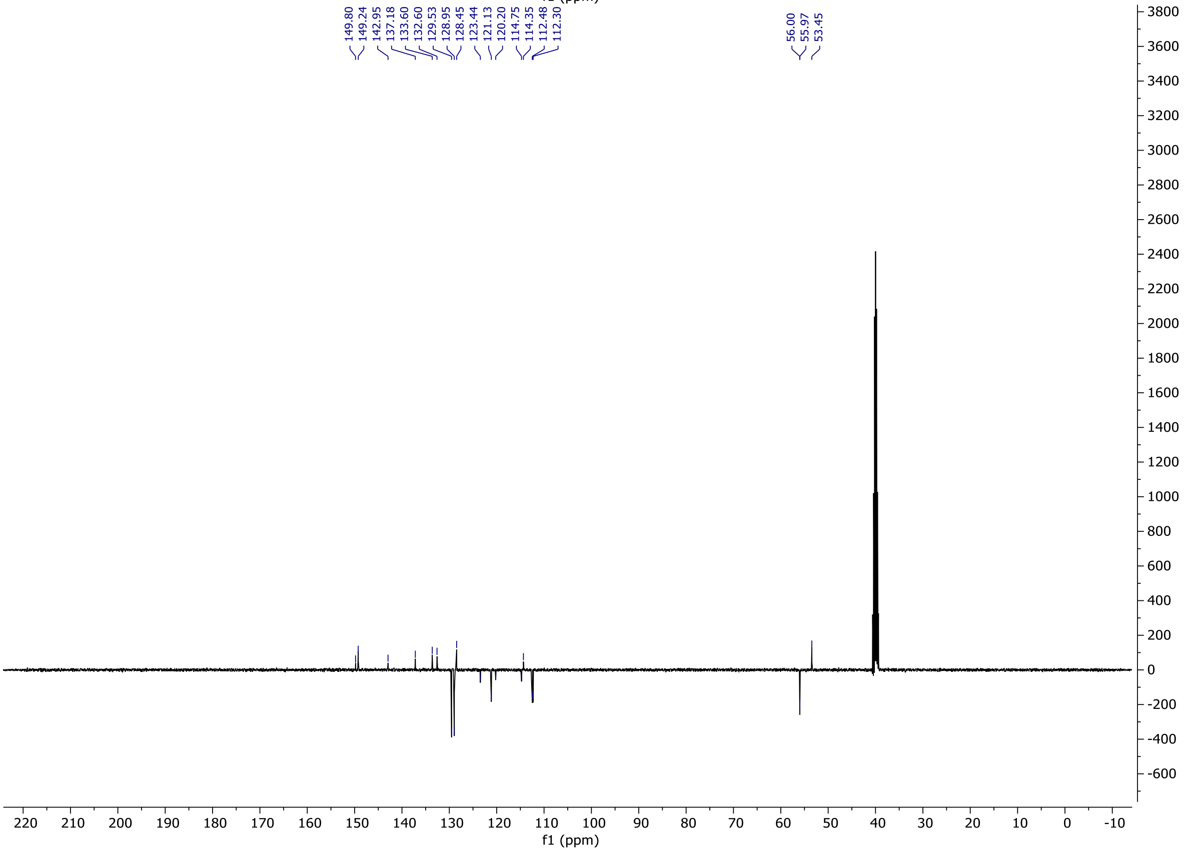
149.80
149.24
142.95
137.18
133.60
132.60
129.53
128.95
128.45
123.44
121.13
120.20
114.75
114.35
112.48
112.30
56.00
55.97
53.45
f1 (ppm)

14c

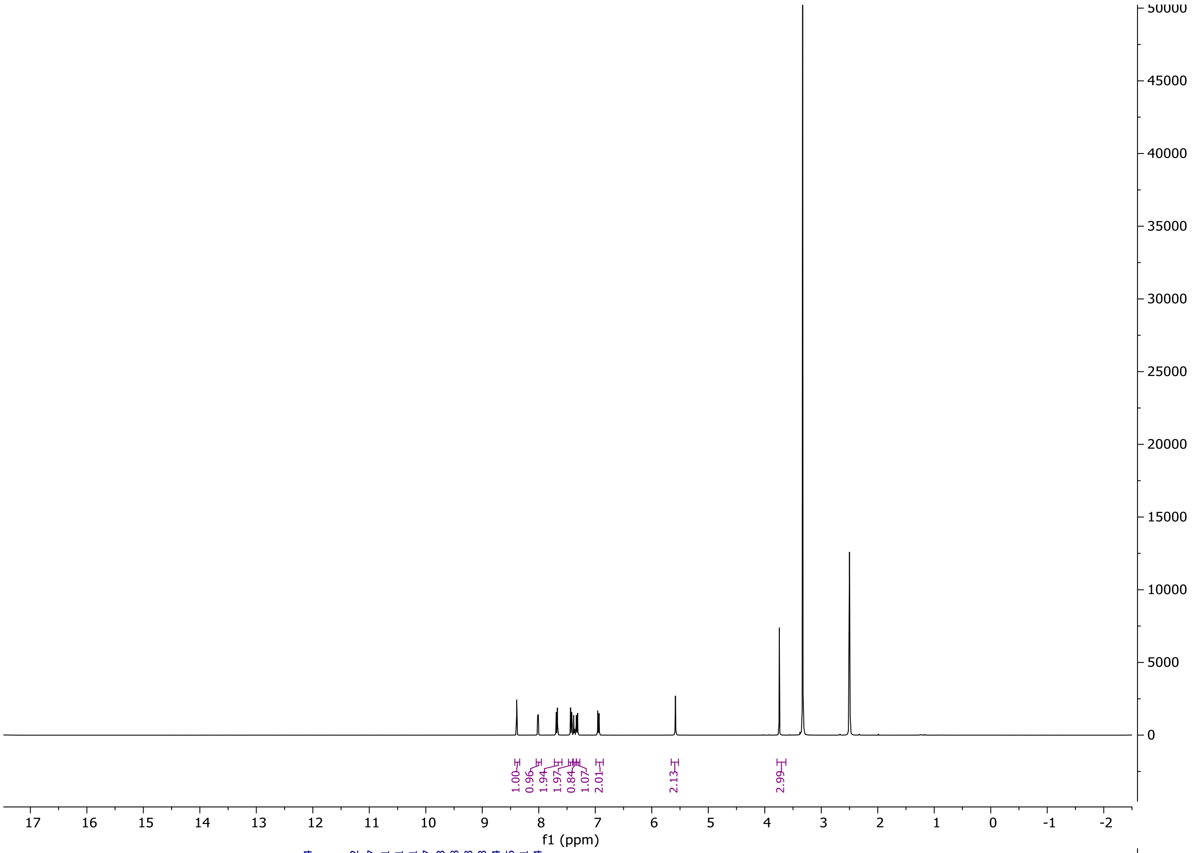

1.00
0.96
1.94
1.97
0.84
1.07
2.01
2.13
2.99
f1 (ppm)

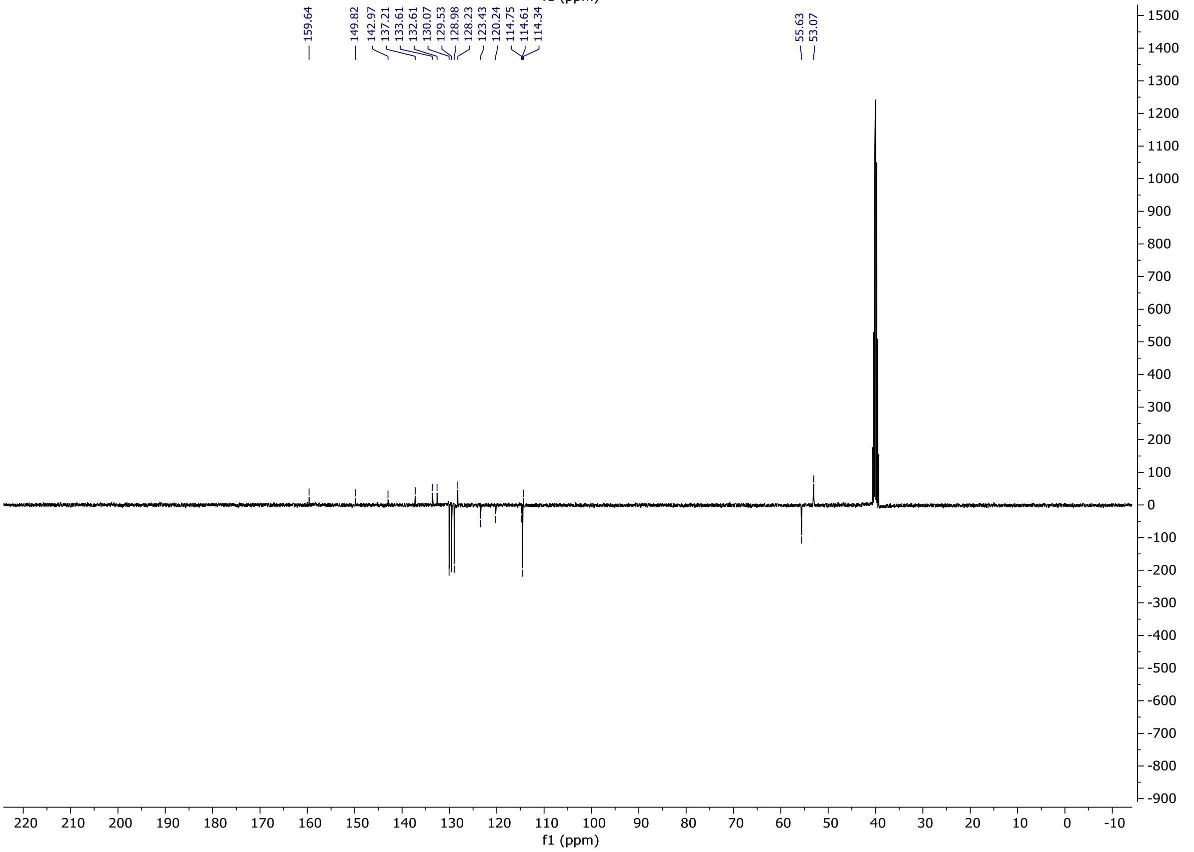

159.64
149.82
142.97
137.21
133.61
132.61
130.07
129.53
128.98
128.23
123.43
120.24
114.75
114.61
114.34
55.63
53.07
f1 (ppm)

14d

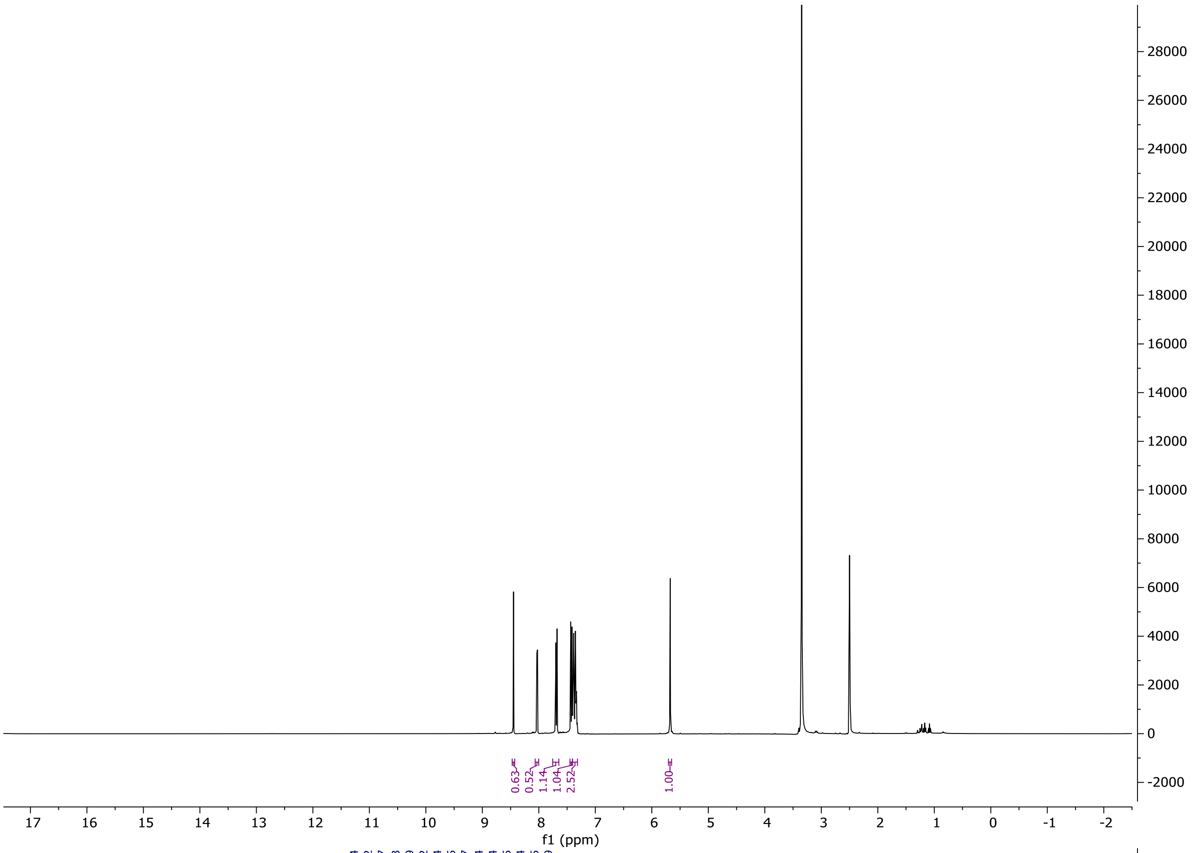
0.63
0.52
1.14
1.04
2.52
1.00
f1 (ppm)

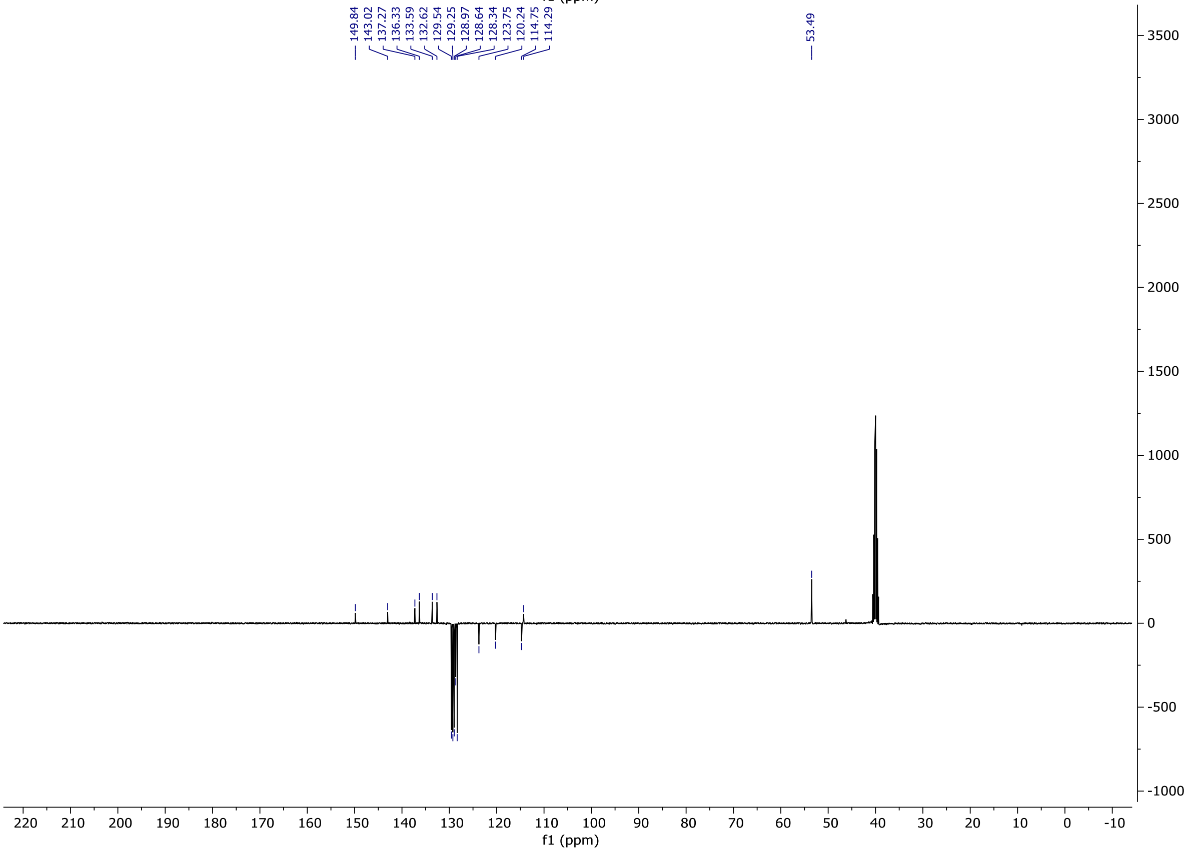
149.84
143.02
137.27
136.33
133.59
132.62
129.54
129.25
128.97
128.64
128.34
123.75
120.24
114.75
114.29
53.49
f1 (ppm)

14e

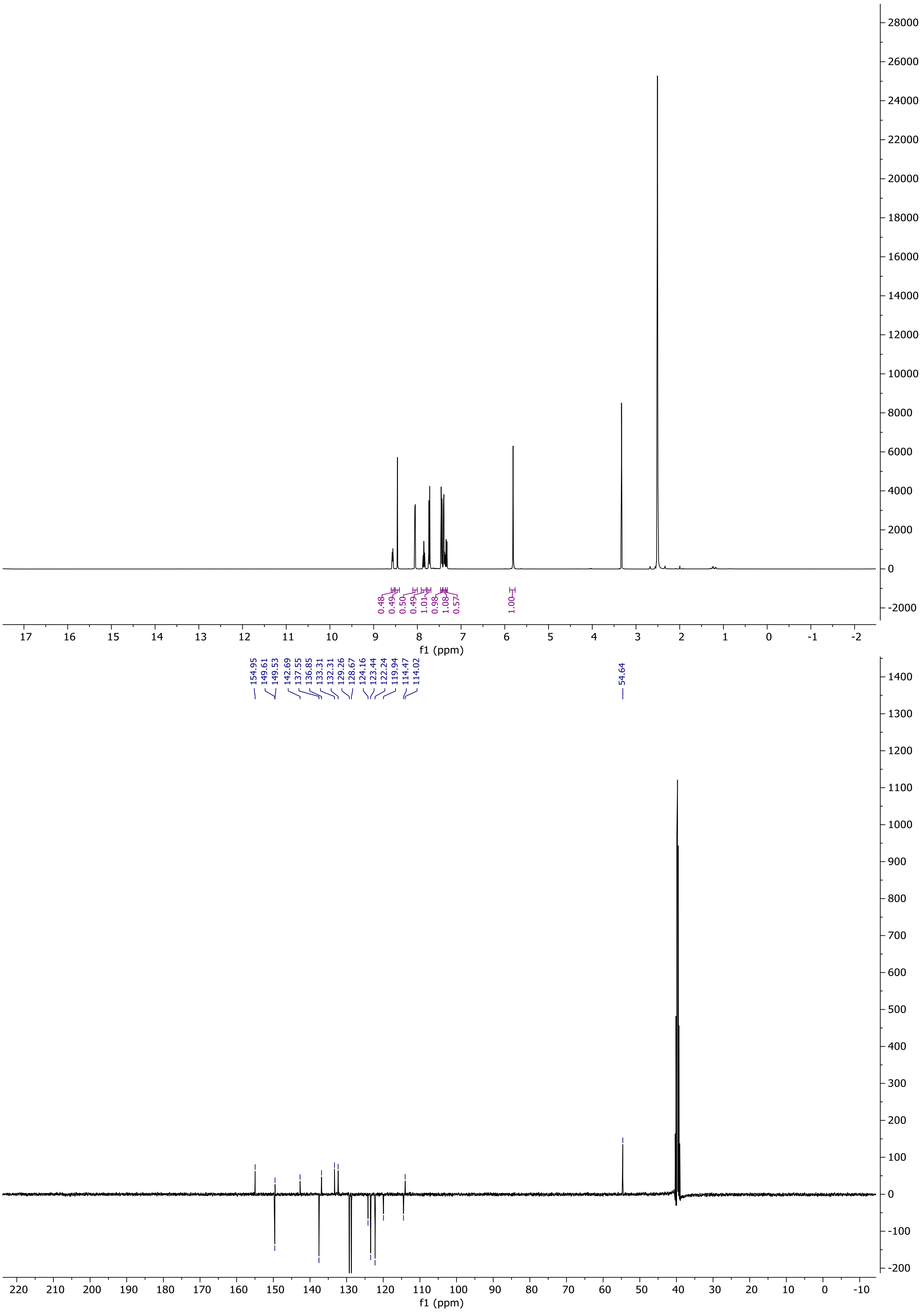
0.48
0.49
0.50
0.49
1.01
0.98
1.08
0.57
1.00
f1 (ppm)
154.95
149.61
149.53
142.69
137.55
136.85
133.31
132.31
129.26
128.67
124.16
123.44
122.24
119.94
114.47
114.02
54.64
f1 (ppm)

14f

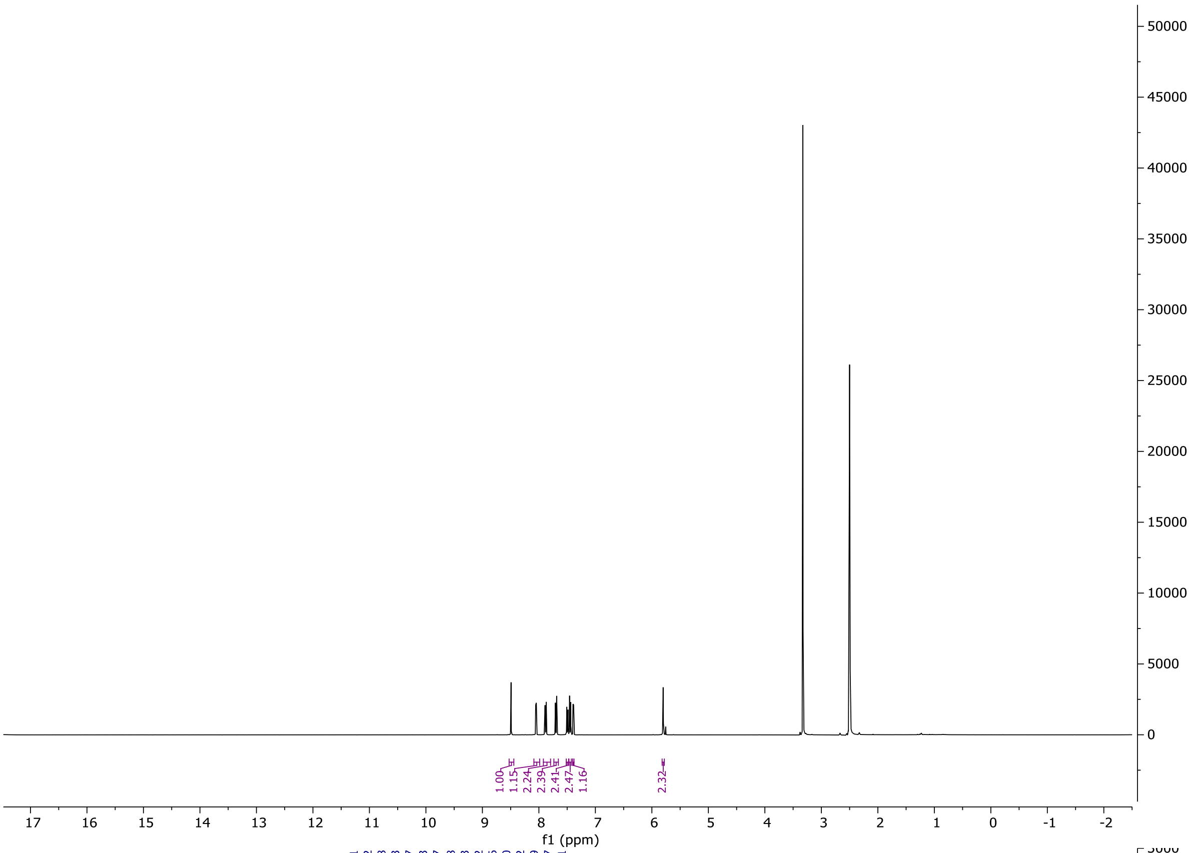
1.00
1.15
2.24
2.39
2.41
2.47
1.16
2.32
f1 (ppm)

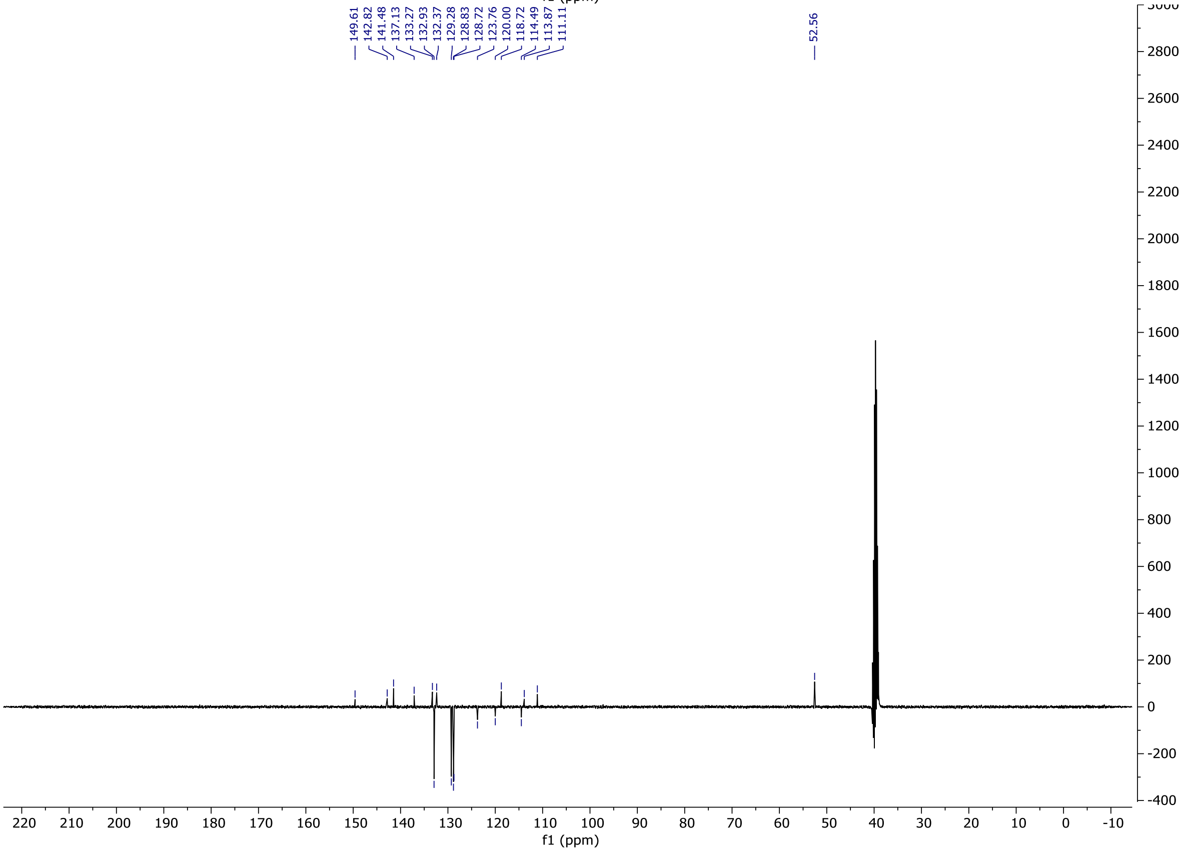
149.61
142.82
141.48
137.13
133.27
132.93
132.37
129.28
128.83
128.72
123.76
120.00
118.72
114.49
113.87
111.11
52.56
f1 (ppm)

15a

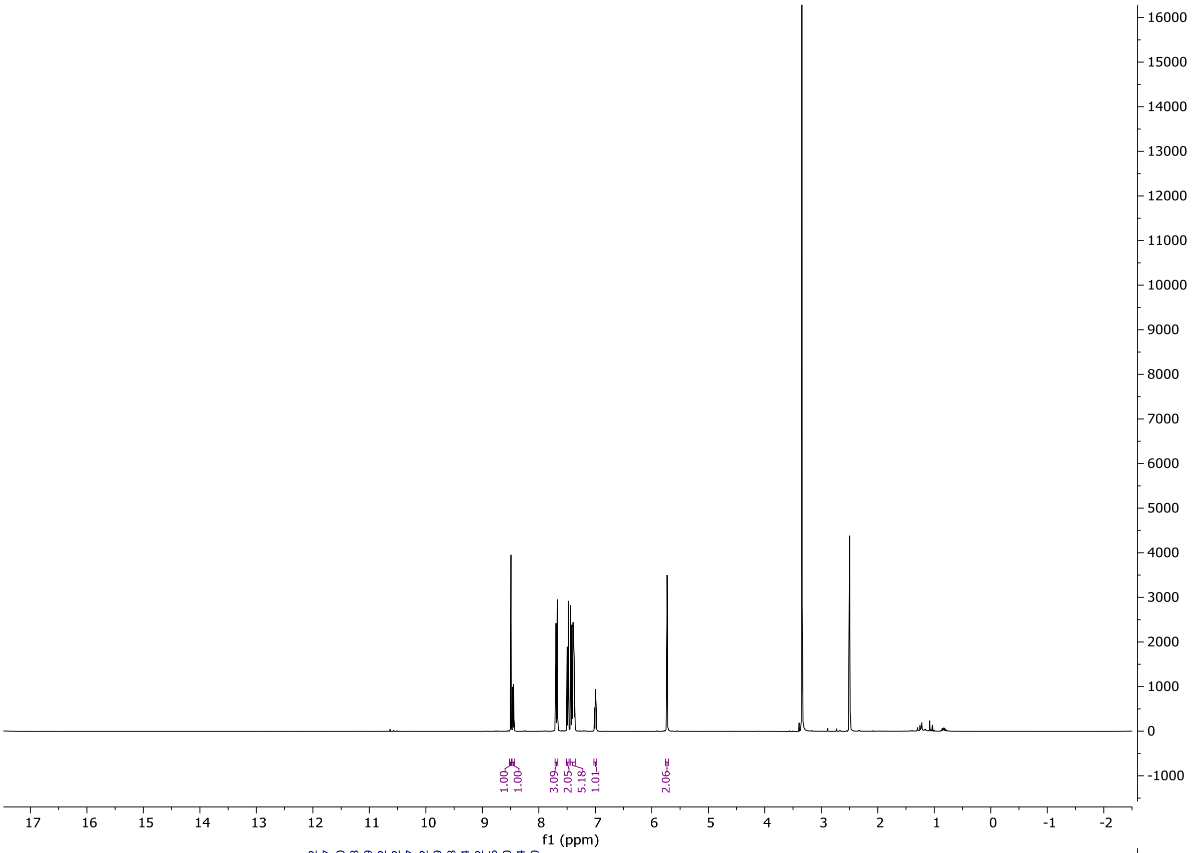
1.00
1.00
3.09
2.05
5.18
1.01
2.06
f1 (ppm)

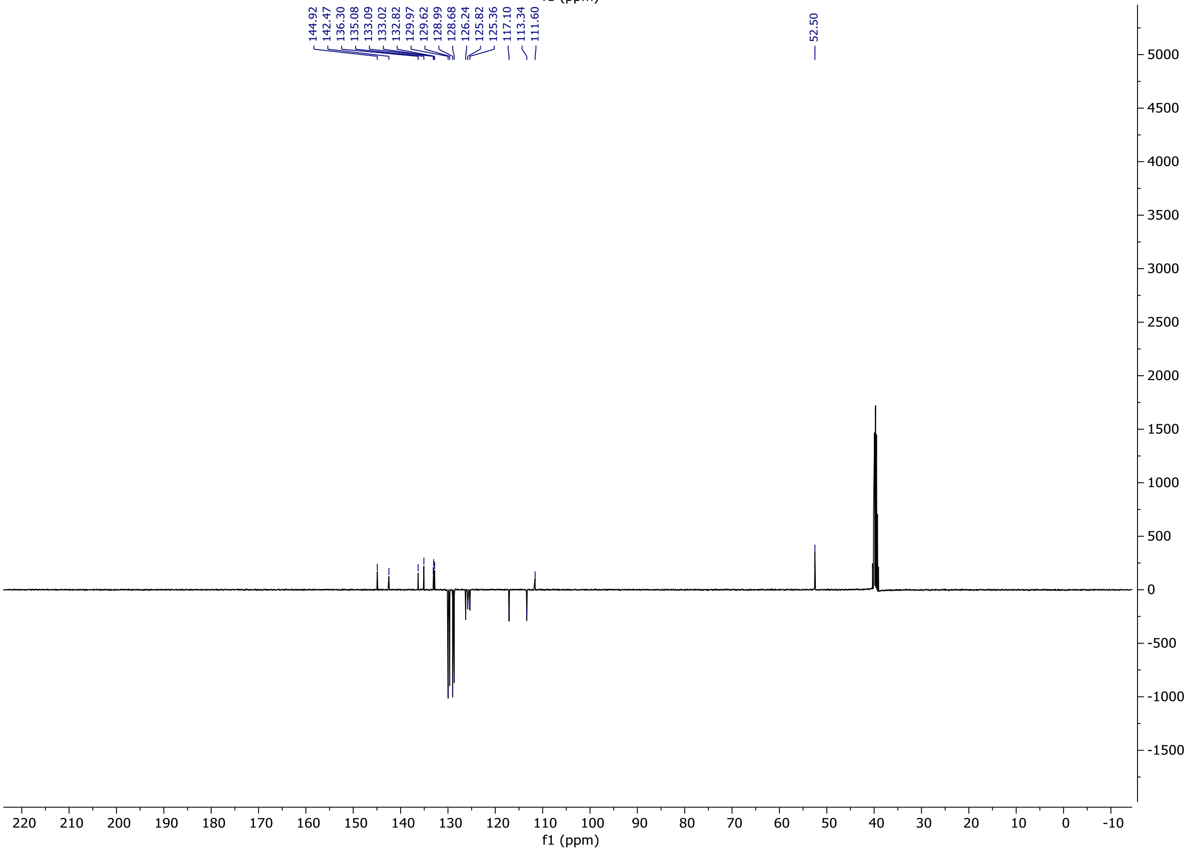
144.92
142.47
136.30
135.08
133.09
133.02
132.82
129.97
129.62
128.99
128.68
126.24
125.82
125.36
117.10
113.34
111.60
52.50
f1 (ppm)

15b

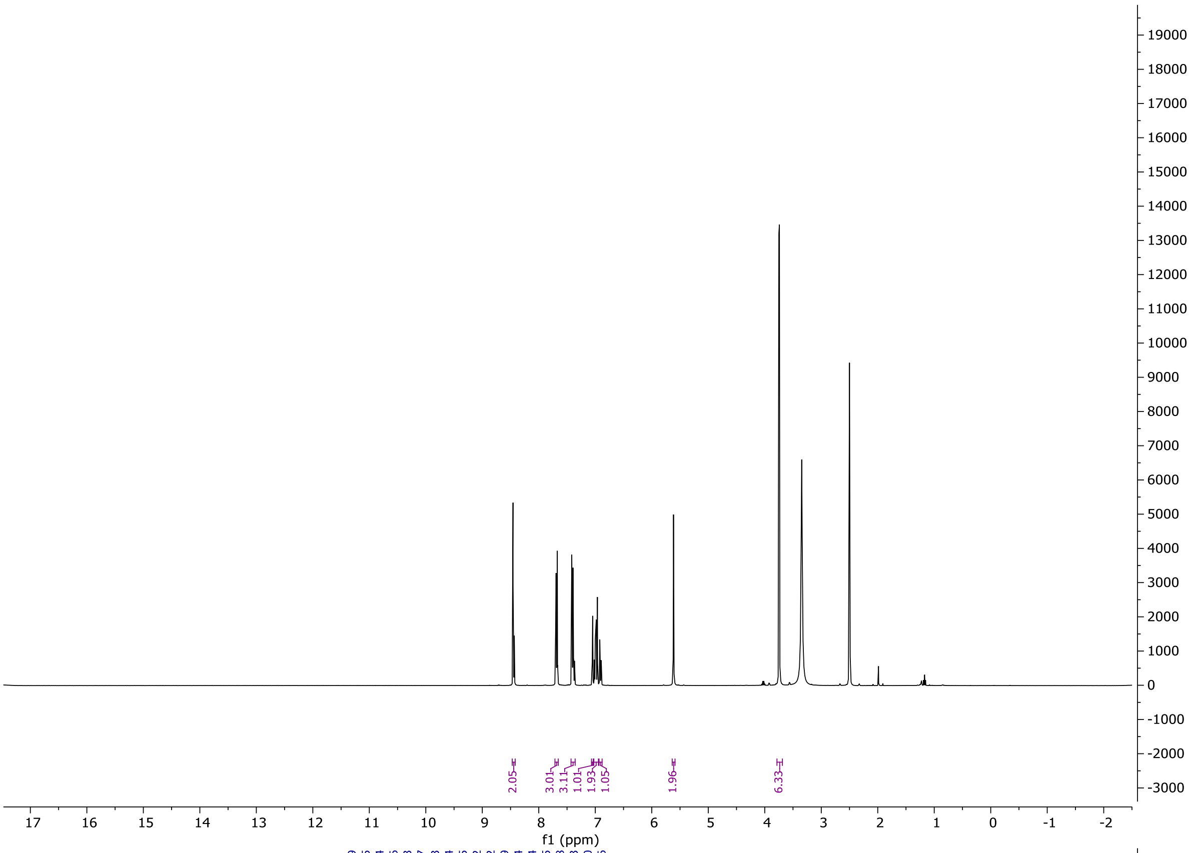

2.05
3.01
3.11
1.01
1.93
1.05
1.96
6.33
f1 (ppm)

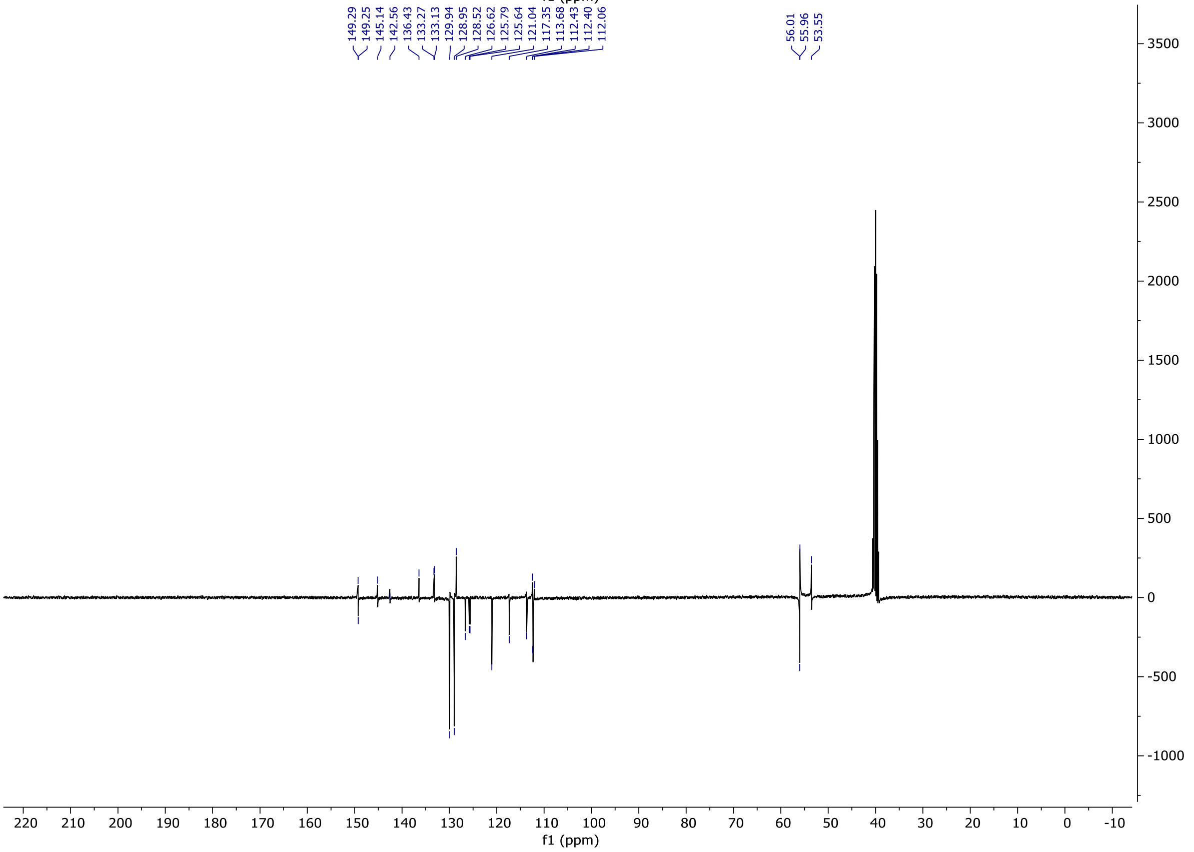

149.29
149.25
145.14
142.56
136.43
133.27
133.13
129.94
128.95
128.52
126.62
125.79
125.64
121.04
117.35
113.68
112.43
112.40
112.06
56.01
55.96
53.55
f1 (ppm)

15c

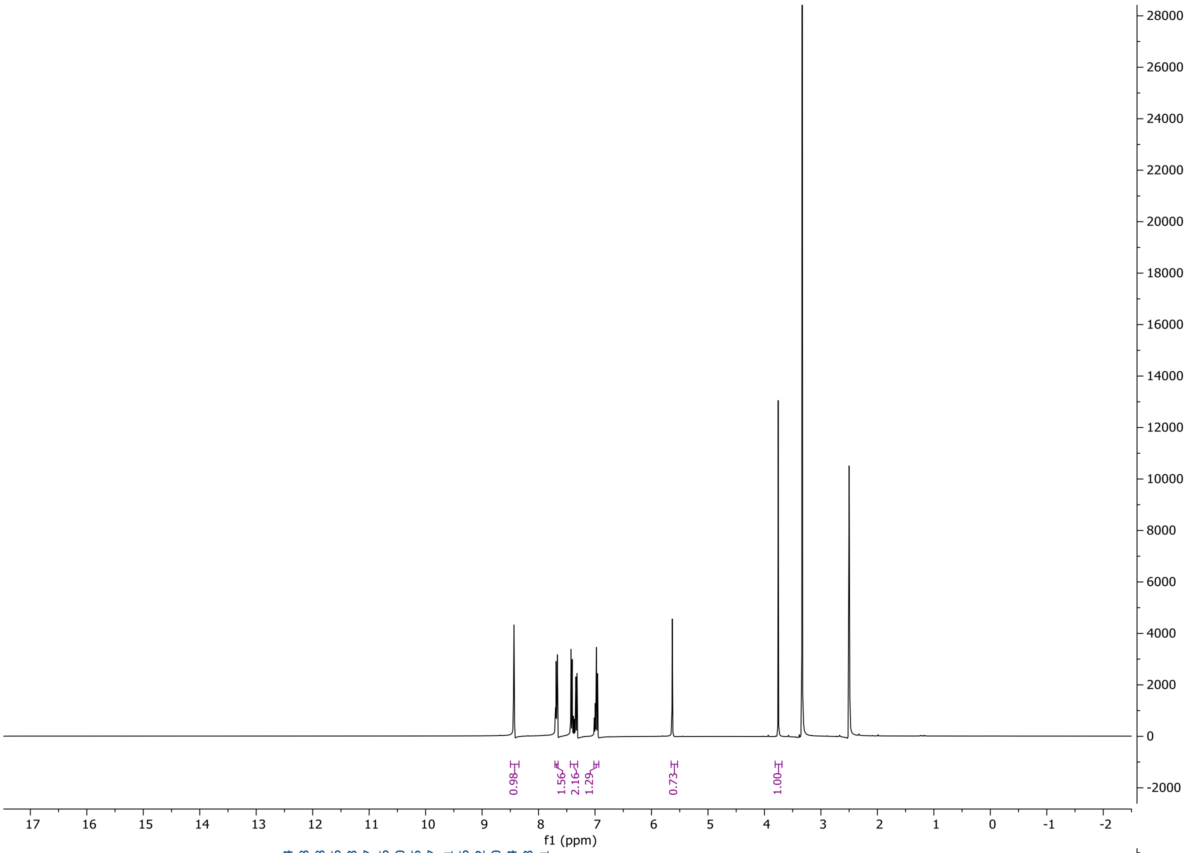
0.98
1.56
2.16
1.29
0.73
1.00
f1 (ppm)

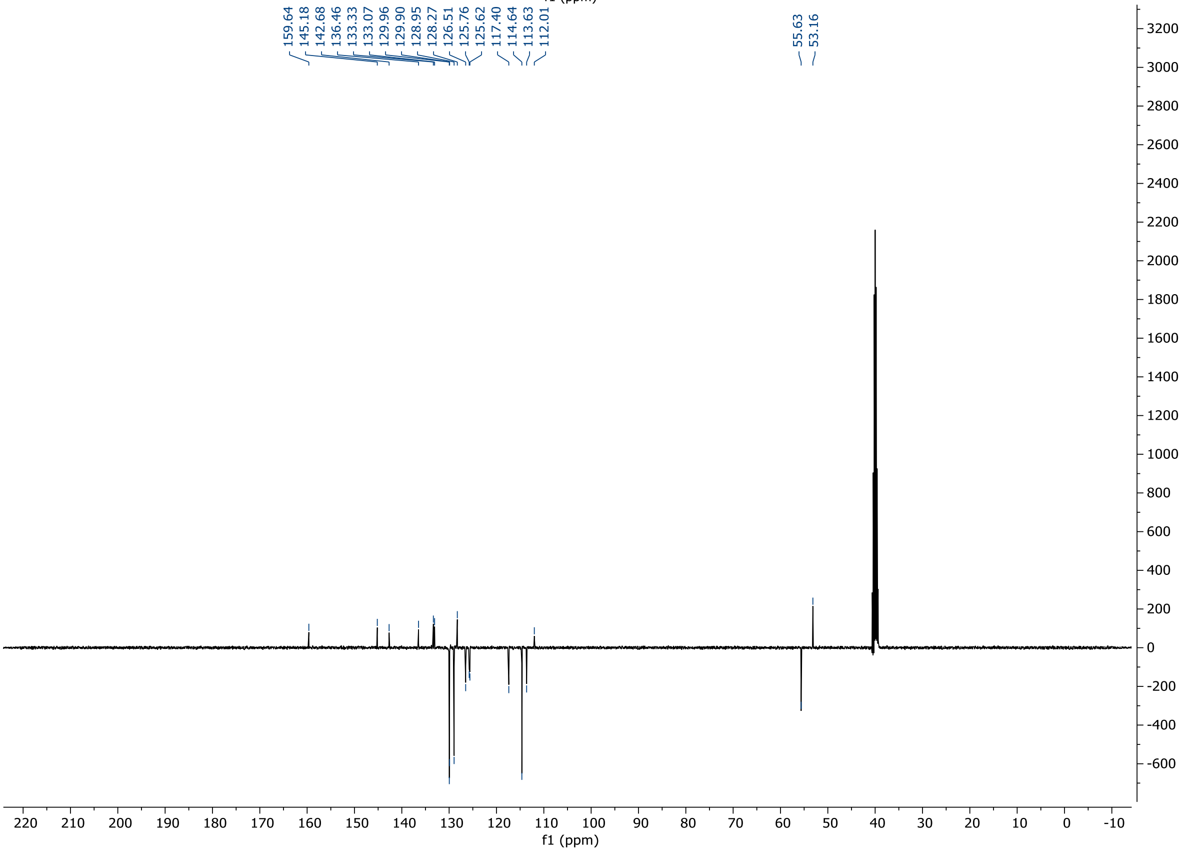
159.64
145.18
142.68
136.46
133.33
133.07
129.96
129.90
128.95
128.27
126.51
125.76
125.62
117.40
114.64
113.63
112.01
55.63
53.16
f1 (ppm)

15d

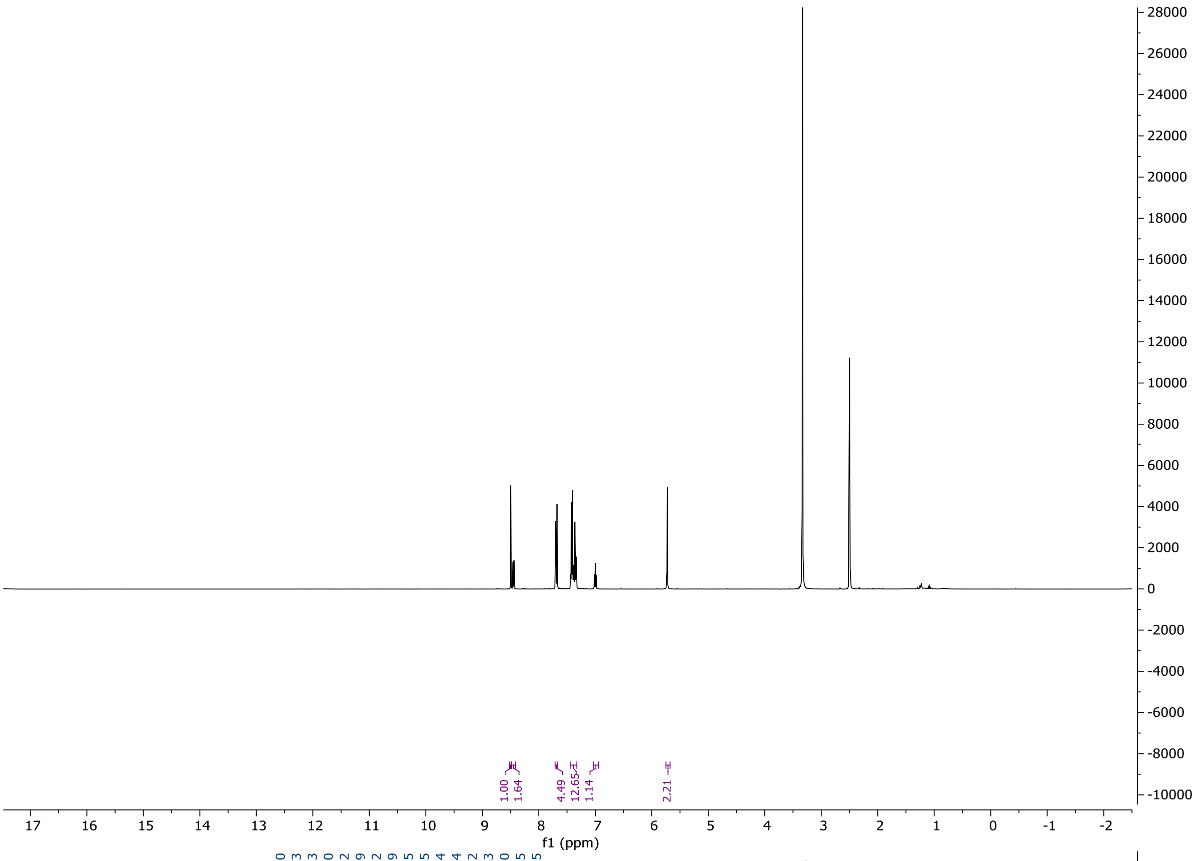
1.00
1.64
4.49
12.65
1.14
2.21
f1 (ppm)

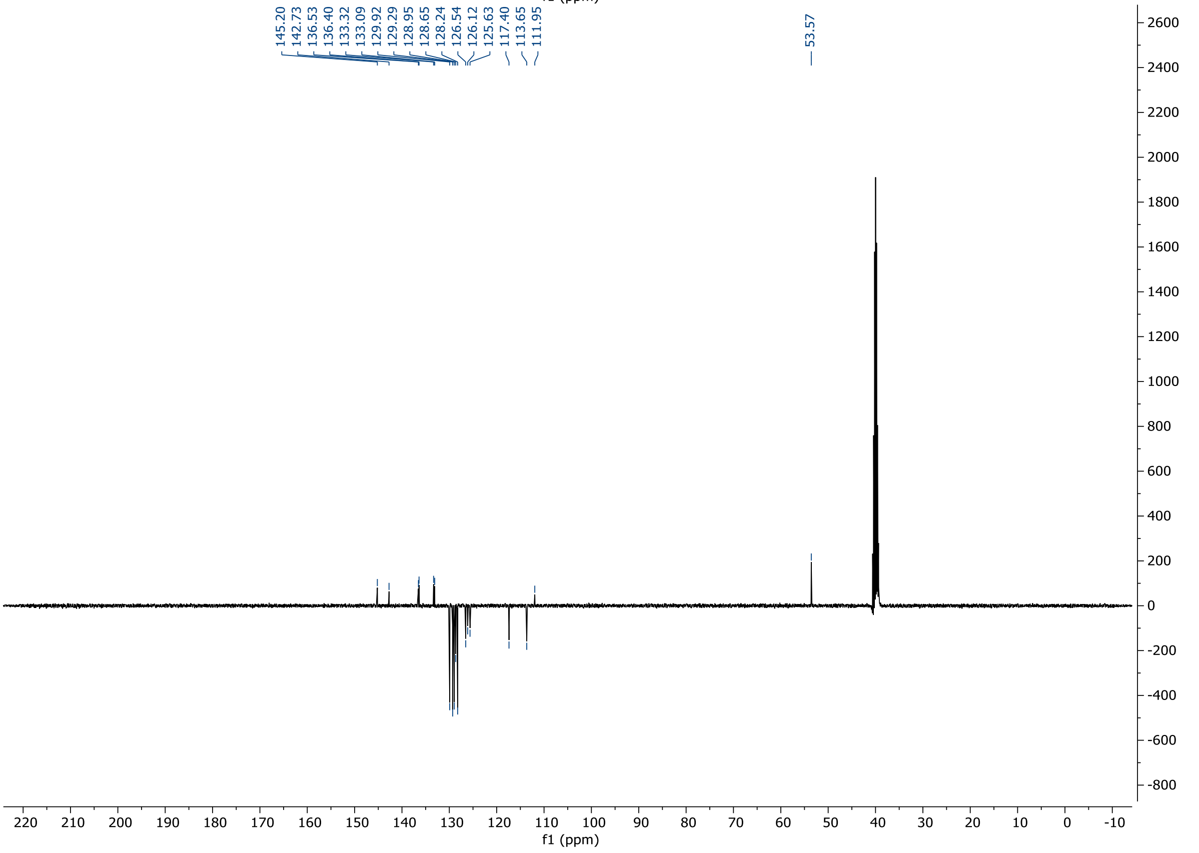
145.20
142.73
136.53
136.40
133.32
133.09
129.92
129.29
128.95
128.65
128.24
126.54
126.12
125.63
117.40
113.65
111.95
53.57
f1 (ppm)

15e

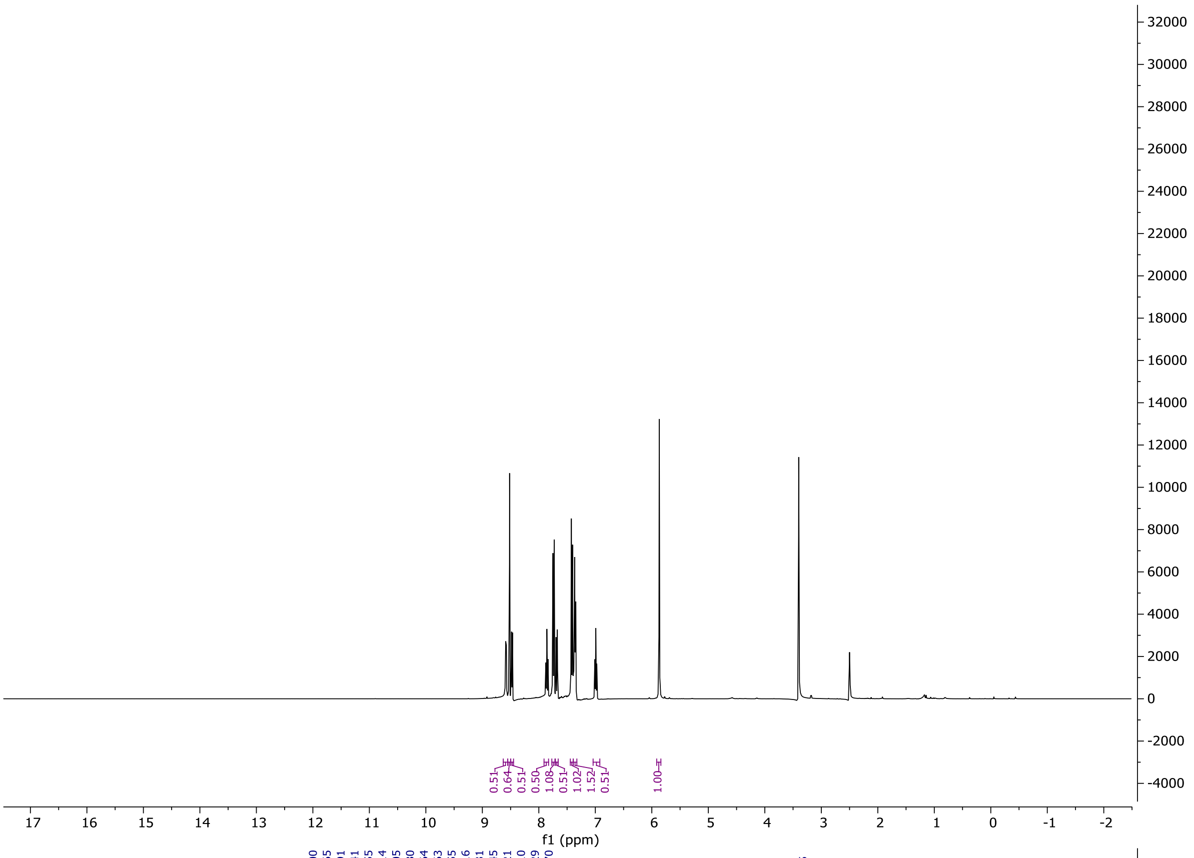
0.51
0.64
0.51
0.50
1.08
0.51
1.02
1.52
0.51
1.00
f1 (ppm)

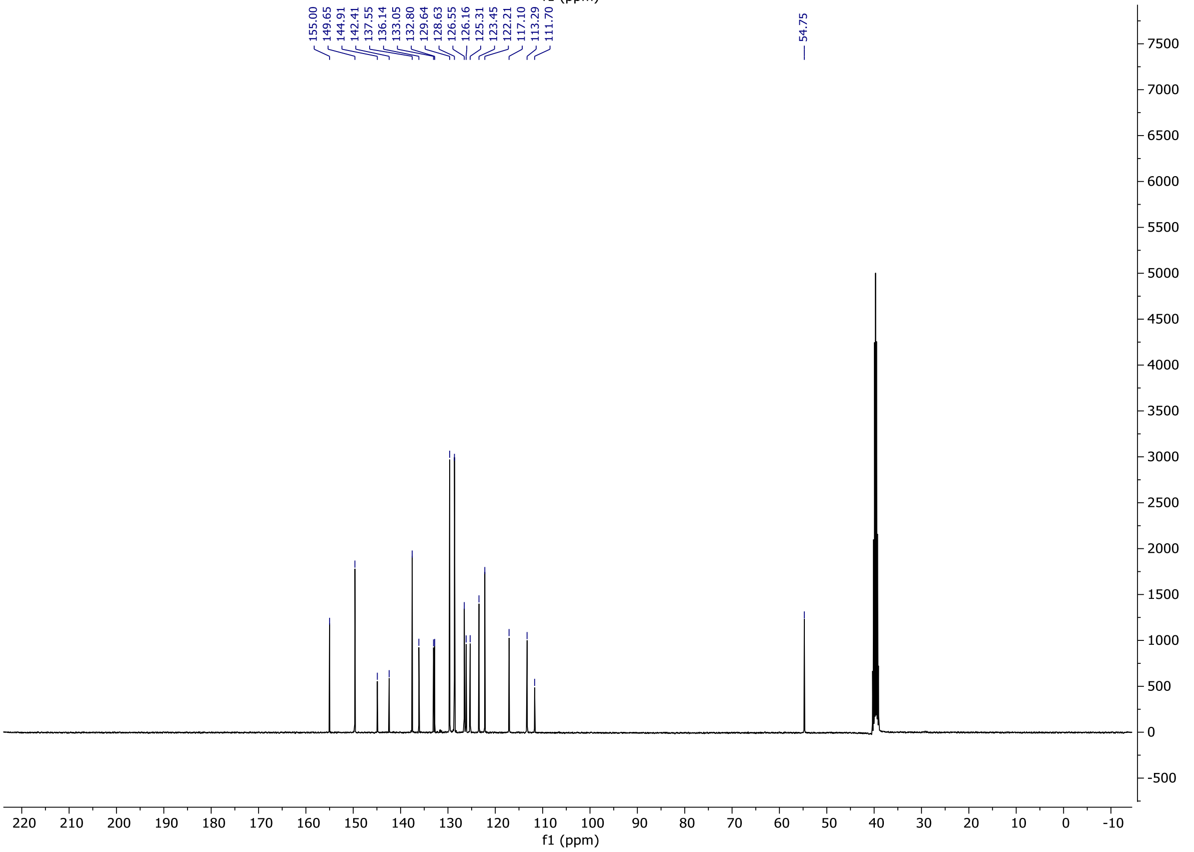
155.00
149.65
144.91
142.41
137.55
136.14
133.05
132.80
129.64
128.63
126.55
126.16
125.31
123.45
122.21
117.10
113.29
111.70
54.75
f1 (ppm)

15f

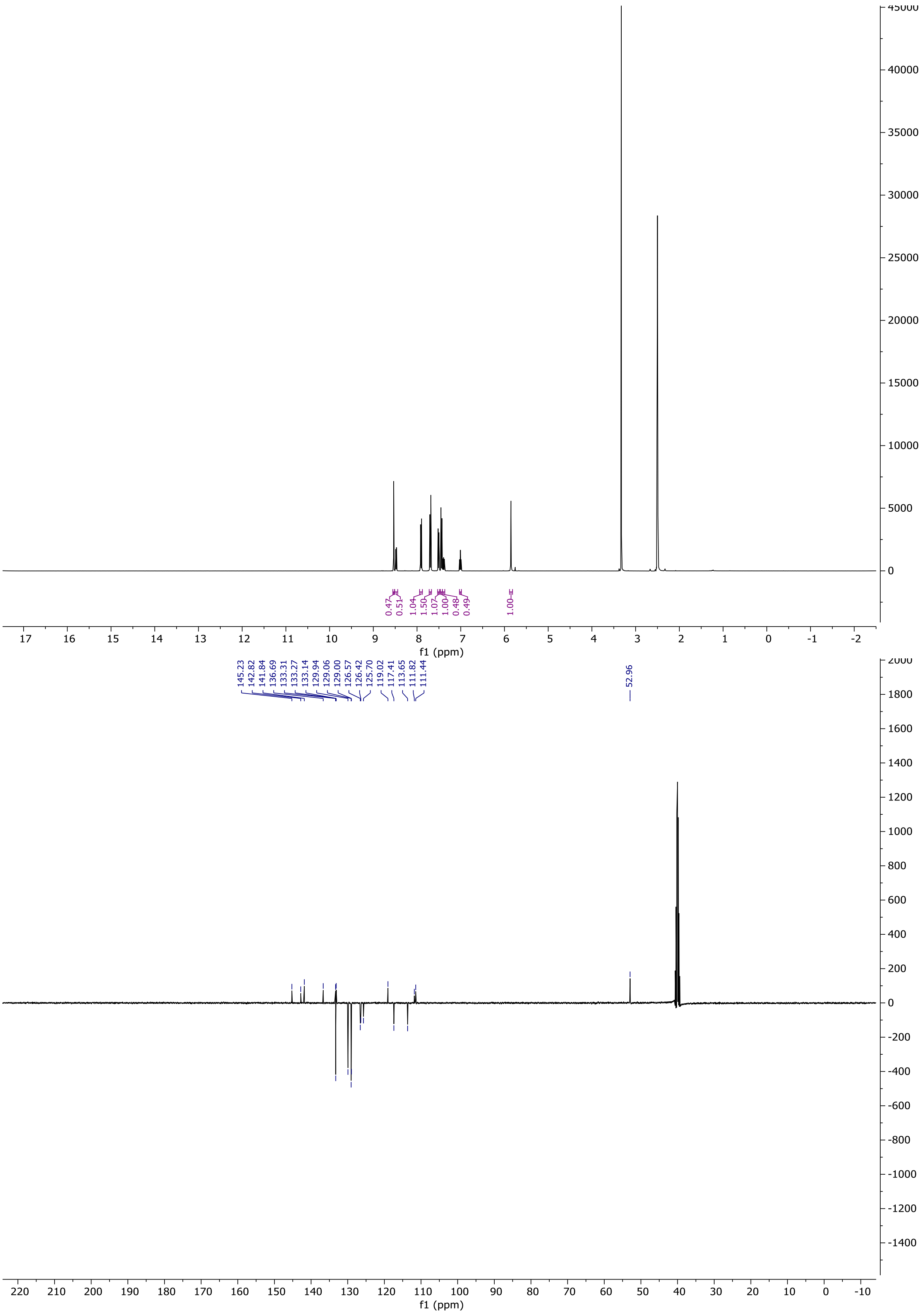
0.47
0.51
1.04
1.50
1.07
1.00
0.48
0.49
1.00
f1 (ppm)
145.23
142.82
141.84
136.69
133.31
133.27
133.14
129.94
129.06
129.00
126.57
126.42
125.70
119.02
117.41
113.65
111.82
111.44
52.96
f1 (ppm)

15g

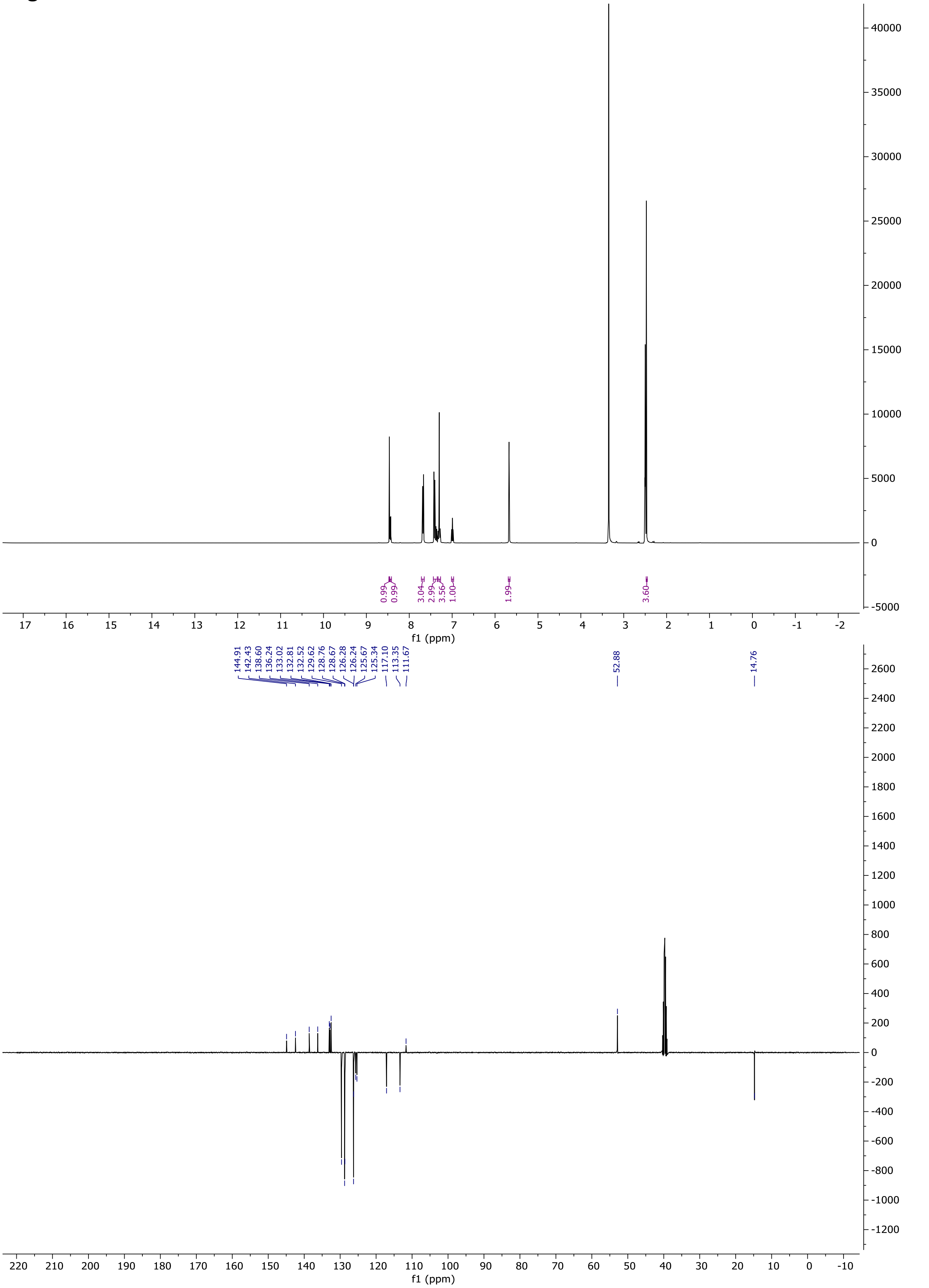

0.99
0.99
3.04
2.99
3.56
1.00
1.99
3.60
f1 (ppm)
144.91
142.43
138.60
136.24
133.02
132.81
132.52
129.62
128.76
128.67
126.28
126.24
125.67
125.34
117.10
113.35
111.67
52.88
14.76
f1 (ppm)

15h

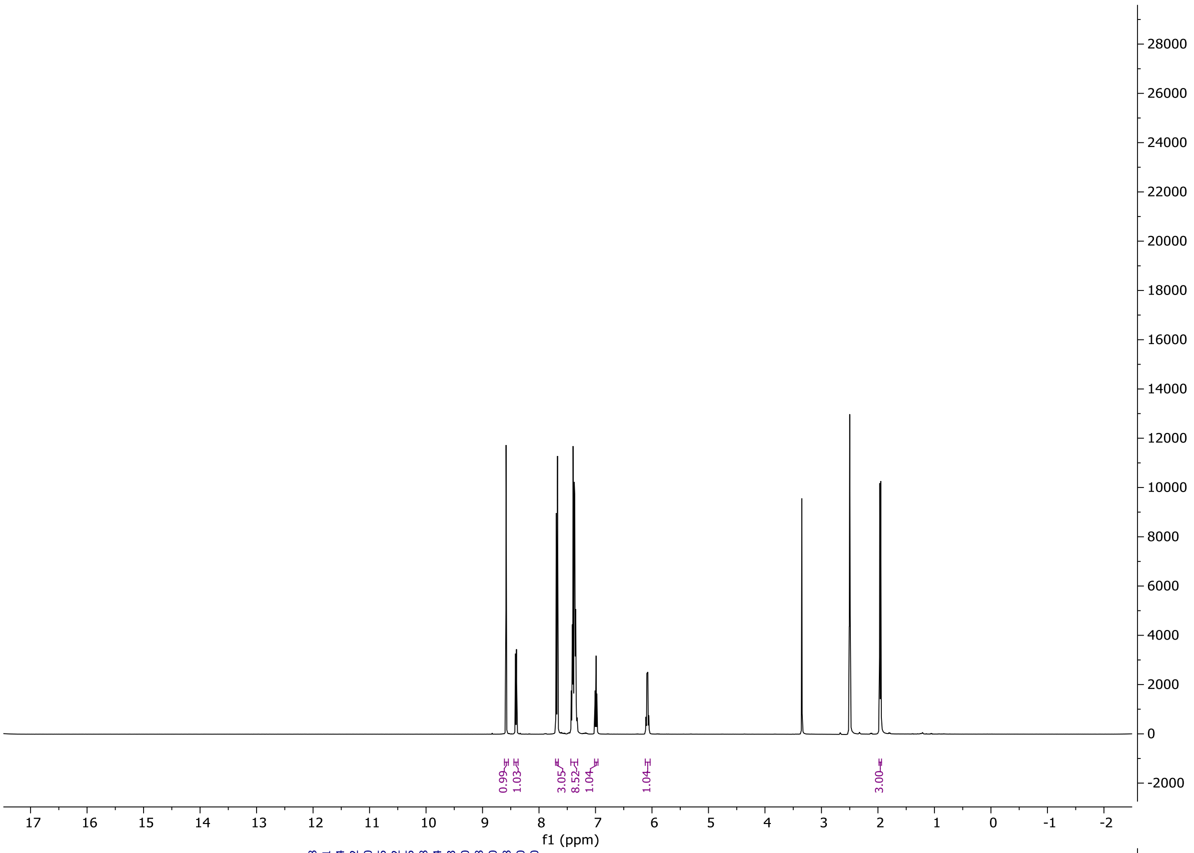
0.99
1.03
3.05
8.52
1.04
1.04
3.00
f1 (ppm)

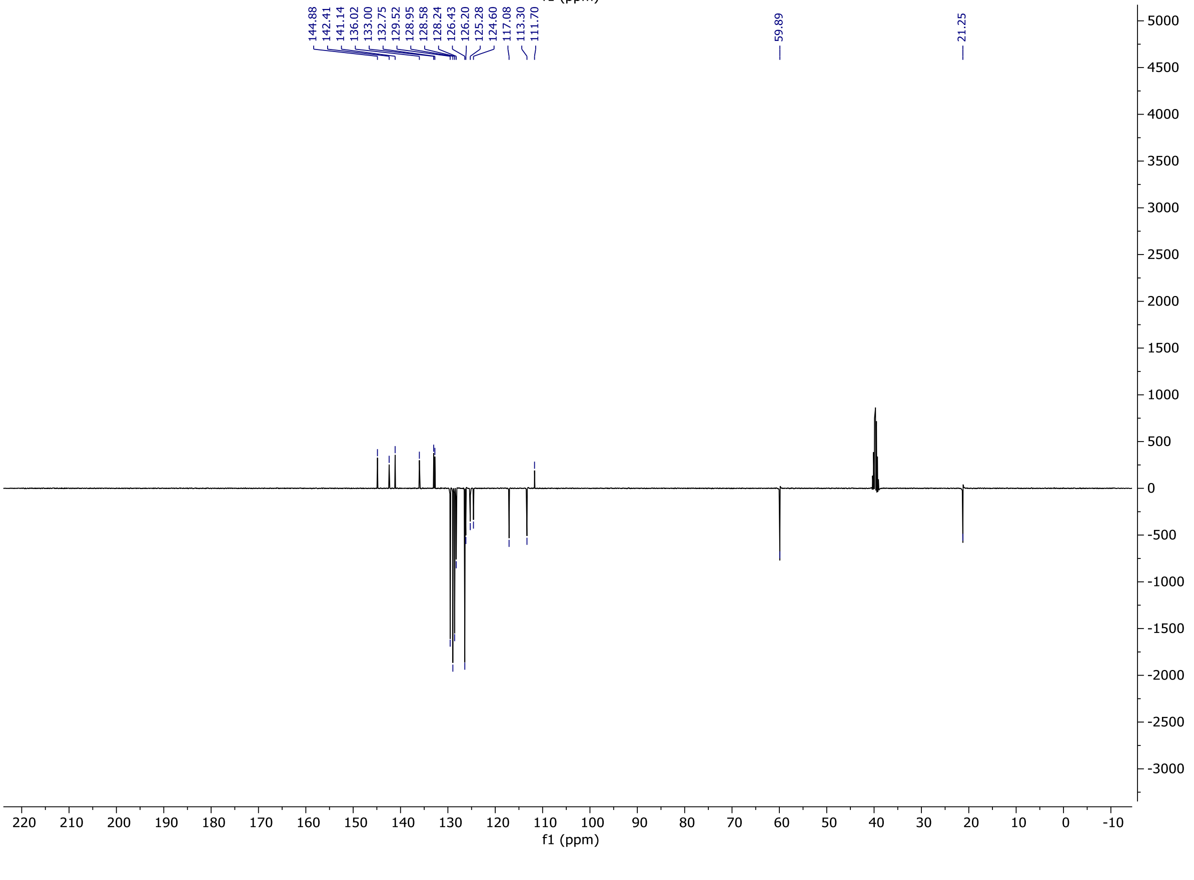
144.88
142.41
141.14
136.02
133.00
132.75
129.52
128.95
128.58
128.24
126.43
126.20
125.28
124.60
117.08
113.30
111.70
59.89
21.25
f1 (ppm)

15i

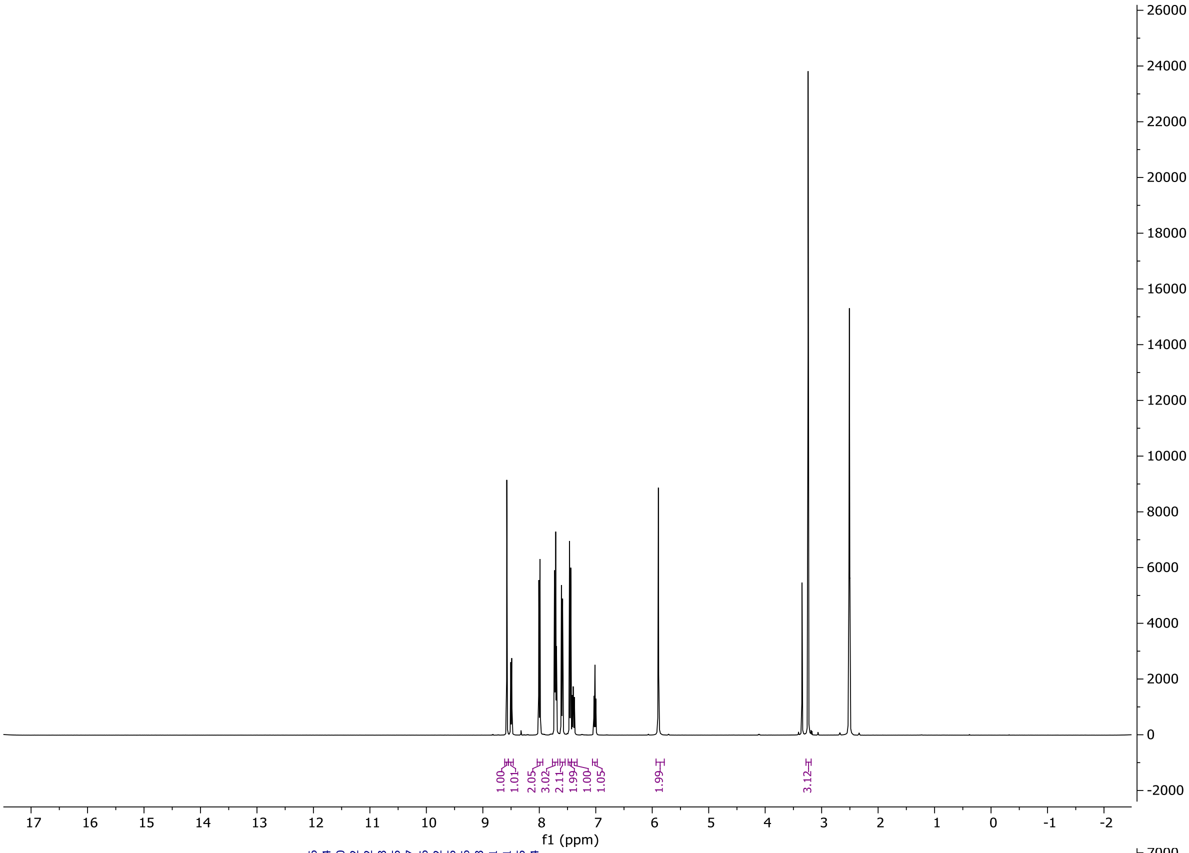

1.00
1.01
2.05
3.02
2.11
1.99
1.00
1.05
1.99
3.12
f1 (ppm)

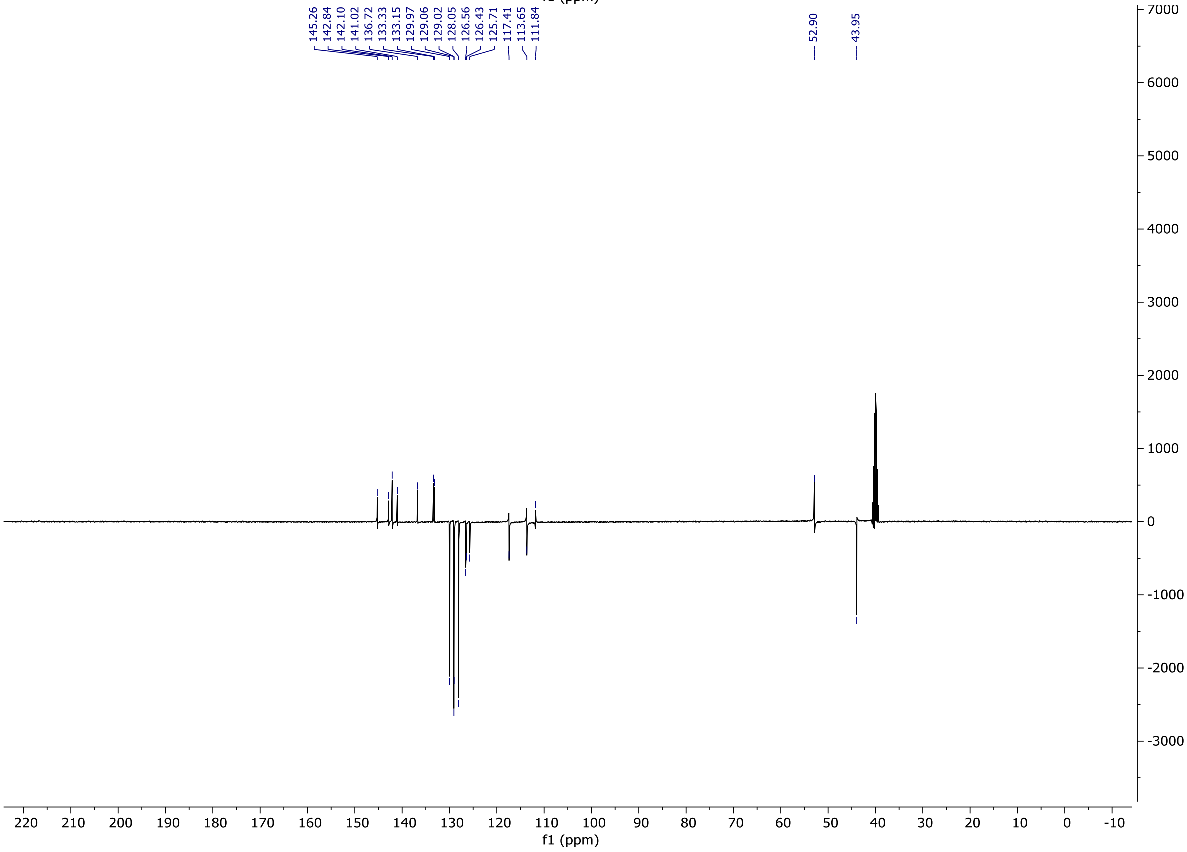

145.26
142.84
142.10
141.02
136.72
133.33
133.15
129.97
129.06
129.02
128.05
126.56
126.43
125.71
117.41
113.65
111.84
52.90
43.95
f1 (ppm)

15j

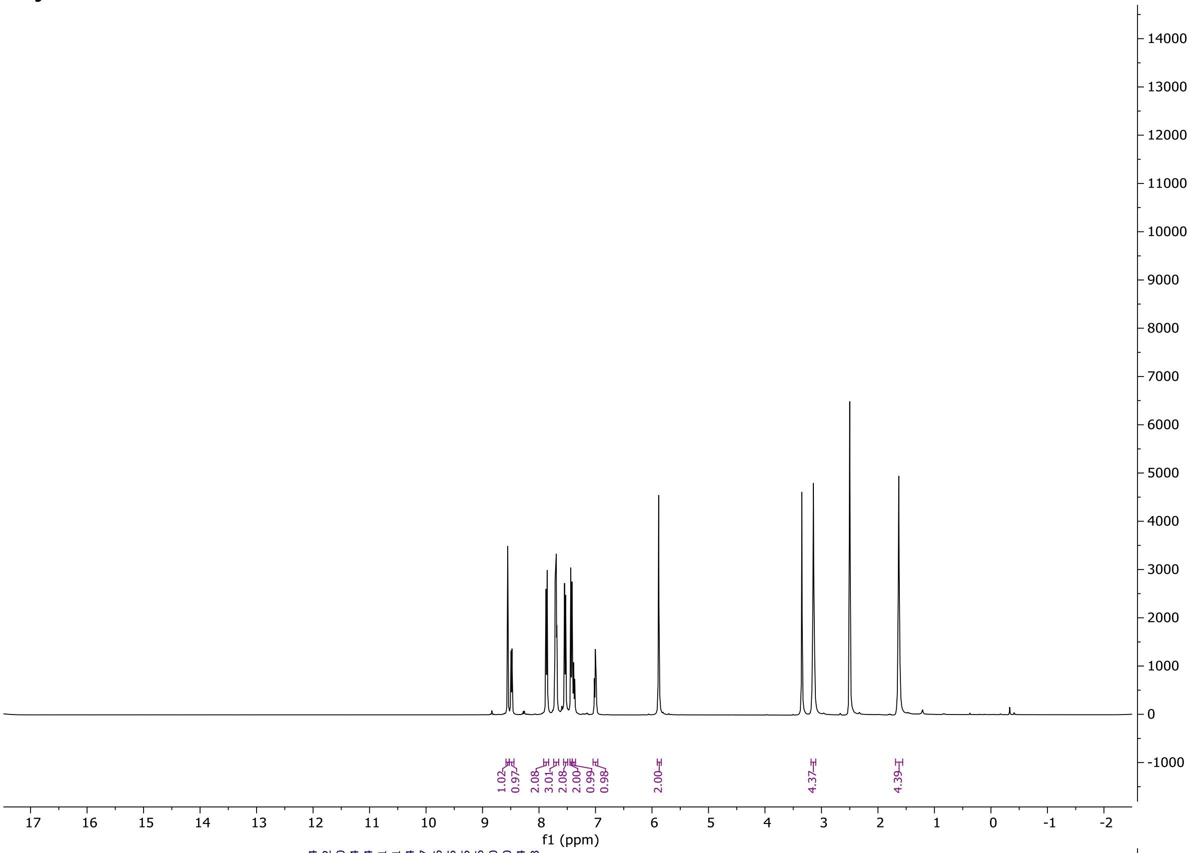
1.02
0.97
2.08
3.01
2.08
2.00
0.99
0.98
2.00
4.37
4.39
f1 (ppm)

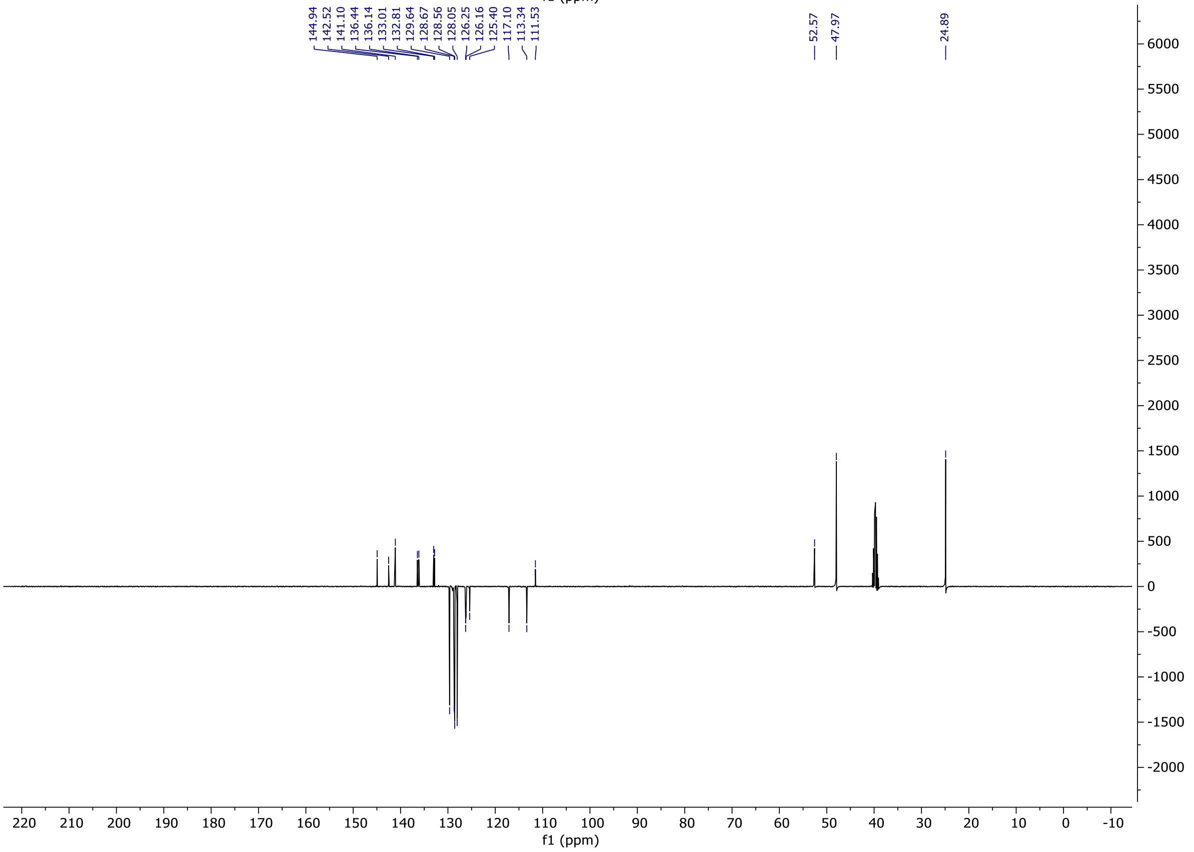
144.94
142.52
141.10
136.44
136.14
133.01
132.81
129.64
128.67
128.56
128.05
126.25
126.16
125.40
117.10
113.34
111.53
52.57
47.97
24.89
f1 (ppm)

15k

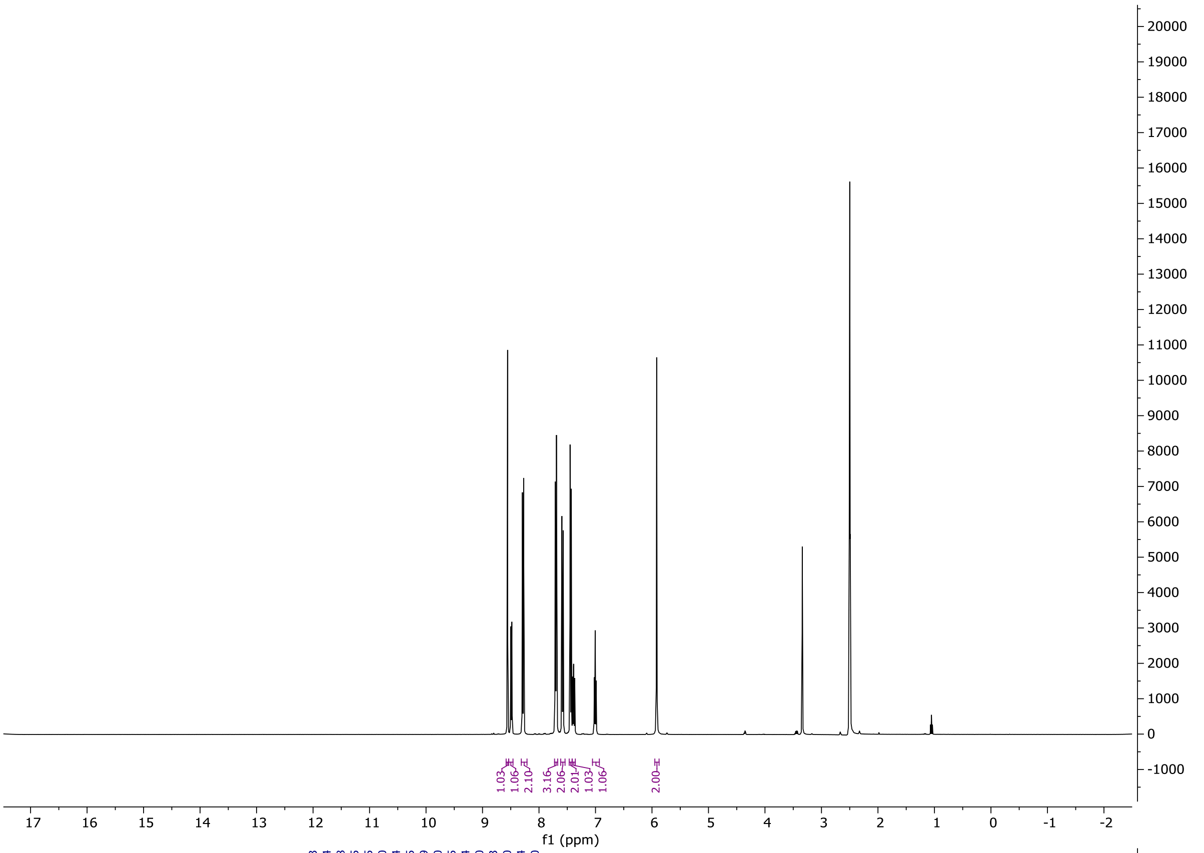
1.03
1.06
2.10
3.16
2.06
2.01
1.03
1.06
2.00
f1 (ppm)

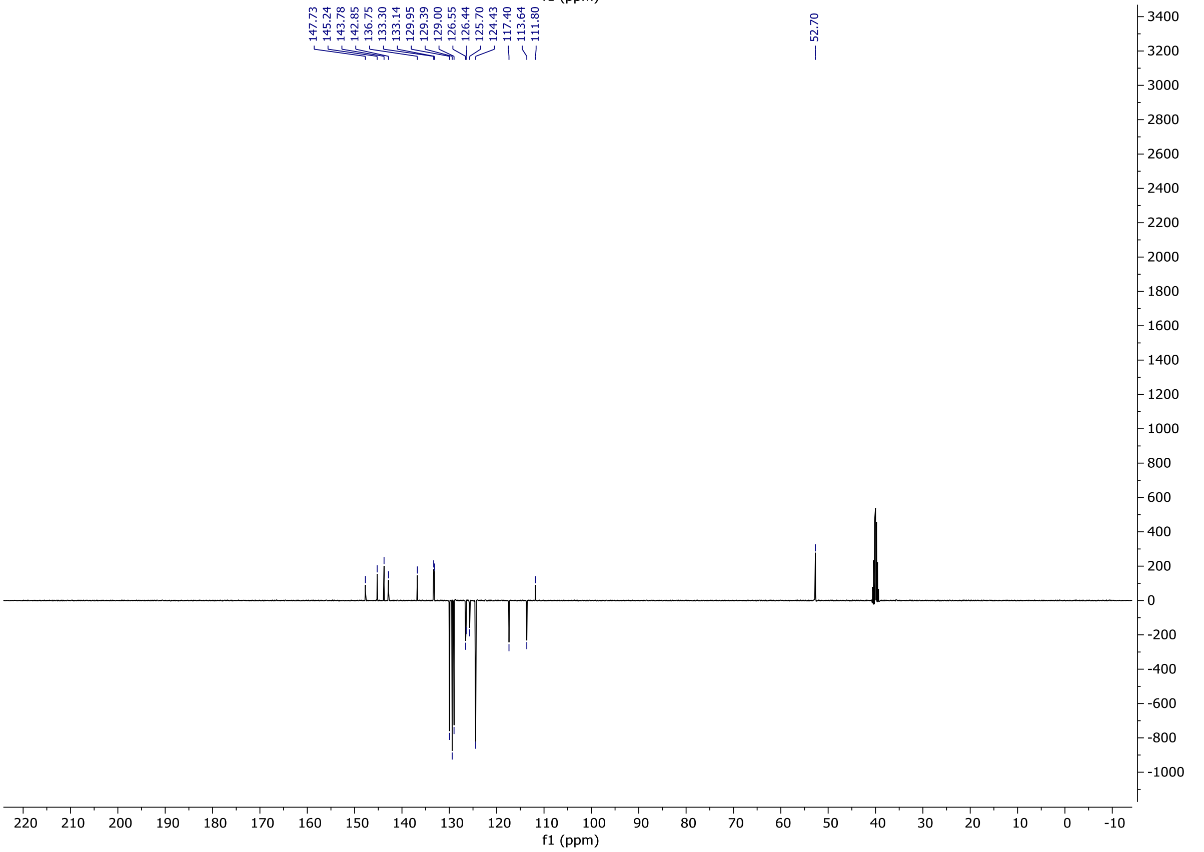
147.73
145.24
143.78
142.85
136.75
133.30
133.14
129.95
129.39
129.00
126.55
126.44
125.70
124.43
117.40
113.64
111.80
52.70
f1 (ppm)

15l

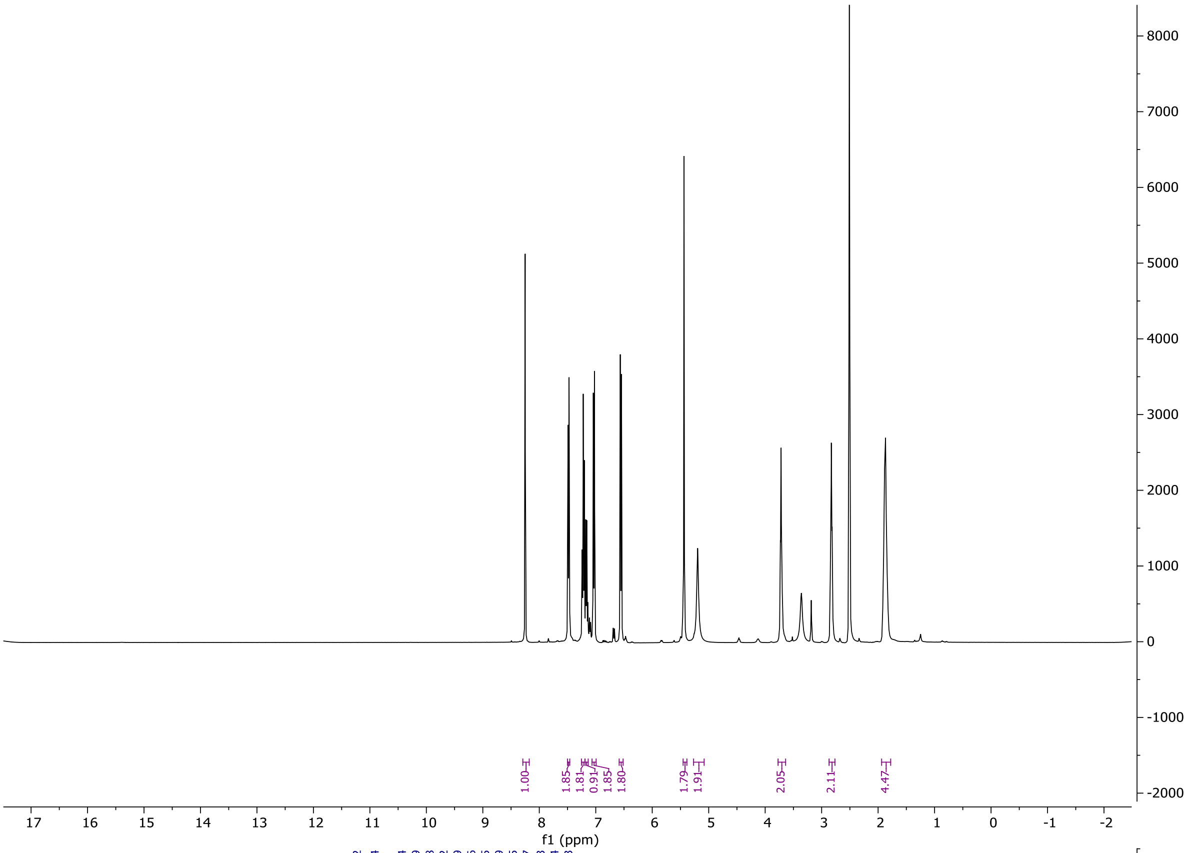
1.00
1.85
1.81
0.91
1.85
1.80
1.79
1.91
2.05
2.11
4.47
f1 (ppm)

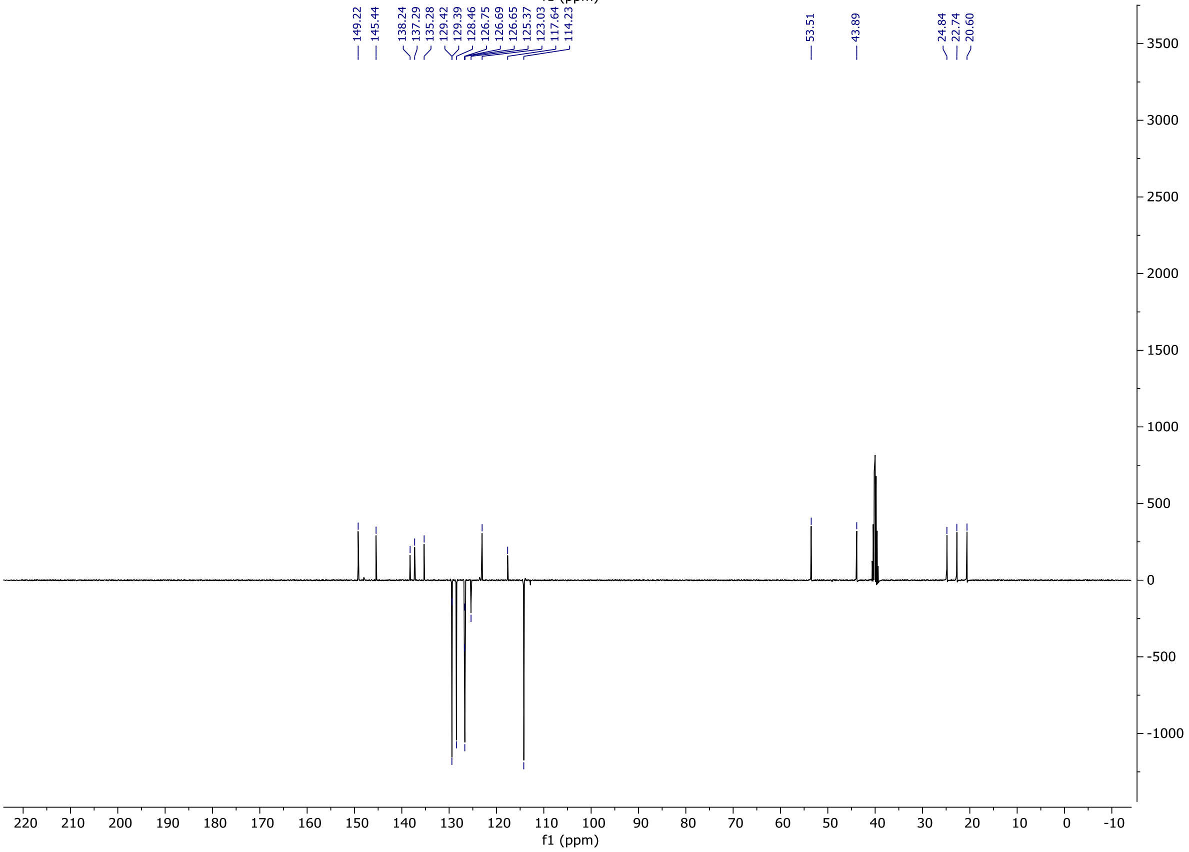
149.22
145.44
138.24
137.29
135.28
129.42
129.39
128.46
126.75
126.69
126.65
125.37
123.03
117.64
114.23
53.51
43.89
24.84
22.74
20.60
f1 (ppm)

15m

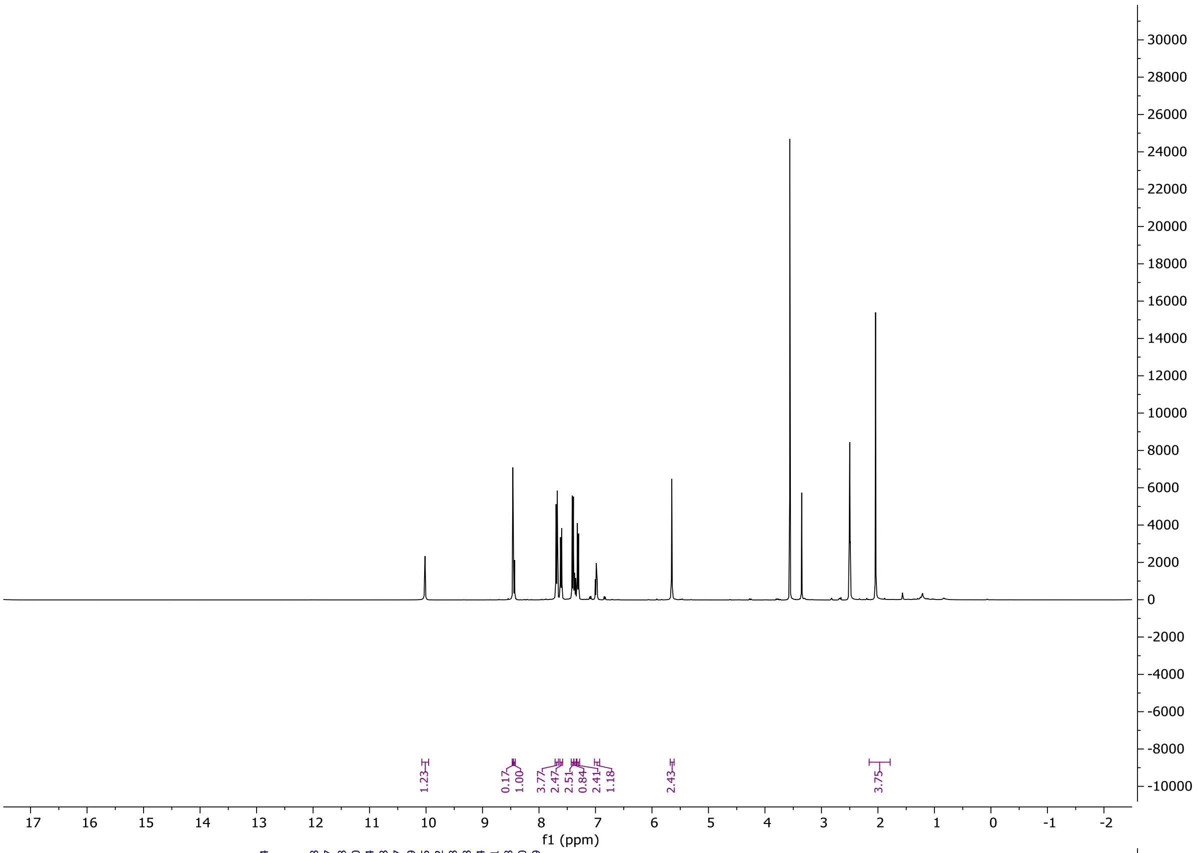
1.23
0.17
1.00
3.77
2.47
2.51
0.84
2.41
1.18
2.43
3.75
f1 (ppm)

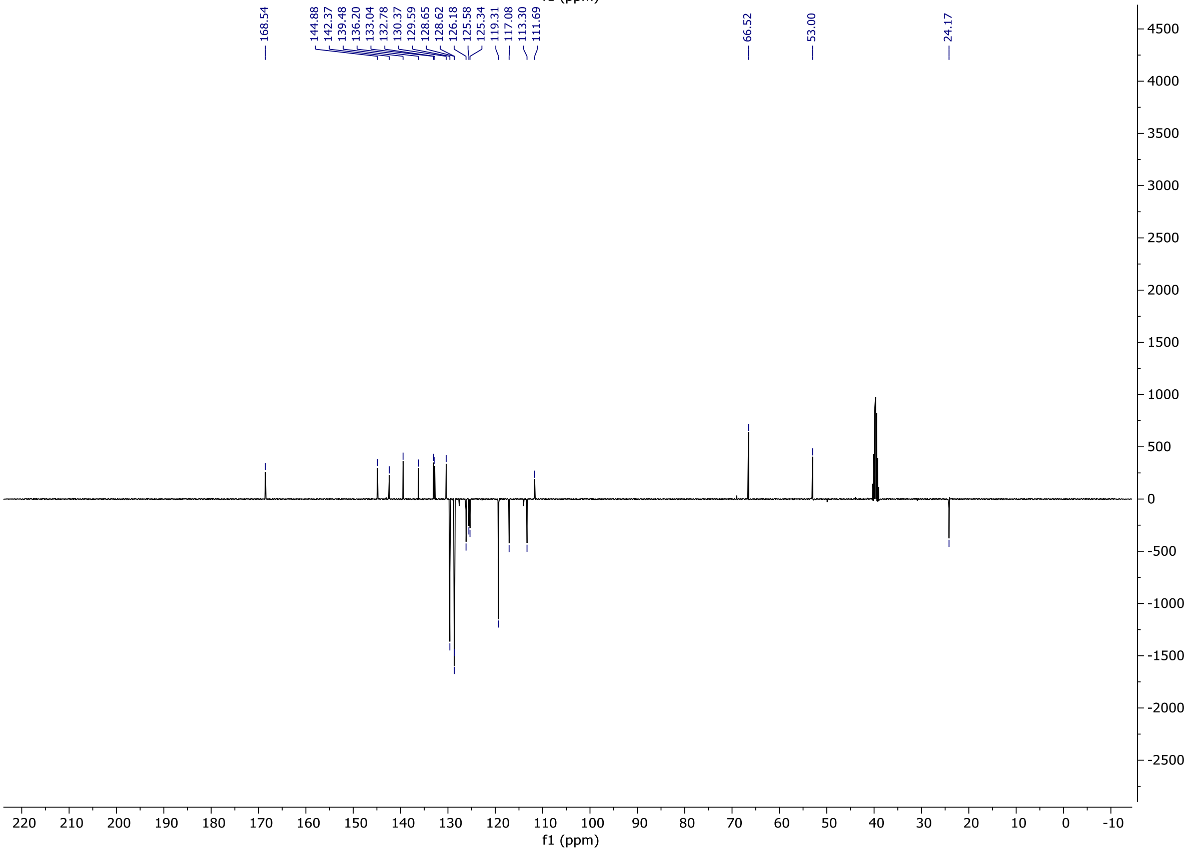
168.54
144.88
142.37
139.48
136.20
133.04
132.78
130.37
129.59
128.65
128.62
126.18
125.58
125.34
119.31
117.08
113.30
111.69
66.52
53.00
24.17
f1 (ppm)

16A

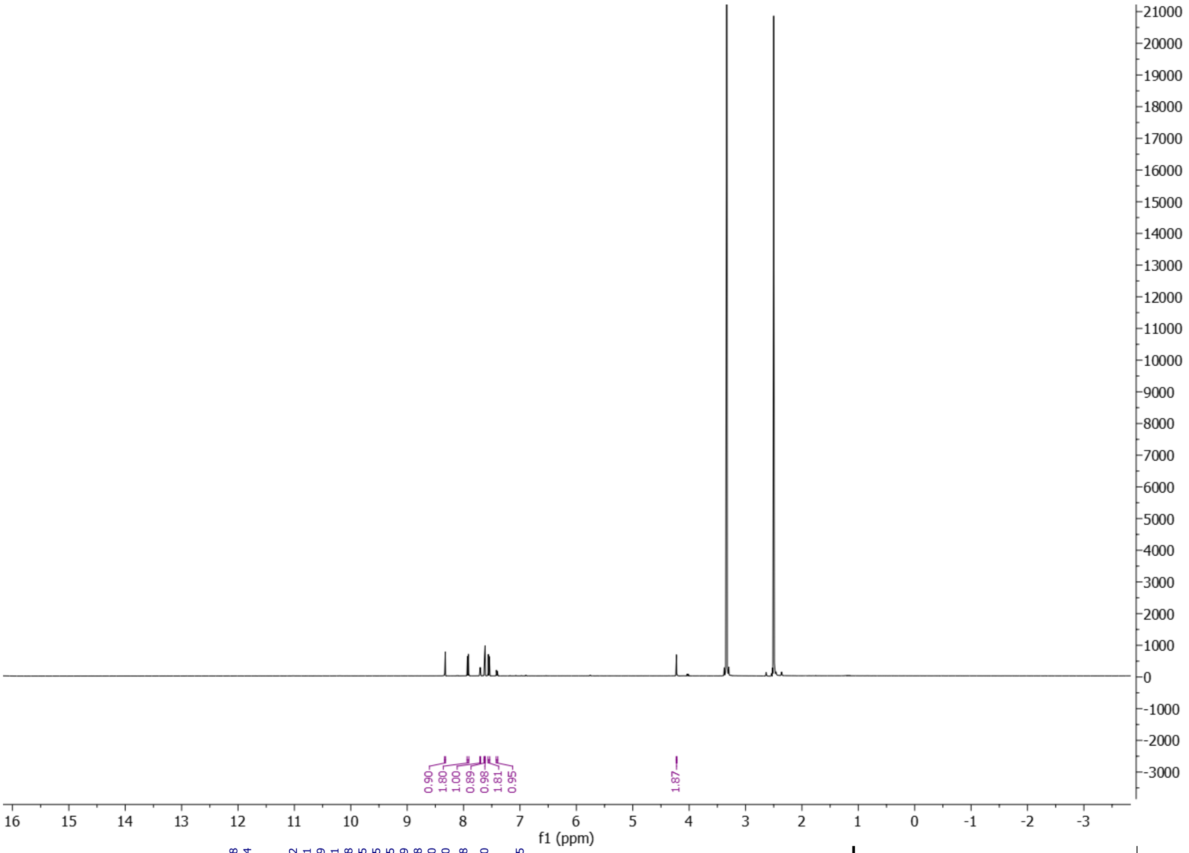
0.90
1.80
1.00
0.89
0.98
1.81
0.95
1.87
f1 (ppm)

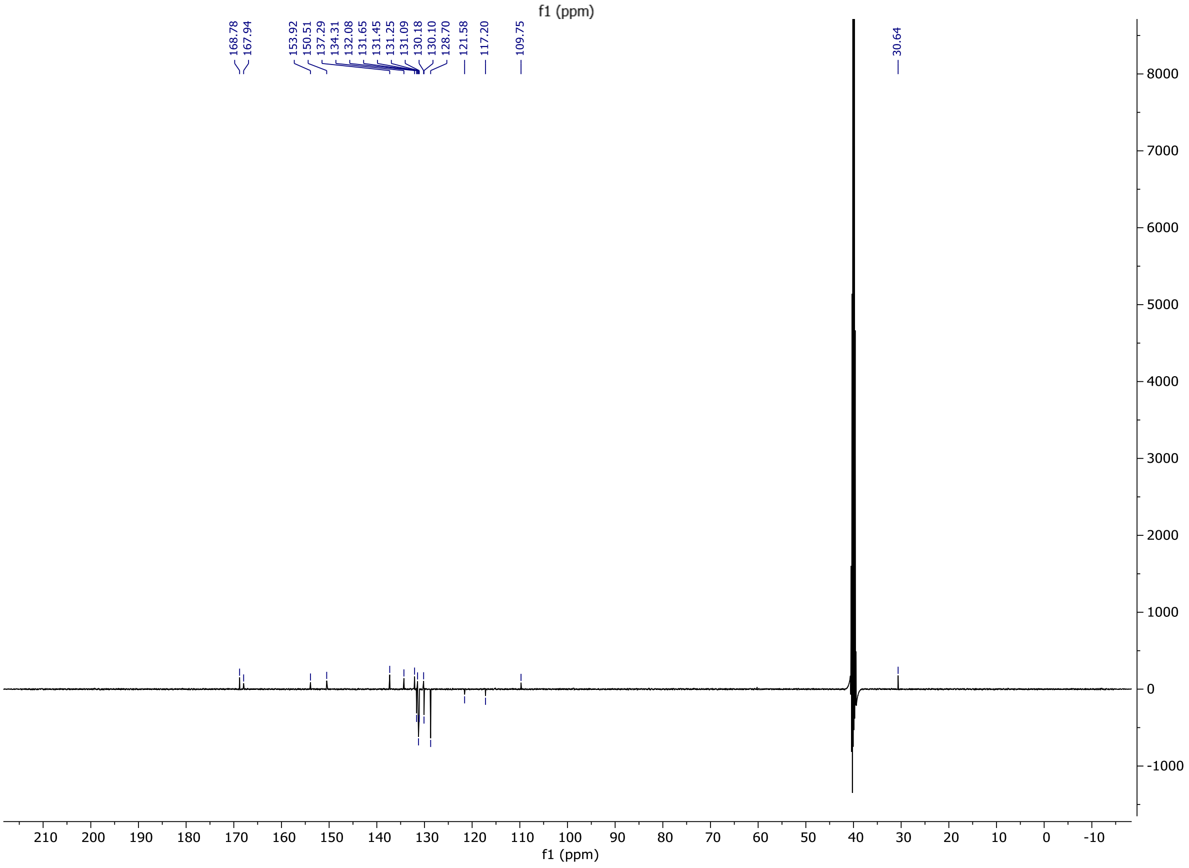
168.78
167.94
153.92
150.51
137.29
134.31
132.08
131.65
131.45
131.25
131.09
130.18
130.10
128.70
121.58
117.20
109.75
30.64
f1 (ppm)

16B

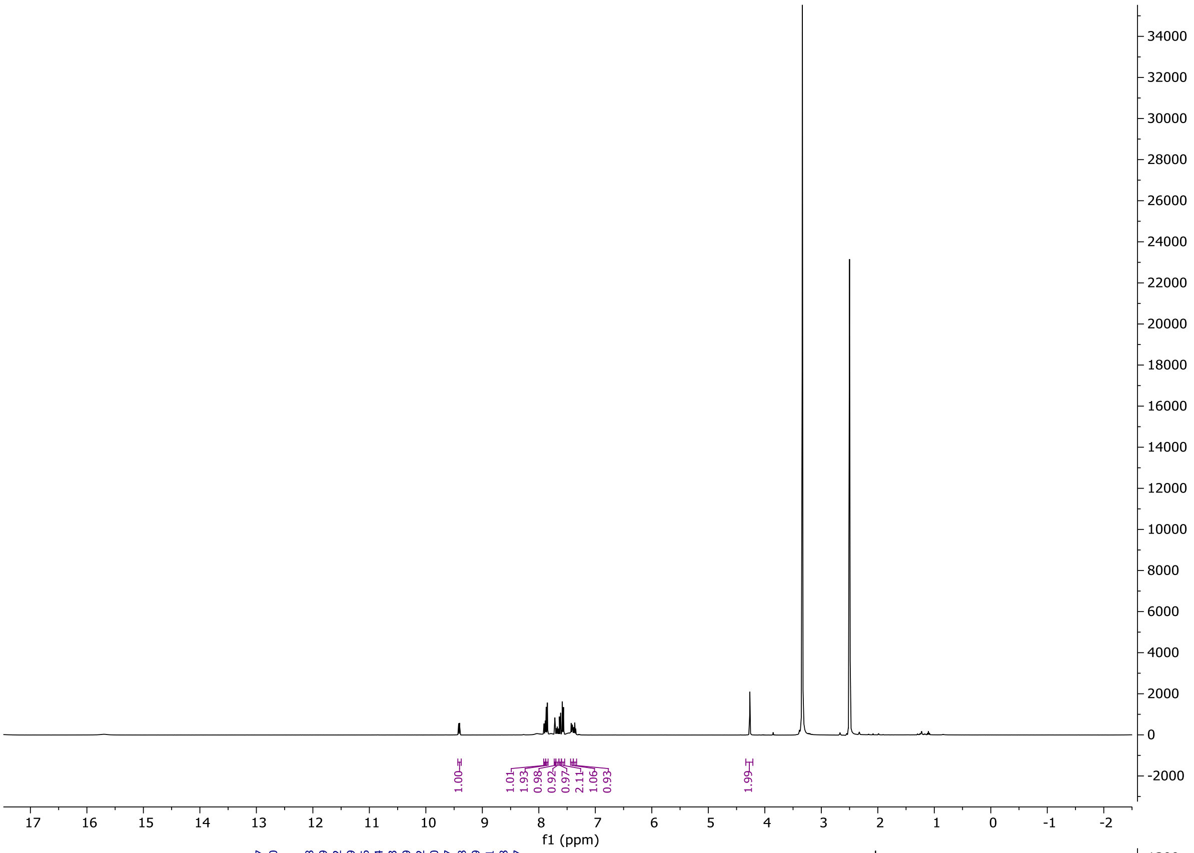
1.00
1.01
1.93
0.98
0.92
0.97
2.11
1.06
0.93
1.99
f1 (ppm)

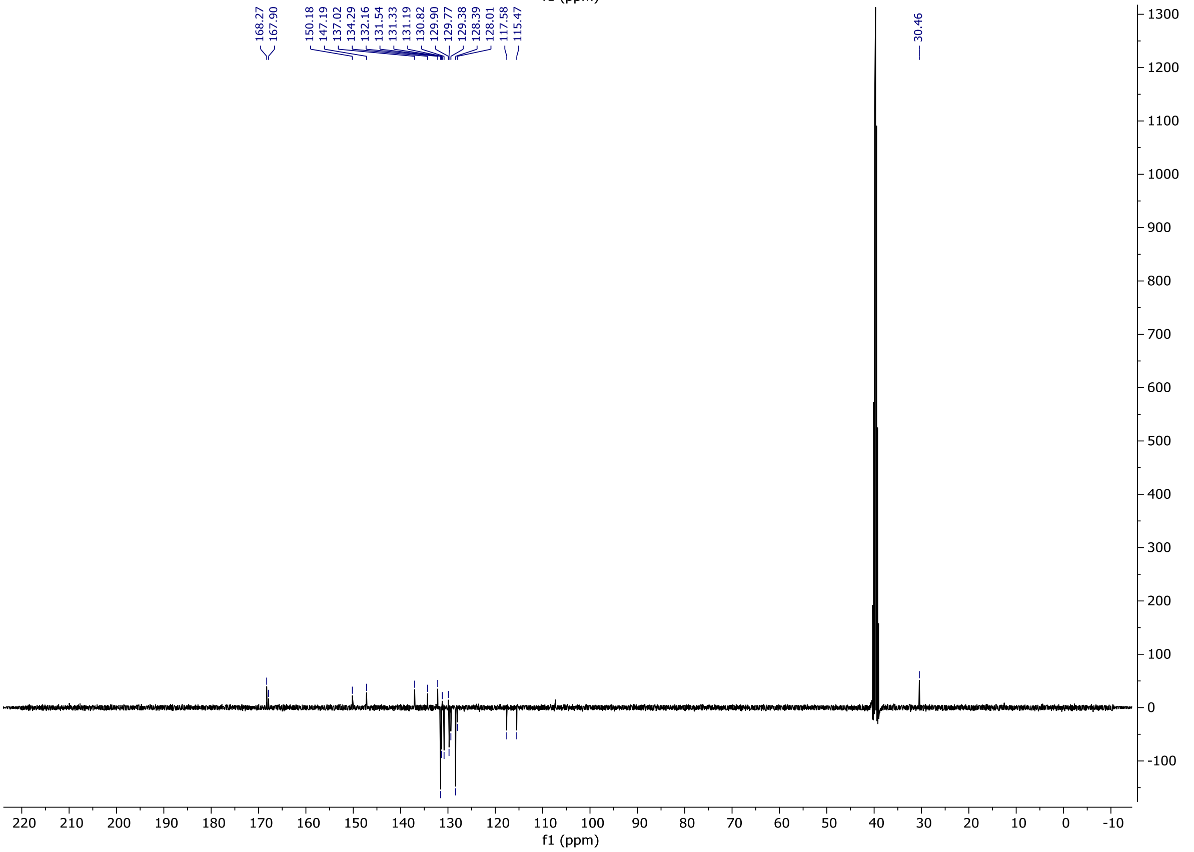
168.27
167.90
150.18
147.19
137.02
134.29
132.16
131.54
131.33
131.19
130.82
129.90
129.77
129.38
128.39
128.01
117.58
115.47
30.46
f1 (ppm)

17

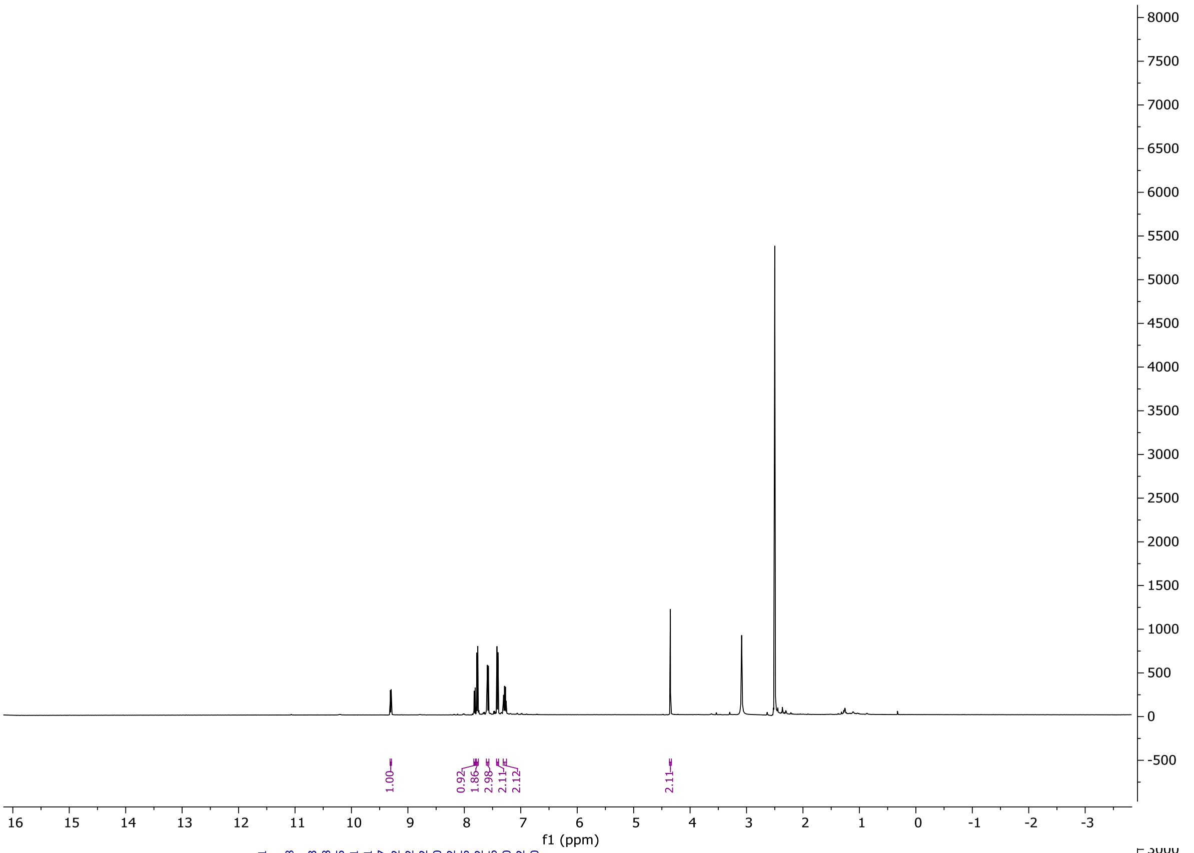

1.00
0.92
1.86
2.98
2.11
2.12
2.11
f1 (ppm)

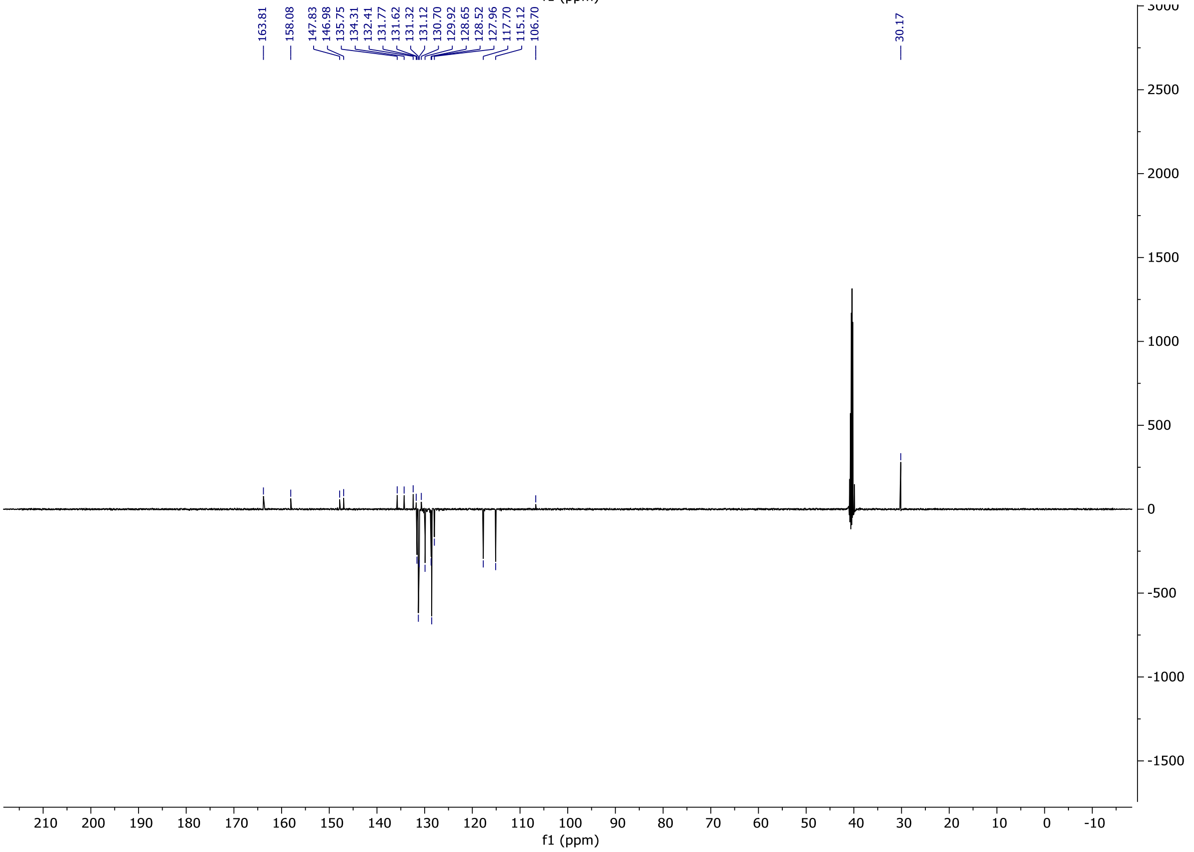

163.81
158.08
147.83
146.98
135.75
134.31
132.41
131.77
131.62
131.32
131.12
130.70
129.92
128.65
128.52
127.96
117.70
115.12
106.70
30.17
f1 (ppm)

18

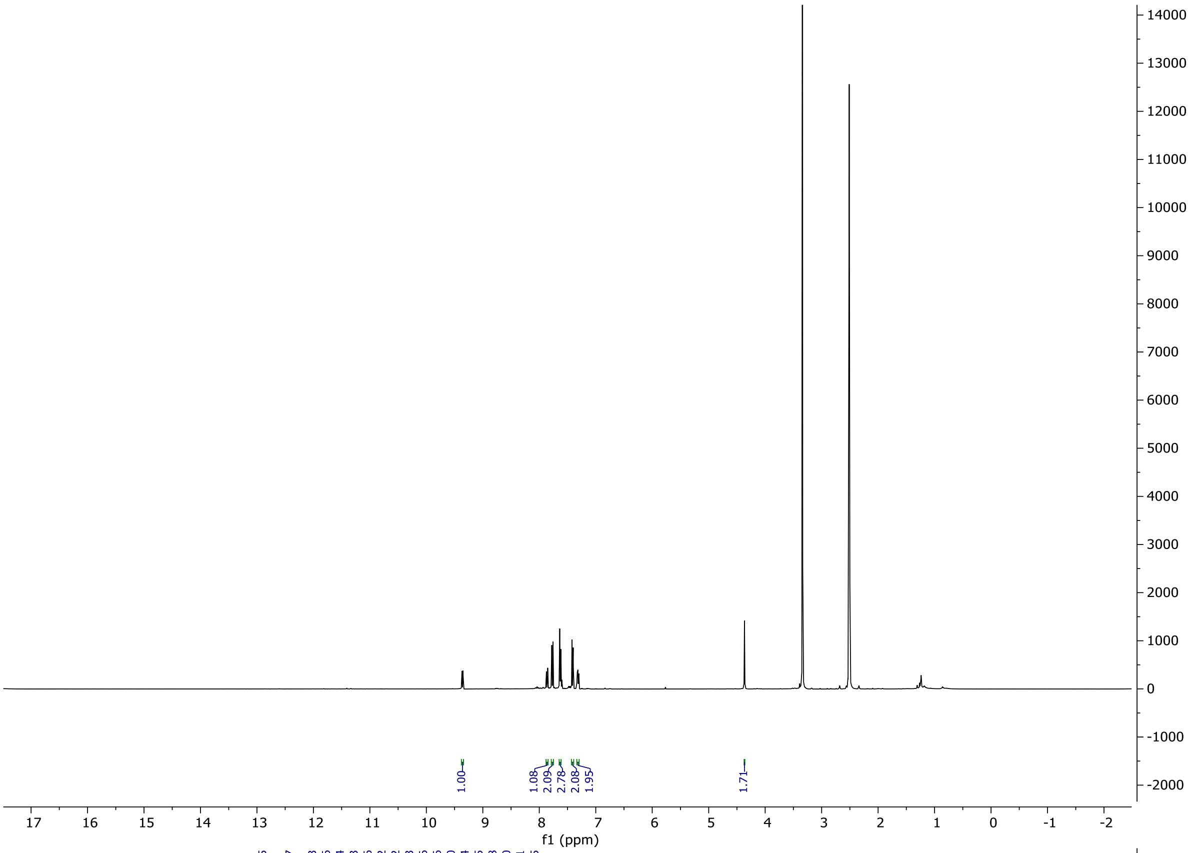
1.00
1.08
2.09
2.78
2.08
1.95
1.71
f1 (ppm)

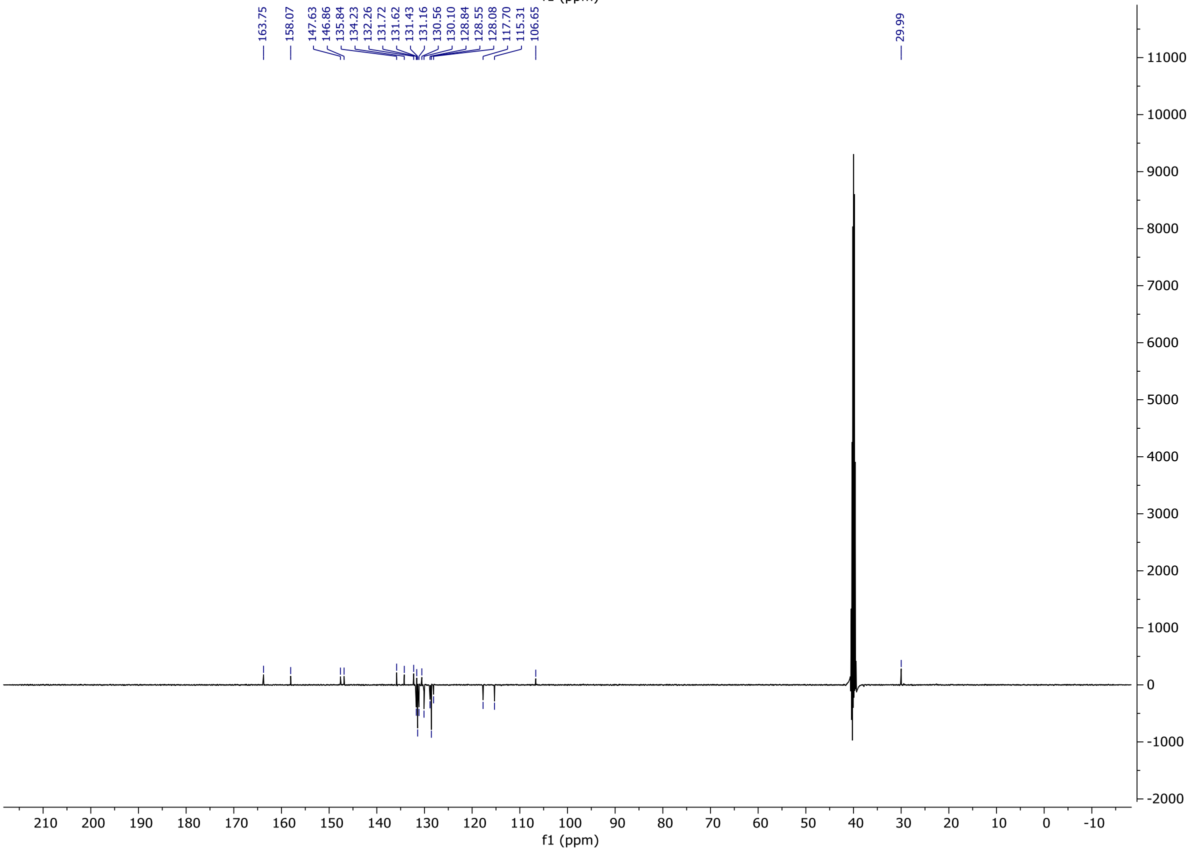
163.75
158.07
147.63
146.86
135.84
134.23
132.26
131.72
131.62
131.43
131.16
130.56
130.10
128.84
128.55
128.08
117.70
115.31
106.65
29.99
f1 (ppm)

19A

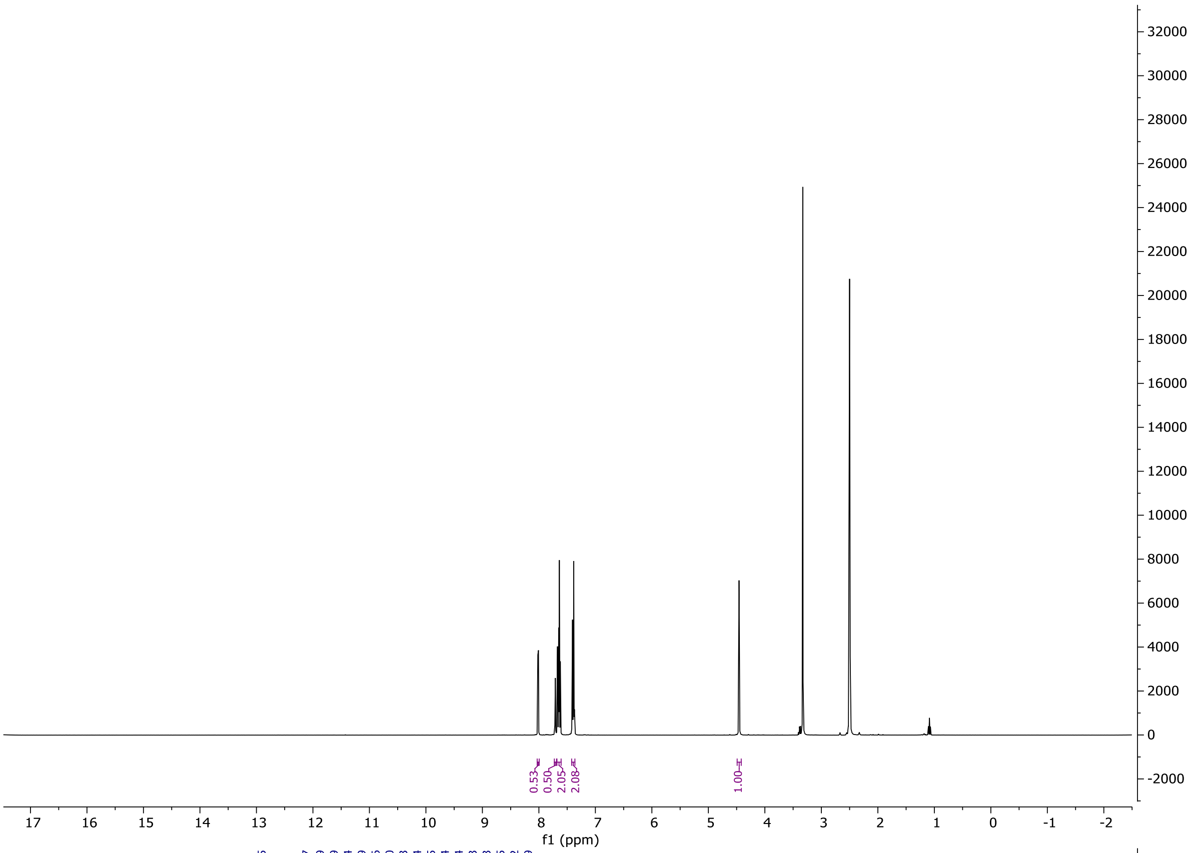
0.53
0.50
2.05
2.08
1.00
f1 (ppm)

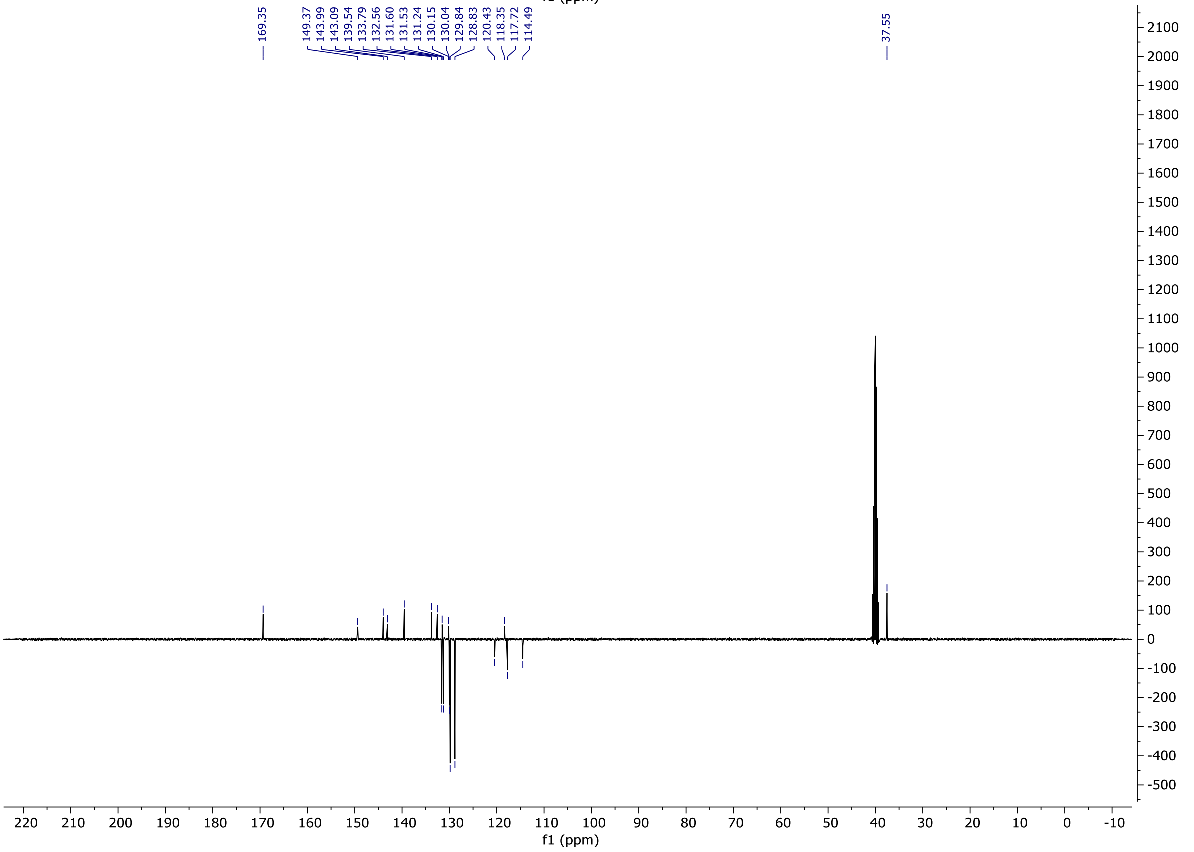
169.35
149.37
143.99
143.09
139.54
133.79
132.56
131.60
131.53
131.24
130.15
130.04
129.84
128.83
120.43
118.35
117.72
114.49
37.55
f1 (ppm)

19B

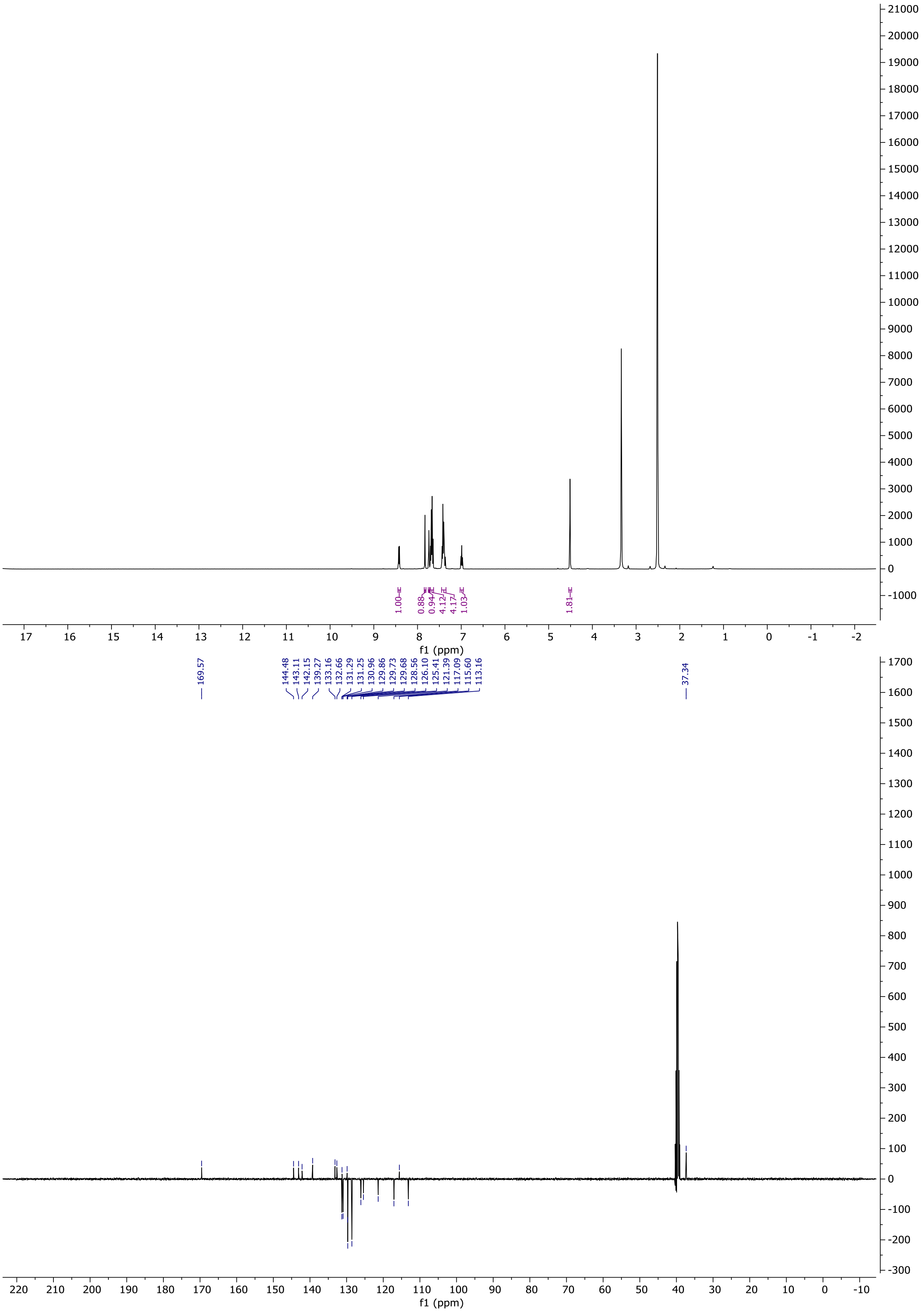

1.00
0.88
0.94
4.12
4.17
1.03
1.81
f1 (ppm)
169.57
144.48
143.11
142.15
139.27
133.16
132.66
131.29
131.25
130.96
129.86
129.73
129.68
128.56
126.10
125.41
121.39
117.09
115.60
113.16
37.34
f1 (ppm)

20

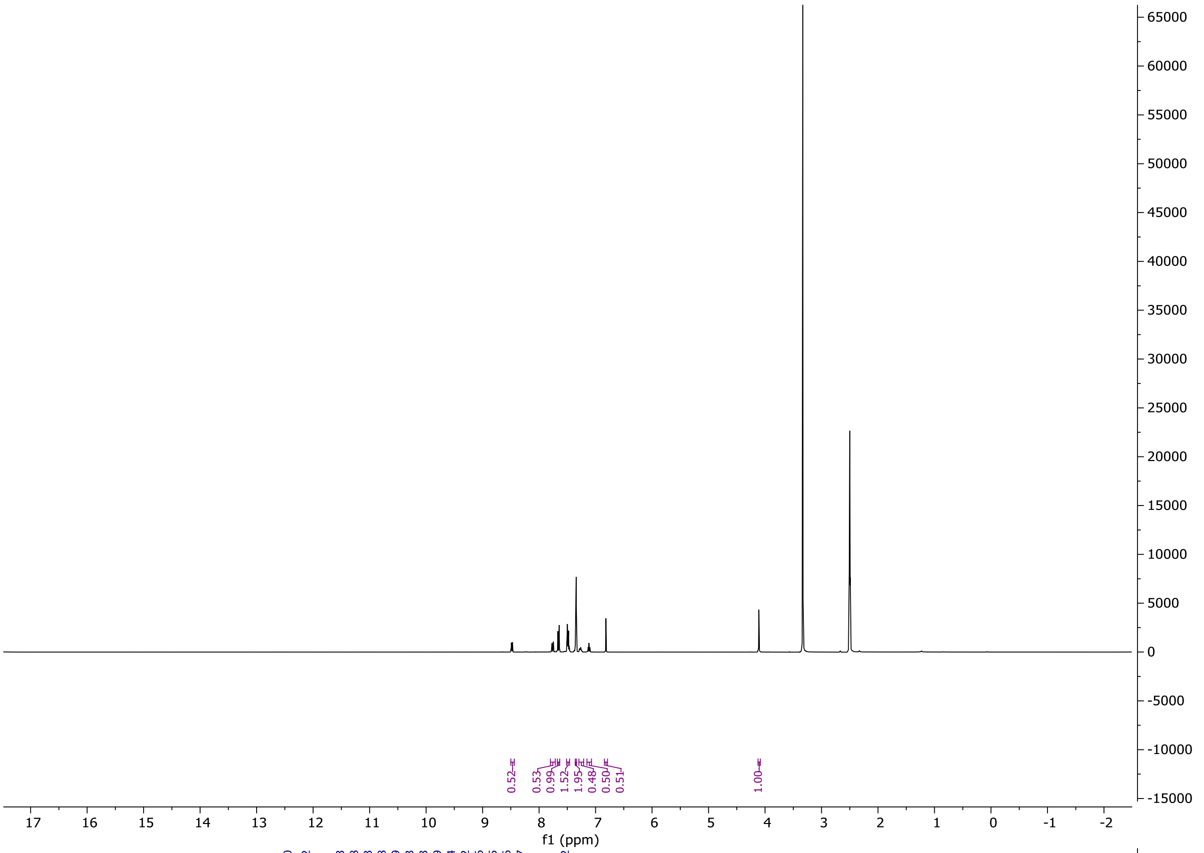
0.52
0.53
0.99
1.52
1.95
0.48
0.50
0.51
1.00
f1 (ppm)

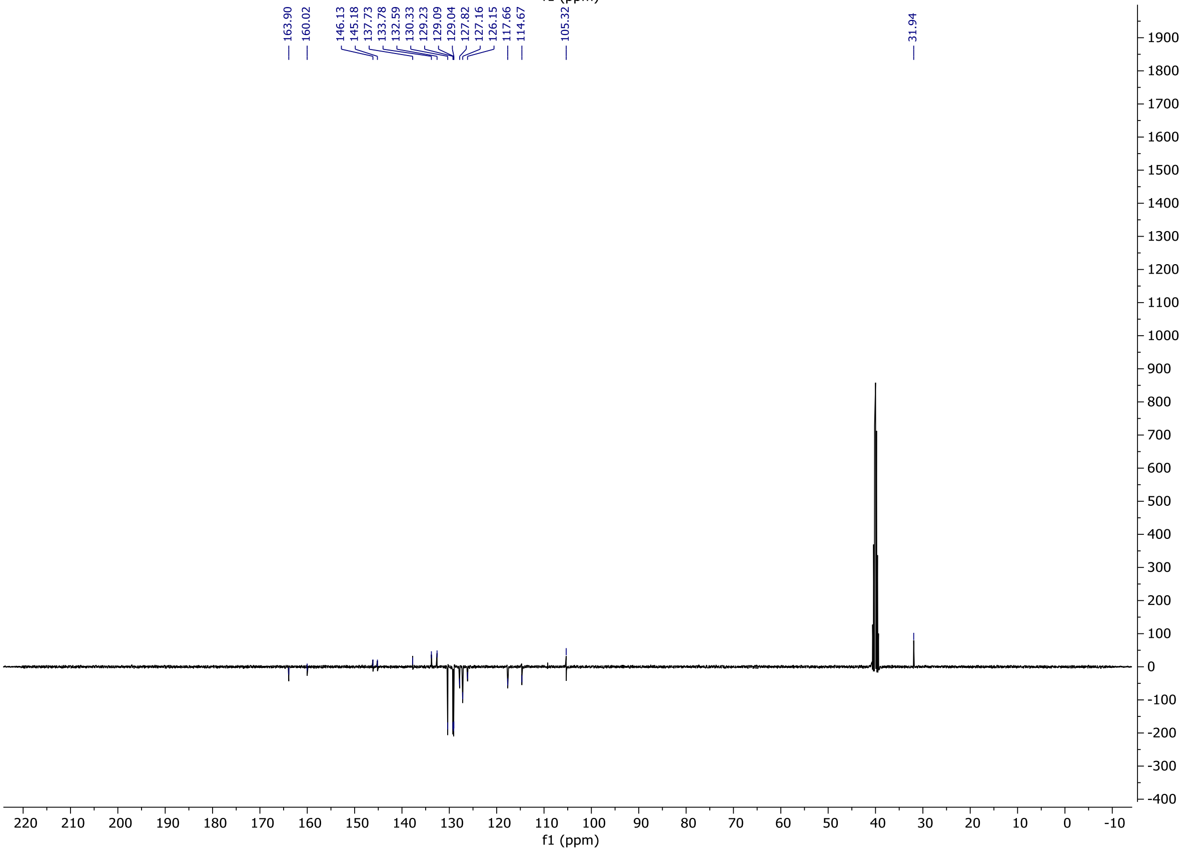
163.90
160.02
146.13
145.18
137.73
133.78
132.59
130.33
129.23
129.09
129.04
127.82
127.16
126.15
117.66
114.67
105.32
31.94
f1 (ppm)

21

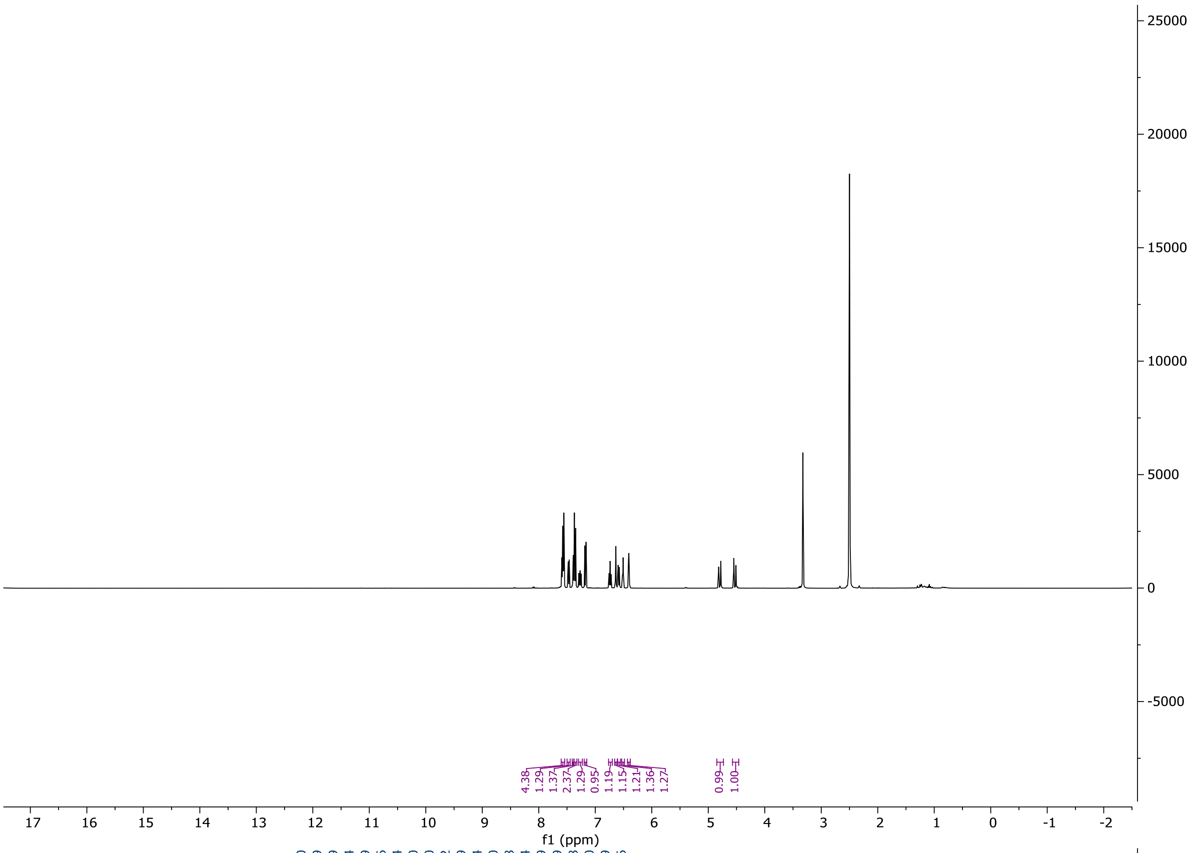
4.38
1.29
1.37
2.37
1.29
0.95
1.19
1.15
1.21
1.36
1.27
0.99
1.00
f1 (ppm)

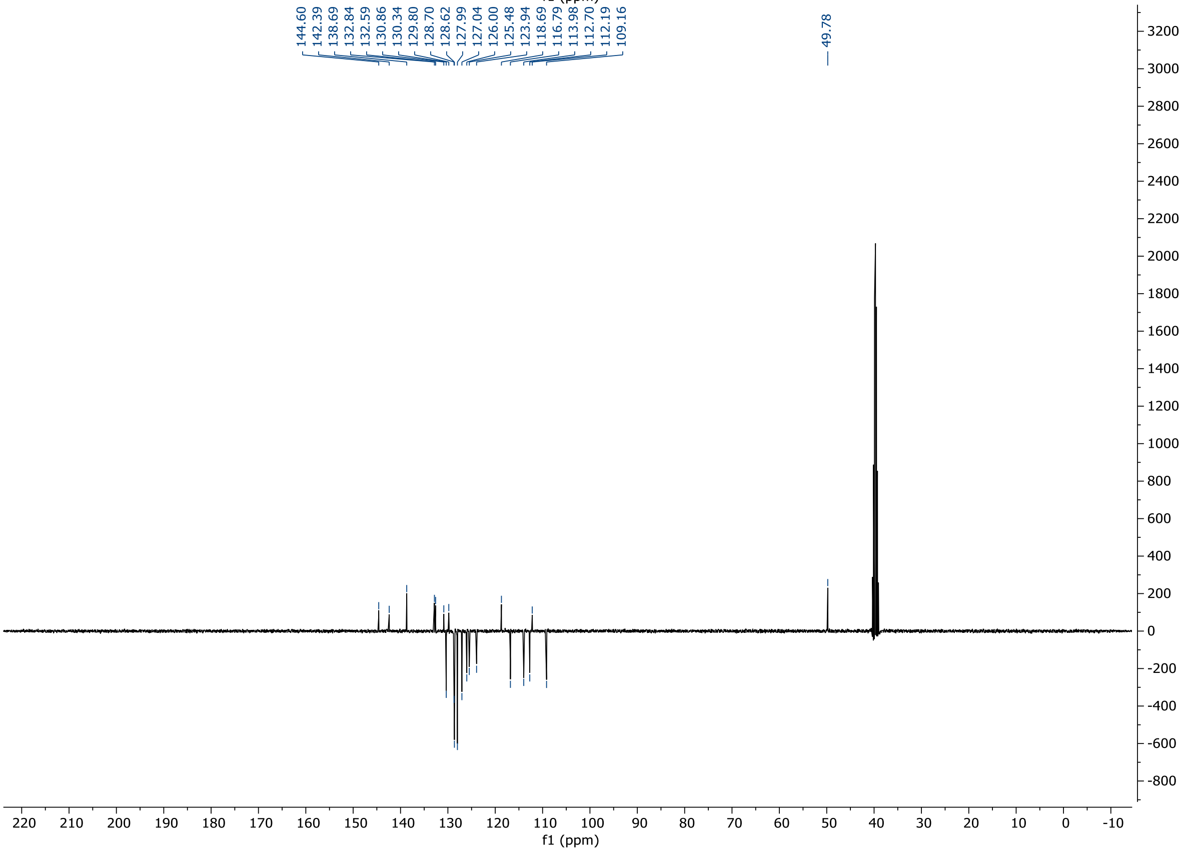
144.60
142.39
138.69
132.84
132.59
130.86
130.34
129.80
128.70
128.62
127.99
127.04
126.00
125.48
123.94
118.69
116.79
113.98
112.70
112.19
109.16
49.78
f1 (ppm)

22

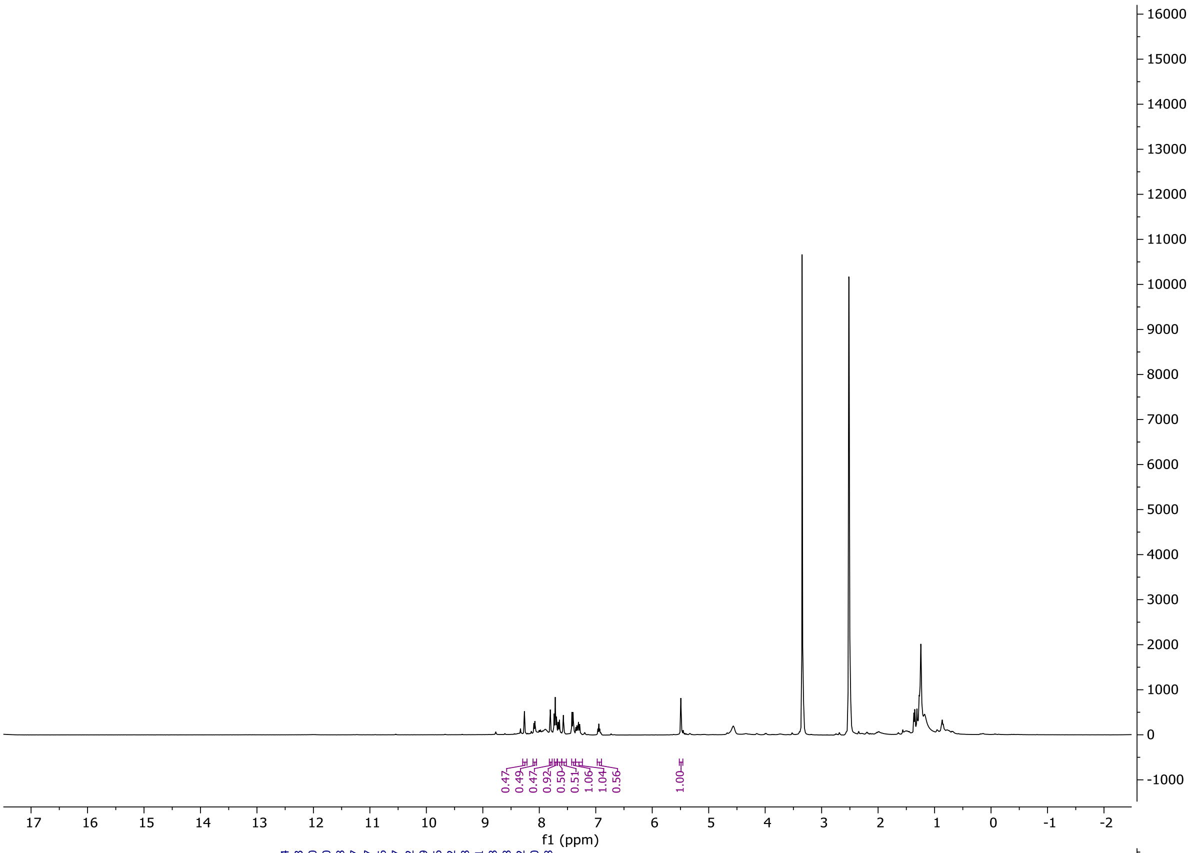
0.47
0.49
0.47
0.92
0.50
0.51
1.06
1.04
0.56
1.00
f1 (ppm)

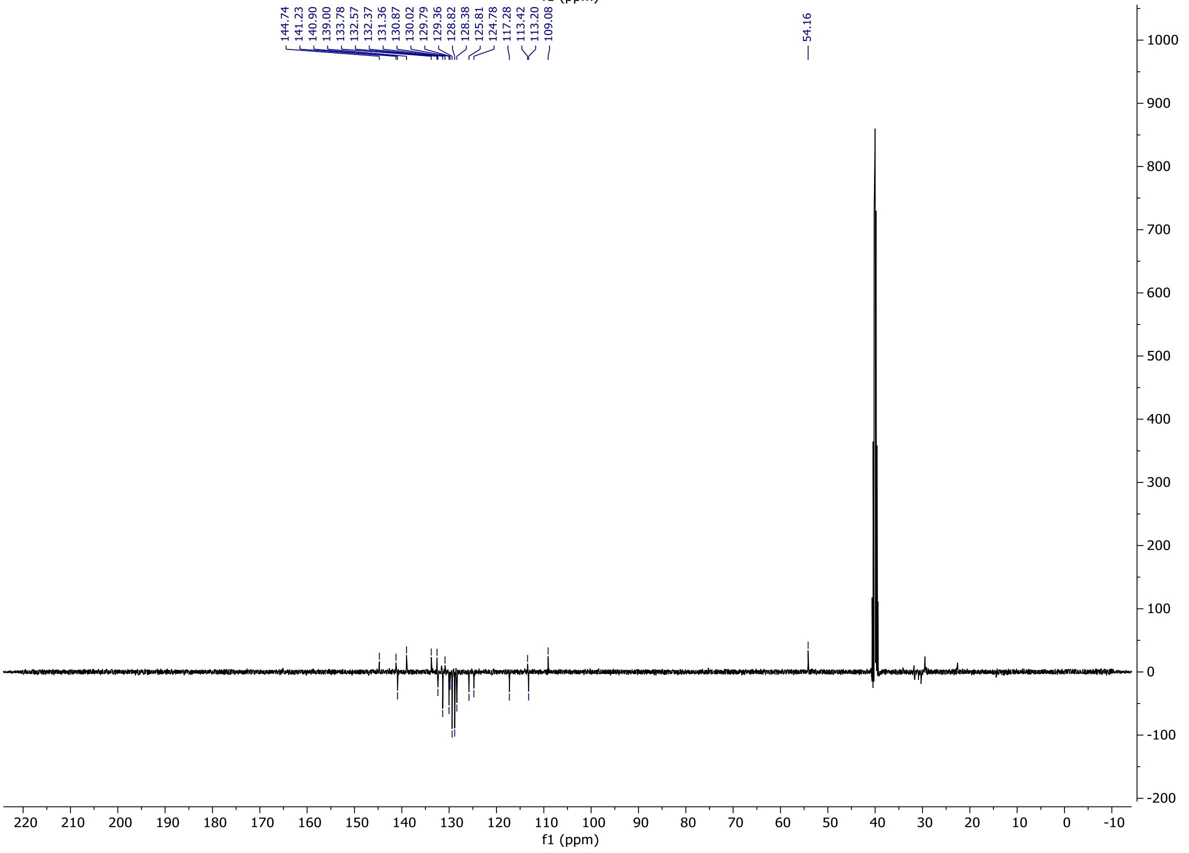
144.74
141.23
140.90
139.00
133.78
132.57
132.37
131.36
130.87
130.02
129.79
129.36
128.82
128.38
125.81
124.78
117.28
113.42
113.20
109.08
54.16
f1 (ppm)

23

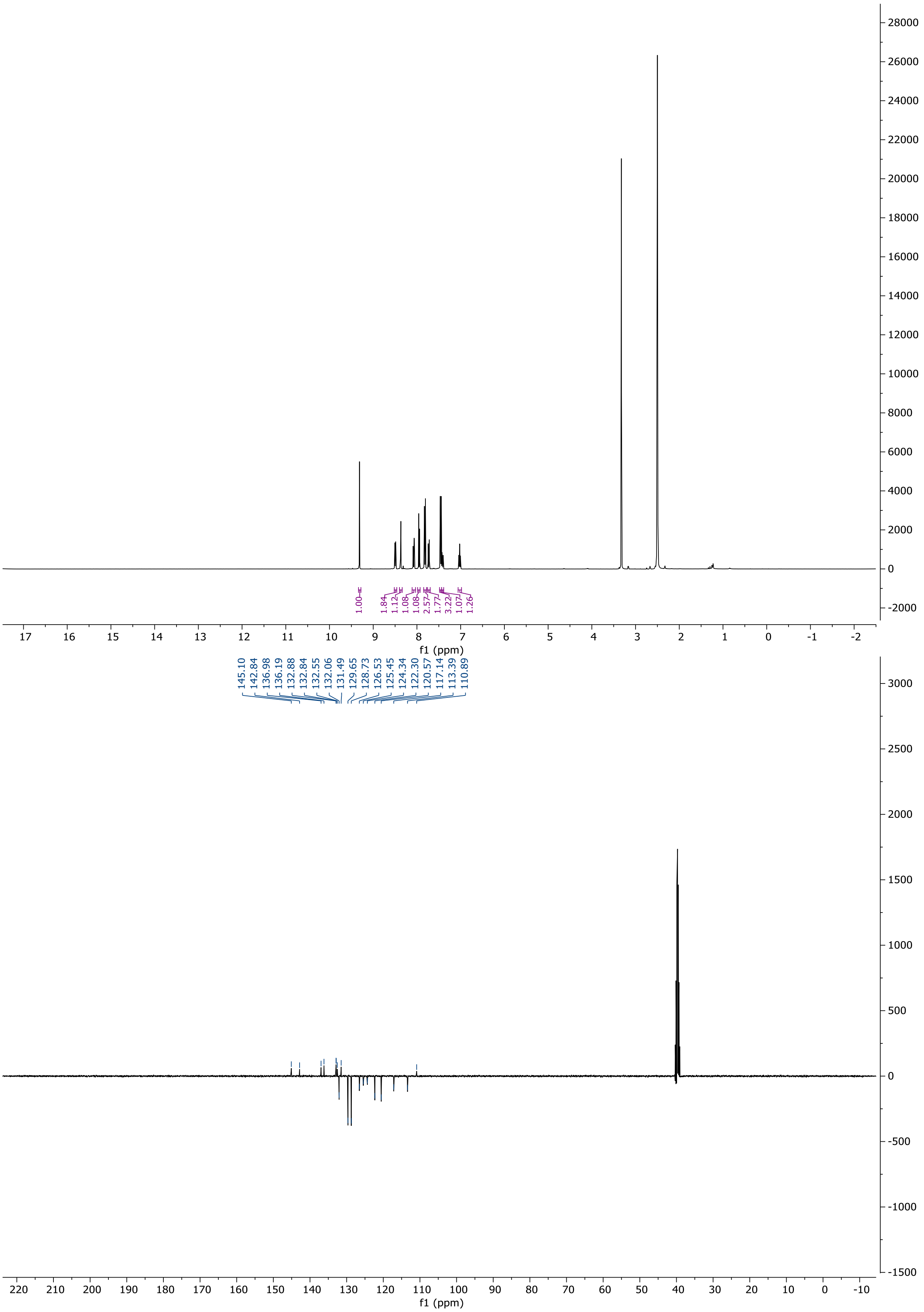

1.00
1.84
1.12
1.08
1.08
2.57
1.77
3.22
1.07
1.26
f1 (ppm)
145.10
142.84
136.98
136.19
132.88
132.84
132.55
132.06
131.49
129.65
128.73
126.53
125.45
124.34
122.30
120.57
117.14
113.39
110.89
f1 (ppm)

24

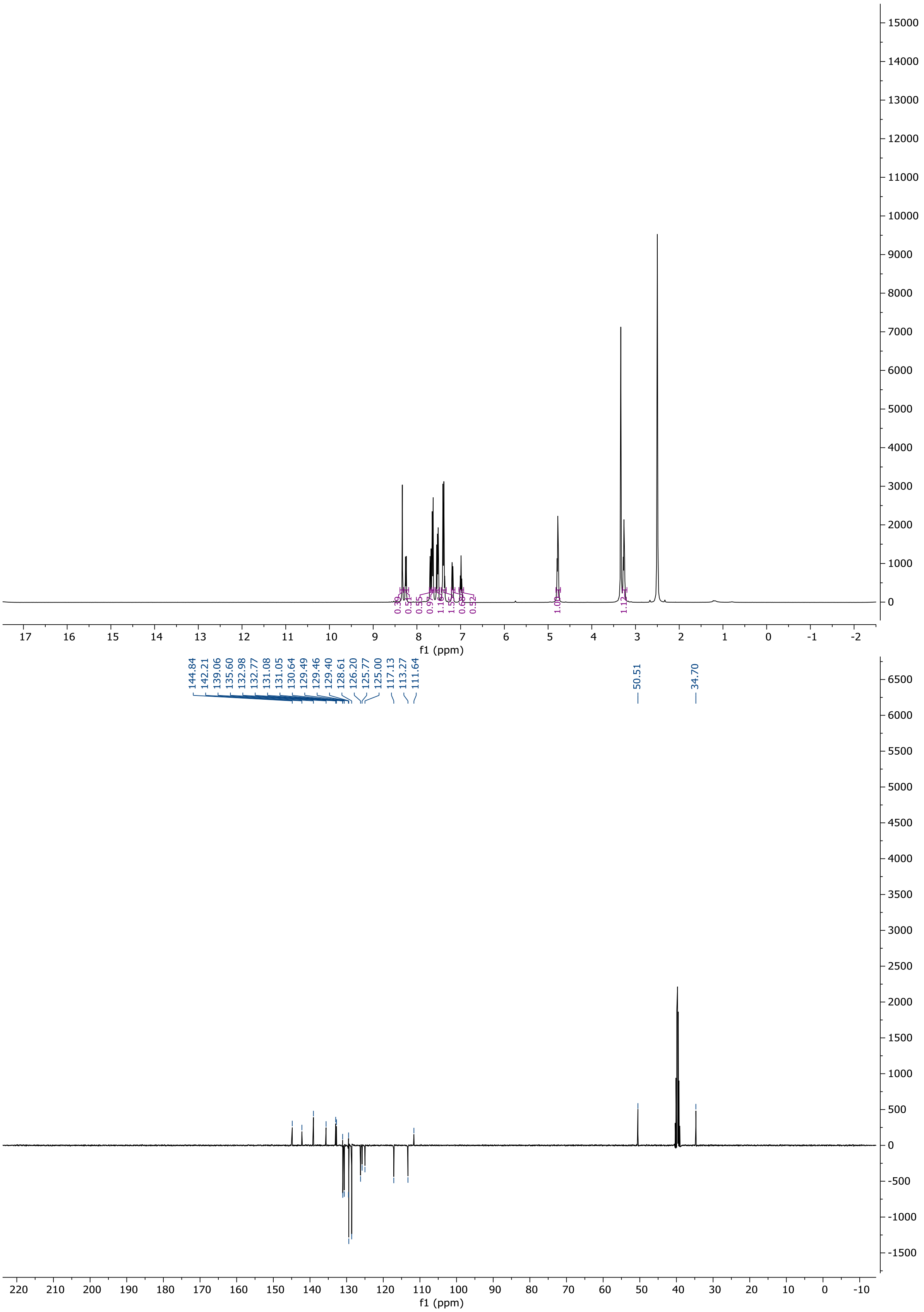

144.84
142.21
139.06
135.60
132.98
132.77
131.08
131.05
130.64
129.49
129.46
129.40
128.61
126.20
125.77
125.00
117.13
113.27
111.64
50.51
34.70
f1 (ppm)
f1 (ppm)

37

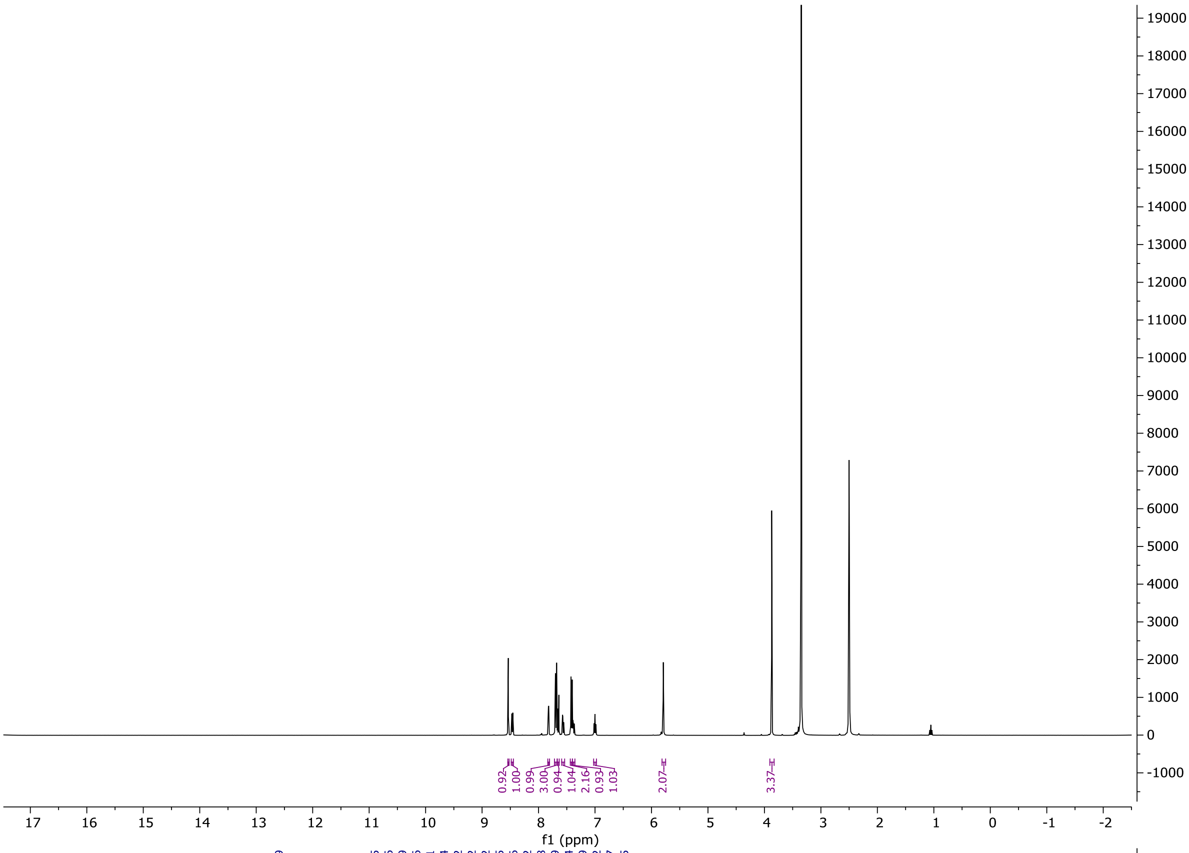
0.92
1.00
0.99
3.00
0.94
1.04
2.16
0.93
1.03
2.07
3.37
f1 (ppm)

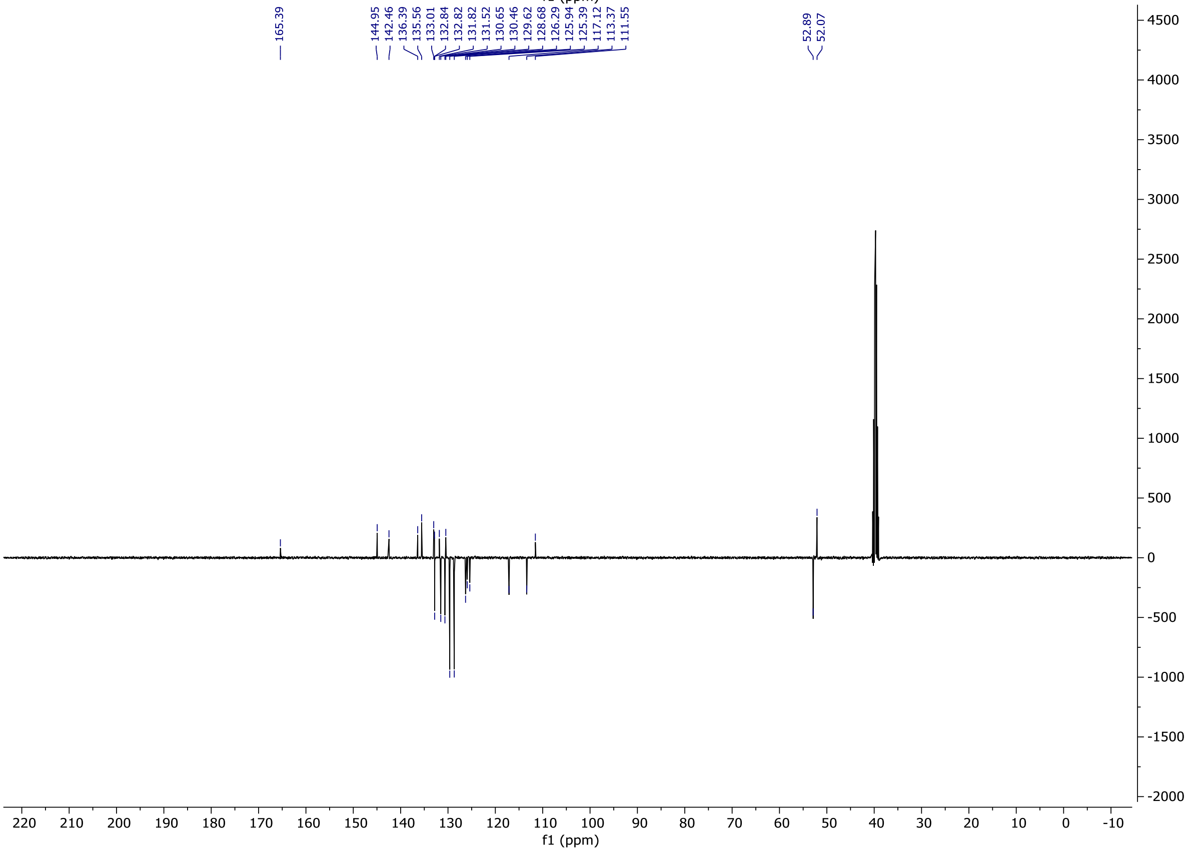
165.39
144.95
142.46
136.39
135.56
133.01
132.84
132.82
131.82
131.52
130.65
130.46
129.62
128.68
126.29
125.94
125.39
117.12
113.37
111.55
52.89
52.07
f1 (ppm)

38

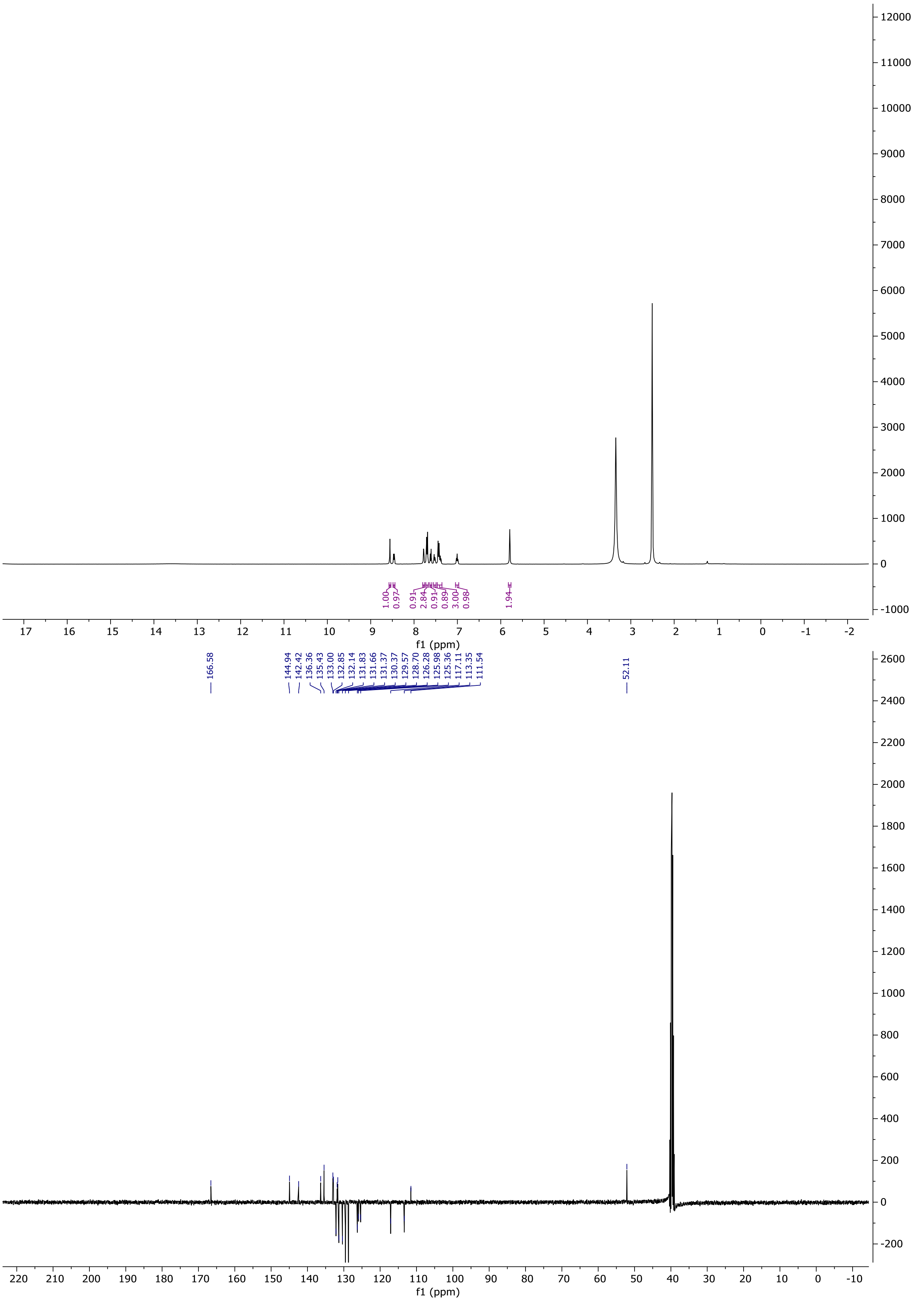
1.00
0.97
0.91
2.84
0.91
0.89
3.00
0.98
1.94
f1 (ppm)
166.58
144.94
142.42
136.36
135.43
133.00
132.85
132.14
131.83
131.66
131.37
130.37
129.57
128.70
126.28
125.98
125.36
117.11
113.35
111.54
52.11
f1 (ppm)

39

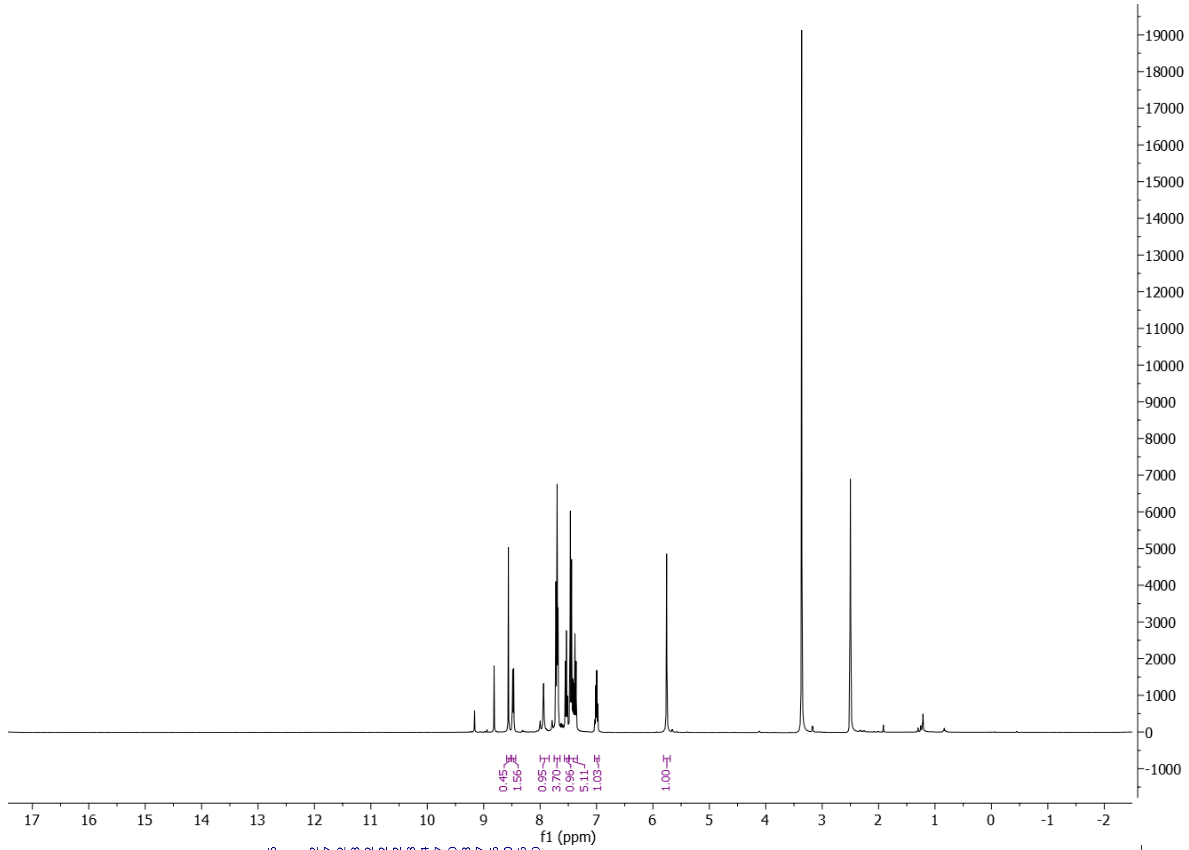
0.45
1.56
0.95
3.70
0.96
5.11
1.03
1.00
f1 (ppm)

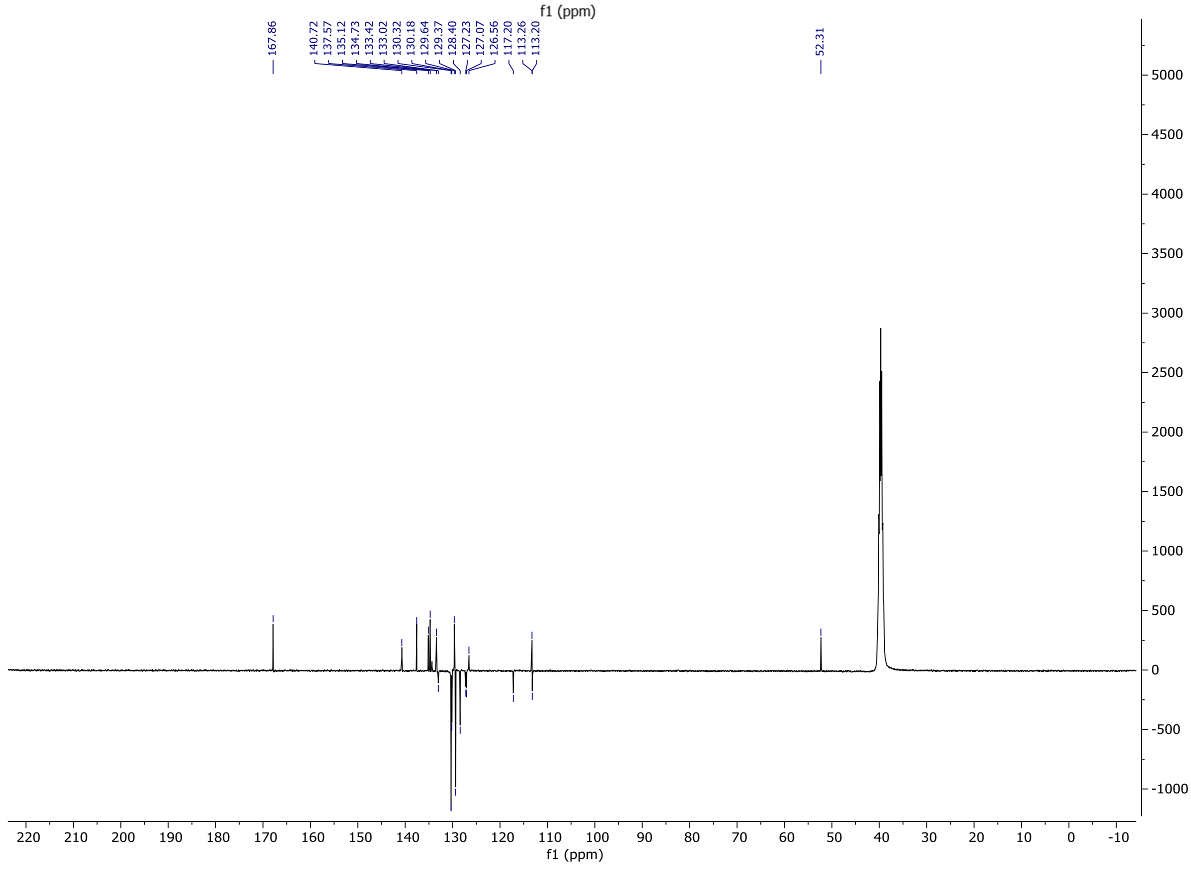
167.86
140.72
137.57
135.12
134.73
133.42
133.02
130.32
130.18
129.64
129.37
128.40
127.23
127.07
126.56
117.20
113.26
113.20
52.31
f1 (ppm)

40

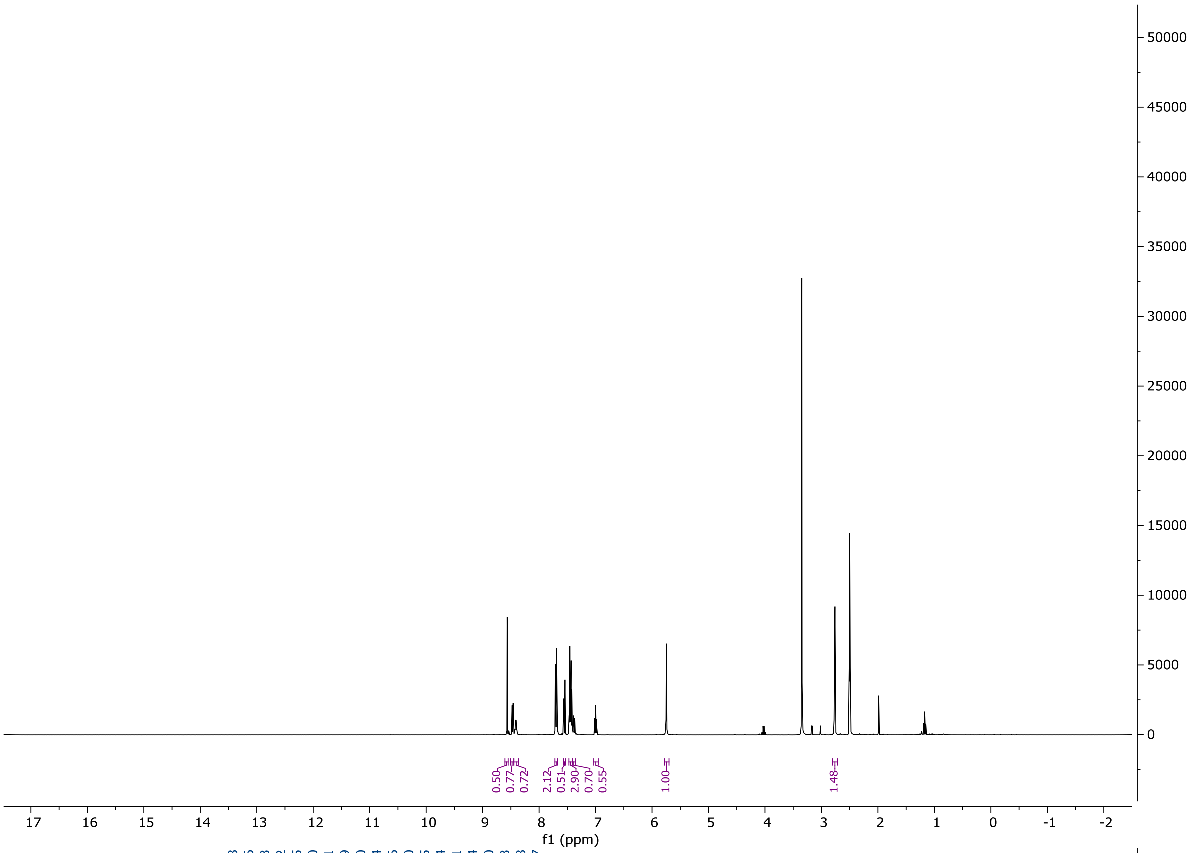

0.50
0.77
0.72
2.12
0.51
2.90
0.70
0.55
1.00
1.48
f1 (ppm)

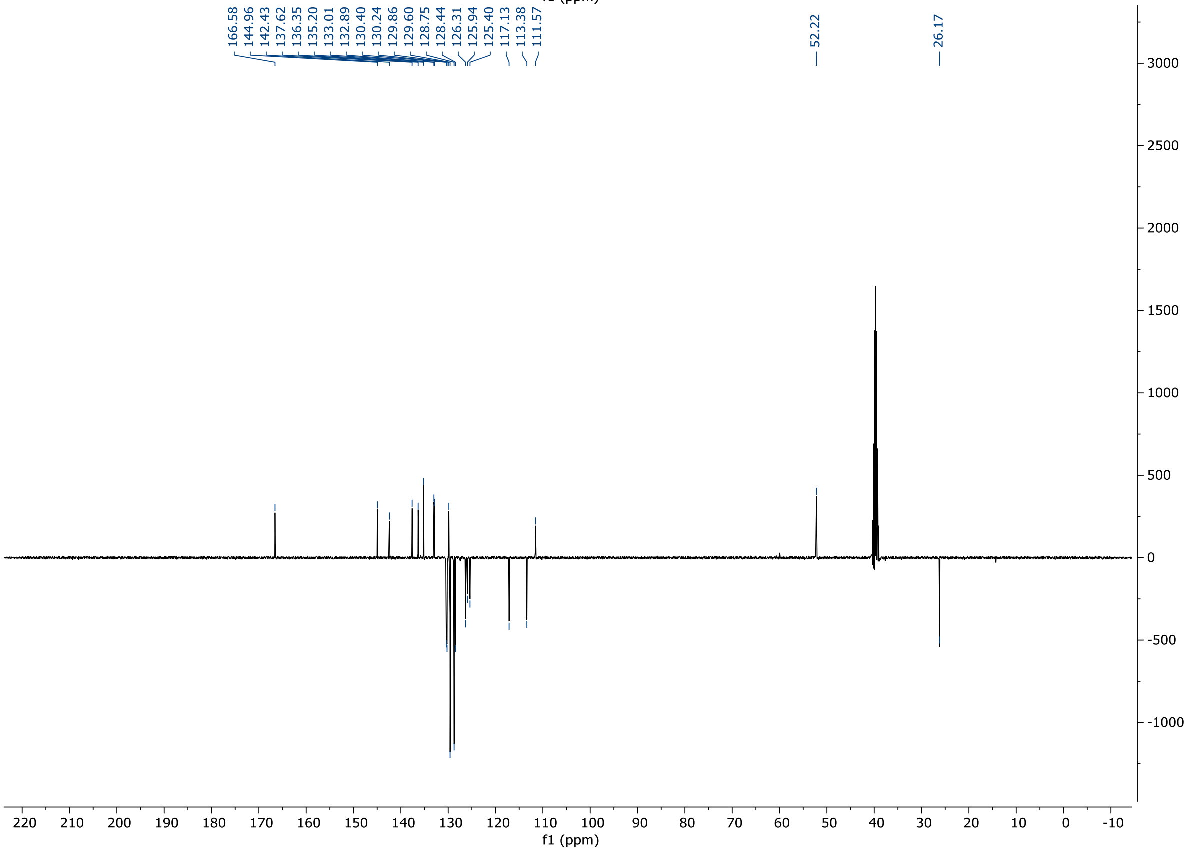

166.58
144.96
142.43
137.62
136.35
135.20
133.01
132.89
130.40
130.24
129.86
129.60
128.75
128.44
126.31
125.94
125.40
117.13
113.38
111.57
52.22
26.17
f1 (ppm)

41

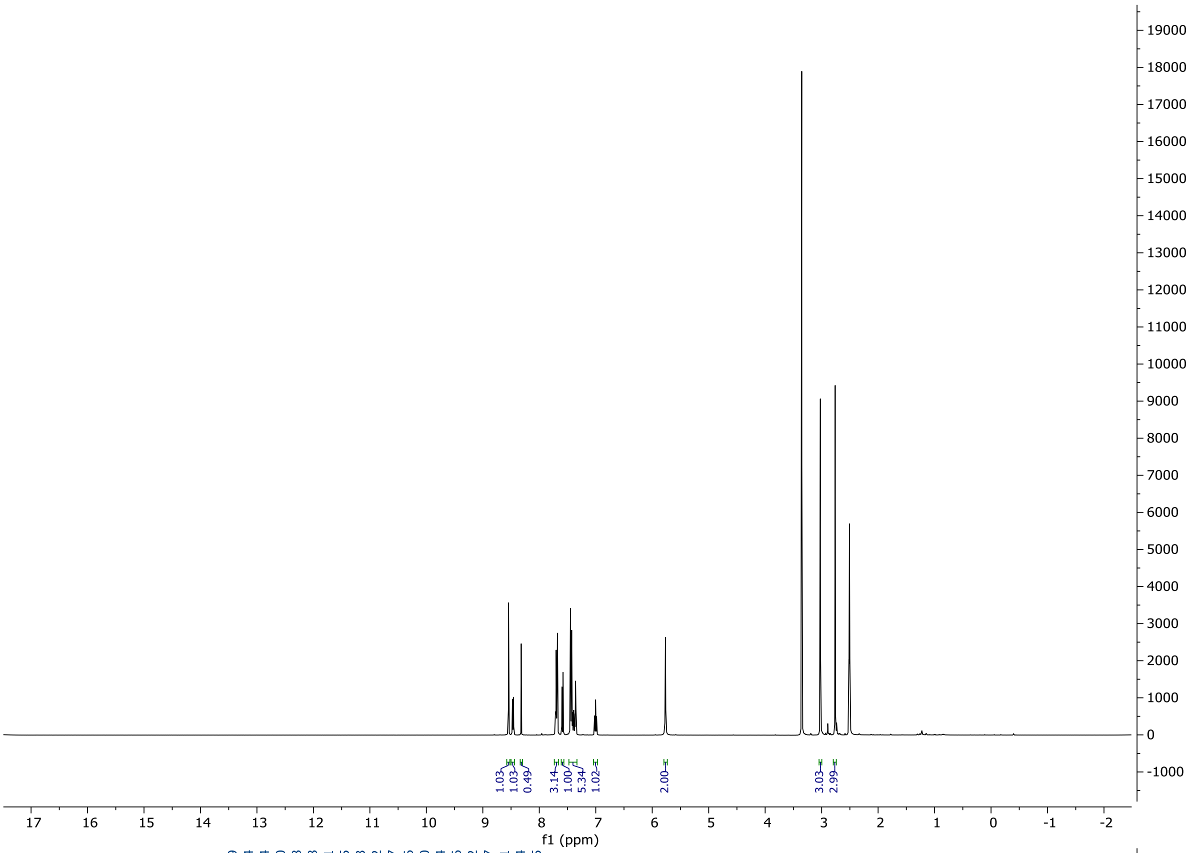
1.03
1.03
0.49
3.14
1.00
5.34
1.02
2.00
3.03
2.99
f1 (ppm)

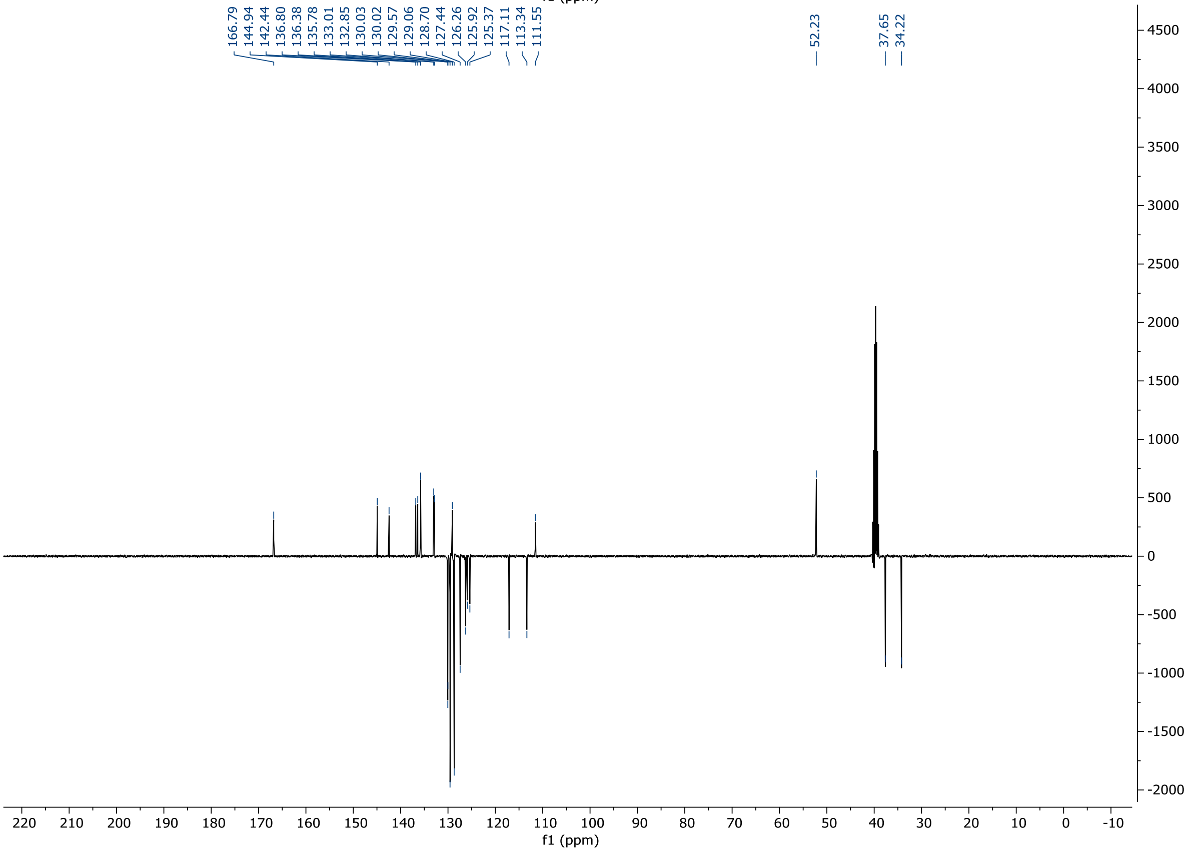
166.79
144.94
142.44
136.80
136.38
135.78
133.01
132.85
130.03
130.02
129.57
129.06
128.70
127.44
126.26
125.92
125.37
117.11
113.34
111.55
52.23
37.65
34.22
f1 (ppm)

42

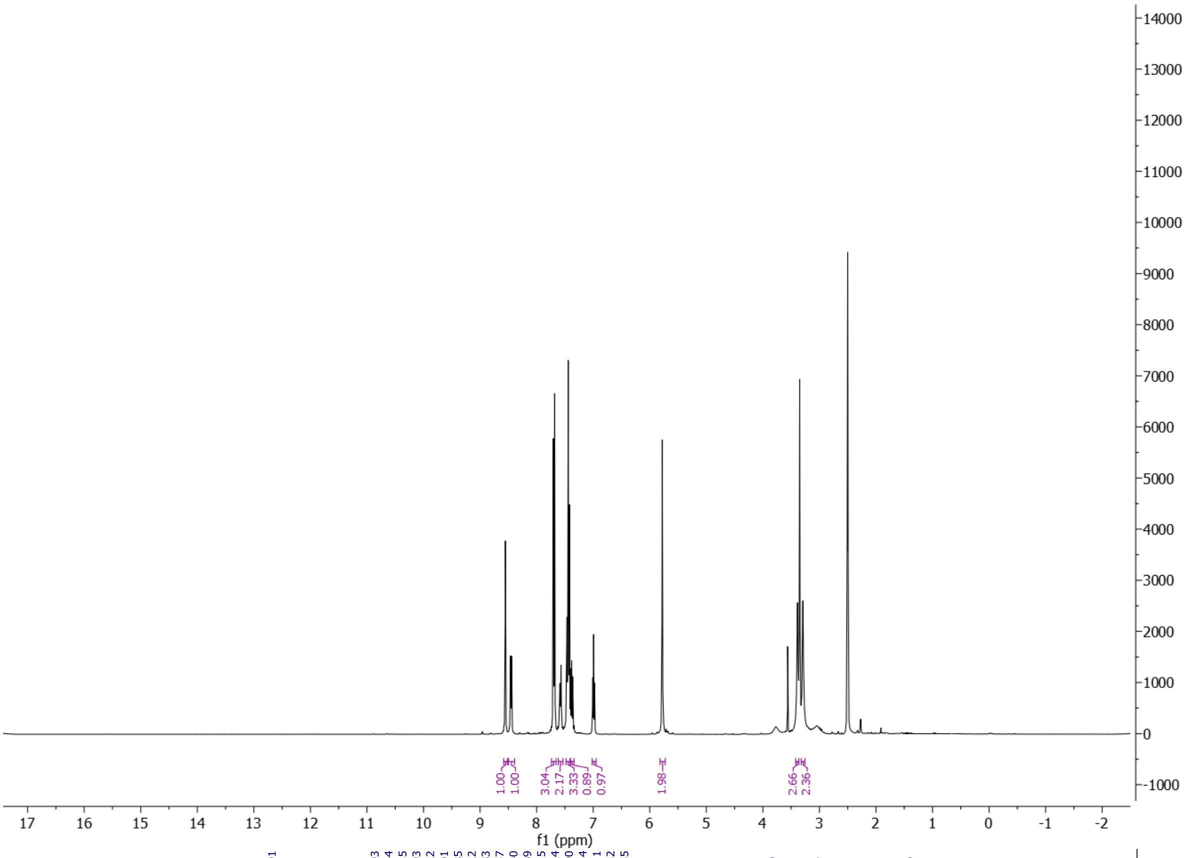
1.00
1.00
3.04
2.17
3.33
0.89
0.97
1.98
2.66
2.36
f1 (ppm)

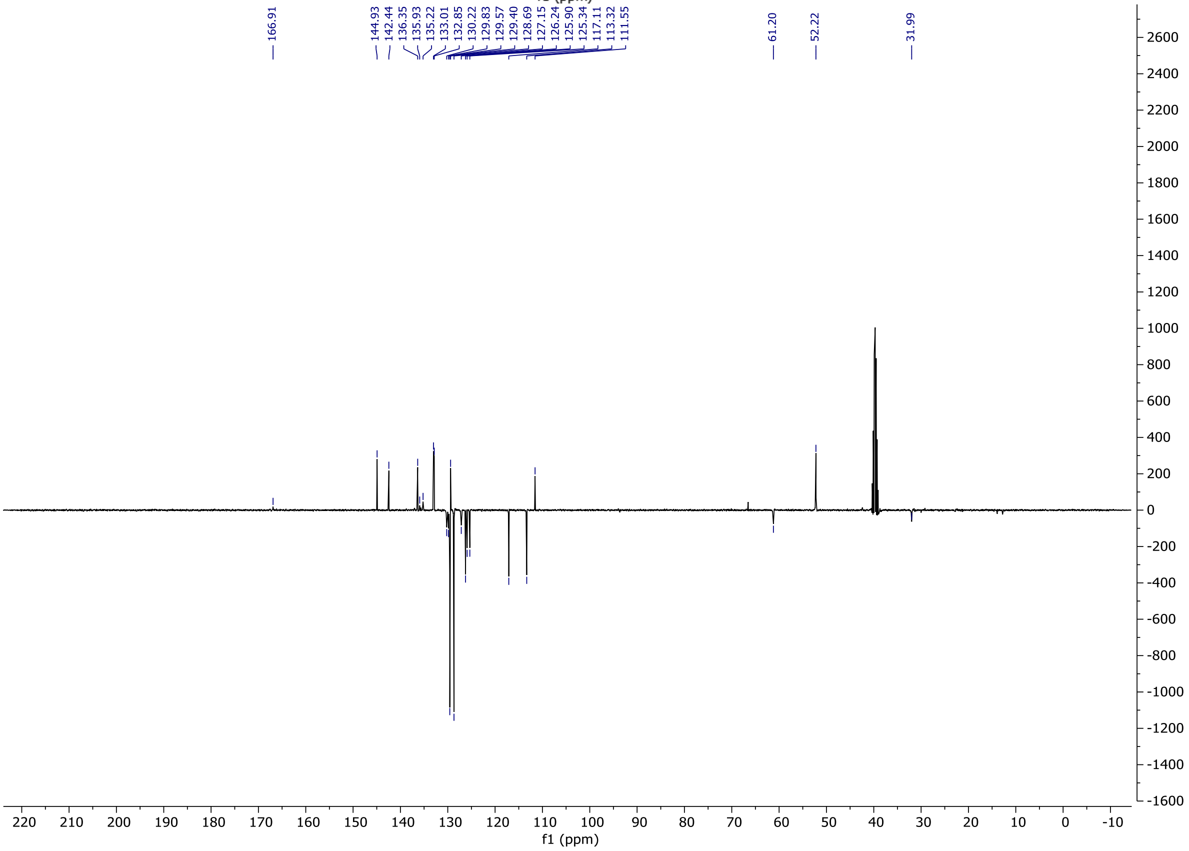
166.91
144.93
142.44
136.35
135.93
135.22
133.01
132.85
130.22
129.83
129.57
129.40
128.69
127.15
126.24
125.90
125.34
117.11
113.32
111.55
61.20
52.22
31.99
f1 (ppm)

43

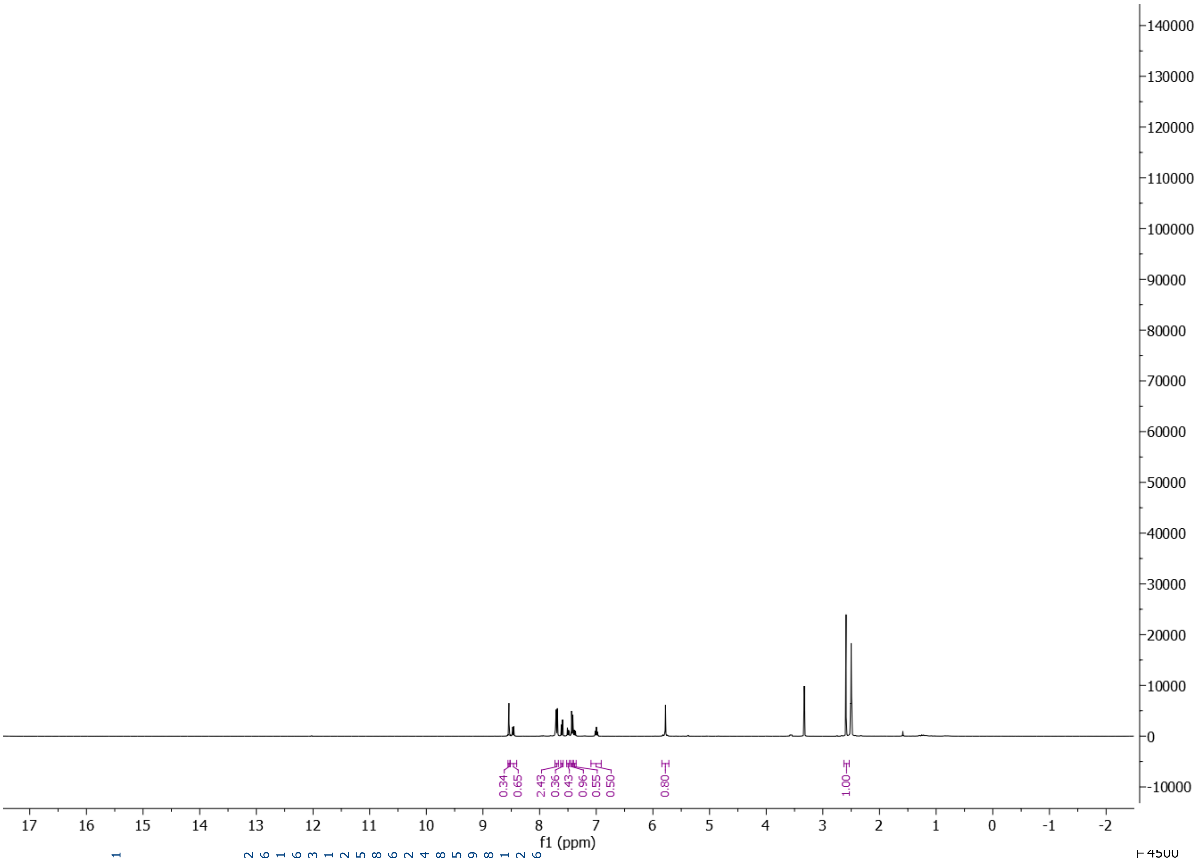
0.34
0.65
2.43
0.36
0.43
0.96
0.55
0.50
0.80
1.00
f1 (ppm)

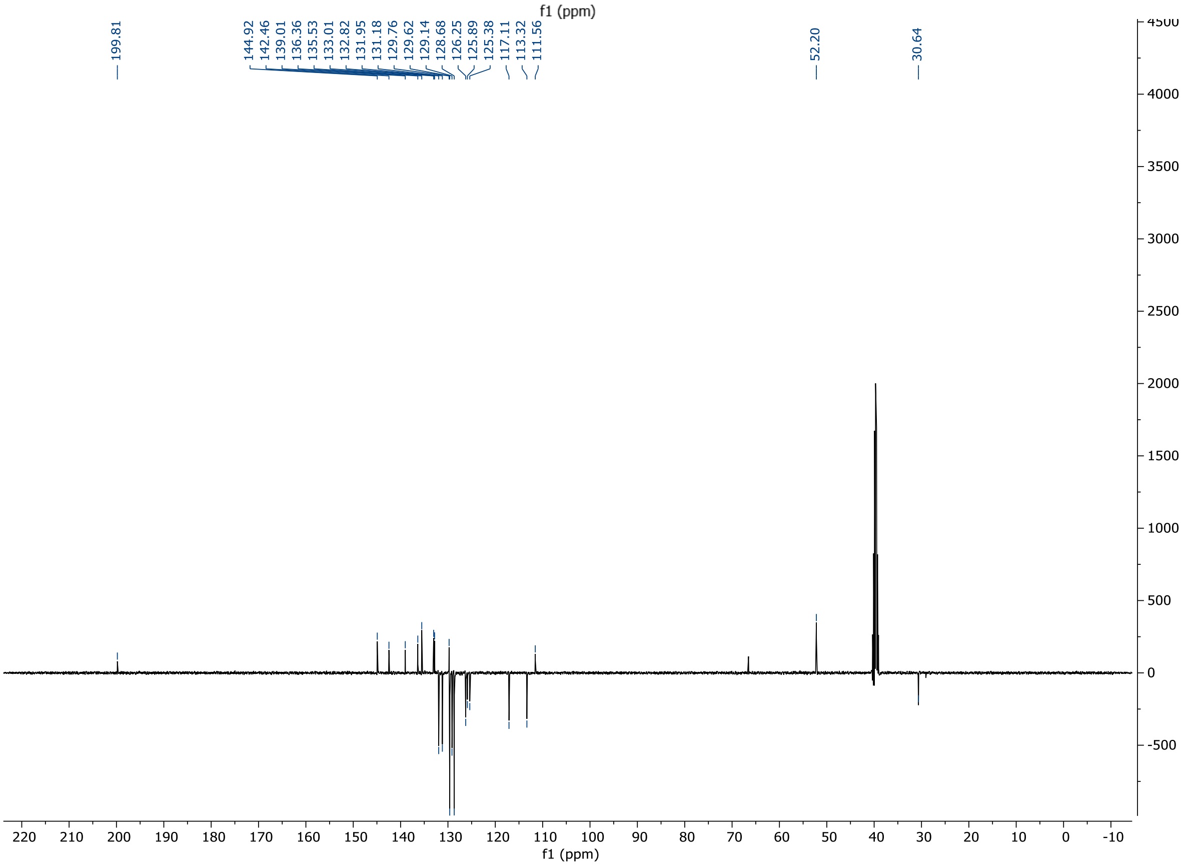
199.81
144.92
142.46
139.01
136.36
135.53
133.01
132.82
131.95
131.18
129.76
129.62
129.14
128.68
126.25
125.89
125.38
117.11
113.32
111.56
52.20
30.64
f1 (ppm)

44

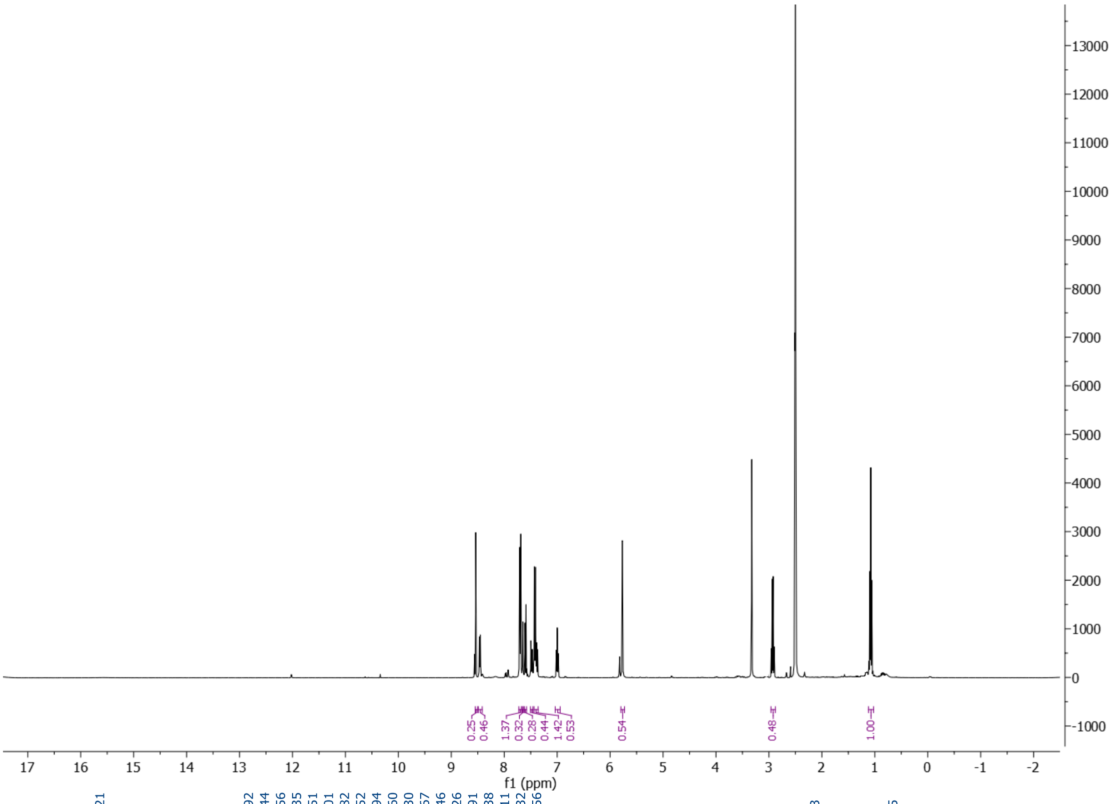
0.25
0.46
1.37
0.32
0.28
0.44
1.42
0.53
0.54
0.48
1.00
f1 (ppm)

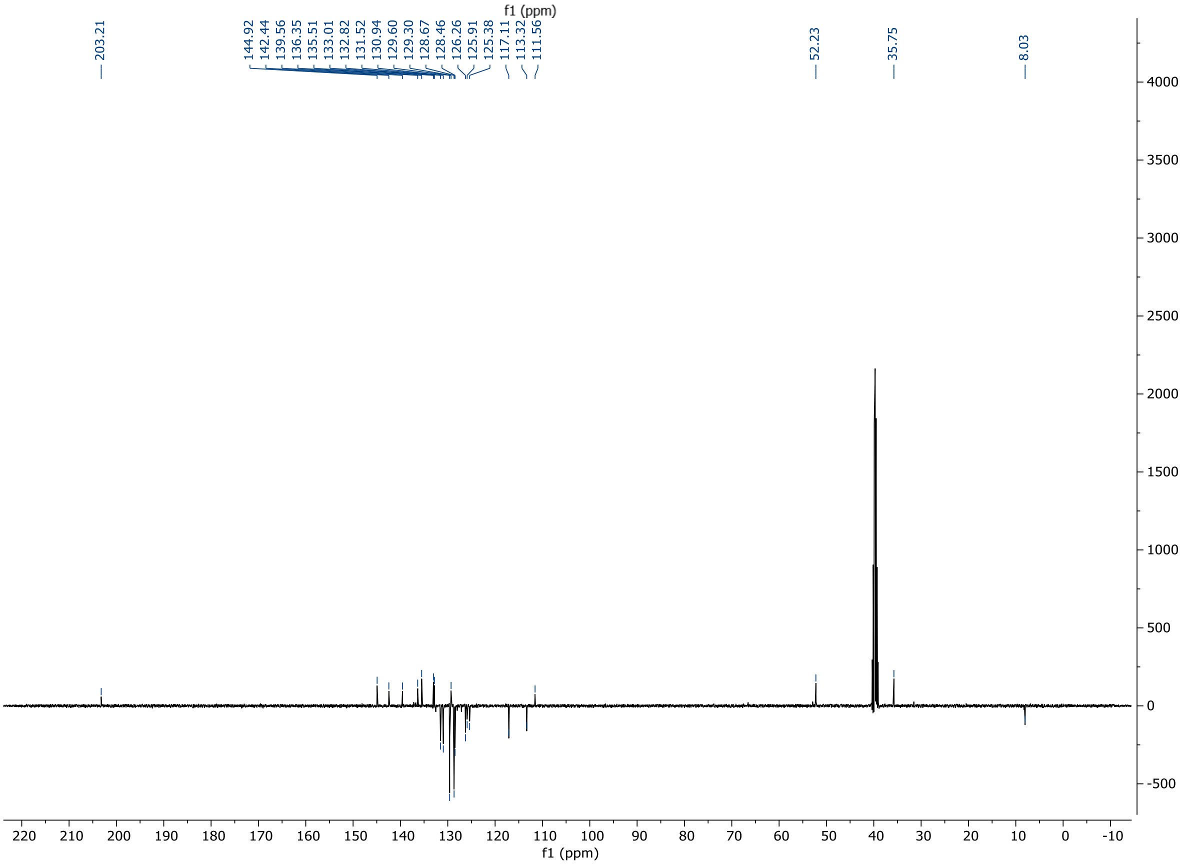
203.21
144.92
142.44
139.56
136.35
135.51
133.01
132.82
131.52
130.94
129.60
129.30
128.67
128.46
126.26
125.91
125.38
117.11
113.32
111.56
52.23
35.75
8.03
f1 (ppm)

45

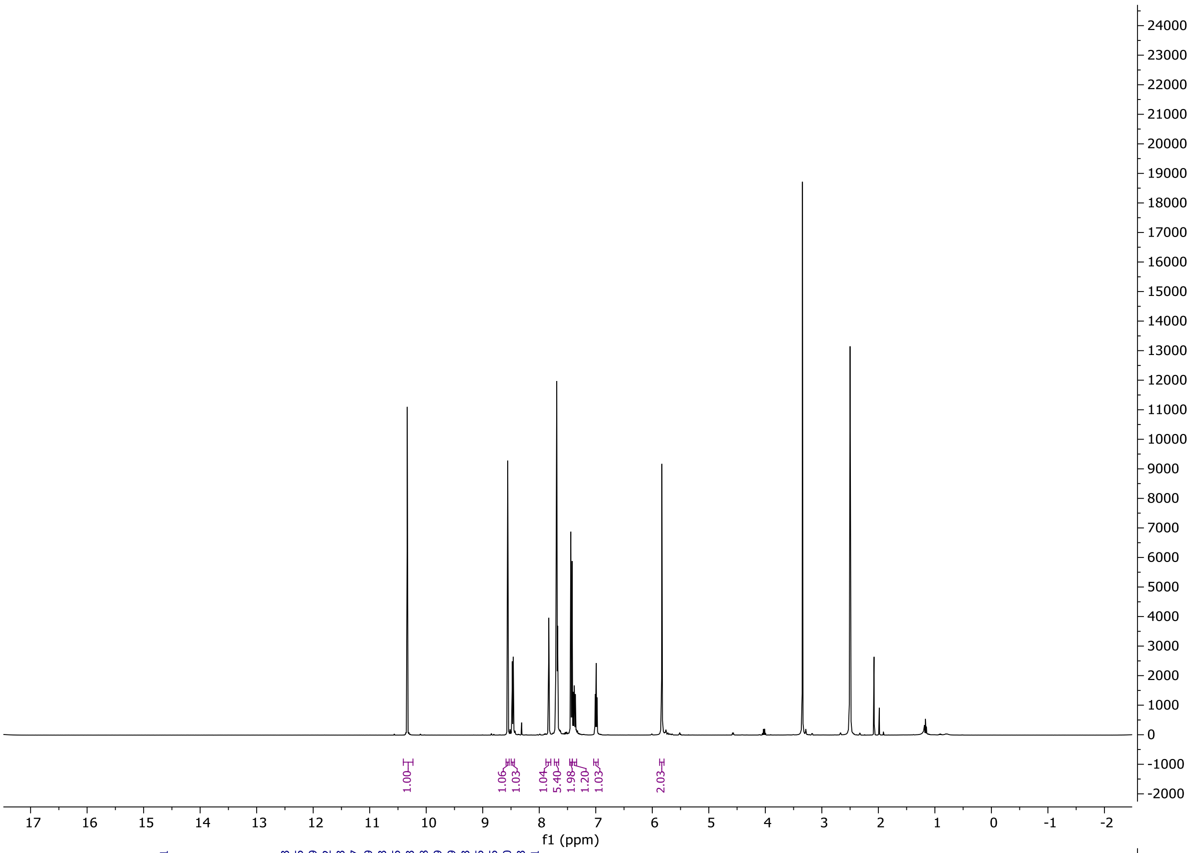

1.00
1.06
1.03
1.04
5.40
1.98
1.20
1.03
2.03
f1 (ppm)

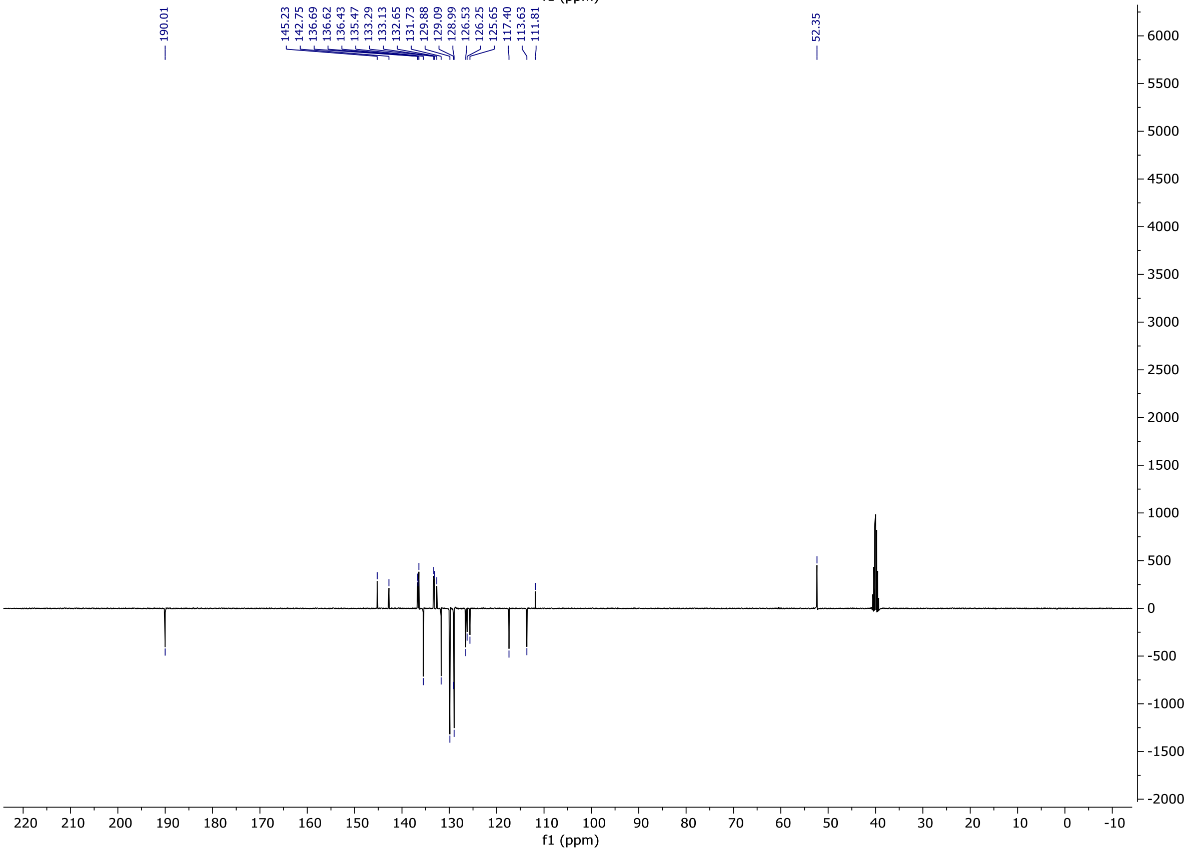

190.01
145.23
142.75
136.69
136.62
136.43
135.47
133.29
133.13
132.65
131.73
129.88
129.09
128.99
126.53
126.25
125.65
117.40
113.63
111.81
52.35
f1 (ppm)

46

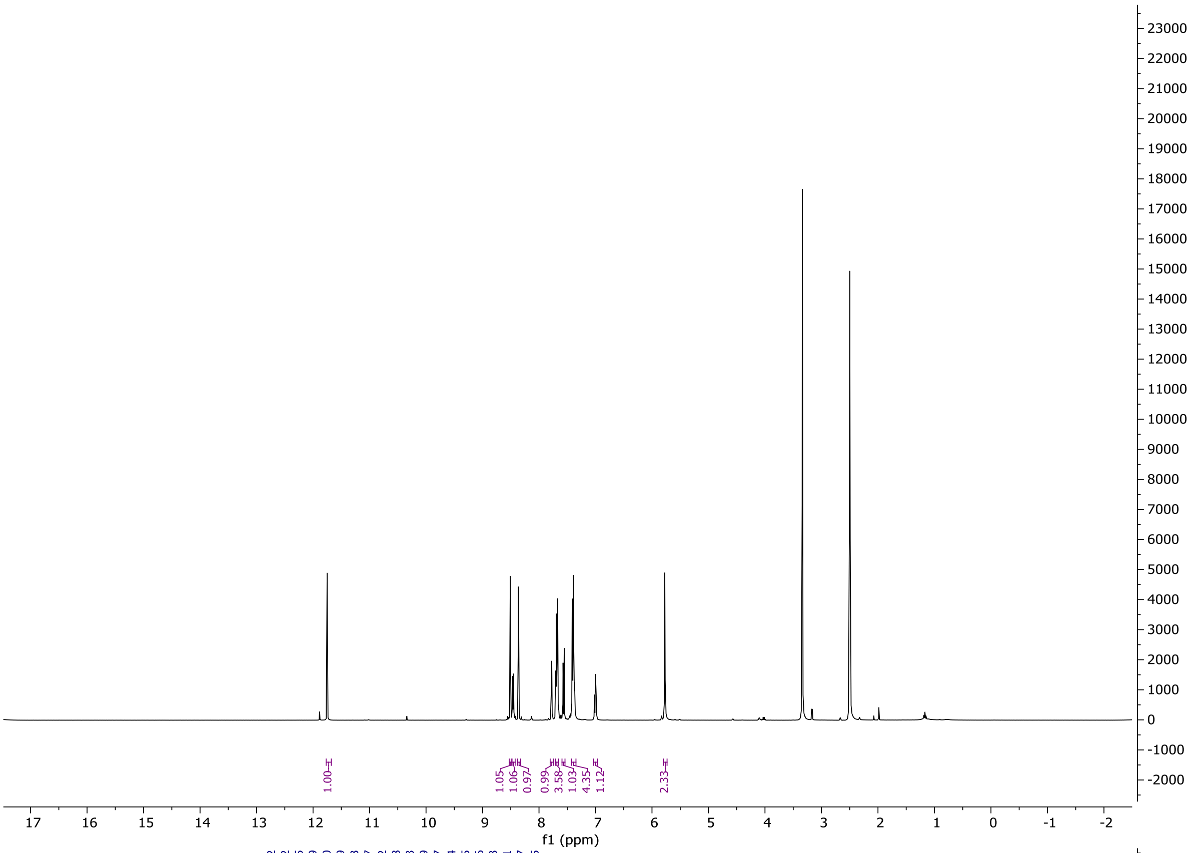

1.00
1.05
1.06
0.97
0.99
3.58
1.03
4.35
1.12
2.33
f1 (ppm)

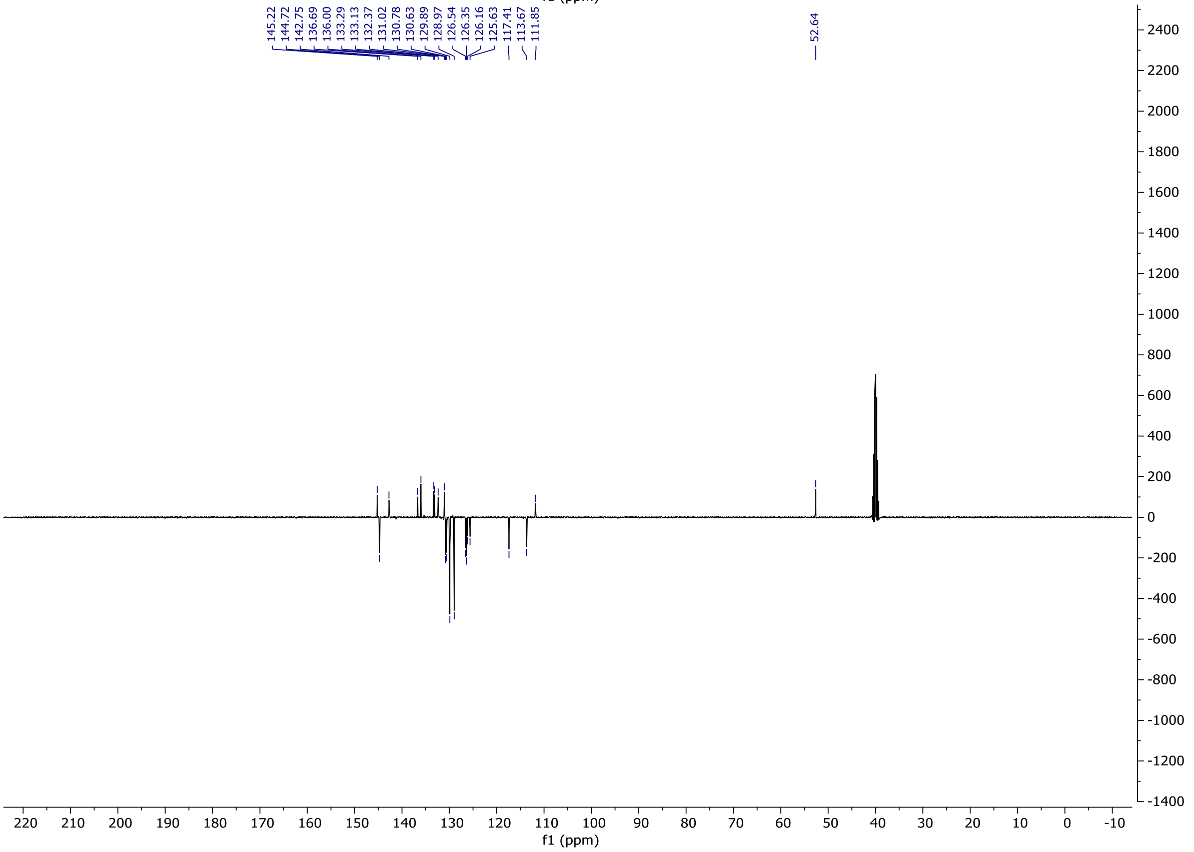

145.22
144.72
142.75
136.69
136.00
133.29
133.13
132.37
131.02
130.78
130.63
129.89
128.97
126.54
126.35
126.16
125.63
117.41
113.67
111.85
52.64
f1 (ppm)

47

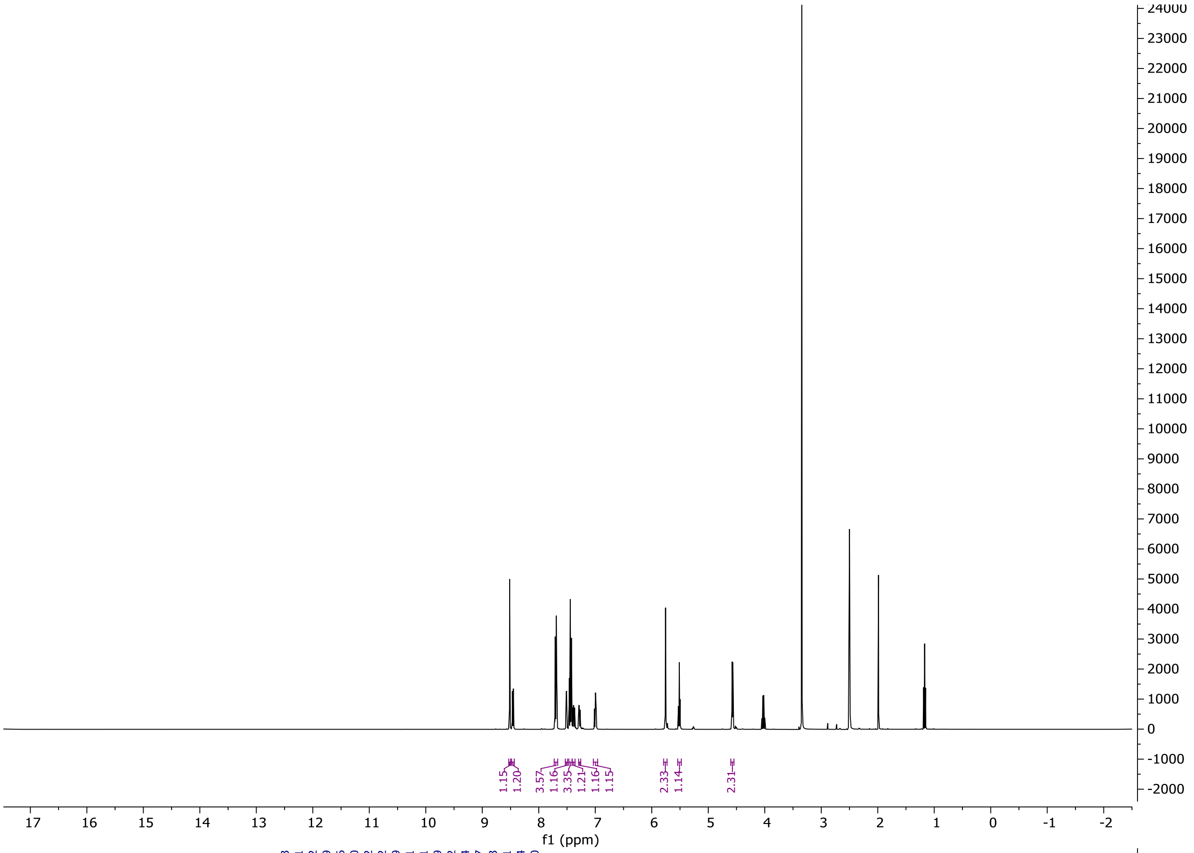
1.15
1.20
3.57
1.16
3.35
1.21
1.16
1.15
2.33
1.14
2.31
f1 (ppm)

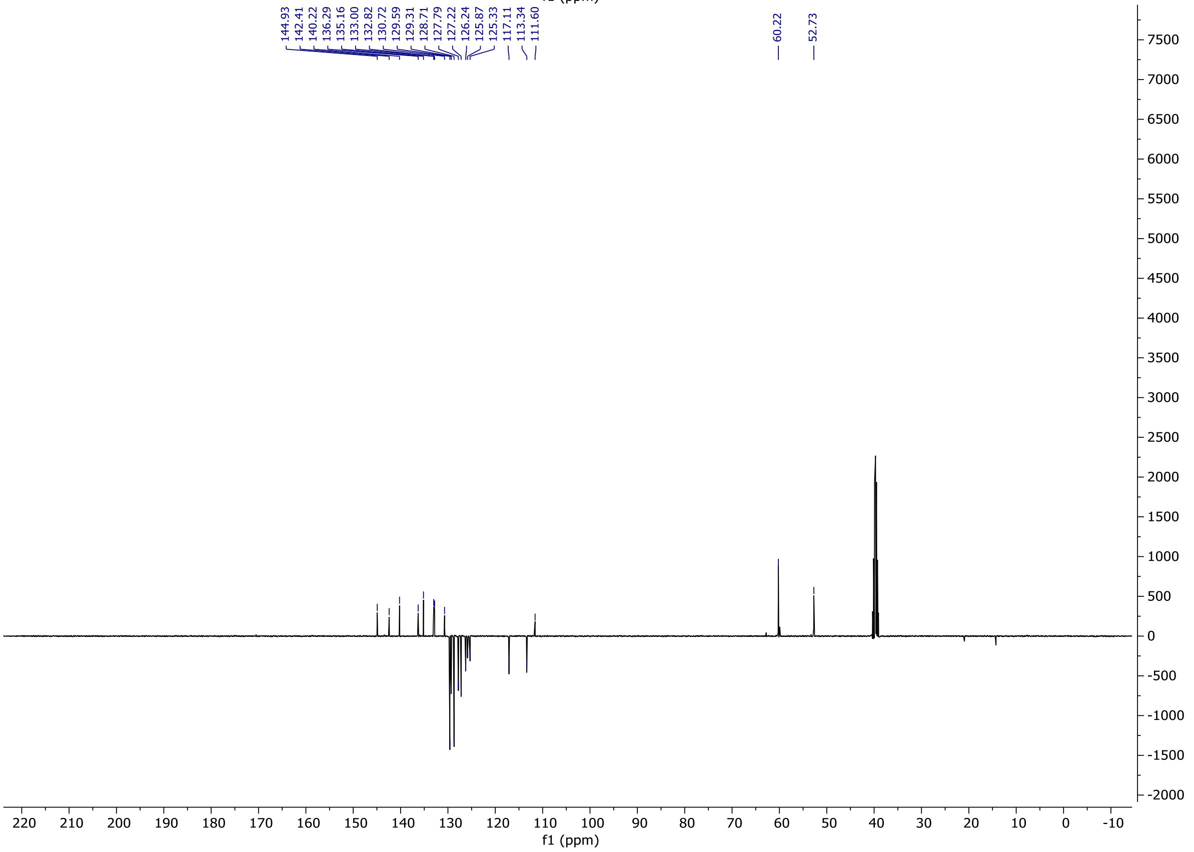
144.93
142.41
140.22
136.29
135.16
133.00
132.82
130.72
129.59
129.31
128.71
127.79
127.22
126.24
125.87
125.33
117.11
113.34
111.60
60.22
52.73
f1 (ppm)

48

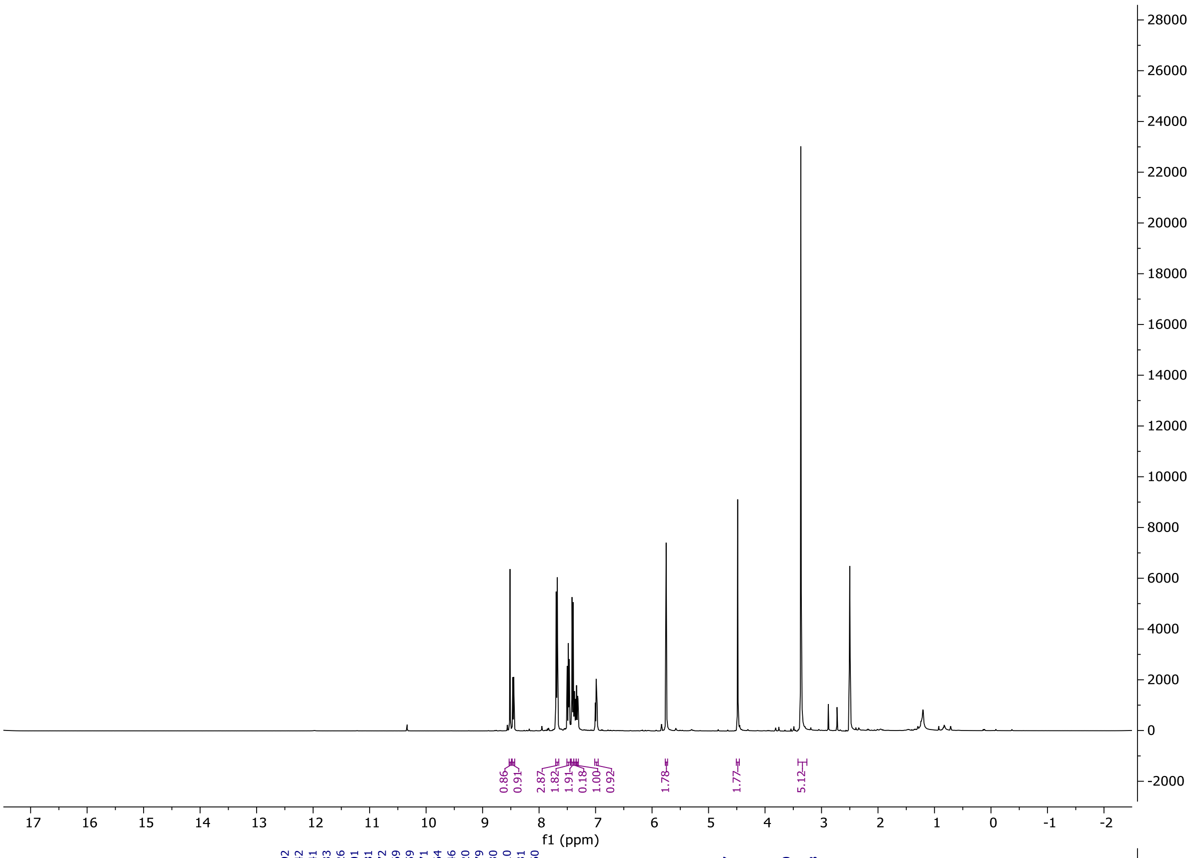

0.86
0.91
2.87
1.82
1.91
0.18
1.00
0.92
1.78
1.77
5.12
f1 (ppm)

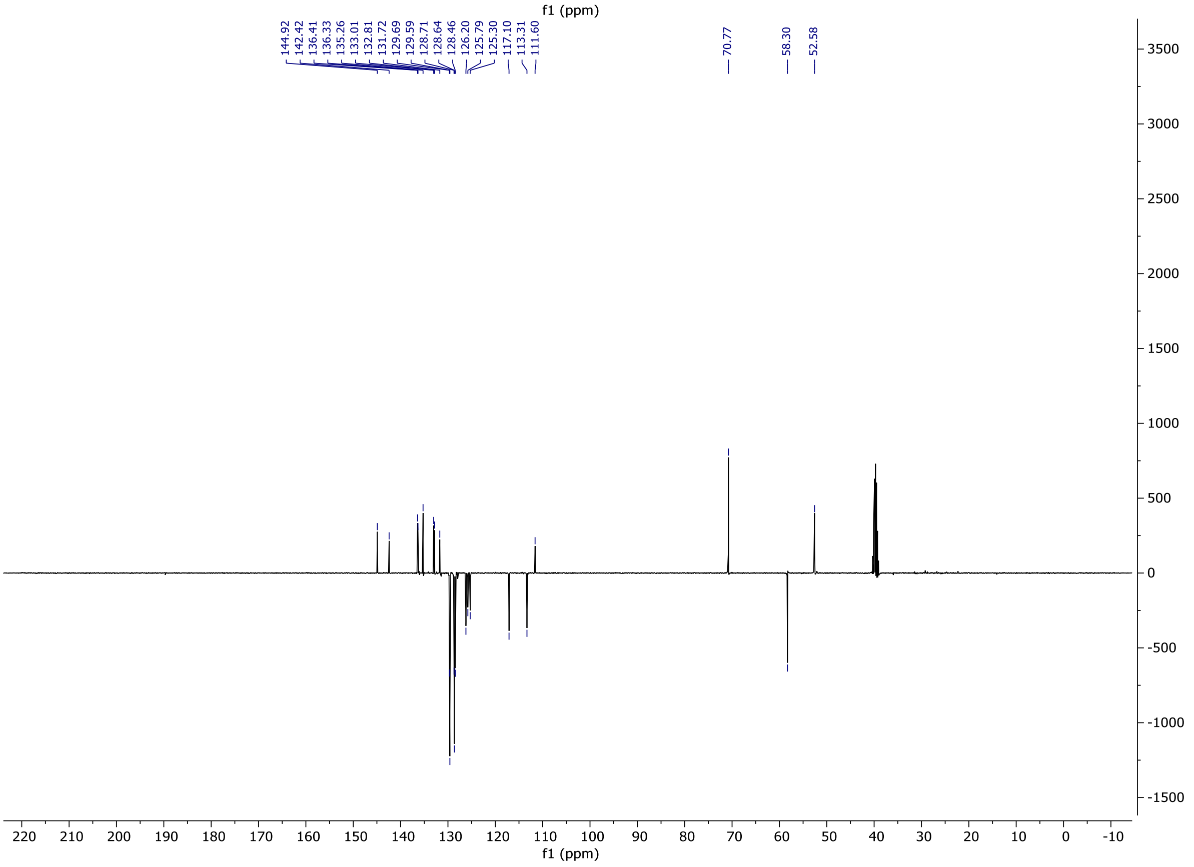

144.92
142.42
136.41
136.33
135.26
133.01
132.81
131.72
129.69
129.59
128.71
128.64
128.46
126.20
125.79
125.30
117.10
113.31
111.60
70.77
58.30
52.58
f1 (ppm)

49

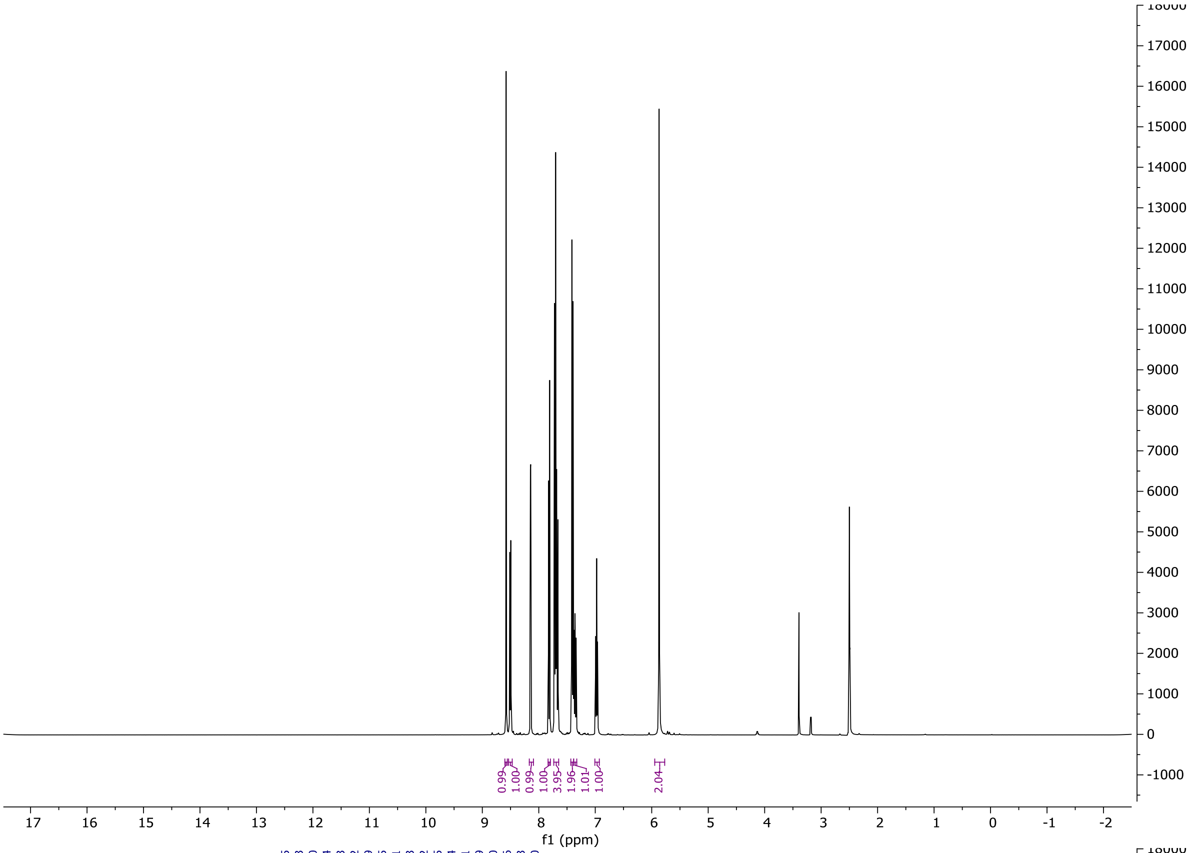

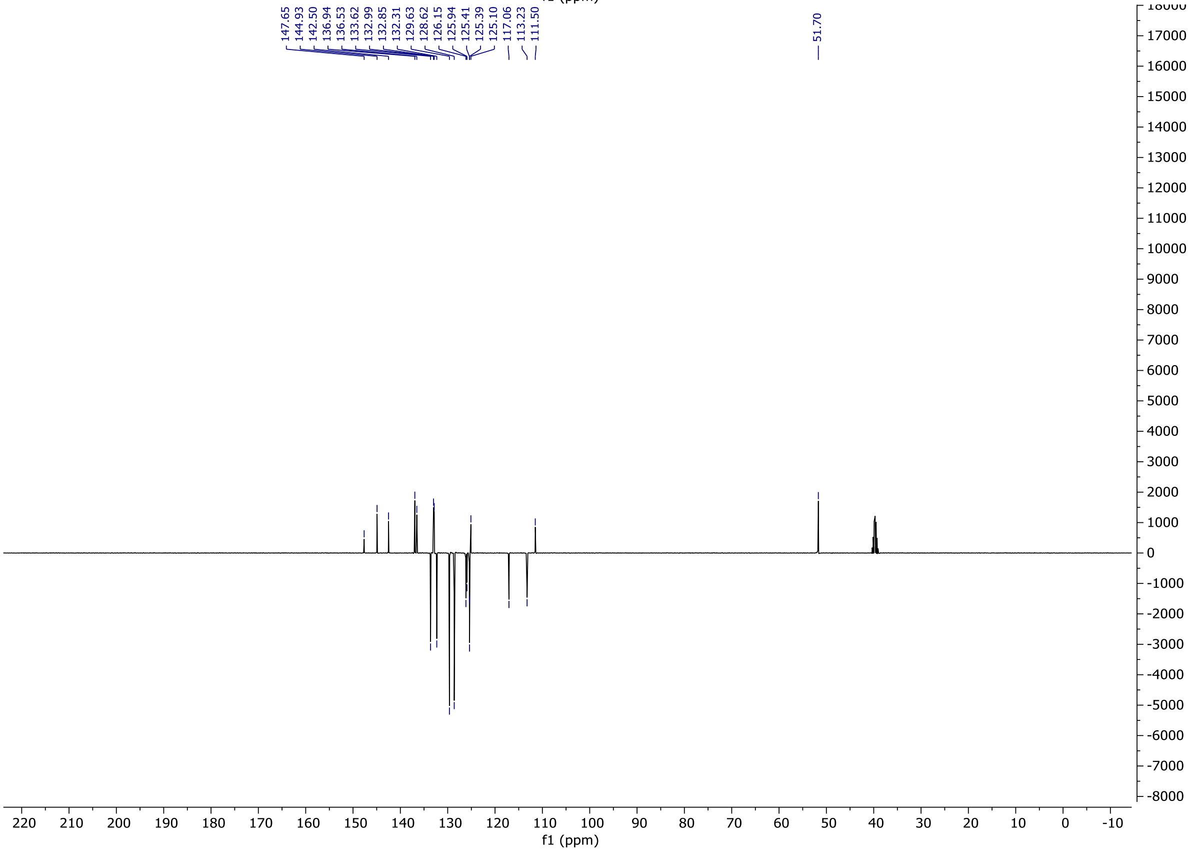

50

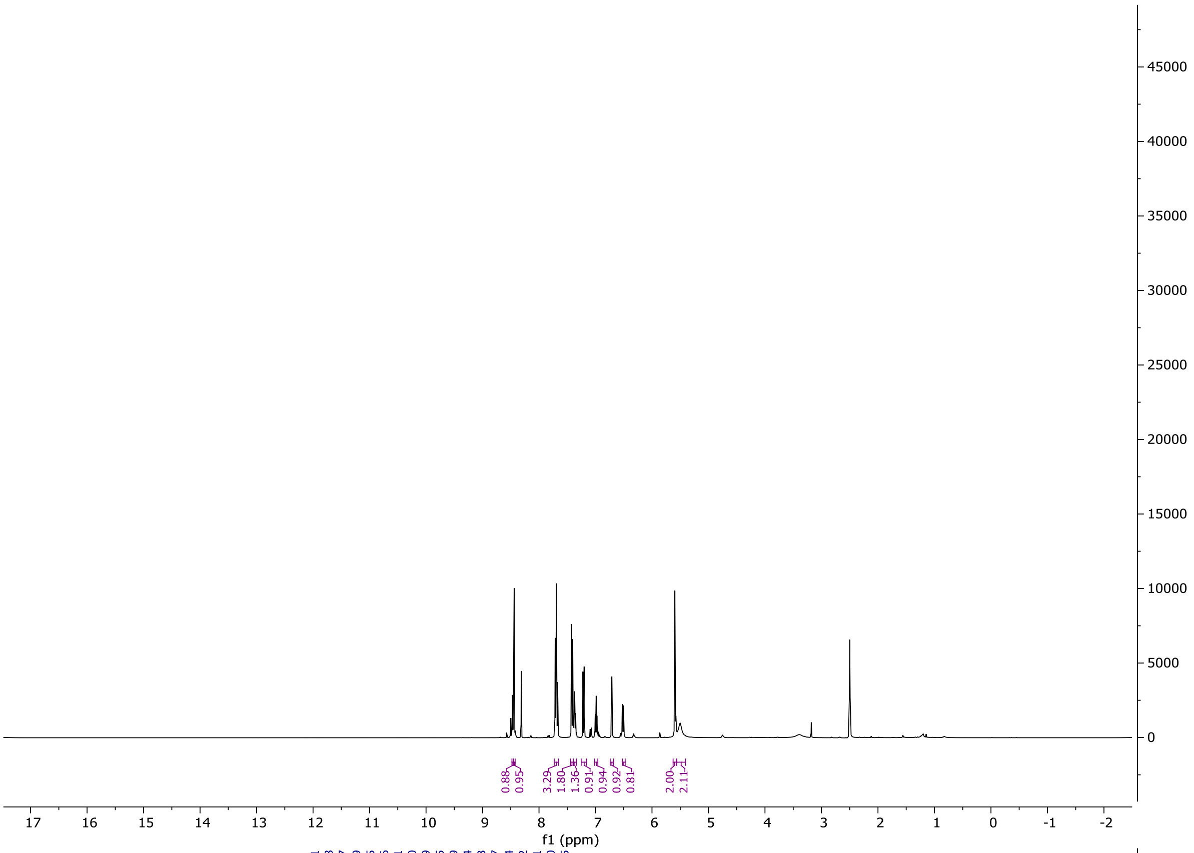
0.88
0.95
3.29
1.80
1.36
0.91
0.94
0.92
0.81
2.00
2.11
f1 (ppm)

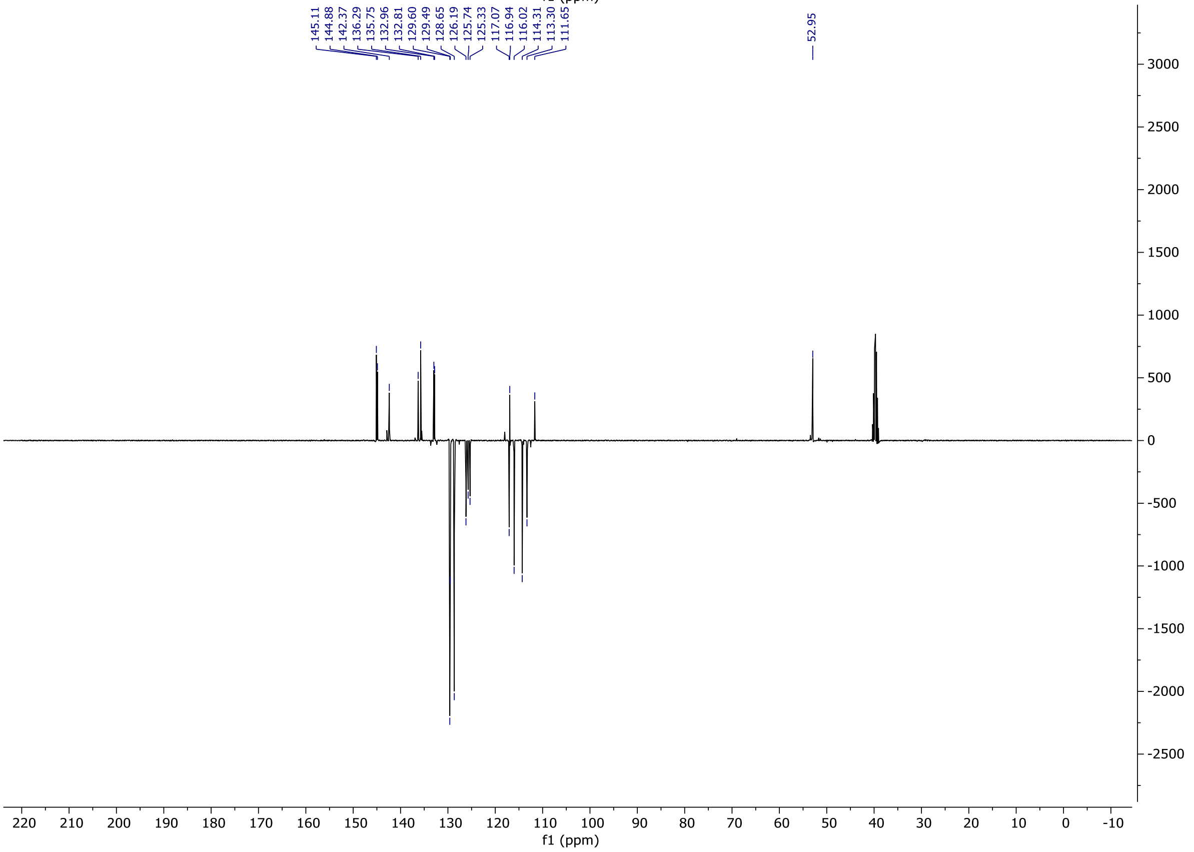
145.11
144.88
142.37
136.29
135.75
132.96
132.81
129.60
129.49
128.65
126.19
125.74
125.33
117.07
116.94
116.02
114.31
113.30
111.65
52.95
f1 (ppm)

51

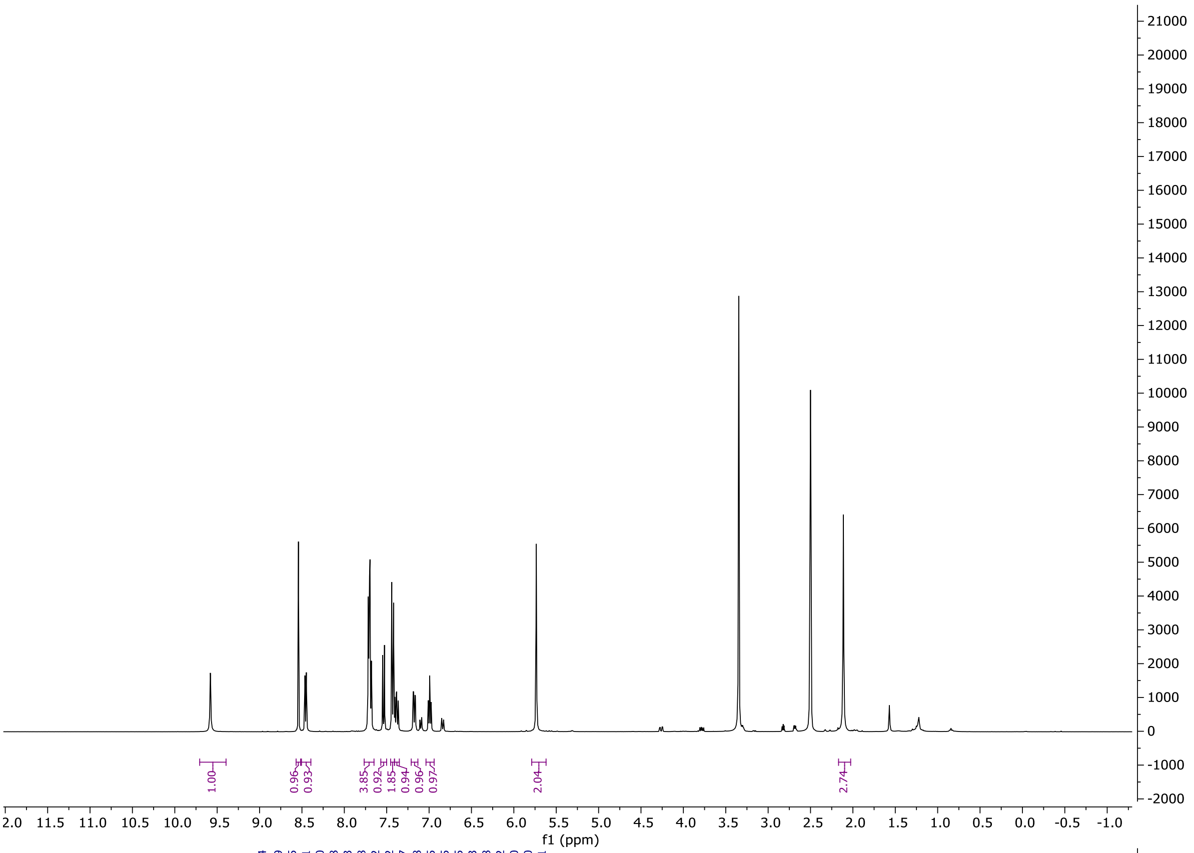

1.00
0.96
0.93
3.85
0.92
1.85
0.94
0.96
0.97
2.04
2.74
f1 (ppm)

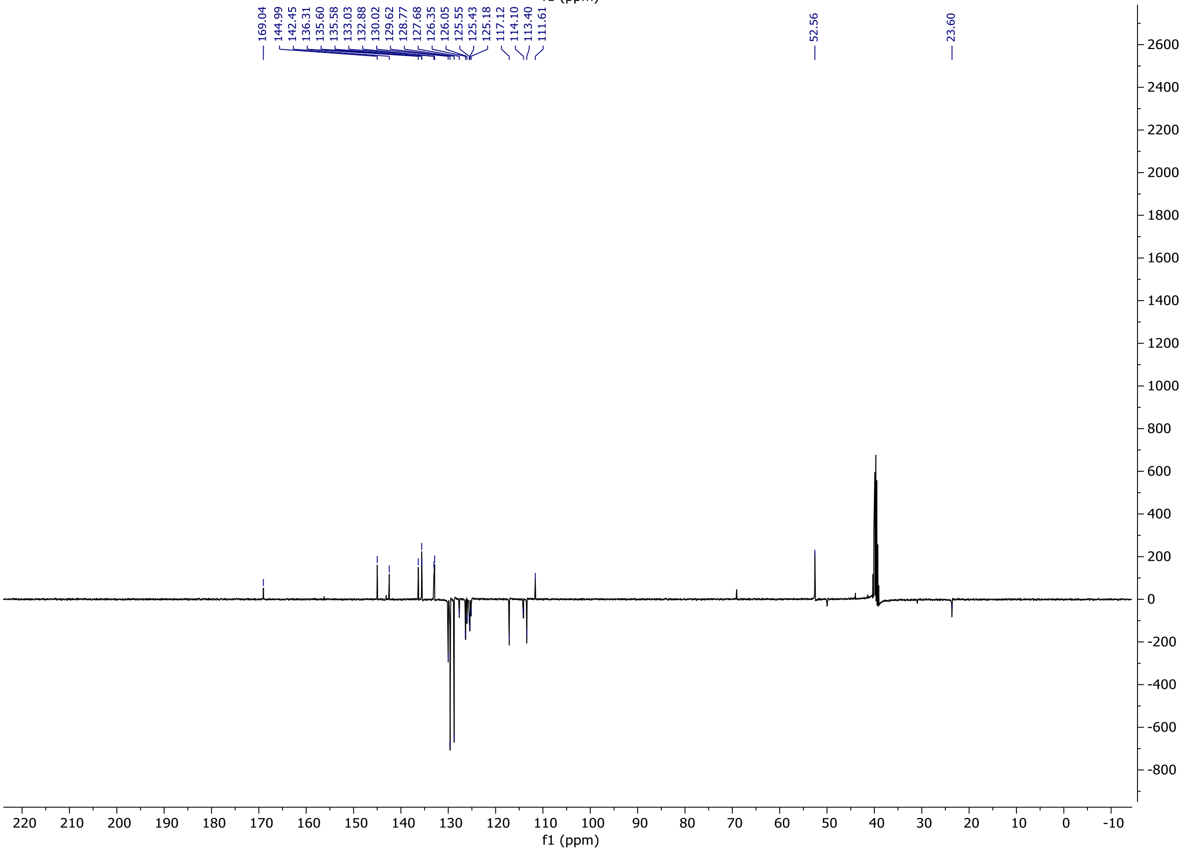

169.04
144.99
142.45
136.31
135.60
135.58
133.03
132.88
130.02
129.62
128.77
127.68
126.35
126.05
125.55
125.43
125.18
117.12
114.10
113.40
111.61
52.56
23.60
f1 (ppm)

55

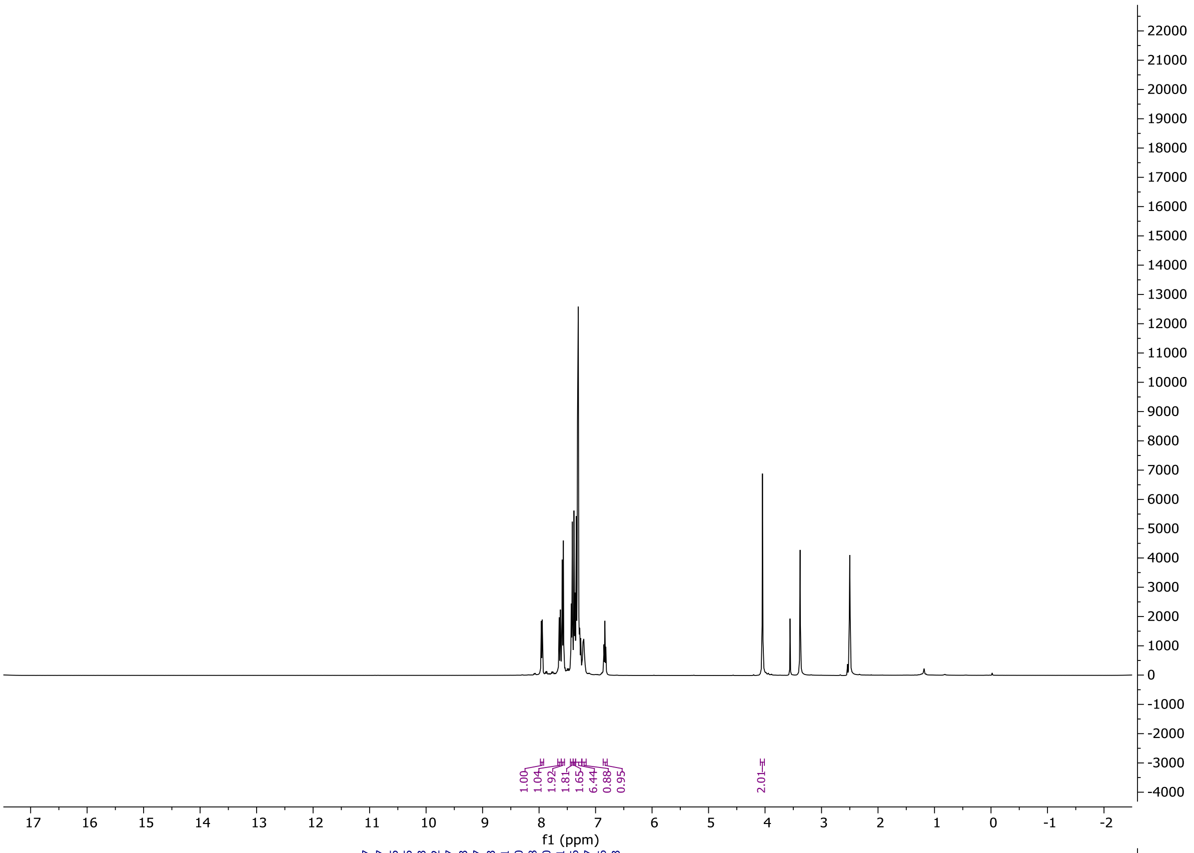
1.00
1.04
1.92
1.81
1.65
6.44
0.88
0.95
2.01
f1 (ppm)

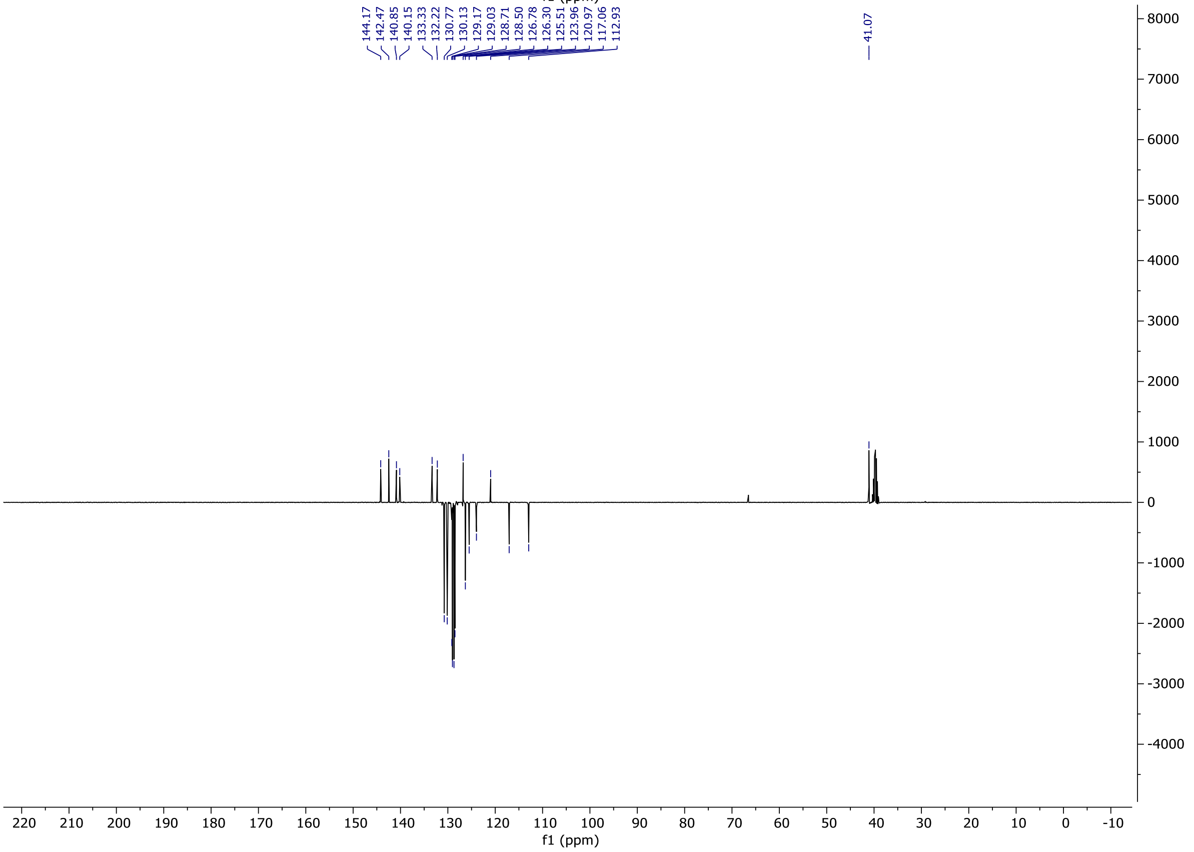
144.17
142.47
140.85
140.15
133.33
132.22
130.77
130.13
129.17
129.03
128.71
128.50
126.78
126.30
125.51
123.96
120.97
117.06
112.93
41.07
f1 (ppm)

56

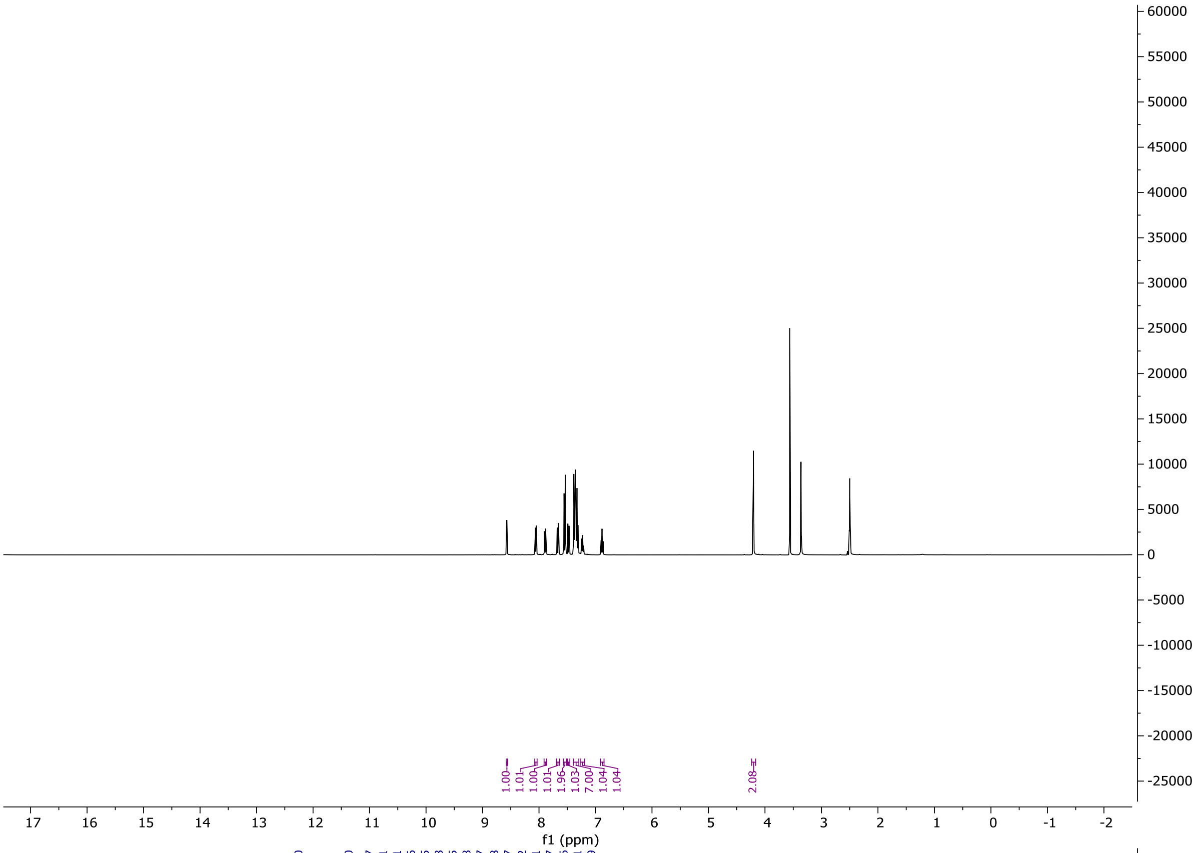
1.00
1.01
1.00
1.01
1.96
1.03
7.00
1.04
1.04
2.08
f1 (ppm)

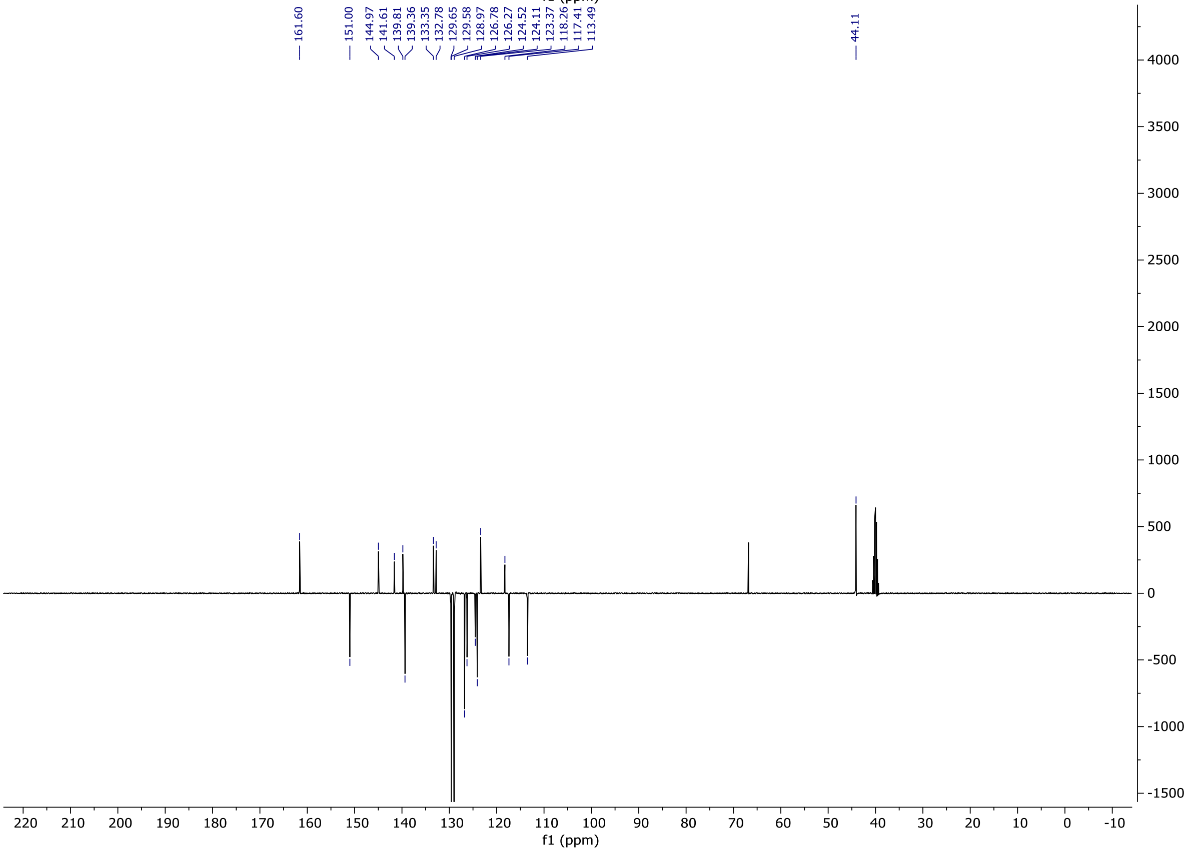
161.60
151.00
144.97
141.61
139.81
139.36
133.35
132.78
129.65
129.58
128.97
126.78
126.27
124.52
124.11
123.37
118.26
117.41
113.49
44.11
f1 (ppm)

60

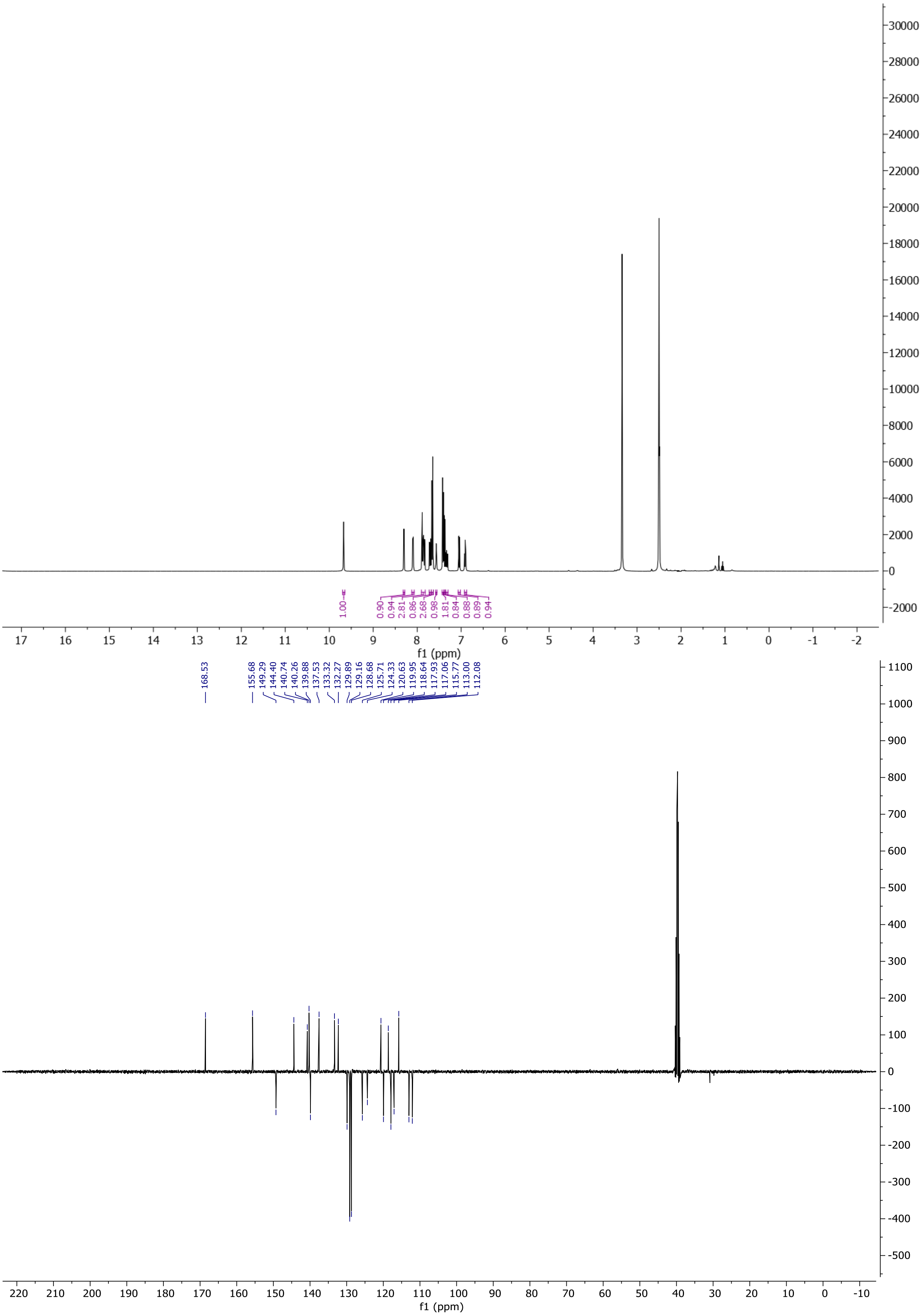
1.00
0.90
0.94
2.81
0.86
2.68
0.98
1.81
0.84
0.88
0.89
0.94
f1 (ppm)
168.53
155.68
149.29
144.40
140.74
140.26
139.88
137.53
133.32
132.27
129.89
129.16
128.68
125.71
124.33
120.63
119.95
118.64
117.93
117.06
115.77
113.00
112.08
f1 (ppm)

61

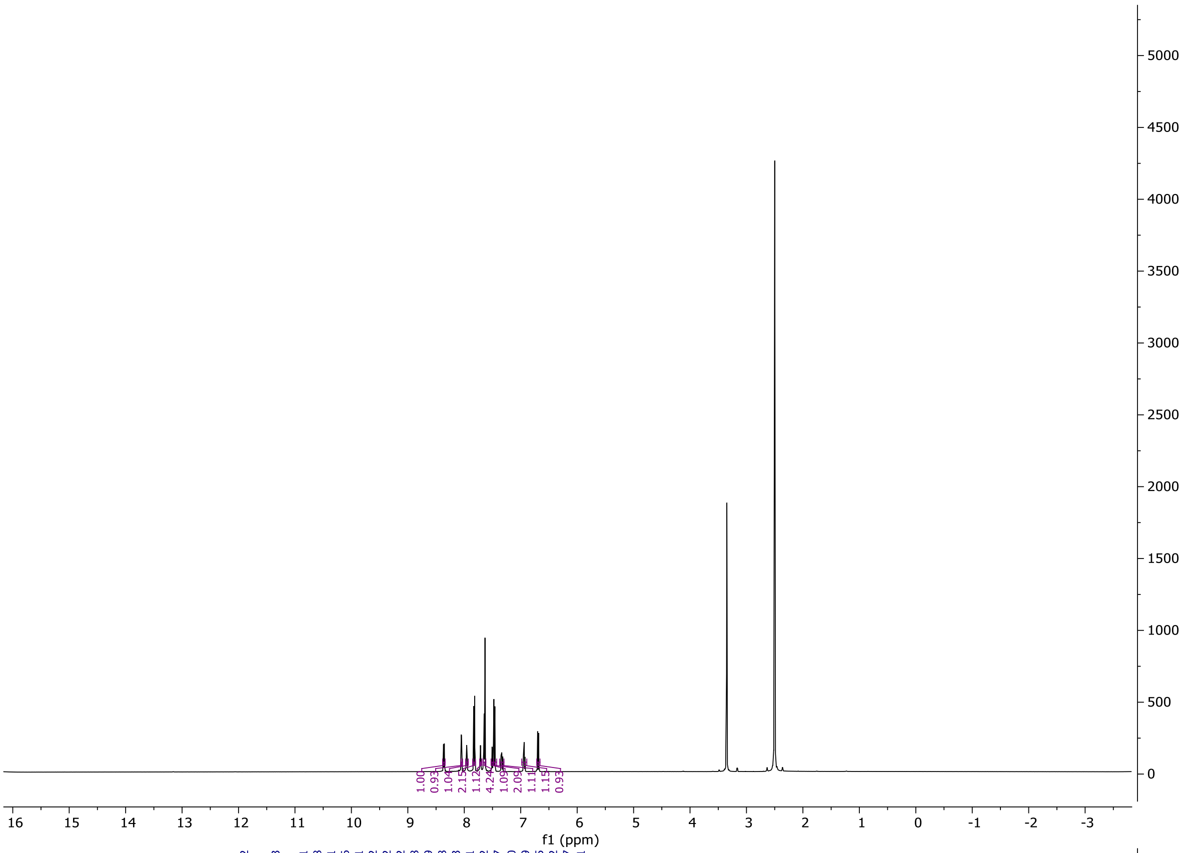
1.00
0.93
1.04
2.15
1.12
4.24
1.09
2.09
1.10
1.15
0.93
f1 (ppm)

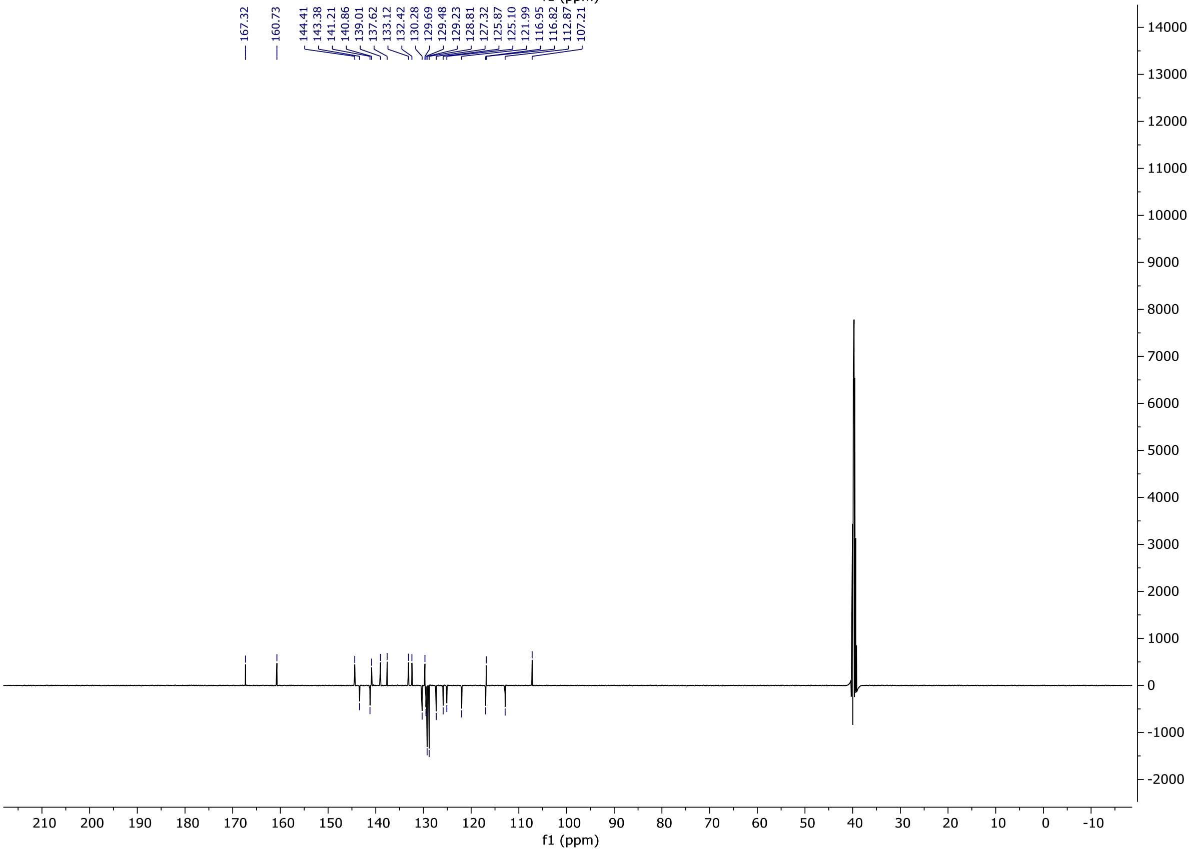
167.32
160.73
144.41
143.38
141.21
140.86
139.01
137.62
133.12
132.42
130.28
129.69
129.48
129.23
128.81
127.32
125.87
125.10
121.99
116.95
116.82
112.87
107.21
f1 (ppm)

15. SI HPLC

Compound **37**

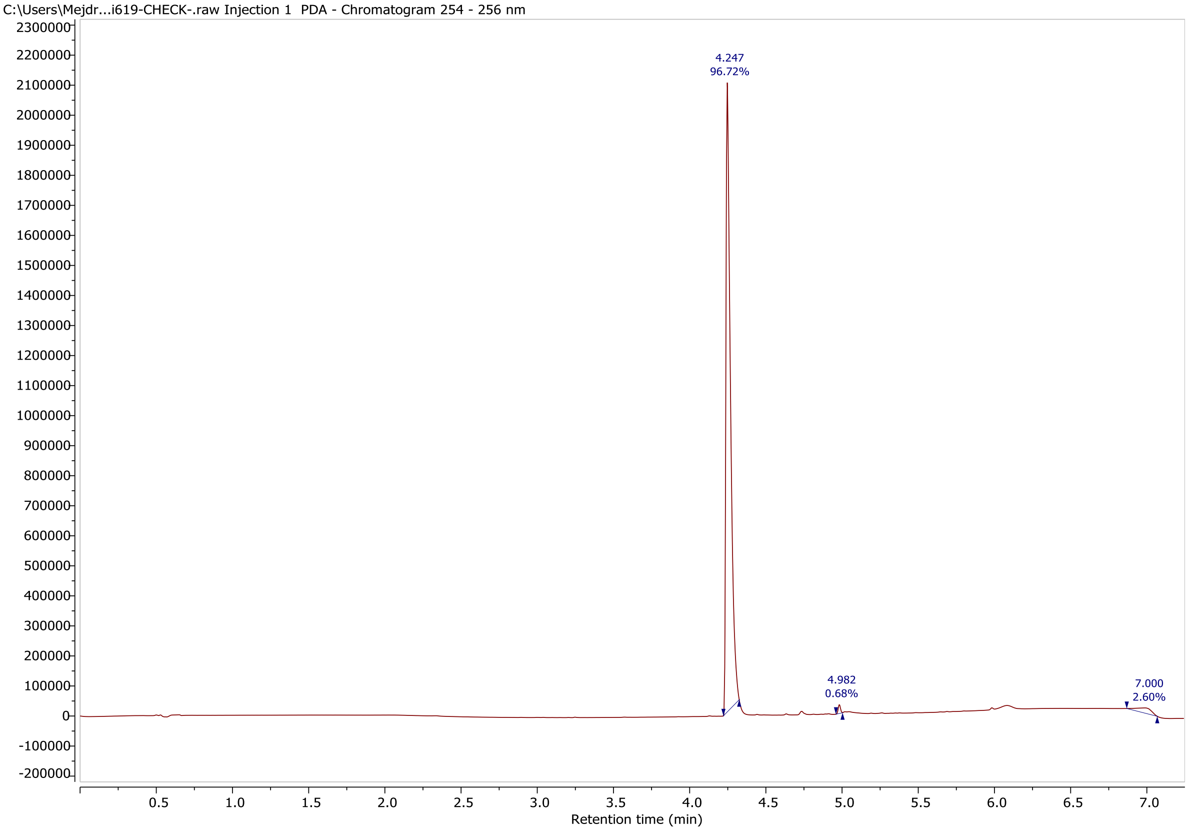

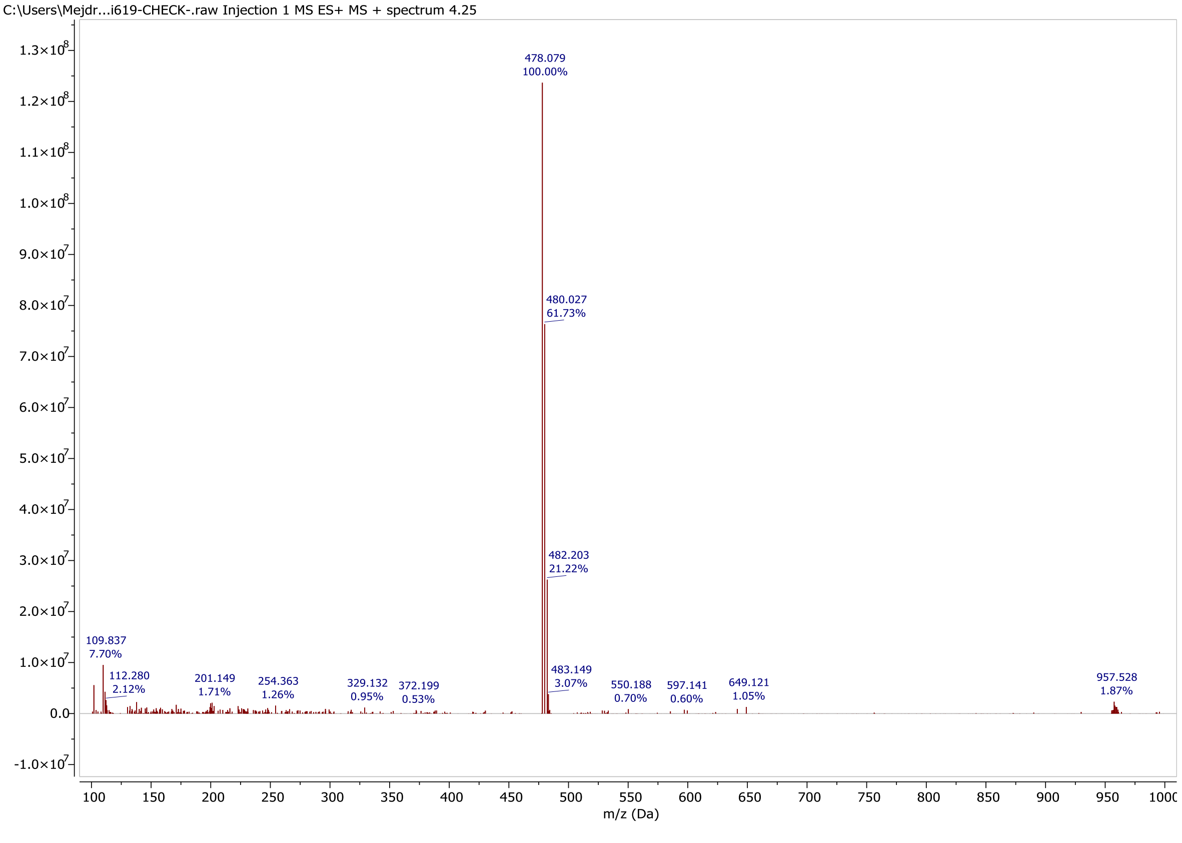

Compound **39**

C:\Users\Mejdr...MI676-solid.raw Injection 1 PDA - Chromatogram 254 - 256 nm

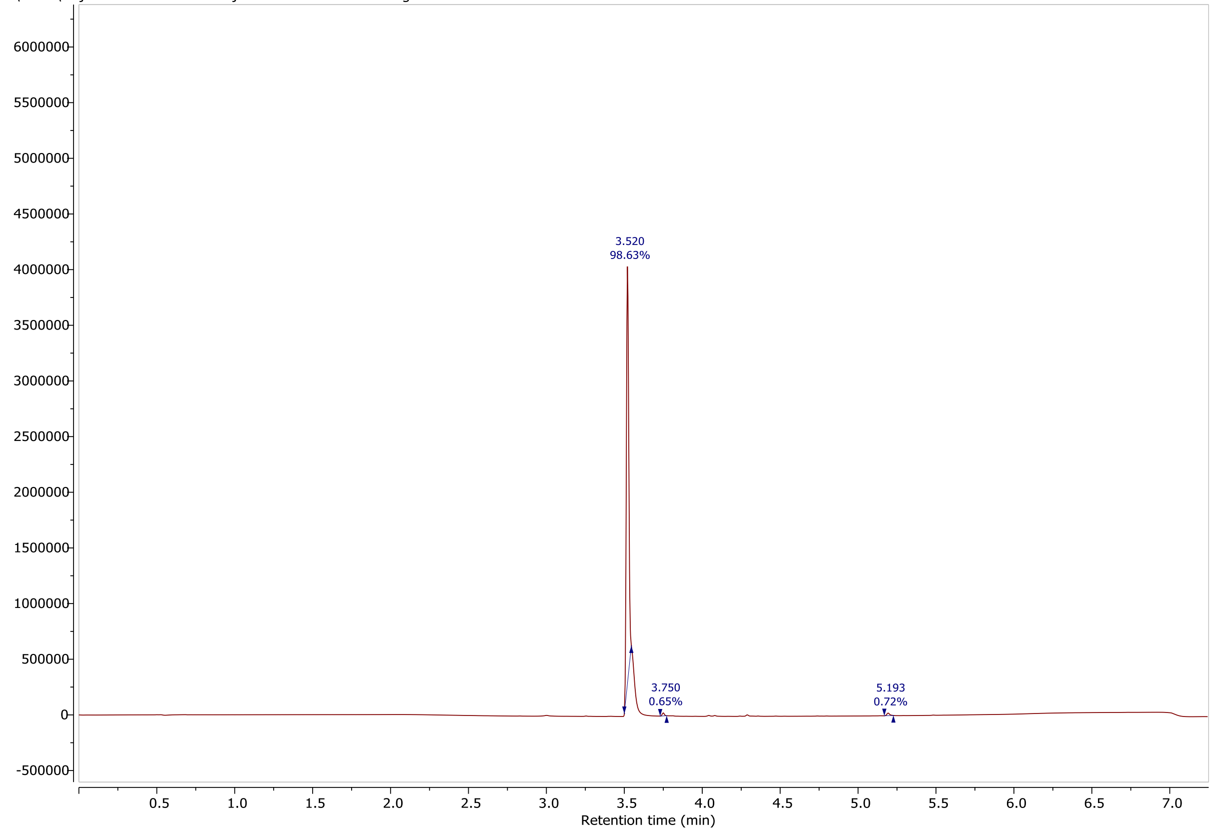

C:\Users\Mejdr...MI676-solid.raw Injection 1 MS ES+ MS + spectrum 3.52

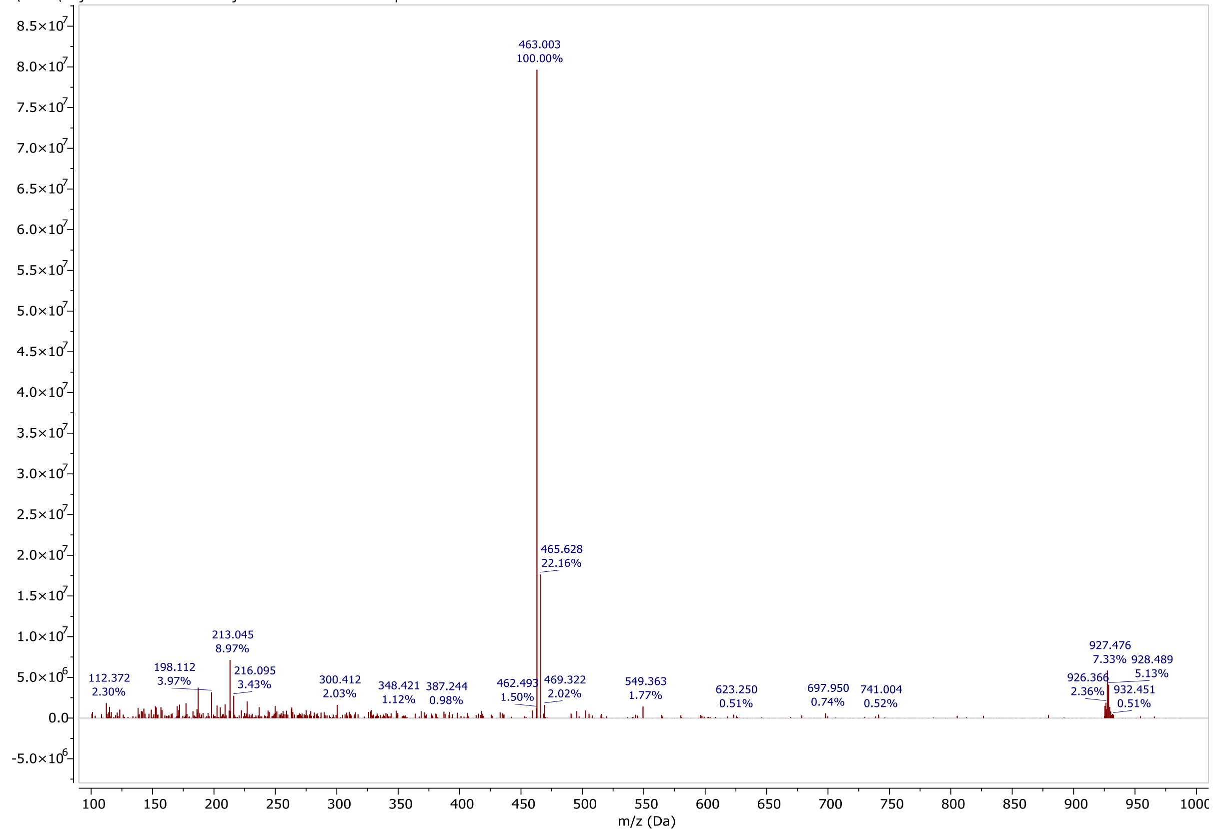

Compound **40**

C:\Users\Mejdr...sktop\MI693.raw Injection 1 PDA - Chromatogram 254 - 256 nm

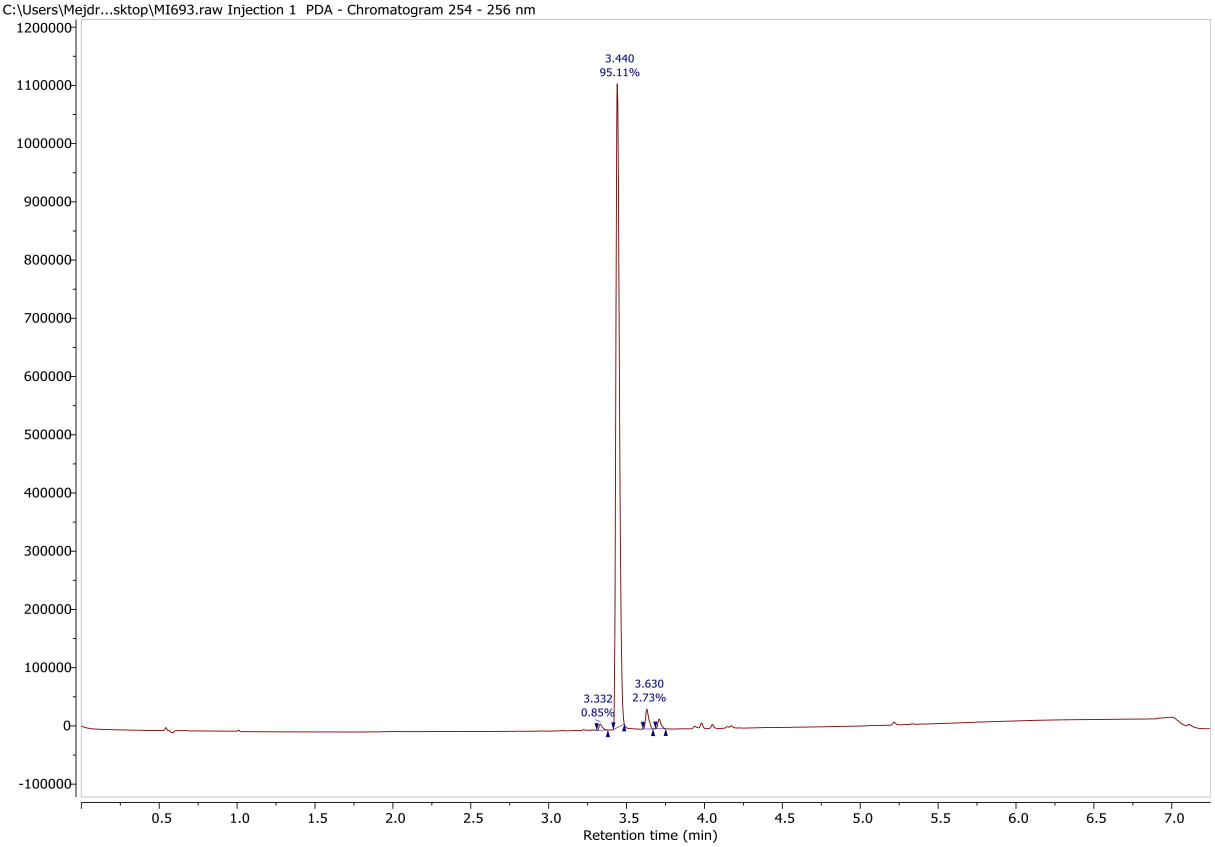

C:\Users\Mejdr...sktop\MI693.raw Injection 1 MS ES+ MS + spectrum 3.44

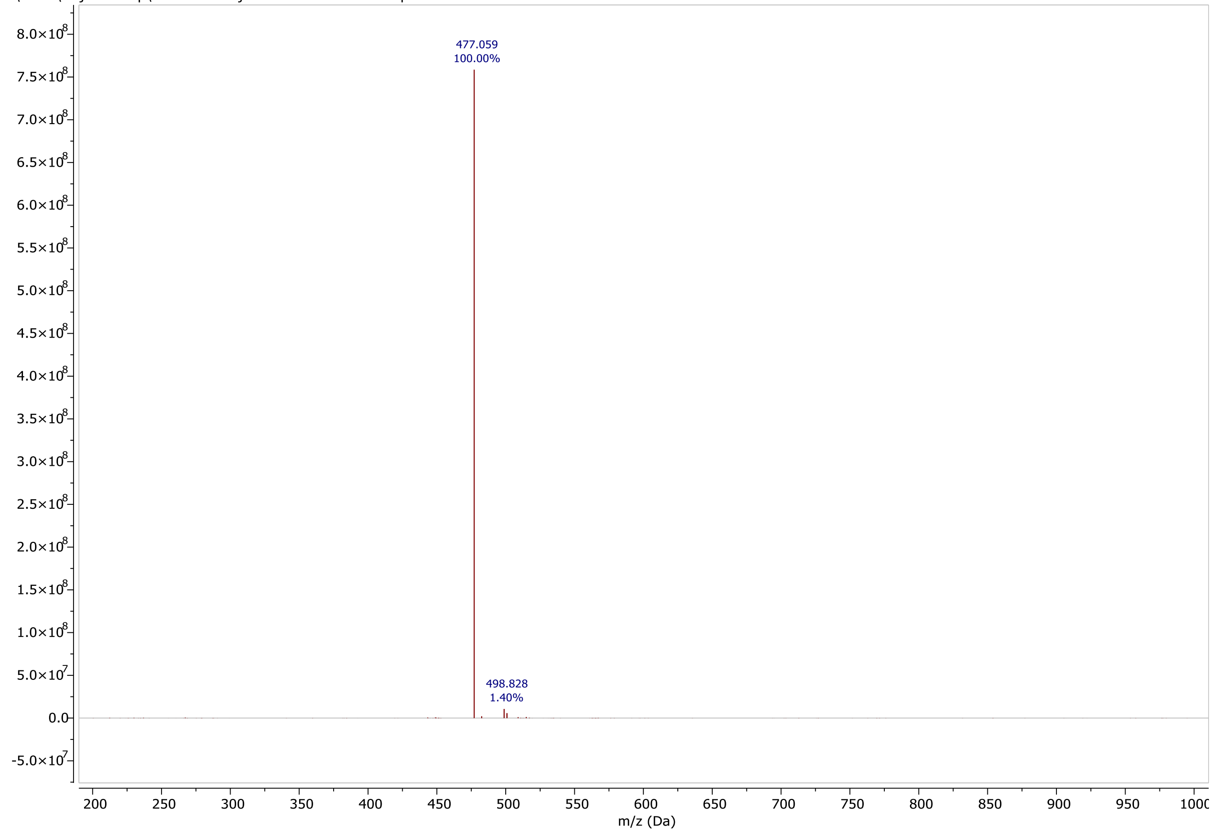

Compound **41**

C:\Users\Mejdr...ktop\MI677.raw\ Injection 1 PDA - Chromatogram 254 - 256 nm

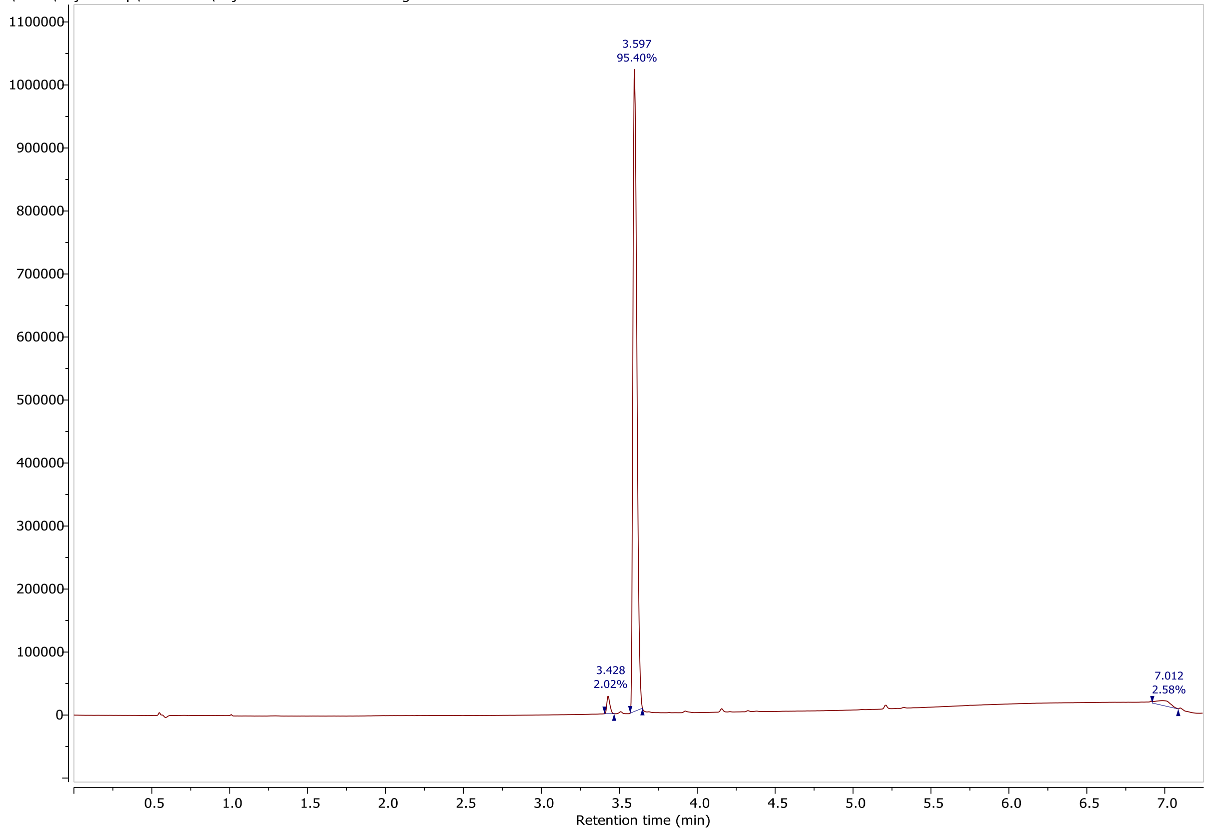

C:\Users\Mejdr...ktop\MI677.raw\ Injection 1 MS ES+ MS + spectrum 3.60

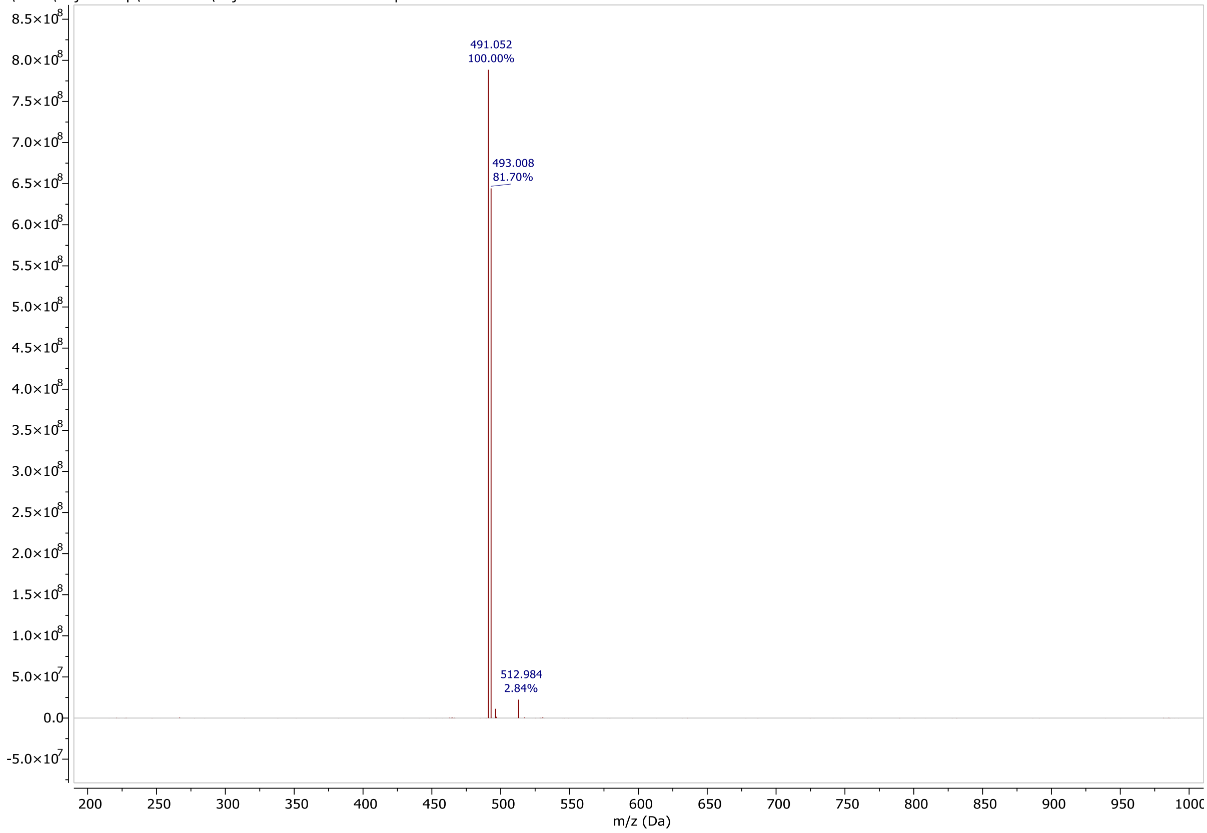

Compound **42**

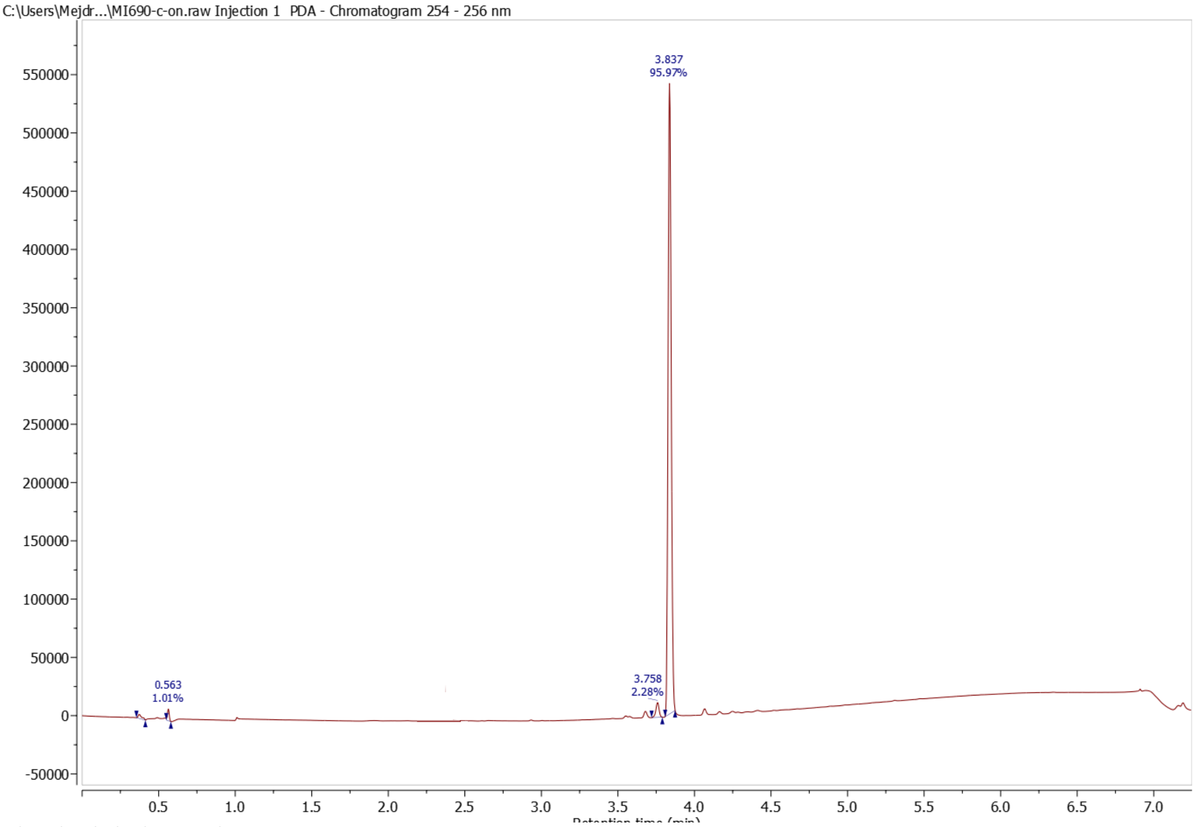
C:\Users\Mejdr...\MI690-c-on.raw Injection 1 PDA - Chromatogram 254 - 256 nm
3.837
95.97%
0.563
1.01%
3.758
2.28%

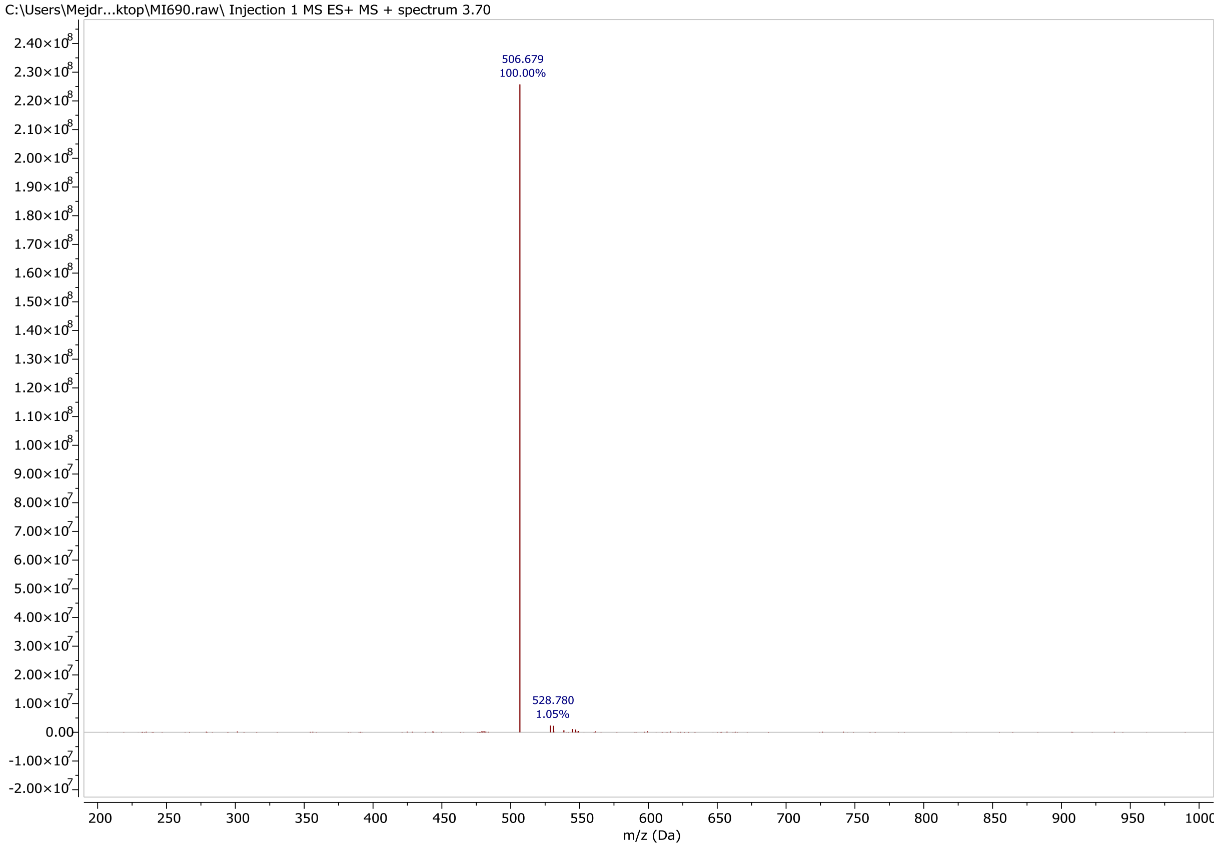
C:\Users\Mejdr...ktop\MI690.raw\ Injection 1 MS ES+ MS + spectrum 3.70
506.679
100.00%
528.780
1.05%
m/z (Da)

15. References

(1) Dusek, J.; Skoda, J.; Holas, O.; Horvatova, A.; Smutny, T.; Linhartova, L.; Hirsova, P.; Kucera, O.; Micuda, S.; Braeuning, A.; et al. Stilbene compound trans-3,4,5,4 -tetramethoxystilbene, a potential anticancer drug, regulates constitutive androstane receptor (Car) target genes, but does not possess proliferative activity in mouse liver. *Toxicology letters* **2019**, *313*, 1-10. DOI: 10.1016/j.toxlet.2019.05.024.

(2) Mejdrova, I.; Chalupska, D.; Plackova, P.; Muller, C.; Sala, M.; Klima, M.; Baumlova, A.; Hrebabecky, H.; Prochazkova, E.; Dejmek, M.; et al. Rational Design of Novel Highly Potent and Selective Phosphatidylinositol 4-Kinase III beta (PI4KB) Inhibitors as Broad-Spectrum Antiviral Agents and Tools for Chemical Biology. *Journal of Medicinal Chemistry* **2017**, *60* (1), 100-118. DOI: 10.1021/acs.jmedchem.6b01465. Mejdrova, I.; Chalupska, D.; Kogler, M.; Sala, M.; Plackova, P.; Baumlova, A.; Hrebabecky, H.; Prochazkova, E.; Dejmek, M.; Guillon, R.; et al. Highly Selective Phosphatidylinositol 4-Kinase III beta Inhibitors and Structural Insight into Their Mode of Action. *Journal of Medicinal Chemistry* **2015**, *58* (9), 3767-3793. DOI: 10.1021/acs.jmedchem.5b00499.

(3) Xu, R. X.; Lambert, M. H.; Wisely, B. B.; Warren, E. N.; Weinert, E. E.; Waitt, G. M.; Williams, J. D.; Collins, J. L.; Moore, L. B.; Willson, T. M.; et al. A structural basis for constitutive activity in the human CAR/RXRalpha heterodimer. *Mol Cell* **2004**, *16* (6), 919-928. DOI: 10.1016/j.molcel.2004.11.042.

(4) Trott, O.; Olson, A. J. Software News and Update AutoDock Vina: Improving the Speed and Accuracy of Docking with a New Scoring Function, Efficient Optimization, and Multithreading. *Journal of Computational Chemistry* **2010**, *31* (2), 455-461. DOI: 10.1002/jcc.21334.

(5) Pettersen, E. F.; Goddard, T. D.; Huang, C. C.; Couch, G. S.; Greenblatt, D. M.; Meng, E. C.; Ferrin, T. E. UCSF chimera - A visualization system for exploratory research and analysis. *Journal of Computational Chemistry* **2004**, *25* (13), 1605-1612. DOI: 10.1002/jcc.20084.

(6) Bowers, K. J.; Chow, E.; Xu, H.; Dror, R. O.; Eastwood, M. P.; Gregersen, B. A.; Klepeis, J. L.; Kolossvary, I.; Moraes, M. A.; Sacerdoti, F. D.; et al. Scalable Algorithms for Molecular Dynamics Simulations on Commodity Clusters. In 2006. https://doi.org/10.1145/1188455.1188544. *Proceedings of the 2006 ACM/IEEE Conference on Supercomputing; SC '06; ACM: New York, NY, USA,* **2006**. DOI: https://doi.org/10.1145/1188455.1188544. Roos, K.; Wu, C.; Damm, W.; Reboul, M.; Stevenson, J. M.; Lu, C.; Dahlgren, M. K.; Mondal, S.; Chen, W.; Wang, L.; et al. OPLS3e: Extending Force Field Coverage for Drug-Like Small Molecules. *J Chem Theory Comput* **2019**, *15* (3), 1863-1874. DOI: 10.1021/acs.jctc.8b01026.

(7) Jorgensen, W. L.; Chandrasekhar, J.; Madura, J. D.; Impey, R. W.; Klein, M. L.; . Comparison of Simple Potential Functions for Simulating Liquid Water. . *J. Chem. Phys.* **1983**, *79* (2), 926–935. . DOI: https://doi.org/10.1063/1.445869.

(8) Darden, T.; York, D.; Pedersen, L. Particle Mesh Ewald: An N·log(N) Method for Ewald Sums in Large Systems. . *J. Chem. Phys.* **1993**, *98* (12), 10089–10092. . DOI: https://doi.org/10.1063/1.464397.

(9) Law, S. M. PyMol Script: Modevectors.Py. 2012.

(10) Waskom, M. L. Seaborn: Statistical Data Visualization. . *Journal of Open Source Software* **2021**, *6* (60), 3021. . DOI: https://doi.org/10.21105/joss.03021.

(11) Hunter, J. D. Matplotlib: A 2D Graphics Environment. *Computing in Science Engineering* **2007**, *9* (3), 90–95. . DOI: https://doi.org/10.1109/MCSE.2007.55.

(12) Hill, J. R. In vitro drug metabolism using liver microsomes. *Curr Protoc Pharmacol* **2004**, *Chapter 7*, Unit7 8. DOI: 10.1002/0471141755.ph0708s23.
